# Anti–Programmed Death Ligand 1 Plus Targeted Therapy in Anaplastic Thyroid Carcinoma: A Nonrandomized Clinical Trial

**DOI:** 10.1001/jamaoncol.2024.4729

**Published:** 2024-10-24

**Authors:** Maria E. Cabanillas, Ramona Dadu, Renata Ferrarotto, Maria Gule-Monroe, Suyu Liu, Bryan Fellman, Michelle D. Williams, Mark Zafereo, Jennifer R. Wang, Charles Lu, Matthew Ning, Brian A. McKinley, Scott E. Woodman, Dzifa Duose, Gary B. Gunn, Naifa L. Busaidy

**Affiliations:** 1 Department of Endocrine Neoplasia and Hormonal Disorders, The University of Texas MD Anderson Cancer, Houston; 2 Department of Head and Neck Medical Oncology, The University of Texas MD Anderson Cancer, Houston; 3 Department of Neuroradiology, The University of Texas MD Anderson Cancer, Houston; 4 Department of Biostatistics, The University of Texas MD Anderson Cancer, Houston; 5 Department of Pathology, The University of Texas MD Anderson Cancer, Houston; 6 Department of Head and Neck Surgery, The University of Texas MD Anderson Cancer, Houston; 7 Department of Radiation Oncology, The University of Texas MD Anderson Cancer, Houston; 8 Department of Genomic Medicine, The University of Texas MD Anderson Cancer, Houston; 9 Department of Translational Molecular Pathology, The University of Texas MD Anderson Cancer, Houston

## Abstract

**Importance:**

Anaplastic thyroid carcinoma (ATC) is a rare and lethal cancer. Although progress has been made in recent years in patients with mutated BRAF tumors, those who respond initially eventually die of their disease; furthermore, there are no approved therapies for non-BRAF mutated tumors.

**Objective:**

To determine whether treatment with matched-targeted therapy plus immune checkpoint inhibitors were associated with improved overall survival (OS).

**Design, Setting, and Participants:**

A phase 2 trial at a single center, tertiary institution with parallel cohorts, assigning treatment with targeted therapy according to the tumor mutation status. Patients with mutated *BRAF* V600E tumors received vemurafenib/cobimetinib plus atezolizumab (cohort 1); those with mutated *RAS (NRAS, KRAS, or HRAS)* or *NF1/2* tumors received cobimetinib plus atezolizumab (cohort 2). Patients without any of these variants were assigned to receive bevacizumab plus atezolizumab (cohort 3). Patients were enrolled from August 3, 2017, to July 7, 2021. All consecutive, systemic therapy-naive patients with ATC with active disease and who met eligibility criteria were considered for participation. The analysis was conducted in September 2023.

**Interventions:**

Patients were assigned to targeted therapy based on the driver mutation as follow: *BRAF* V600E (cohort 1, vemurafenib plus cobimetinib), *RAS/NF* (cohort 2, cobimetinib), or non-*BRAF*/*RAS*/*NF* (cohort 3, bevacizumab). All received atezolizumab.

**Main Outcomes and Measures:**

The primary outcome of the study was median OS of the entire targeted therapy cohort, compared with historical median OS of 5 months.

**Results:**

Forty-three patients with ATC were enrolled in the targeted therapy cohorts, of which 42 were included in the primary analysis. The median OS in patients across these 3 cohorts was 19 months (95% CI, 7.79-43.24). Median OS and progression-free survival per cohort were as follows: cohort 1: 43 months (95% CI, 16-not estimable [NE]), 13.9 months (6.6-64.1); cohort 2: 8.7 months (95% CI, 5.1-37.0) and 4.8 months (1.8-14.7); cohort 3 (vascular endothelial growth factor inhibitor group): 6.21 months (4.1-NE) and 1.3 months (1.3-NE), respectively.

**Conclusions and Relevance:**

In this nonrandomized clinical trial, atezolizumab combined with targeted therapy resulted in a longer median OS than historical landmark, achieving the study’s primary end point, with cohort 1 achieving the longest OS.

**Trial Registration:**

ClinicalTrials.gov Identifier: NCT03181100

## Key Points

**Question:**

What is the efficacy of combination targeted therapy plus checkpoint inhibition in patients with anaplastic thyroid cancer (ATC)?

**Findings:**

In this nonrandomized, phase 2 trial of 42 patients with ATC assigned to genetically-matched targeted therapy plus immune checkpoint inhibitors, there were 3 genetically-matched targeted therapy cohorts (*BRAF* V600E variant: vemurafenib/cobimetinib; RAS or NF variant: cobimetinib; non-BRAF/RAS/NF variant: bevacizumab). All patients received the immune checkpoint inhibitor, atezoliuzumab.

**Meaning:**

The trial met its primary end point, and demonstrated the longest overall survival published to date for systemic therapy in patients with ATC, and should be studied further.

## Teaser

This nonrandomized clinical trial examines whether matched-targeted therapy based on tumor mutation status with a PD-L1 inhibitor is associated with improved overall survival among patients with anaplastic thyroid carcinoma.

## Introduction

Anaplastic thyroid cancers (ATC) are one of the deadliest tumors in humans for a number of reasons including (1) rapid tumor growth located in the neck causes respiratory compromise and inability to swallow, (2) delay in diagnosis is common because these are rare tumors, (3) the invasiveness of the locoregional disease results in most being inoperable at diagnosis, (4) the high rate of metastatic disease at diagnosis, and (5) patients are usually of advanced age at diagnosis and have multiple comorbidities. The median overall survival (OS) in ATC was historically 5 months.^1^

ATC is derived from the different subtypes of differentiated thyroid cancer (DTC), which have distinct driver mutations. Only one of these mutations—*BRAF* V600E—has been shown to be actionable at this time. In 2018, the Food and Drug Administration (FDA) approved the combination of the BRAF inhibitor dabrafenib with the MEK inhibitor trametinib for *BRAF* V600E–mutated ATC, which has an incidence of approximately 40%.^2,3^ Unfortunately, there are no approved and effective therapies for non-BRAF mutated ATC. Clinical trials with single-agent targeted therapy have shown limited efficacy.^4,5,6^ In the only single-agent immunotherapy trial with a programed death ligand 1 (PD-L1) inhibitor in ATC, the response rate (RR) and median OS were 19% and 5.9 (95% CI, 2.4-not estimable [NE]) months, respectively.^7^

In this prospective clinical trial, we treated patients with ATC based on their tumor mutation status, combining matched-targeted therapy with a PD-L1 inhibitor (atezolizumab). The rationale for this combination strategy was based on the observation that checkpoint inhibitors result in a slower response when compared with targeted therapy; therefore, in a tumor type with rapid locoregional progression, it was important to find a strategy that would have a faster onset of action. Given that development of bypass variants is known to be a mechanism of resistance to targeted therapy, adding immunotherapy, which has a completely different mechanism of action, was the strategy we chose. Furthermore, there are data to suggest that targeted agents may change the tumor immune environment and potentially synergize with immunotherapy.^8,9^ We hypothesized that combining a PD-L1 inhibitor (atezolizumab) with targeted therapy would lead to improved OS in patients with ATC.

## Methods

**Figure 1.**
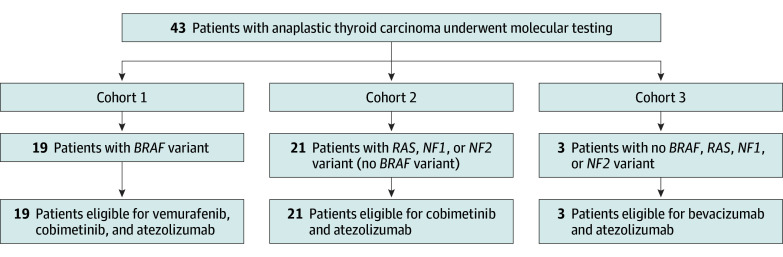
Schema of Trial Design All patients received atezolizumab as a part of their regimen, in addition to targeted therapy, assigned based on the driver mutation in their tumor. If patients were not eligible for their tumor mutation-determined cohort, they could be screened for another targeted-therapy cohort 1 to 3. If they were not eligible for cohorts 1 to 3 they were assigned to cohort 4, paclitaxel-based therapy with atezolizumab. The primary end point was based on the targeted-therapy cohorts 1 to 3.

The trial protocol (Supplement 1) and amendments were approved by the University of Texas MD Anderson Cancer Center institutional review board and each patient provided written informed consent prior to initiating protocol-specific studies and treatments. We designed a phase 2 trial with parallel cohorts at a single institution, assigning treatment with targeted therapy according to the tumor mutation status (Figure 1). We included patients with ATC with unresectable, locoregionally advanced disease and/or distant metastases. The Response Evaluation Criteria in Solid Tumors (RECIST) measurable target lesions were not required in patients with ATC (but they had to have active disease), as the primary end point was OS. Eastern Cooperative Oncology Group (ECOG) performance status of 2 or lower and adequate organ function were required for entry. We excluded patients who had received previous anti-PD1 or PD-L1 antibody or pathway-targeting agents. The use of corticosteroids was not allowed for 10 days prior to initiation of atezolizumab except patients who were taking steroids for physiological replacement. We did not exclude patients with prior cancer history because many patients with ATC are of advanced age and have been reported to have a history of other cancers.^10^ An induction phase with paclitaxel at 80 mg/m^2^ or nab-paclitaxel at 125 mg/m^2^ administered weekly for up to 3 doses was permitted, serving as a bridge to mutation-driven treatment assignment. Other entry criteria are listed in eMethods in Supplement 2. Patients who achieved sufficient response to therapy were allowed to undergo surgery with or without radiation to control their locoregional disease throughout the duration of the trial. Patients were permitted to remain in the study past progression if, in the opinion of the treating physician, they were felt to be benefitting from the trial therapy.

Treatment allocation was determined by the presence of a tumor mutation detected on any Clinical Laboratory Improvement Amendments–certified mutation panel. Patients with a *BRAF *V600E mutation received vemurafenib/cobimetinib plus atezolizumab (cohort 1) and those with *RAS (NRAS, KRAS, or HRAS)* or *NF1/2* driver mutations received cobimetinib plus atezolizumab (cohort 2). Patients without any of these mutations were assigned to receive the vascular endothelial growth factor (VEGF) inhibitor bevacizumab plus atezolizumab (cohort 3), or if they had contraindications for the use of VEGF inhibitors (eg, high risk of bleeding or fistula), they were assigned to chemotherapy with a paclitaxel-based regimen plus atezolizumab (exploratory cohort 4). Doses and schedules for each of these regimens are listed in the eMethods and eFigure 1 in Supplement 2. Patients who could not swallow whole pills were allowed to crush vemurafenib and/or were provided with cobimetinib suspension (instructions in the eMethods in Supplement 2).

The primary study objective was to determine if targeted therapy in combination with atezolizumab (cohorts 1-3) would lead to improved OS in patients with ATC, compared with historical controls. Secondary objectives included overall response rate (ORR) by RECIST v1.1, OS, and progression-free survival (PFS) in cohorts 1 to 3 as well as ORR, OS, and PFS in each cohort. Exploratory cohorts included patients receiving chemotherapy plus atezolizumab and those with poorly differentiated thyroid cancer and are not reported here.

OS and PFS were estimated using the Kaplan-Meier method. Median OS and PFS and 1- and 2-year survival probabilities were estimated with 95% CIs. For the primary end point, with a minimal sample size of 36 patients, a 33-month accrual period and 3-month after accrual follow-up, the power was 90% to detect an observed 9-month median OS vs a historical 5-month OS with a 1-sided 5% level of significance. Power calculations were performed using the DSTPLAN program (version 4.5; copyright 2000 for The University of Texas MD Anderson Cancer Center Department of Biomathematics). The data cutoff was August 16, 2023.

We monitored for futility continuously using a formal Bayesian monitoring plan to ensure patients were not receiving an ineffective treatment. Details of this plan are outlined in the eMethods in Supplement 2. Descriptive statistics were used to describe patient characteristics. Frequencies and percentages were used to describe the categorical variables and means with standard deviates, medians, and ranges for continuous variables. 95% CIs were used to describe objective response. Adverse events were graded per National Cancer Institute Common Terminology Criteria for Adverse Events 2 (NCI CTCAE; version 4.0) and summarized with frequencies and percentages. All statistical analysis were performed using Stata/MP statistical software (version 16.0; Stata Corp). Analyses were performed in September 2023.

## Results

Between August 3, 2017, and July 7, 2021, a total of 43 patients with ATC were enrolled in cohorts 1 to 3 (details in eFigure 2 in Supplement 2). Nineteen were assigned to cohort 1 (BRAF), 21 to cohort 2 (RAS, NF), and 3 to cohort 3 (VEGFi). One patient in cohort 1 withdrew consent before starting atezolizumab, resulting in 42 evaluable in cohorts 1 to 3. At the time of data cutoff, 29 patients (69%) had died, and 13 (31%) were alive. Patient demographics are summarized using the descriptive statistics in Table 1. The 1 patient in cohort 1 with stage IVA ATC at diagnosis had developed biopsy-proven metastatic ATC to the lungs at the time of study entry. Six of 39 patients (15%) assigned to cohorts 1 and 2 required alternative drug administration due to inability to swallow pills.

**Table 1.** Baseline Demographics on Patients With Anaplastic Thyroid Cancer (ATC) by Cohort

Characteristic	No. (%)		
	Cohort 1 (BRAF)	Cohort 2 (MEK)	Cohort 3 (VEGF)
No.	18	21	3
Age, median (range), y	66 (44-80)	66 (46-83)	51 (47-69)
Race			
Asian	0	1 (4.76)	0
Black	1 (5.56)	0	0
White	15 (83.33)	20 (95.24)	3 (100)
Other	2 (11.11)	0	0
Ethnicity			
Hispanic/Latino	3 (16.67)	2 (9.52)	0
Non-Hispanic	15 (83.33)	19 (90.48)	3 (100)
Sex			
Female	8 (44.40)	12 (57.14)	2 (66.67)
Male	10 (55.60)	9 (42.86)	1 (33.33)
Stage at diagnosis			
IVA	1 (5.56)	0	0
IVB	3 (16.67)	7 (33.33)	1 (33.33)
IVC	14 (77.78)	14 (66.67)	2 (66.67)
Distant metastases at start of trial			
No	5 (27.78)	2 (9.52)	0
Yes	13 (72.22)	19 (90.48)	3 (100)
Previous therapy for primary ATC tumor			
Neck radiation	1 (6)	4 (19)	0
Neck radiation with chemotherapy	2 (11)	12 (57)	2 (67)
None	15 (83)	5 (24)	1 (33)
Bridging chemotherapy			
No	16 (88.89)	20 (95.24)	2 (66.67)
Yes	2 (11.11)	1 (4.76)	1 (33.33)
Alternative drug administration			
No	16 (89)	17 (81)	NA
Yes	2 (11)	4 (19)	NA
ECOG			
0	10 (55.56)	9 (42.86)	3 (100)
1	8 (44.44)	11 (52.38)	0
2	0	1 (4.76)	0

Abbreviations: BRAF, BRAF-directed therapy with vemurafenib, cobimetinib, and atezolizumab; ECOG, Eastern Cooperative Oncology Group; MEK, MEK inhibitor therapy with cobimetinib and atezolizumab; VEGF, vascular endothelial growth factor inhibitor therapy with bevacizumab and atezolizumab; NA, not applicable.

**Figure 2.**
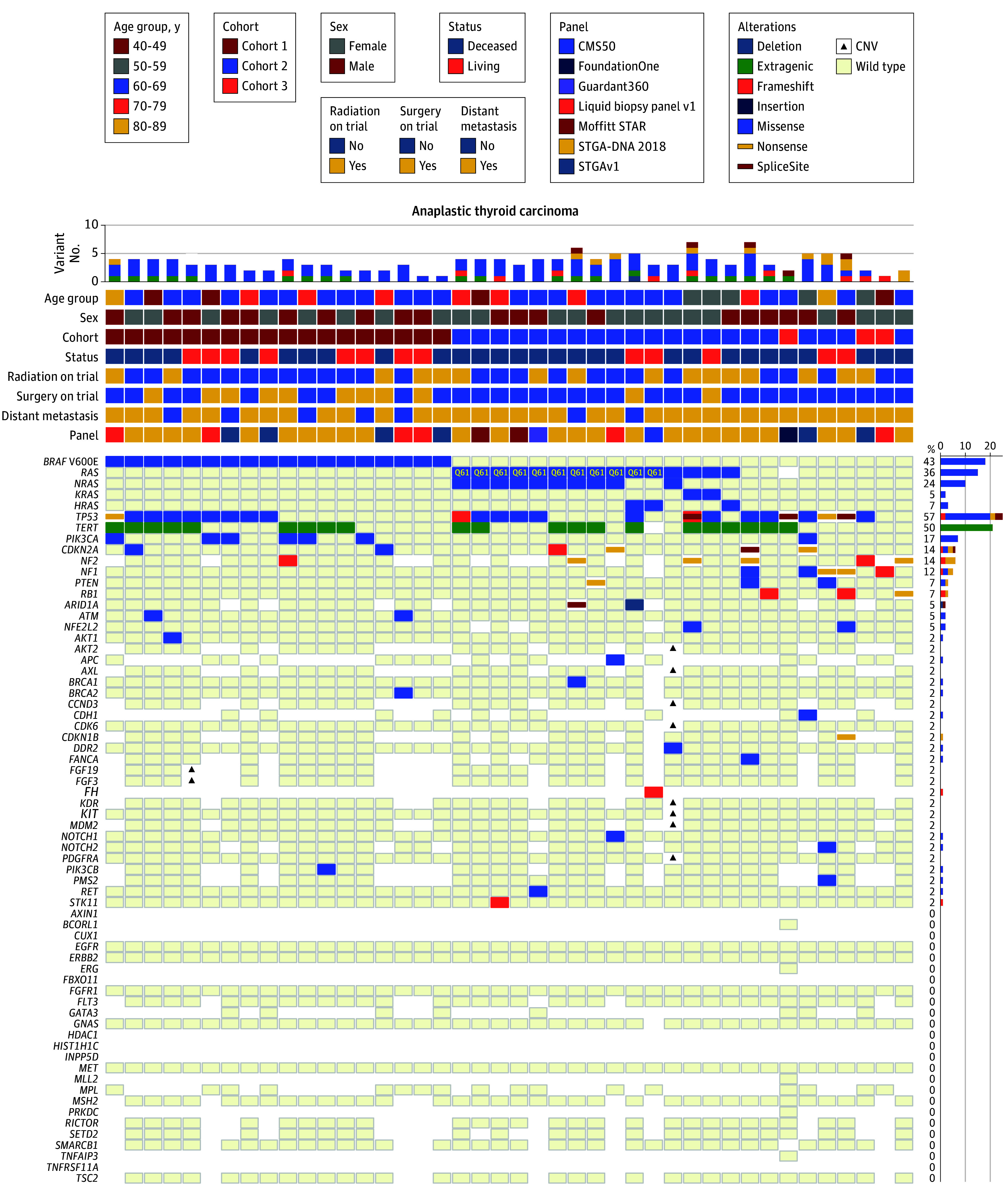
Oncoprint of Targeted Somatic Gene Variants of Tumors in Patients With Anaplastic Thyroid Cancer (ATC) Enrolled in Cohorts 1 to 3 Several Clinical Laboratory Improvement Amendments–certified panels were used to assay for mutations in oncogenes of ATC tumors, including liquid biopsy (shown in figure). The data shown represent the clinical sequencing conducted at or before the start of treatment. Genes that were wild type across all samples were removed from the analysis. The white space represents instances in which a gene was not assayed in a specific panel. CNV indicates copy number variation.

All patients underwent mutation testing as per standard of care at our institution for ATC. Although most had tumor tissue–targeted sequencing performed, some only had liquid biopsy (LB) where tumor materials were unavailable. The oncoprint of the mutations in all patients with ATC assigned to cohorts 1 to 3, and the test performed on each patient can be found in Figure 2.

### Efficacy of Anti–PD-L1 Plus Targeted Therapy in ATC

**Figure 3.**
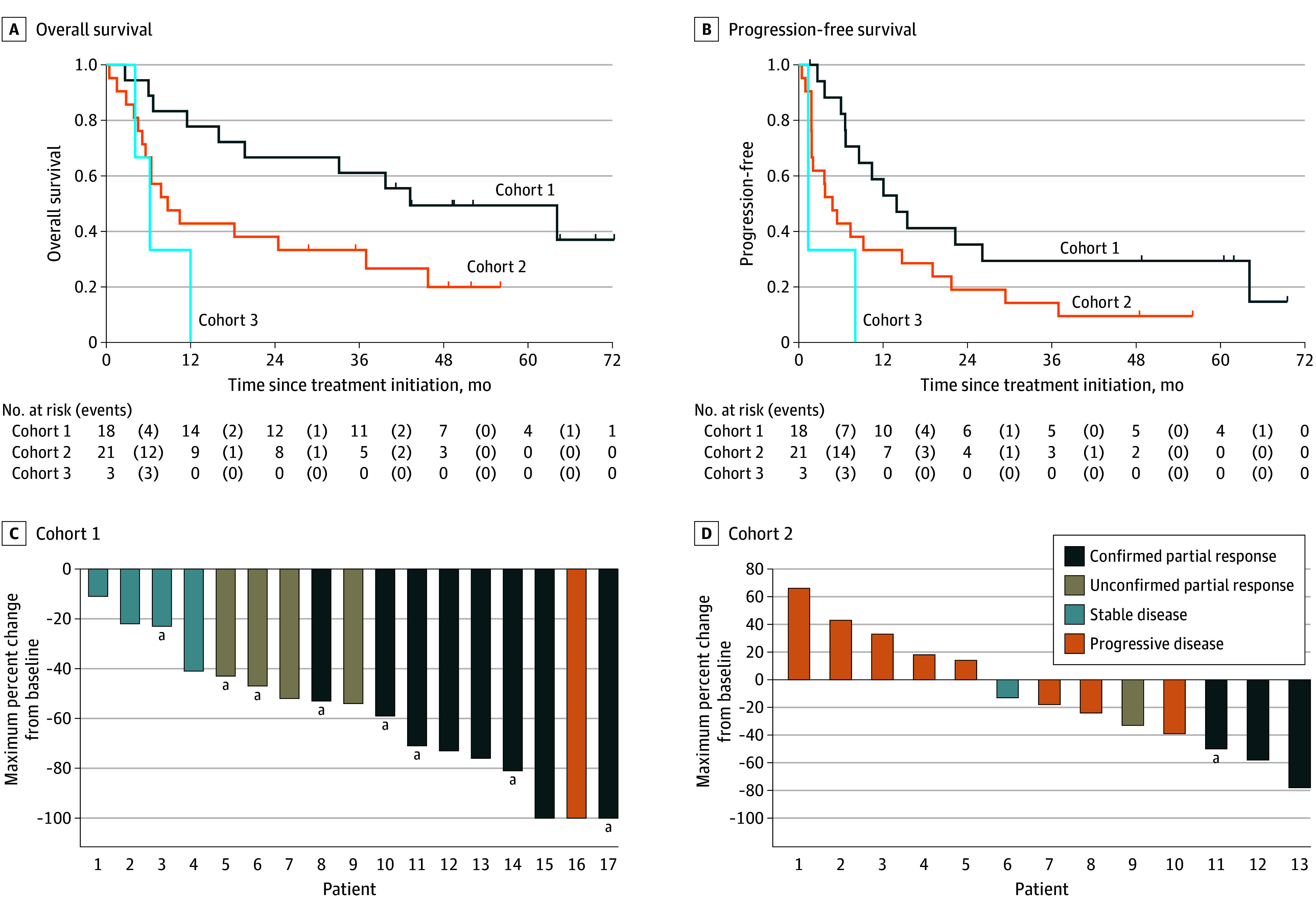
Kaplan-Meir Curves for Overall Survival and Progression-Free Survival in Patients With Anaplastic Thyroid Cancer (ATC) Per Cohort A, Overall survival curves for cohorts 1 to 3. B, Progression-free survival curves for cohorts 1 to 3. C and D, Waterfall plots are shown for the Response Evaluation Criteria in Solid Tumors (RECIST; version 1.1)–evaluable patients with ATC in cohorts 1 and 2, respectively. Best percent change from baseline in the target lesions in 30 patients with ATC are shown (17 in cohort 1 and 13 in cohort 2). One patient in cohort 1 had a large, nonmeasurable thyroid mass that was later resected and the patient is alive and disease-free. Six patients in cohort 2 had nonmeasurable disease, 1 died early before restaging, and 1 was not adequately restaged prior to surgical resection of their primary tumor (due to kidney insufficiency, the patient was not given contrast media; the patient is alive and disease-free). ^a^Patients who underwent surgical resection of the primary tumor. Two patients (both alive at data cutoff) in cohort 1 had surgical resection of the primary tumor before a partial response was confirmed and therefore were counted as stable disease in the overall response rate.

A summary of efficacy end points for patients with ATC in all cohorts and cohorts 1 to 3 is listed in Table 2. The Kaplan-Meier method was used to estimate the OS and PFS. The percentage of ORR was reported, and the frequency table for best response was provided as well. The median OS for patients with ATC in cohorts 1 to 3 (primary end point) was 18.23 months (95% CI, 7.79-43.24), with a median follow-up time of 18.97 months (95% CI, 0.43-72.11). The median OS for cohort 1 was 43.24 months (95% CI, 16-NE), median PFS was 13.93 months (95% CI, 6.60-64.13) with a median follow-up time of 42.14 months (95% CI, 2.66-72.11). The median OS for cohort 2 was 8.74 months (95% CI, 5.13-36.96), median PFS 4.80 months (95% CI, 1.84-14.69), and median follow-up time 8.74 months (95% CI, 0.43-55.92). Kaplan-Meier curves for all patients (ATC) in cohorts 1 to 3 are shown in Figure 3, A and B.

**Table 2.** Efficacy in Patients With Anaplastic Thyroid Cancer by Cohort

Variable	No (%)			
	Cohort 1 (BRAF)	Cohort 2 (MEK)	Cohort 3 (VEGF)	Cohorts 1 to 3 (primary end point)
Total, No.	18	21	3	42
Overall response rate, %	50	14	33	31
Best response				
CR	1 (5.6)	0	0	1 (2.0)
cPR	8 (44.4)	3 (14.0)	1 (33.0)	12 (28.5)
uPR	4 (22.2)	0	0	4 (9.5)
SD	4 (22.2)	3 (14.0)	0	7 (17.0)
PD	0	9 (43.0)	2 (67.0)	11 (26.0)
Non-CR, non-PD^a^	1 (5.6)	4 (19.0)	0	5 (12.0)
NE	0	2 (10)	0	2 (5)
OS, median (95% CI), mo	43.24 (16-NE)	8.74 (5.13-36.96)	6.21 (4.11-NE)	18.23 (7.79-43.24)^b^
PFS, median (95% CI), mo	13.93 (6.6-64.13)	4.80 (1.84-14.69)	1.35 (1.35-NE)	7.98 (2.43-12.88)
Follow-up, median (95% CI), mo	42.14 (2.66-72.11)	8.74 (0.43-55.92)	6.21 (4.11-11.99)	18.97 (0.43-72.11)

Abbreviations: BRAF, BRAF-directed therapy with vemurafenib, cobimetinib, and atezolizumab; CR, complete response; cPR, confirmed partial response; MEK, MEK inhibitor therapy with cobimetinib and atezolizumab; NE, not estimable; OS, overall survival; PD, progressive disease; PFS, progression-free survival; SD, stable disease; uPR, unconfirmed partial response; VEGF, vascular endothelial growth factor inhibitor therapy with bevacizumab and atezolizumab.

^a^ Non-CR, Non-PD are designations in the RECIST 1.1 that refer to patients who do not have measurable target lesions per the accepted criteria. uPR was considered SD for the purpose of overall response rate.

^b^ Denotes the primary end point of the study.

The ORR for individual cohorts was 50% (BRAF cohort 1) and 14% (RAS/NF cohort 2). The waterfall plots for cohorts 1 and 2 are shown in Figure 3, C and D. Two patients (both alive at data cutoff) in cohort 1 had surgical resection of the primary tumor before a partial response was confirmed and therefore were counted as SD in the ORR analysis. Fifteen patients with ATC (36%) in cohorts 1 to 3 received treatment for longer than 12 months, and 20 (48%) were alive at 2 years, as shown on a swimmer plot in eFigure 3 in Supplement 2.

As a result of treatment during the clinical trial, 12 of the patients with ATC had locoregional tumors that became resectable. All except 1 of these patients who were considered resectable after treatment underwent surgery during the clinical trial (cohort 1: n = 9, cohort 2: n = 2), of which 8 of 11 (73%)are still alive. Four (36%) were stage IVB and 8 (72%) were stage IVC. Two patients required temporary tracheostomies as a result of surgery and/or postoperative radiation. None of these patients required a permanent tracheostomy or laryngectomy. The 1 patient who did not have surgery had poor performance status and progressed soon after the time that their tumor became resectable. The surgical pathologic findings showed no viable ATC in 9 of 11 (81%) of the tumor specimens.

### Biomarker Analysis

Of patients who had tissue available for PD-L1 expression, 27 of 30 (90%) had a PD-L1 score of 5% or higher by tumor proportion score (TPS). There was no correlation between PD-L1 expression with OS; however, it is notable that 3 of the 4 patients who had less than 5% PD-L1 expression progressed early (between course 2 and 4 of treatment).

At progression, a liquid biopsy and optional biopsy were planned. In cohort 1, there were 8 patients who progressed on study drug or died early due to progression. Of these, only 2 were found to have a new mutation: 1 NRAS Q61K and the other TERT promoter −124 C. However, it is interesting to note that 5 of 8 (63%) of these patients had mutations along the PI3K/Akt/mTOR pathway at baseline: 3 patients with PIK3CA (1 with N345K, 1 with E545K, and 1 patient with both E545K and H1047R), 1 mTOR (E1442Q), and 1 AKT1 (E17K) mutations.

### Adverse Events

eTable 1 in Supplement 2 shows the most common adverse events for all cohorts. There was 1 death that was possibly related to the trial participation due to colonic perforation in cohort 1. There was also a grade 2 colonic perforation in cohort 2. Other notable serious adverse events included colitis (1 grade 3 in cohort 1 and 1 grade 1 in cohort 2), papilledema due to optic nerve neuritis (1 case of grade 3 in cohort 1), retinopathy (grade 1; 1 case in cohort 1), left ventricular dysfunction/decreased ejection fraction (2 cases of grade 3 in cohort 2), pneumonitis (1 case of each: grade 2 in cohort 2 and grade 1 in cohort 4), pancreatitis (2 grade 2 cases in cohort 1), and esophageal perforation (1 grade 2 case in cohort 3).

## Discussion

ATC is an aggressive cancer that is now better understood to be the end stage of dedifferentiation from differentiated thyroid cancer.^11^ Primary resistance to single-agent targeted therapy is common in ATC,^4,5,6^ given multiple co-mutations along different signaling pathways. Despite success with BRAF and MEK inhibitors,^12^ specifically for BRAF mutation, inevitably resistance occurs.^13^ Recognizing that these tumors have oncogenic driver mutations, but that invariably develop resistance to targeted therapy alone, we designed this novel trial combining atezolizumab with targeted therapy, hypothesizing that this approach would result in a significantly longer median OS, compared with historical controls. Targeted therapy was assigned by driver mutations into 3 cohorts: vemurafenib/cobimetinib for *BRAF* V600E mutations, cobimetinib for RAS or NF mutations, and VEGF inhibitor for non-BRAF/RAS-NF mutations. The genetically-matched targeted therapy plus immunotherapy combination strategy across cohorts 1 to 3 resulted in an OS of 19 months—to our knowledge the longest OS reported to date in an ATC clinical trial, achieving the primary objective of the study.

Responses and OS were particularly promising in cohort 1 with vemurafenib plus cobimetinib combined with atezolizumab in *BRAF *V600E–mutated tumors, resulting in an ORR of 50%, median PFS of 13.93 (95% CI, 6.6-64.1), and median OS of 43.24 (3.5 years; 95% CI, 16.0-NE) months. This study was designed prior to the FDA approval of a similar BRAF/MEK inhibitor combination, dabrafenib/trametinib, which resulted in an ORR of 56%, median PFS of 6.7 months, and median overall survival of 14.5 months.^12^ Although it is not possible to directly compare these studies, our findings exceeded the survival with dabrafenib/trametinib in BRAF mutant ATC by more than 2 years. It should be noted that in our study, the ORR was lower than expected because 2 patients underwent surgical resection of the primary tumor were considered unconfirmed PRs. In a retrospective trial of the doublet of dabrafenib/trametinib vs the triplet of dabrafenib/trametinib/pembrolizumab, the median OS was significantly longer with the triplet (17.0 months; 95% CI, 11.9-22.1) compared with the doublet (9.0 months; 95% CI, 4.5-13.5; *P* = .04).^14^ A single agent trial with spartalizumab (anti–PD-1) resulted in an ORR of 19%, median PFS of 1.7 (95% CI, 1.2-1.9) months, and median OS of 5.9 (95% CI, 2.4-NE) months. In the spartalizumab trial, survival in patients with tumoral expression of PD-L1 was significantly higher (1 year survival of 52%) than those who did not express PD-L1 (median OS of 1.6 months; 95% CI, 1.0-19.6).^7^

Importantly, of the tumor collected at progression in cohort 1, we found 2 patients to have new tumor mutations that were not present at baseline: NRAS and TERT promoter. Resistance mechanisms in patients with ATC treated with BRAF inhibitors remain an area of active investigation; however, this particular RAS mutation has been previously reported as a mechanism of resistance in a patient with papillary thyroid cancer^13^ and an individual with ATC.^15^ These tumors could be using this alternate pathway as a bypass to the inhibition of the MAPK pathway.

In cohort 2, which included patients with RAS- and NF-mutated tumors, the median OS was shorter than in cohort 1, at 8.74 (95% CI, 5.13-36.96) months, comparable to the OS in clinical trial with single agent spartalizumab (median OS 5.9 months). It is not clear if cobimetinib single agent adds efficacy to PD-1/PD-L1 inhibitors and therefore further research must continue in these patients. Presumably, these patients have MAP kinase output, which is likely not completely blocked by the commercially available MEK inhibitors. Thus, better RAF inhibitors, which are currently in clinical trials (NCT04913285, NCT03284502, NCT05557045, NCT04985604, NCT04249843, NCT05907304), should be studied in RAS- and NF-mutated ATC. These trials will hopefully pave the way to combination strategies in ATC. Another strategy is the use of the multikinase inhibitor lenvatinib, which targets VEGF, with pembrolizumab, an anti–PD-1 drug. Cohort 3, which used a VEGF-directed therapy closed early in our trial; however, lenvatinib is a more potent inhibitor of the VEGF pathway than bevacizumab. Lenvatinib plus the anti–PD-1 pembrolizumab is currently being studied in the US (NCT04171622) but efficacy preliminary results have already been reported in a German trial.^16^ These investigators demonstrated a response rate of approximately 52% and median OS of 11 months. Given the bleeding and fistula risk with potent VEGF-directed therapy, these drugs may not be appropriate for all patients with ATC.

In terms of safety, the adverse event and serious adverse event profile were expected with these combinations. There was only 1 event—colonic perforation—that led to the death of a patient in cohort 1. The likely cause was the immunotherapy, although colonic perforation has also been described with BRAF inhibitors.

The findings of this nonrandomized clinical trial likely reflect a cohort of patients with ATC who would be treated in clinical practice because we allowed enrollment of patients who were unable to swallow in the cohorts that required oral administration of targeted therapy. Patients in cohorts 1 and 2 who could not swallow whole pills or capsules were permitted to use cobimetinib in suspension or allowed to crush the vemurafenib and place in ascorbic acid containing food. This is an important difference to point out when comparing clinical trials, particularly when considering how to design trials for a rare population of patients. For example, the trial that led to the approval of dabrafenib/trametinib excluded patients who could not swallow whole pills or capsules. The spartalizumab trial only enrolled patients with ECOG performance status of 0 to 1. Our trial also allowed bridging chemotherapy during the screening process, given the potential for rapid progression and deterioration of performance status in patients with ATC while awaiting molecular testing results. More permissive entry criteria also allowed us to enroll 52 patients over nearly 4 years at a single institution, which is remarkable, given that, historically, most ATC trials have failed to complete enrollment. Insisting on sensible trial designs for patients with ATC is essential for these trials to translate to the clinical setting and allow for timely completion of future studies in this rare cancer.

### Limitations

There are some limitations of our study. First, there was no control arm, which is a universal challenge in rare tumors, particularly when considering that they were treated according to the driver mutation. Second, we allowed surgery to remove the primary tumor, as well as radiation in this trial, which could have contributed to the improvement in survival; however, patients were unresectable at baseline and were only able to undergo resection of primary tumor due to favorable response to treatment. Retrospective studies have shown that removal of the primary tumor may improve OS in ATC^2,17,18^ and therefore this has become our standard. It is remarkable that so many patients were able to undergo larynx-sparing surgery after responding to the treatment administered. Although local therapy may have skewed the OS analysis, our reported unprecedent survival outcome argues that future clinical trials in patients with ATC should be designed to allow for surgery because it likely improves OS—the goal for patients. Neoadjuvant dabrafenib/trametinib combined with pembrolizumab followed by surgery with or without radiation is being explored in a prospective, multicenter clinical trial (NCT04675710). However, this sequential approach requires critical sequential treatment decisions to be made by a team of multidisciplinary physicians with expertise in the treatment and care of patients with ATC, which may be challenging to implement in many standard-of-care settings.

## Conclusion

This nonrandomized clinical trial found that mutation-directed targeted therapy in combination with PD-L1 inhibitor immunotherapy is a promising strategy to extend OS in patients with ATC. Further studies in patients with non–BRAF-mutated ATC, using better targeted agents, are needed.

## Notes

Supplement 1. Trial Protocol

Supplement 2. eMethods 1. Entry criteria eMethods 2. Doses of study drugs eMethods 3. Patient instructions for alternative drug administration of vemurafenib eMethods 4. Futility analysis eFigure 1. Cohort 1 run-in and dosing eFigure 2. Treatment assignment eFigure 3. Oncoprint eFigure 4. Swimmer Plot eTable. Adverse Events amongst all cohorts

Supplement 3. The members of The Rare Tumor Initiative Team

Supplement 4. Data Sharing Statement
