# Supplementary material for: Anti–Programmed Death Ligand 1 Plus Targeted Therapy in Anaplastic Thyroid Carcinoma: A Nonrandomized Clinical Trial
Source: JAMA Oncol. 2024 Oct 24;10(12):1672–80. doi: 10.1001/jamaoncol.2024.4729 (PMC11581602; doi:10.1001/jamaoncol.2024.4729)
Supplement: Supplement 1. — Trial Protocol [file jamaoncol-e244729-s001.pdf]

MD Anderson IND Sponsor Cover Sheet	
**Protocol ID**	2016-0916
**Protocol Title**	ATEZOLIZUMAB COMBINATIONS WITH CHEMOTHERAPY FOR ANAPLASTIC AND POORLY DIFFERENTIATED THYROID CARCINOMAS
**Protocol Version**	23
**Version Date**	01/2024
**Protocol PI**	Maria Cabanillas
**Department**	Endocrine Neoplasia and Hormonal Disorders
**IND Sponsor**	MD Anderson Cancer Center
**IND #**	135299

PROTOCOL

TITLE: **ATEZOLIZUMAB COMBINATIONS WITH CHEMOTHERAPY FOR ANAPLASTIC AND POORLY DIFFERENTIATED THYROID CARCINOMAS**

STUDY NUMBER: (Local) Study # 2016-0916

VERSION NUMBER: 23

TEST PRODUCTS: atezolizumab, vemurafenib, cobimetinib, bevacizumab

INVESTIGATOR: Maria E. Cabanillas
1515 Holcombe Blvd, unit 1461
Houston, TX 77030
Telephone: 713-563-0769
Fax: 713-794-4065
E-mail: mcabani@mdanderson.org

SUB-INVESTIGATORS: Naifa Busaidy
Ramona Dadu
Renata Ferrarotto

SUPPORT PROVIDED BY: Genentech, Inc.

PROTOCOL DATE: January 2023

CONFIDENTIAL

This is a University of Texas MD Anderson Cancer Center document that contains confidential information. It is intended solely for the recipient clinical investigator(s) and must not be disclosed to any other party. This material may be used only for evaluating or conducting clinical investigations; any other proposed use requires written consent from The University of Texas MD Anderson Cancer Center.

TABLE OF CONTENTS

1. INTRODUCTION....15

1.1 Background: Anaplastic thyroid carcinoma 15

1.1.1. Current Management OF ATC 15

1.1.2 IMMUNE MICROENVIRONMENT IN THYROID CANCER 16

1.1.3 RAS-MAP Kinase Signaling Pathway....16

1.1.4 RAS 17

1.1.5 Mitogen-Activated Protein Kinase (MAPK)....17

1.1.6 Mitogen-Activated Protein Kinase (MEK) 17

1.1.7 Oncogenic BRAF Kinase Mutations in Thyroid cancer 18

1.1.8 VEGFR in Anaplastic Thyroid cancer 19

1.2 Background on atezolizumab 19

1.2.2 Clinical Experience with atezolizumab 19

1.2.2.1 Ongoing Clinical Studies....19

1.2.2.2 Clinical Safety....19

1.2.2.3 Clinical Activity....21

1.2.2.4 Clinical Pharmacokinetics and Immunogenicity 21

1.3 Clinical Experience with Vemurafenib AND COBIMETINIB 23

1.3.1 Summary of Clinical Studies and Efficacy with Vemurafenib in Melanoma 23

1.3.1.1 Phase I Dose-Finding Study (PLX 06-02)....23

1.3.1.2 Phase I Extension Cohorts (Study PLX 06-02)....24

1.3.1.3 Phase II Single-Arm Study (NP22657 [BRIM-2]) 24

1.3.1.4 Phase III, Randomized, Controlled Study (NO25026 [BRIM-3])....25

1.3.1.5 Pilot Single-Arm Brain Metastasis Study (MO25653) 26

1.3.1.6 Phase II Single-Arm Brain Metastasis Study (MO25743) 26

1.3.1.7 Phase II Single-Arm Study in Patients with Activating Exon 15 BRAF-Positive Mutation Tumors Other than V600E (ML27763) 27

1.3.1.8 Expanded Access and Post-Approval Safety Studies 27

1.3.2 Summary of Clinical Studies and Efficacy with Vemurafenib in Thyroid Carcinoma 28
1.3.2.1 PLX4032 Phase 1 28
1.3.2.2 Vemurafenib phase 2 in BRAF mutated PTC 28
1.3.2.3 Vemurafenib in non-melanoma BRAF mutated cancers (including ATC) 28
1.3.3 Summary of Clinical Studies with Cobimetinib in Melanoma 29
1.3.3.1 Phase I Cobimetinib Single-Agent Study (MEK4592) 29
1.4 Combined BRAF and MEK Inhibition 29
1.4.1.1 Phase Ib Open-Label Cobimetinib and Vemurafenib Study (NO25395; BRIM-7) 30
1.4.1.2 Randomized Phase III Study of Vemurafenib and Cobimetinib (GO28141; CoBRIM) 31
1.4.2 Clinical Efficacy of Cobimetinib and Vemurafenib in Melanoma 31
1.4.2.1 Study NO25395 (BRIM-7) 31
1.4.2.2 Study GO28141 (CoBRIM) 32
1.4.3 Safety Profile: Combined Vemurafenib and Cobimetinib Safety Summary 32
1.4.3.1 Study NO25395 (BRIM-7) 32
1.4.3.2 Study GO28141 (CoBRIM) 35
1.5 SAFETY PROFILE: VEMURAFENIB MONOTHERAPY 39
1.5.1 Safety in Healthy Subjects 39
1.5.2 Safety in Patients 40
1.5.2.1 Overview of Adverse Events 40
1.5.2.1.1 Study NO25026 40
1.5.2.1.2 Study NP22657 41
1.5.2.1.3 Study NP25163 41
1.5.2.1.4 Study MO25515 43
1.5.2.2 Grade 3 and Grade 4 Adverse Events 43
1.5.2.2.1 Study NO25026 43
1.5.2.2.2 Study NP22657 44

1.5.2.2.3 Study NP25163 45
1.5.2.2.4 Study MO25515 46
1.5.3 Deaths 46
1.5.3.1 Study NO25026 46
1.5.3.2 Study NP22657 47
1.5.3.3 Study NP25163 47
1.5.3.4 Study MO25515 48
1.5.4 Serious Adverse Events 48
1.5.4.1 Study NO25026 48
1.5.4.2 Study NP22657 48
1.5.4.3 Study NP25163 49
1.5.4.4 Study MO25515 49
1.5.5 Discontinuations due to Adverse Events 50
1.5.6 Adverse Events of Special Interest 50
1.5.6.1 Cutaneous Squamous Cell Carcinoma (cuSCC) 51
1.5.6.2 Other Neoplasms 51
1.5.6.2.1 Non-Cutaneous Squamous Cell Carcinoma 51
1.5.6.2.2 Adenomatous Colonic Polyps 52
1.5.6.3 52
1.5.6.4 Liver Injury 52
1.5.6.5 QT Prolongation 52
1.5.7 Safety on the Basis of BRAF V600 Mutation Status 53
1.5.7.1 Study NO25026 53
1.5.7.2 Study ML27763 53
1.5.8 Safety in Patients with Brain Metastases 54
1.5.8.1 Study MO25653 54
1.5.8.2 Study MO25743 55
1.5.9 Safety in Post-approval Studies 56
1.5.9.1 Global Safety Study MO25515 56
1.5.9.2 Expanded Access Study ML25597 56
1.5.10 Safety in Special Populations 57
1.5.10.1 Study YO28390 57
1.5.10.2 Study JO28178 58

1.5.10.3 Study NO25390 59
1.5.11 Safety in the Post Marketing Setting 60
1.5.11.1 Drug Reaction with Eosinophilia and Systemic Symptoms 60
1.5.11.2 Progression of Existing Malignancy 60
1.5.11.3 Neutropenia 61
1.5.11.4 Panniculitis 61
1.5.11.5 Pancreatitis 61
1.5.11.6 Risk of Radiation Recall and Radiation Sensitization 62
1.5.11.7 Acute Kidney Injury (AKI) 64
1.5.11.8 Dupuytren’s Contracture and Plantar Fibromatosis 64
1.5.11.9 Safety in Patients with Papillary Thyroid Cancer 64
1.5.11.10 Safety in Patients with Metastatic CRC 65
1.6 Safety profile: Cobimetinib Monotherapy 66
1.7 Bevacizumab Clinical Experience 67
1.8 Study Rationale 76
2. OBJECTIVES 77
2.1 Primary 77
2.2 Secondary 77
2.3 EXPLORATORY 77
3. STUDY DESIGN 77
3.1 Description of the Study 77
3.2 End of Study 81
3.3 Rationale for Study Design 81
3.4 Outcome Measures 81
3.4.1 Primary Efficacy Outcome Measure 81
3.4.2 Secondary Efficacy Outcome Measures 81
3.4.3 Safety Outcome Measures 81
4. MATERIALS AND METHODS 82
4.1 Study Population 82
4.1.1 Inclusion Criteria 82

4.1.2 Exclusion Criteria 83
4.2 Study Treatment 85
4.2.2 Study Drug: atezolizumab 86
4.2.1.1 Formulation 86
4.2.1.2 Dosage, Administration, and Storage 86
4.2.2 Other Study Drug(s): Vemurafenib, cobimetinib, bevacizumab....88
4.2.3 Vemurafenib Dosage, Storage, and Administration 88
4.2.4 Cobimetinib Dosage, Storage, and Administration....88
4.2.5 Bevacizumab Dosage, Storage, and Administration 89
4.2.6 Protocol-Specified Chemotherapy (nab-paclitaxel or paclitaxel) 90
4.2.7 Summary of Pharmacy Data 90
4.2.8 Concomitant Therapy and Additional Restrictions 90
4.2.8.1 Permitted Therapy....91
4.2.8.2 Prohibited Therapy 92
4.2.8.3 Prohibited Foods and Drinks 93
4.2.8.4 Cautionary Medications 93
4.3 Systemic corticosteroids are recommended, at the discretion of the investigator, for the treatment of specific adverse events when associated with atezolizumab therapy. General Plan to Manage Safety Concerns 93
4.3.1 Management of Specific Safety Concerns with atezolizumab....94
4.3.2 Guidelines for Dosage Modification and Treatment Interruption or Discontinuation of atezolizumab 95
4.4 Study Treatment Modifications 96
4.4.1 Dose Modifications for Vemurafenib 96
4.4.1.1 For New Primary Cutaneous Malignancies....96
4.4.1.2 For Other Adverse Reactions 96
4.4.2 Dose Modification Guidelines for Cobimetinib....97
4.4.3 Dose Modification Guidelines for bevacizumab....101
4.4.3.1 Proteinuria....105
Hemorrhage 107

4.4.4 Dose Modification Guidelines for nab-paclitaxel or paclitaxel (cohort 4) 109
4.5 Study Treatment Discontinuation 109
4.6 Study and Site Discontinuation 110
4.7 Clinical and Laboratory Evaluations 110
4.7.1 Pretreatment Evaluations 110
4.7.2 Study Assessments 110
4.7.2.1 Medical History 110
4.7.2.2 Vital Signs 110
4.7.2.3 Physical Examination 111
4.7.2.4 Tumor and Response Evaluation 111
4.7.2.5 Laboratory Assessments 111
4.7.2.6 Outside Physician Participation During Treatment 113
4.7.3 Specialized Evaluations Required for Safety Monitoring 113
4.7.3.1 LVEF Assessment 113
4.7.3.2 Prolongation of QT Interval 113
4.7.3.3 Secondary Malignancies 114
4.7.3.4 Dermatologic Exam 114
4.7.3.5 Ophthalmologic Exam 114
4.7.4 Treatment Discontinuation Visit 114
4.7.5 Follow-Up Assessments 115
4.7.6 Post-Treatment Evaluations 115
5. STATISTICAL CONSIDERATIONS 115
5.1 Determination of Sample Size 115
5.2 Planned Efficacy Evaluations 115
5.3 Primary Efficacy Variables 116
5.4 Secondary Efficacy Variables 116
5.5 Method of Analysis 116
5.6 Futility Monitoring Plan 116
6. ASSESSMENT OF SAFETY 118
6.1 Risks Associated with atezolizumab 118
6.2 Safety Parameters and Definitions 119

6.2.1 Adverse Events 119
6.2.2 Serious Adverse Event (SAE) Reporting 119
6.3 Methods and Timing for Assessing and Recording Safety Variables 120
6.3.1 The investigator is responsible for ensuring that all AEs and SAEs that are observed or reported during the study are collected and reported to Genentech, Inc., in accordance with CFR 312.32 (Investigational New Drug [IND] Safety Reports). Adverse Event Reporting Period 120
6.3.2 Assessment of Adverse Events 121
6.4 Procedures for Eliciting, Recording, and Reporting Adverse Events 122
6.4.1 Eliciting Adverse Events 122
6.4.2 Specific Instructions for Recording Adverse Events 122
6.4.2.1 Diagnosis versus Signs and Symptoms 122
6.4.2.2 Deaths 122
6.4.2.3 Pre-existing Medical Conditions 122
6.4.2.4 Hospitalizations for Medical or Surgical Procedures 122
6.4.2.5 Pregnancies in Female Patients 123
6.4.2.6 Pregnancies in Female Partners of Male Patients 123
6.4.2.7 Abortions 124
6.4.2.8 Congenital Anomalies/Birth Defects 124
6.4.2.9 Post-Study Adverse Events 124
6.4.2.10 Safety Reconciliation 124
6.4.2.11 Adverse Events of Special Interest 124
6.4.2.12 Adverse Event Reporting 127
6.4.3 Additional Reporting Requirements for IND 128
6.4.4 IND Annual Reports 129
6.5 Study Close-Out 129
6.6 IND Office Review 130
7. ETHICAL CONSIDERATIONS 130
7.1 Compliance with Laws and Regulations 130
8. STUDY MEDICAL MONITORING REQUIREMENTS 130
8.1 Study Medication Accountability 130

8.2 Data Collection 131

9. DATA SECURITY AND CONFIDENTIALITY...131

10. REFERENCES ……………………………………………………………………132

LIST OF TABLES

Table 1 Treatment-Emergent Adverse Events That Occurred in 10% or More of Patients in Study NO25395 (BRIM-7) 32
Table 2 Summary of Patients Experiencing Protocol-Defined AESIs Irrespective of Relationship to Study Drug in Study NO25395 35
Table 3 Most Common Adverse Events (Occurring in □ 20% of Patients) in Study GO28141..36
Table 4 Grade □ 3 Adverse Events Occurring in at Least 2% of Patients in Either Arm (Safety-Evaluable Population) 37
Table 5 Summary of Adverse Events of Special Interest (Safety-Evaluable Population)..39
Table 6 Study NO25026: Overview of AEs (Safety Population) 41
Table 7 Study NP22657: Overview of AEs and Deaths (Safety Population)...41
Table 8 NP25163: Overview of AEs and Deaths (Safety Population) 42
Table 9 Vemurafenib Dose Modification Schedules for QTc Prolongation...97
Table 10 Vemurafenib General Dose Modifications 97
Table 11 Cobimetinib General Dose Modifications 98
Table 12 Cobimetinib or Cobimetinib/Vemurafenib Dose Modification Guidelines for Left Ventricular Dysfunction..................................99
Table 13 Cobimetinib Dose Modification Guidelines for Specified Adverse Drug Reactions 100
Table 14 Bevacizumab Dose Modification Guidelines for Specified Adverse Drug Reactions 102
Table 15 Bevacizumab Treatment Management for Proteinuria 106
Table 16 Dose Modification Guidelines for Nab-paclitaxel or paclitaxel (Cohort 4).. 109

LIST OF FIGURES

Figure 1: Study Design 80
Figure 2: Triplet dosing schedule with run-in for vemurafenib + cobimetinib + atezolizumab...86

TABLE OF CONTENTS (CONT.)

LIST OF APPENDICES

Appendix 1 Study Flowchart 134
Appendix 2 Calculation of Creatinine Clearance Using the Cockcroft-Gault Formula 140
Appendix 3 Current National Cancer Institute Common Terminology Criteria for Adverse Events (NCI CTCAE) 141
Appendix 4 Response Evaluation Criteria in Solid Tumors (RECIST) 142
Appendix 5 Immune-Related Response Criteria 152
Appendix 6 Eastern Cooperative Oncology Group (ECOG) Performance Status Scale 154
Appendix 7 Anaphylaxis Precautions 155
Appendix 8 Safety Reporting Fax Cover Sheet 156
Appendix 9 Cohort 1 (Vemurafenib/Cobimetinib/atezolizumab) AE Management 157
Appendix 10 Cohort 2 (Cobimetinib+atezolizumab) AE Management 180
Appendix 11 Guidelines for Managing atezolizumab-related AEs 200
Appendix 12 Translational studies – atezolizumab combinations with chemotherapy for anaplastic and poorly differentiated thyroid carcinomas 219
Appendix 13 Patient Instructions for Oral Vemurafenib 225
Appendix 14 COVID-19 Pandemic: Standard Operating Procedures and Guidelines 227

List of Abbreviations and Definition of Terms

Abbreviation	Definition
AE	adverse event
AESI	adverse event of special interest
anti-HBc	antibody to hepatitis B core antigen
ATA	anti-therapeutic antibody
AUC	area under the concentration-time curve
BSA	body surface area
CFR	Code of Federal Regulations
C_{max}	maximum serum concentration
C_{min}	minimum serum concentration
CNS	central nervous system
CL	clearance
CR	complete response
CRF	Case Report Form
CRO	contract research organization
CT	computed tomography
DL_{CO}	diffusion capacity of the lung for carbon monoxide
DLT	dose-limiting toxicity
EBV	Epstein-Barr virus
EBNA	Epstein-Barr nuclear antigen
EC	Ethics Committee
EC_{50}	50% effective concentrations
ECOG	Eastern Cooperative Oncology Group
FDA	U.S. Food and Drug Administration
FFPE	formalin-fixed paraffin-embedded
FOLFOX	leucovorin, 5-fluorouracil, oxaliplatin
GCP	Good Clinical Practice
HAV	hepatitis A virus
HBsAg	hepatitis B surface antigen
HBV	hepatitis B virus
HCV	hepatitis C virus
HDV	hepatitis D virus
IC_{50}	50% inhibitory concentration
ICF	Informed Consent Form
ICH	International Conference on Harmonisation
IFN	interferon
Ig	immunoglobulin

List of Abbreviations and Definition of Terms

Abbreviation	Definition
IHC	immunohistochemistry
IL	interleukin
IMP	investigational medicinal product
IND	Investigational New Drug (application)
irAE	immune-related adverse event
IRB	Institutional Review Board
IRF	independent review facility
IRR	infusion-related reaction
irRC	immune-related response criteria
IV	intravenous
LFT	liver function test
LPLV	last patient, last visit
MRI	magnetic resonance imaging
MTD	maximum tolerated dose
NCI CTCAE	National Cancer Institute Common Terminology Criteria for Adverse Events
NOAEL	no observed adverse effect level
NSCLC	non-small cell lung cancer
ORR	objective response rate
OS	overall survival
PBMC	peripheral blood mononuclear cell
PCR	polymerase chain reaction
PD	progressive disease
PD-1	programmed death-1
PD-L1	programmed death-ligand 1
PES	polyethersulfone
PET	positron emission tomography
PFS	progression-free survival
PI	Package Insert
PK	pharmacokinetic
PR	partial response
PSA	prostate-specific antigen
PUVA	psoralen plus ultraviolet A radiation
PVC	polyvinylchloride
qRT-PCR	quantitative reverse-transcription polymerase chain reaction
RCC	renal cell carcinoma

List of Abbreviations and Definition of Terms

Abbreviation	Definition
RECIST	Response Evaluation Criteria in Solid Tumors
SAE	serious adverse event
SD	stable disease
TNF	tumor necrosis factor
TSH	thyroid-stimulating hormone
UBC	urothelial bladder cancer
ULN	upper limit of normal
V_{ss}	volume at steady state

1. INTRODUCTION

1.1 BACKGROUND: ANAPLASTIC THYROID CARCINOMA

Anaplastic thyroid carcinoma (ATC) is the most aggressive type of thyroid malignancy with very poor prognosis due to its aggressive behavior and resistance to standard treatment. ATC patients have a median overall survival of 5 months and a 20% 1-year survival rate (Smallridge, Ain et al. 2012). Poorly differentiated thyroid cancer (PDTC) is intermediate on the spectrum between well-differentiated and ATC and may represent a transition form as a consequence of acquiring new mutations (e.g. TP53). PDTC patients experience less aggressive clinical course than ATC but still have very poor outcomes. Both groups are at high risk of distant metastatic disease. PDTC and ATC are in most cases derived from a more differentiated type of thyroid cancer (DTC), thus the mutation profile is very similar. Similar to DTC, the RAS-MAP-kinase pathway plays a fundamental role in ATC and PDTC. In ATC, the most common mutations are p53 (41%), RAS (27%), and BRAF (25%) (Cabanillas, Zafereo et al. 2016). At MD Anderson, BRAF mutations are more prevalent (48%), followed by TP53 (35%), PIK3CA (28%) and RAS (21%). The prevalence of BRAF mutations is similar to another tertiary cancer center's experience (Landa, Ibrahimpasic et al. 2016).

1.1.1. CURRENT MANAGEMENT OF ATC

First-line therapy: More than 80% of ATC patients present with extensively invasive primary tumors. Surgical resections of these tumors provides little benefit to patients unless the tumor is small and can be removed with clean margins. Patients who have complete resections have the best prognosis, yet only a small fraction of patients will be resectable. In patients with unresectable disease (the majority of patients), first -line treatment consists of palliative external beam radiation (60-66 Gy in 30 fractions, with or without concurrent chemotherapy for radiosensitizing effects) to prevent a life threatening airway compromise (Smallridge, Ain et al. 2012). Despite best initial therapeutic efforts patients develop disease progression or distant metastatic disease in a median of 6 months.

In patients who present with widely metastatic disease, the treatment is the same as patients presenting without distant metastases (neck radiation with radiosensitizing chemotherapy). In these cases, the radiation is purely palliative. Patients undergoing palliative radiation often die of distant disease before they can start any effective therapy for their systemic disease. Cytotoxic chemotherapy is an alternative to palliative radiation however, the response are short-lived. Paclitaxel induction in ATC was first described by Ain el al (Ain, Egorin et al. 2000). Using high dose paclitaxel every 3 weeks, they were able to achieve a 53% response rate but these were transient. Subsequently, Higashiyama et al (Higashiyama, Ito et al. 2010) published their trial results using weekly paclitaxel in patients with stage IVB and IV C ATC. Patients were treated with induction paclitaxel for 2-3 courses and then surgically resected when feasible, followed by adjuvant paclitaxel. This approach resulted in a significantly longer overall survival in IVB patients who received induction chemotherapy versus those who did not. The response rate (CR+PR) was 33% in IVB patients and 25% in IVC patients. The mean time to PR for the entire cohort was 6 weeks.

We believe that the current approach to ATC must be completely revised in the age of such promising, emerging targeted and immunotherapies. However, due to the highly aggressive nature of this disease, cytotoxic chemotherapy needs to be used as a bridge

while molecular testing is being processed. Responses to targeted therapies (i.e., vemurafenib (Rosove, Peddi et al. 2013, Hyman, Puzanov et al. 2015), (Prager, Koperek et al. 2016), dabrafenib (Falchook, Millward et al. 2015), dabrafenib+trametinib (Cabanillas, Busaidy et al. 2016, Iyer, Dadu et al. 2016), and the antiangiogenic drug, Lenvatinib (Iyer, Dadu et al. 2016, Takahashi, Kiyota et al. 2016) are more durable (PFS=7.4 months with lenvatinib) and quite promising. Clinical trials with dabrafenib + trametinib (NCT02034110) and with lenvatinib (NCT02657369) in ATC are currently ongoing. ATC and PDTC have florid immune infiltrates that highly express PD-L1, suggesting that ATC is a good candidate for immunotherapy. Due to the rapid growth of anaplastic tumors, single agent immunotherapy is not a feasible approach, as these patients need responses to occur within weeks.

1.1.2 IMMUNE MICROENVIRONMENT IN THYROID CANCER

We have generated pre-clinical data to support the use of immunotherapy in ATC (Dadu, Vilalobos et al. 2016). Immunoprofiling of a large ATC cohort shows that PD-L1+ tumor cells and TILs are present in high frequency. Twenty-one ATC tumors (15 primary, 5 nodal, 1 distant metastasis) that were resected prior to treatment were analyzed. Fifty-two percent were BRAF V600E mutated. Median percent PD-L1 positive tumor cells=40% (0.4-99.9) and PD-L1 H-score=41 (0.4-233). Median cell density: PD1=63 (7-102); CD4=702 (47-4028), CD8=430 (54-3196), CD45Ro=538 (113-2682), FoxP3=217 (43-873), GrB=156 (23-1265), CD68=196 (23-1568). Higher PDL1 expression was observed in BRAF mutant tumors.

In an immunocompetent mouse model of anaplastic thyroid cancer with tumors harboring the BRAFV600E mutation, Brauner et al (Brauner, Gunda et al. 2016) investigated the antitumor effect of anti-PD-L1 antibody therapy, selective BRF inhibition (PLX4720), and both treatments in combination after 8 days of treatment. The combination dramatically reduced the tumor size in the animals compared to either drug alone. Immunohistochemistry showed CD8+ T cell infiltration and a favorable CD8+:Treg ratio with the combination.

1.1.3 RAS-MAP KINASE SIGNALING PATHWAY

The RAS-MAP-kinase signaling pathway is a highly conserved enzymatic pathway that transduces extracellular signals into long-term changes in intracellular biochemistry and gene expression (Alberts et al. 2008). Since the pathway, in its different forms, is critically involved in cell-cycle control and development, mutations in genes that affect the system—in particular, in genes that encode the RAS-MAP-kinase signaling proteins themselves, their intracellular regulators, or their cognate membrane receptors —are among the most common mutations found in cancer cells. Consequently, the RAS-MAP-kinase pathway has been the subject of intense pharmacologic analysis, as any agent that specifically targets this pathway could have important clinical utility in a variety of cancers.

The core of the RAS-MAP-kinase signal transduction system consists of a membrane-associated RAS protein and 3 serine/threonine protein kinases.

1.1.4 RAS

The RAS proteins belong to the large RAS superfamily of monomeric GTPases (Alberts et al. 2008). Like other GTP-binding proteins, RAS functions as a switch, cycling between two distinct conformational states: active when GTP is bound and inactive when GDP is bound. Two classes of signaling proteins regulate RAS activity by influencing its transition between active and inactive states. Guanine nucleot ide exchange factors (GEFs) promote the exchange of bound nucleotide by stimulating the dissociation of GDP and the subsequent uptake of GTP from the cytosol, thereby activating RAS. GTPase-activating proteins (GAPs) increase the rate of hydrolysis of bound GTP by RAS, thereby inactivating RAS.

Three RAS proteins (HRAS, KRAS, and NRAS) are implicated in human cancer. Mutations in genes encoding these three proteins can produce hyperactive variants that are resistant to GAP-mediated GTPase stimulation. These mutational alterations lock the proteins permanently into their GTP-bound active states, which may ultimately promote dysregulated growth and cancer. Activated RAS mutations are particularly common in cancer. Mutations in KRAS, for instance, have been identified in 58% of pancreatic, 34% of large intestine, 29% of biliary tract, 20% of small intestine, 17% of lung, 15% of endometrial, and 14% of ovarian cancer samples sequenced to date (Forbes et al. 2008; Forbes et al. 2011).

1.1.5 Mitogen-Activated Protein Kinase (MAPK)

Once activated (either by binding GTP in normal cells or as a result of mutational alterations in cancer cells), RAS activates a downstream serine/threonine phosphorylation cascade composed of 3 mitogen-activated protein kinases (MAPK; Alberts et al. 2008)(Alberts, Johnson et al. 2008)(Alberts, Johnson et al. 2008)(Alberts, Johnson et al. 2008). The pathway activated by RAS begins with a MAPK-kinase-kinase called RAF, which activates the MAPK-kinase (MEK). MEK, in turn, activates a MAP-kinase called ERK.

The MAP-kinase ERK then relays the signal further downstream by phosphorylating various proteins in the cell, including gene regulatory proteins and other protein kinases. Among the genes activated by this pathway are those required for cell proliferation, such as the genes encoding G1 cyclins. Consequently, constitutive activation of the phosphorylation cascade can result in inappropriate mitotic drive, resulting in the unregulated growth that characterizes cancer cells.

1.1.6 Mitogen-Activated Protein Kinase (MEK)

The ERK/MAPK signaling cascade transduces multiple proliferation and differentiation signals within the cell via activation of the RAS GTPase and subsequent sequential activation of RAF, MEK, and ERK kinases. Dysregulation of this pathway contributes to many hallmarks of cancer cells, including uncontrolled proliferation, invasion, metastasis, angiogenesis, and evasion of apoptosis (Downward 2003; Roberts and Der 2007).

Inhibition of MEK1/2 (MAPK/ERK kinase) is a promising strategy in the development of oncology therapeutics to control the growth of tumors that are dependent on aberrant ERK/MAPK pathway signaling (Wellbrock et al. 2004; Solit et al. 2006; Roberts and Der 2007). The ERK/MAPK pathway is upregulated in 30% of all tumors, and oncogenic activating mutations in KRAS and BRAF have been identified in 22% and 18% of all cancers, respectively (Davies et al. 2002; Allen et al. 2003; Malumbres and Barbacid 2003; Bamford et al. 2004). Activating BRAF mutations are prevalent in malignant melanomas (66%) and are also found in colon cancer (15%) and thyroid papillary carcinoma (27%). Eighty percent of the constitutive activating BRAF mutations are a single amino-acid substitution (V600E) in the activation loop of the kinase (Davies et al. 2002; Downward 2003; Bamford et al. 2004). Cancer cells transformed by $BRAF^{V600E}$ are highly sensitive to MEK1/2 inhibition (Solit et al. 2006); therefore, MEK1/2 inhibitors may have particular clinical utility in melanoma and other tumors harboring this BRAF mutation.

1.1.7 ONCOGENIC BRAF KINASE MUTATIONS IN THYROID CANCER

The MAP-kinase-kinase-kinase RAF acts at the intersection between the initial part of the signaling pathway, comprised of a receptor tyrosine kinase and RAS, and the subsequent phosphorylation cascade that transduces the extracellular signal to the nucleus. To date, mutations in three different RAF proteins (ARAF, BRAF, and CRAF) have been implicated in human cancer (Forbes et al. 2008; Forbes et al. 2011). Among these, mutations in BRAF are the most common, particularly in melanoma, where BRAF mutations have been identified in 67% of primary melanoma tumors and 80% of melanoma short-term cultures (Davies et al. 2002)(Davies, Bignell et al. 2002)(Davies, Bignell et al. 2002)(Davies, Bignell et al. 2002). BRAF mutations have also been identified in 38% of thyroid, 12% of large intestine, 12% of genital tract, 11% of ovarian, 11% of eye, and 10% of biliary tract cancer cell line isolates sequenced to date and described in the Catalog of Somatic Mutations in Cancer (COSMIC) database (Forbes et al. 2008; Forbes et al. 2011).

Activating mutations in BRAF have been identified at high frequency in papillary thyroid carcinoma primary tumors, occurring in up to 40% of sequenced melanoma samples (Davies et al. 2002), and in approximately 25% of anaplastic thyroid carcinomas. BRAF mutated ATC represents a dedifferentiated PTC. These mutations typically fall within the kinase domain of the protein, with a single substitution (V600E) accounting for 90% of the sequenced mutants.

Therapeutic inhibition of the activating BRAF V600E mutation with vemurafenib, a selective BRAF kinase inhibitor, has demonstrated significant anticancer activity in melanoma, as well as papillary thyroid and anaplastic thyroid carcinoma patients, as described in Section 1.3.

1.1.8 VEGFR IN ANAPLASTIC THYROID CANCER

Angiogenesis is an important target in solid tumors. In thyroid cancers, particularly in ATC, alterations of VEGF and VEGFR are observed. Targeting the VEGF pathway may result in inhibition of tumor growth. Two antiangiogenic drugs, sorfenib and Lenvatinib, are approved for differentiated thyroid cancer. The potent anti-angiogenic drug, lenvatinib, has shown efficacy in ATC (Takahashi, Kiyota et al. 2016), and is approved for ATC in Japan. Bevacizumab has not been tested in thyroid cancer or thyroid cancer cell lines. Although there are publications showing work in anaplastic thyroid cancer cell lines and xenografts, these cell lines were later shown to be contaminated with other cancer cell lines (Schweppe, Klopper et al. 2008).

1.2 BACKGROUND ON ATEZOLIZUMAB

Atezolizumab is a humanized immunoglobulin (Ig) G1 monoclonal antibody that targets PD-L1 and inhibits the interaction between PD-L1 and its receptors, PD-1 and B7-1 (also known as CD80), both of which function as inhibitory receptors express ed on T cells. Therapeutic blockade of PD-L1 binding by atezolizumab has been shown to enhance the magnitude and quality of tumor-specific T-cell responses, resulting in improved anti-tumor activity (Fehrenbacher et al. 2016; Rosenberg et al. 2016). atezolizumab has minimal binding to Fc receptors, thus eliminating detectable Fc-effector function and associated antibody-mediated clearance of activated effector T cells.

Atezolizumab shows anti-tumor activity in both nonclinical models and cancer patients and is being investigated as a potential therapy in a wide variety of malignancies. Atezolizumab is being studied as a single agent in the advanced cancer and adjuvant therapy settings, as well as in combination with chemotherapy, targeted therapy, and cancer immunotherapy.

Atezolizumab is approved in the United States for both the treatment of locally advanced or metastatic urothelial carcinoma and metastatic non-small cell lung cancer (NSCLC) in patients with disease progression during or after platinum-based chemotherapy.

Refer to the atezolizumab Investigator's Brochure for details on nonclinical and clinical studies.

1.2.2 Clinical Experience with atezolizumab

1.2.2.1 Ongoing Clinical Studies

As of 10 May 2016, 42 studies are ongoing with atezolizumab as a single agent or in combination with other therapies. Details of all ongoing studies can be found in the atezolizumab Investigator’s Brochure.

1.2.2.2 Clinical Safety

As of 10 May 2016, an estimated total of 6053 patients with solid tumor and hematologic malignancies have received atezolizumab in clinical trial participation as a single agent or in combination with cytotoxic chemotherapy and/or targeted therapy. No dose-limiting

toxicities (DLTs) no maximum tolerated dose (MTD) and no clear dose-related trends in the incidence of adverse events (AEs) have been determined Across all tumor types and studies, the most commonly reported AEs with single-agent atezolizumab include fatigue, nausea, decreased appetite, diarrhea, constipation, and cough. The adverse events observed with atezolizumab in combination with chemotherapy and/or targeted therapies are consistent with the known risks of each study treatment.

Adverse Events

Study PCD4989g is an ongoing Phase Ia trial evaluating the safety and pharmacokinetics of single agent atezolizumab in patients with locally advanced or metastatic solid tumors or hematologic malignancies. As of the CCOD of 15 December 2015, safety information was available for 629 safety-evaluable patients from all lines of therapy. Among 629 treated patients, 98.4% reported an AE while on studyThe most frequently observed AEs (occurring in □ 10% of treated patients) included fatigue, nausea, decreased appetite, pyrexia, dyspnea, diarrhea, constipation, cough, headache, back pain, vomiting, anemia, arthralgia, rash, insomnia, asthenia, abdominal pain, peripheral edema, pruritus, urinary tract infection, and dizziness.

Grade 3-4 AEs on the basis of National Cancer Institute Common Terminology Criteria for Adverse Events, Version 4.0 (NCI CTCAE v4.0), were reported by 316 of 629 patients (50.2%). There were 86 patients (13.7%) who reported Grade 3-4 AEs that were assessed as related to study drug by the investigators. The most frequently reported related Grade 3-4 AEs included fatigue and asthenia (1.3%), increased AST and dyspnea (1.1% each); and hyponatremia (>= 0.8%).

Ten patients (1.6%) had Grade 5 events. The events were single occurrences. The 7 events assessed as unrelated to atezolizumab were acute respiratory failure, pneumonia, sepsis, head injury, overdose, acute myocardial infarction, and hepatic hematoma. The 3 events assessed by the investigator as related to atezolizumab were death (not otherwise specified), hepatic failure, and pulmonary hypertension.

Immune-Related Adverse Events

Atezolizumab has been generally well tolerated. Adverse events with potentially immune-related causes consistent with an immunotherapeutic agent, including rash, influenza-like illness endocrinopathies, hepatitis or transaminitis, pneumonitis colitis, and myasthenia gravis, have been observed (see atezolizumab Investigator's Brochure for detailed safety results). To date, these events have been manageable with treatment.

.

For further details, see the atezolizumab Investigator’s Brochure.

1.2.2.3 Clinical Activity

Efficacy data were most extensive for patients with metastatic urothelial carcinoma (mUC; approximately 520) and with non−small cell lung cancer (NSCLC; approximately 1170) who were administered atezolizumab as a single agent. At the most recent clinical cutoff of 14 March 2016 for the Phase II IMvigor 210 study, the objective response rate (ORR) for allcomers in Cohort 2 (comprising second-line and beyond [2L +] mUC patients) was 15.8% (95% CI: 11.9, 20.4), with complete responses (CRs) observed in 42.9% of responders. The ORR for allcomers in Cohort 1 (comprising first-line [1L] cisplatin-ineligible mUC patients) was 23.5% (95% CI: 16.2, 32.2), and CRs were observed in 28.6% of responders. Patients from both cohorts also reported clinically meaningful and durable responses (Cohort 2 = 71.4% of responses ongoing with minimum follow-up of 16 months; Cohort 1 = 75% of responses ongoing with minimum follow-up of 12 months), in addition to survival.

The results from the four studies evaluating atezolizumab as monotherapy in locally advanced or metastatic NSCLC (PCD4989g, FIR, POPLAR, and BIRCH) demonstrated that higher PD-L1 expression on TCs or ICs was associated with higher ORRs (see Section 5.3.3 for details) and that responses were durable. In the primary analysis of the Phase II POPLAR (cutoff of 8 May 2015), intent-to-treat (ITT) patients had clinically meaningful and statistically significant overall survival (OS) improvement compared with docetaxel (stratified hazard ratio [HR] of 0.73 [95% CI: 0.53, 0.99]; p-value = 0.04). Further, improvement in OS in the atezolizumab arm relative to docetaxel increased with increasing PD-L1 expression. Other available efficacy data suggested that treatment with atezolizumab as a single agent or in combination with other therapeutic agents resulted in anti-tumor activity across a range of other tumor types and hematologic malignancies (i.e., renal cell carcinoma [RCC], triple-negative breast cancer [TNBC], melanoma, colorectal cancer [CRC], and non-Hodgkin’s lymphoma [NHL]), as well as across lines of therapy

For further details, see the atezolizumab Investigator’s Brochure.

1.2.2.4 Clinical Pharmacokinetics and Immunogenicity

Atezolizumab pharmacokinetics and other data have been analyzed from the following atezolizumab monotherapy studies: PCD4989g, JO28944, IMvigor 210, BIRCH, POPLAR, and FIR. No final PK data are available from studies where atezolizumab has been dosed in combination with other anti-cancer agents.

The key PK findings from the above-listed atezolizumab monotherapy clinical studies are summarized below:

- The pharmacokinetics of atezolizumab monotherapy have been characterized in patients in Study PCD4989g at doses 0.01 mg/kg to 20 mg/kg q3w, including the fixed dose 1200 mg (equivalent to 15 mg/kg). Exposure to atezolizumab increased dose proportionally over the dose range of 1 mg/kg to 20 mg/kg.

While a subset of ATA-positive patients in Study PCD4989g receiving 0.3 to 3 mg/kg atezolizumab q3w experienced a reduction of atezolizumab Cmin to below the PK assay lower limit of quantification (LOQ), patients receiving 10 to 20 mg/ kg atezolizumab, including the fixed 1200 mg dose, maintained geometric mean Cmin that was in excess of both the LOQ and the target serum concentration of 6 μg/mL (Deng et al. 2016).

- A Phase I popPK analysis that included 472 patients from Studies PCD4989g and JO28944 described atezolizumab pharmacokinetics for the dose range 120 mg/kg with a linear two-compartment disposition model with first-order elimination. The popPK analysis indicated that central compartment volume of distribution (V1) was 3.28 L and the Vss was 6.91 L in the typical patient. Further, the CL of atezolizumab was 0.20 L/day and the t1/2 was 27 days. Steady state was obtained after 6 to 9 weeks (2 to 3 cycles) of repeated dosing. The systemic accumulation in AUC, Cmax, and Cmin was 1.91, 1.46, and 2.75-fold, respectively.

- Based on an analysis of exposure, safety, and efficacy data, the following factors had no clinically relevant effect: age (21-89 years), body weight, gender, positive ATA status, albumin levels, tumor burden, region or ethnicity, renal impairment, mild hepatic impairment, level of PD-L1 expression, or ECOG status.

- The effect of moderate or severe hepatic impairment (bilirubin ≥ULN and AST≥ULN or bilirubin ≥ 1.0 to 1.5 ≥ULN and any AST elevation) on the pharmacokinetics of atezolizumab is unknown.

- No formal PK drug-drug interaction studies have been conducted with atezolizumab. The drug interaction potential of atezolizumab is unknown.

Immunogenicity data are available for the following single-agent atezolizumab studies: PCD4989g, IMvigor 210, POPLAR, BIRCH, and FIR. ATAs to atezolizumab have been observed at all dosing levels. Treatment-emergent (treatment-induced plus treatment-enhanced) ATAs were detected at one or more post-dose time points. ATA positivity had no major effect on atezolizumab concentrations and pharmacokinetics although there was a trend for lower Cmin values in the ATA−positive subgroup. For doses ≥ 10 mg/kg, average Cmin remained well in excess of the target serum concentrat ion of 6 μg/mL in the ATA−positive patients. The presence of ATAs did not appear to have a clinically significant impact on pharmacokinetics, safety, or efficacy. Samples that could be evaluated for neutralizing antibodies in these studies were too few in number (approximately 6%) to draw any conclusions.

1.3 CLINICAL EXPERIENCE WITH VEMURAFENIB AND COBIMETINIB

Vemurafenib (also known as RO5185426, PLX4032, or RG7204) is a low molecular weight, orally available inhibitor of the activated form of the BRAF serine-threonine kinase enzyme, which is commonly found in melanoma and thyroid cancers. Vemurafenib selectively inhibits oncogenic BRAF kinase. The rationale for identifying such a compound was first provided in 2002, when the high prevalence of activat ing mutations in the BRAF gene was identified in a variety of cancers, including melanoma (Stevens et al. 2004). The high level of selectivity of vemurafenib has been demonstrated in biochemical, cell-based, and in vivo assays (see the Vemurafenib Investigator's Brochure for details).

1.3.1 Summary of Clinical Studies and Efficacy with Vemurafenib in Melanoma

The following are key clinical trials in the vemurafenib clinical development program for melanoma.

1.3.1.1 Phase I Dose-Finding Study (PLX 06-02)

The recommended Phase II dose of vemurafenib was established in a multicenter, Phase I, dose-escalation study with a total of 55 patients, 49 of whom had a diagnosis of melanoma (Flaherty el al 2010b).

Of the patients enrolled in the dose-escalation portion of Study PLX 06-02 who received doses of 240 mg BID or more, 16 presented with tumors that harbored the $BRAF^{V600}$ mutation. Among these 16 patients, a partial response (PR) or complete response (CR) was seen in the following patients:

- One patient receiving 240 mg BID
- Two of the 4 patients receiving 320 or 360 mg BID
- Four of the 6 patients receiving 720 mg BID
- Four of the 5 patients receiving 1120 mg BID

The ORR, including either confirmed or unconfirmed responses, was 69% (11 of 16 patients) with 10 PR and one CR. Responses were seen at all sites of metastatic disease including liver, small bowel, and bone. The DOR ranged from 2 to more than 18 months.

Five patients with metastatic melanoma without BRAF mutation received vemurafenib doses of at least 240 mg BID. None had evidence of tumor regression during the study. Four patients developed progressive disease (PD) within the first 2 months of treatment (Flaherty et al. 2010b).

1.3.1.2 Phase I Extension Cohorts (Study PLX 06-02)

Once the recommended Phase II dose of 960 mg twice daily (BID) orally (PO) had been identified, a cohort of 32 additional patients with metastatic melanoma and prospectively identified BRAF^{V600} mutations were enrolled in the extension phase of this study (Flaherty et al. 2010b). A different cohort of 21 patients with metastatic colorectal cancer and identified BRAF^{V600} mutations were treated in the extension phase with the established dose of 960 mg BID (Flaherty et al. 2010b). The primary objective of these extension cohorts was to determine clinical RR. Secondary objectives were safety and additional pharmacokinetic and pharmacodynamic evaluations.

All 32 patients enrolled in the extension cohort of Study PLX 06-02 had metastatic melanoma with BRAF^{V600E} mutation. All were treated with vemurafenib at the recommended Phase II dose of 960 mg BID PO. Thirteen patients (41%) required a dose reduction during therapy (i.e., to 720 mg BID in 10 patients, to 600 mg BID in one patient, and to 480 mg BID in 2 patients). Among the 32 evaluable patients in the melanoma extension cohort, the unconfirmed RR was 81.3%: 3 patients had a CR and 24 patients had a PR. The confirmed response rate (CR + PR) was 56.3%.

Responses were observed in visceral organs and bone metastases, as well as lungs and lymph nodes. Responses (i.e., 10 PR in 13 patients) were also observed in patients with increased concentrations of serum lactate dehydrogenase (LDH). Responses were observed in patients who had received no previous therapy (6 of 7 patients responded with vemurafenib in first-line treatment) and in patients who received one or more prior systemic therapies (9 of 9 patients in second-line, 4 of 4 patients in third-line, and 7 of 12 patients in more than third-line). The median OS was 16 months with a 2-year survival rate of 44%.

1.3.1.3 Phase II Single-Arm Study (NP22657 [BRIM-2])

Study NP22657 (BRIM-2) was an open-label, single-arm, multicenter Phase II study in previously treated patients with metastatic melanoma harboring the BRAF ^{V600} mutation. In this study, 132 patients were enrolled and treated with vemurafenib 960 mg BID PO. The tumor BRAF mutation status was assessed by the cobas® 4800 BRAF^{V600} Mutation Test. The primary objective of this study was to evaluate the efficacy of vemurafenib using best overall response rate (BORR) as assessed by an independent review committee (IRC; Response Evaluation Criteria in Solid Tumors [RECIST], Version 19.1). Secondary objectives included BORR assessed by the investigator, duration of response (DOR), PFS, OS, safety/toxicity, effect on QT interval, quality of life (QOL) using FACT-M (Version 4), validation of the cobas® 4800 BRAF^{V600} Mutation Test, and pharmacodynamic parameters.

In Study NP22657 (BRIM-2), 132 patients were enrolled between October 2009 and March 2010 (Sosman et al. 2010; Ribas et al. 2011). Of these, 122 (92.5%) harbored the BRAF^{V600E} mutation and 10 (7.5%) the BRAF^{V600K} mutation.

At the data cut-off of 1 July 2011, the median follow-up was 12.9 months (range 0.6 to 20.1). In total, 8 CR, 62 PR, 38 stable disease (SD), and 18 PD have been confirmed by an IRC, resulting in an IRC-assessed ORR of 53% (primary endpoint). Investigator - assessed ORR and RR, the latter includes unconfirmed responses, were 57% and 69%, respectively. Median DOR was 6.7 months (95% CI: 5.6−9.8 months; range 1.3−12.7 months). Median PFS was 6.8 months (95% CI, 5.6−8.1 months), with a 6-month PFS rate of 56% (95% CI: 47%-64%). The median OS was 15.9 months (95% CI: 11.6−18.3). The OS rate at 6 months was 77% (95% CI: 70−85 months) and 58% (95% CI: 49−67 months) at 12 months (Sosman et al. 2010).

1.3.1.4 Phase III, Randomized, Controlled Study (NO25026 [BRIM-3])

Study NO25026 (BRIM-3) was a randomized, open-label, multicenter, Phase III study examined patients with treatment-naïve metastatic melanoma confirmed by histopathology (unresectable stage IIIC or stage IV) and with a BRAF ^{V600} mutation by the cobas□ 4800 BRAF^{V600} Mutation Test (Chapman et al. 2011). Patients (N = 675) were randomly assigned to be treated with vemurafenib 960 mg BID PO or IV dacarbazine 1000 mg/m^2 on Day 1 every 3 weeks. Within this trial, OS and PFS were defined as co-primary endpoints (NO25026 protocol version C). Major secondary study objectives included comparisons of BORR, time to response, DOR, time to treatment failure, and tolerability/safety. Further assessments of the pharmacokinetic profile of vemurafenib, validation of the cobas□ 4800 BRAF^{V600} Mutation Test, evaluation of QOL, and additional pharmacodynamic analyses were planned. The final analysis was planned to occur after 196 deaths, and an interim analysis was planned after 50% of the projected deaths (n = 98). The final analysis of PFS was to occur at the interim analysis of OS.

In Study NO25026 (BRIM-3), a global, randomized Phase III study, 675 patients with previously untreated, metastatic melanoma harboring the BRAF^{V600E} mutation were randomly assigned to receive either vemurafenib or dacarbazine between January 2010 and December 2010 (Chapman et al. 2011). In the interim analysis for OS and the final analysis for PFS (see Section 1.3.2), vemurafenib was associated with a relative reduction of 63% in the risk of death and of 74% in the risk of either death or disease progression, as compared with dacarbazine (P □ 0.001 for both comparisons). The survival benefit in the vemurafenib group was observed in each prespecified subgroup, according to age, sex, Eastern Cooperative Oncology Group (ECOG) performance status, tumor stage, LDH level, and geographic region. After review of the interim analysis by an independent data and safety monitoring board, crossover from dacarbazine to vemurafenib was recommended.

In the vemurafenib group, most patients had a detectable decrease in tumor size and 106 of 219 patients (48%; 95% CI: 42%–55%) had a confirmed objective response, including 2 patients with a CR and 104 with a PR. Median time to response was 1.45 months. Ten patients in the vemurafenib group were later found to have BRAF^{V600K} mutations; of these, 4 had a PR (40%). In the dacarbazine group, a minority of patients had a detectable decrease in tumor size, and 12 of 220 patients (5%; 95% CI: 3% − 9%)

met the criteria for a confirmed response (all PRs). The median time to response was 2.7 months. The difference in confirmed RR between the two study groups (48% vs. 5%) was statistically significant (P □ 0.001).

In a recent post hoc analysis (data cut 1 February 2012), updated survival data censored at crossover showed a median OS of 13.6 months for the vemurafenib treatment arm and 9.7 months for the dacarbazine treatment arm (HR 0.70 [95% CI: 0.57–0.87] P□ 0.001). The median PFS was 6.9 months with vemurafenib treatment compared to 1.6 months with dacarbazine treatment (HR 0.38 [95% CI: 0.32–0.46] P□ 0.0010 (McArthur et al. 2014). This study also assessed efficacy by $BRAF^{V600}$ mutation type (V600E and V600K). Median OS with vemurafenib in the $BRAF^{V600E}$ group (n= 295) was 13.3 months compared with 10.0 months in the dacarbazine group (n = 303; HR 0.75 [95% CI: 0.60-0.93]). Median PFS was 6.9 months and 1.6 months respectively (HR 0.39 [95% CI: 0.33-0.47]). For the $BRAF^{V600K}$ mutation group median OS was 14.5 months with vemuraf enib (n = 33) and 7.6 months with dacarbazine (n = 24; HR 0.43 [95% CI: 0.21-0.90]). Median PFS was 5.9 months and 1.7 months, respectively (HR 0.30 [95% CI: 0.16-0.56)

1.3.1.5 Pilot Single-Arm Brain Metastasis Study (MO25653)

MO25653 was an open-label, pilot study in which 24 patients with metastatic BRAF-mutation positive melanoma with systematic brain metastases received 960 mg vemurafenib BID. The primary end-point was safety. Secondary end-points included BORR, PFS, and OS (Dummer et al. 2014).

In this open-label, pilot study, 24 patients with $BRAF^{V600}$-mutation positive melanoma with non-resectable symptomatic brain metastases received 960 mg BID vemurafenib. The median PFS was 3.9 months (95% CI: 3.0-5.5) and median survival was 5.3 months (95% CI: 3.9-6.6). These data indicate that vemurafenib therapy is feasible in patients with advanced melanoma with symptomatic brain metastases (Dummer et al. 2014).

1.3.1.6 Phase II Single-Arm Brain Metastasis Study (MO25743)

MO25743 is an open-label, single-arm, multicenter, Phase II study to evaluate the efficacy of 960 mg vemurafenib BID in patients with metastatic $BRAF^{V600}$ positive melanoma with symptomatic or non-symptomatic brain metastases (n = 146; Kefford et al. 2013). The study included 2 simultaneous cohorts: previously untreated patients for brain metastasis (n = 90) and previously treated patients for brain metastasis (n = 56). The primary endpoint was intracranial BORR assessed by an IRC using RECIST Version 19.1 for patients with previously untreated brain metastases. Major secondary endpoints included IRC- and investigator-assessed extracranial BORR, intracranial DOR, PFS, time to development of new brain metastases in responding patients, OS, and safety and tolerability in patients with melanoma metastatic to the brain.

In an interim efficacy and safety analyses of MO25743, between July 2011 and April 2013, 146 patients were enrolled where all received □ 1 dose of 960 mg BID

vemurafenib. Best OS rates in the brain as assessed by IRC were 18% (16 of 90 patients) for the untreated brain metastases cohort and 20% (11 of 56 patients) for the previously treated brain metastases cohort. Median PFS and OS were similar in both cohorts. Median investigator-assessed PFS was 3.71 months (95% CI: 3.58−3.78) in the untreated cohort and 4.04 months (95% CI: 3.68 -5.55) in the previously treated cohort. Median OS was 6.47 months (95% CI: 5.91-8.31) for the previously treated cohort. See the Investigator's Brochure for more details.

1.3.1.7 Phase II Single-Arm Study in Patients with Activating Exon 15 BRAF-Positive Mutation Tumors Other than V600E (ML27763)

ML27763 is an open-label, single-arm, multicenter (n = 10 sites), Phase II study to evaluate the efficacy of vemurafenib for in metastatic melanoma patients who se tumor has an activating exon 15 BRAF mutation other than V600E as detected by DNA sequencing performed by a centralized laboratory. As of 15 August 2014, 31 patients had been enrolled: 13 whose tumors are positive for BRAF ^{V600K} mutation and 18 patients whose tumors are positive for other BRAF mutations. All patients received vemurafenib 960 mg BID until disease progression, unmanageable toxicity, patient request for discontinuation, or study termination by Genentech. The primary endpoint is BORR as assessed by the investigator. Secondary endpoints include additional efficacy assessments (i.e., time to tumor response [TTR], DOR, PFS, OS, 6 - and 12-month survival) and evaluation of the safety profile of vemurafenib (Hallmeyer et al. 2014).

In an interim efficacy and safety analyses of ML27763 had a median follow-up for the overall population of 8.8 months for 31 patients at 10 US sites. As of the 15 August 2014 cut-off date, 5 (16%) patients were still receiving study treatment, and 26 (84%) discontinued treatment mainly due to disease progression (19/26, 73%). The investigator-assessed confirmed BORR in the intent-to-treat population was 23% (95% CI: 5-54) in V600K patients, 22% (95% CI: 6-48) in the non-V600E/K patients, and 23% (95% CI: 10-41) in the overall population. Unconfirmed BORR in the intent-to-treat population was 54% (95% CI: 25-81) in V600K patients, 33% (95% CI: 13, 59) in non-V600E/K patients, and 42% (95% CI: 25-61) in the overall patient population (Hallmeyer et al. 2014).

1.3.1.8 Expanded Access and Post-Approval Safety Studies

Two follow-up studies were conducted as expanded access or post-approval studies: ML25597 (N = 371) and MO25515 (N = 3219). Both studies were open-label, single-arm studies assessing 960 mg vemurafenib BID in patients with metastatic melanoma with documented BRAF V600E mutation. ML25597 was an expanded access program to provide patients with vemurafenib between the end of the Phase III study and commercial availability of vemurafenib (Flaherty et al. 2014). MO25515 was a post-approval, global, open-label, multicenter safety study of vemurafenib in patients with metastatic melanoma who had failed at least one previous systemic treatment and were without satisfactory treatment options. Both studies assessed ORR and safety.

In Study ML25597, at baseline, most patients (75%) had Stage M1c disease, and 19% had an ECOG PS of 2 or 3; 72% of patients had received prior systemic therapy for metastatic melanoma, 27% received prior ipilimumab, and 29% radiotherapy for prior brain metastases. Among 241 efficacy-evaluable patients, the ORR was 54% (median time to response, 1.9 months). The ORR in non-central nervous system sites in patients with previously treated brain metastases (n = 68) was 53%. The ORR in prior ipilimumab-treated patients (n = 68) was 52%. For patients with PS of 0 or 1 (n = 210) and 2 or 3 (n = 31), the ORRs were 55% and 42%, respectively.

In Study MO25515, at baseline, most patients (2290/3222, 71.1%) had Stage M1c disease, and 89.2% had an ECOG PS of 1 or 2; 69% of patients had received prior systemic therapy for metastatic melanoma and 34.9% received prior radiotherapy. Results from the third interim analysis (clinical data cut-off date: 31 January 2013) for Study MO25515 (Blank et al. 2014; Queirolo et al. 2014) showed that 2409 patients (74.8%) had a BOR, including 90 (2.8%) CRs, 829 (25.7%) PRs, and 1490 (46.2%) SD. The median response duration was .7.3 months (95% CI: 6.9-7.5) and median PFS was 5.6 months (95% CI: 5.5-5.8). Median OS was 12.4 months (95% CI: 11.9-13.3). OS rates were as follows: 75.1% 6-month OS rate, 51.7% 12-month OS rate, and 35.6% 18-month OS rate of 77.3%.

1.3.2 Summary of Clinical Studies and Efficacy with Vemurafenib in Thyroid Carcinoma

1.3.2.1 PLX4032 Phase 1

As part of the phase 1, dose-escalation trial of vemurafenib, 3 patients with BRAF mutated papillary thyroid carcinoma (PTC) were treated (Flaherty, Puzanov et al. 2010) (Kim, Cabanillas et al. 2013). Among the 3 patients with PTC, one patient experienced a partial response and two patients experienced stable disease with a progression-free survival of 13 and 11 months.

1.3.2.2 Vemurafenib phase 2 in BRAF mutated PTC

A phase 2, open-label, multicenter clinical trial in adult patients with BRAFV600E mutated PTC was performed (Brose, Cabanillas et al. 2016). Patients were stratified into two cohorts—VEGFR multikinase inhibitor naïve and previously treated with VEGFR multikinase inhibitors. The primary endpoint was best overall response in cohort 1. Partial responses were observed in 38.5% of subjects in cohort 1. The median PFS and OS were 18.2 and 16.5 months, respectively. In cohort 2, a 27% partial response rate was observed. Median PFS and OS were 8.9 and 14.4 months, respectively, in this group. The drug was well tolerated.

1.3.2.3 Vemurafenib in non-melanoma BRAF mutated cancers (including ATC)

Six pre-specified cancer cohorts were enrolled on an open label trial with vemurafenib for various non-melanoma cancers(Hyman, Puzanov et al. 2015). A total of 122 patients with BRAFV600E mutation cancers were treated. Seven ATC patients were enrolled in

this trial. A complete response was observed in 1 (14%) patient (duration of >12.7 months), as well as 1 (14%) partial response (duration of 15.5 months). Four patients (57%) had a best response of progressive disease. One patient had missing information. Responses occurred early, at the first restaging visit.

1.3.3 Summary of Clinical Studies with Cobimetinib in Melanoma

The following are key clinical trials in the cobimetinib clinical development program for melanoma.

1.3.3.1 Phase I Cobimetinib Single-Agent Study (MEK4592)

Study MEK4592g was a multicenter, Phase I, non-randomized, open-label, dose-escalation study. The primary objectives of this study were to evaluate the safety, tolerability, and maximum tolerated dose (MTD) of cobimetinib administered orally as repeated doses in patients with solid tumors. In Stage I, 36 patients with advanced solid malignancies were enrolled in successive cohorts and received cobimetinib on a 21 -day on, 7-day off (21/7) dosing schedule at the following dose levels: 0.05 mg/kg, 0.10 mg/kg, and 0.20 mg/kg in liquid dosage formulation and 10 mg, 20 mg, 40 mg, 60 mg, and 80 mg in capsule formulation (Cohorts 1−8, respectively). The maximal administered dose (MAD) during Stage I was 80 mg; the MTD is 60 mg when cobimetinib is administered on a 21/7 dosing schedule. In Stage IA, 20 patients were treated with cobimetinib on a 14-day on, 14-day off (14/14) dosing schedule in successive cohorts at the following dose levels: 60 mg, 80 mg, 100 mg, and 125 mg in capsule formulation (Cohorts 1A − 4A, respectively). The MAD of Stage IA was 125 mg; and the MTD is 100 mg when cobimetinib is administered on a 14/14 dosing schedule. Stages II and IIA are expansion stages that further evaluate the safety, potential efficacy, and pharmacodynamic effects of cobimetinib at the MTDs determined in Stage I and Stage IA in patients with RAS- or RAF-mutant tumors. In Stage II, 20 patients were enrolled and received 60 mg cobimetinib on a 21/7 dosing schedule. In Stage IIA, 21 patients were enrolled and received 100 mg cobimetinib on a 14/14 dosing schedule. In Stage II, the dedicated DDI study, 20 patients with solid tumors received 60 mg QD 21/17.

1.4 COMBINED BRAF AND MEK INHIBITION

No trials using the combination of vemurafenib and cobimetinib have been performed in thyroid cancer.

Based on nonclinical studies to date, multiple mechanisms of acquired resistance to vemurafenib in $BRAF^{V600}$-mutated melanoma have been identified which may account for the majority of disease progression:

- Acquisition of activating RAS (NRAS or KRAS) mutations was reported, which accounts for the reactivation of ERK signaling and increased AKT survival signaling (Nazarian et al. 2010; Su et al. 2012).

- Spliced isoforms of $BRAF^{V600E}$ were identified which led to enhanced dimerization of $BRAF^{V600E}$ and reactivated the MAPK pathway (Poulikakos et al 2010).
- Increased PDGFRb or IGF1R activity was reported, which may enhance signaling through different RAF isoforms and/or the PI3K/AKT pathway to confer resistance (Nazarian et al. 2010; Villanueva et al. 2010).

Based on clinical validation data to date, the most prevalent mechanisms of acquired resistance to vemurafenib appear to be the first two mechanisms described above: the acquisition of activating NRAS mutations and spliced isoforms of BRAF ^{V600E}, which result in reactivation of ERK signaling in the presence of vemurafenib (Zubrilov et al. 2015; Spagnolo et al. 2015). The retention of the $BRAF^{V600E}$ mutation in both nonclinical models of vemurafenib resistance and biopsies from patients who progressed on vemurafenib suggests that continued suppression of the pathway by vemurafenib may be required to control tumor cell proliferation; however, reactivation of ERK signaling points to the potential for combination with another agent such as a MEK inhibitor that further inhibits the ERK signaling pathway and, consequently, overcomes the emergence of the pathway-specific resistance in tumors harboring the BRAF ^{V600E}. Synergistic anti-proliferative activity was observed when vemurafenib was combined with GDC-0973 in multiple $BRAF^{V600E}$ mutant cancer cell lines.

The in vivo combination of vemurafenib with GDC-0973 was also tested in the murine xenograft A375 $BRAF^{V600E}$ mutant melanoma model and in a model of A375 that had acquired resistance to vemurafenib (Baudy et al. 2012). In both models, the combination of vemurafenib and GDC-0973 is efficacious and well-tolerated and shows greater efficacy than either single agent alone at comparable doses.

The objective of the clinical investigation of the combination of vemuraf enib with GDC-0973 (MEK inhibitor) is to simultaneously inhibit both oncogenic BRAF kinase (vemurafenib) and MEK (GDC-0973) in patients with previously untreated, $BRAF^{V600E}$ mutation-positive, locally advanced and unresectable or metastatic melanoma OR those previously treated with vemurafenib in Phase I (PLX06-02, clinical pharmacology), Phase II, and Phase III clinical trials, with evidence of mixed progression.

1.4.1.1 Phase Ib Open-Label Cobimetinib and Vemurafenib Study (NO25395; BRIM-7)

Study NO25395 (BRIM-7) was a Phase Ib study designed to assess the safety, tolerability, and pharmacokinetics of combined MEK inhibition with cobimetinib and BRAF inhibition with vemurafenib. This multicenter study had two stages: a dose-escalation stage and a cohort-expansion stage. This study was being conducted in patients with $BRAF^{V600}$ mutation-positive, unresectable locally advanced or metastatic melanoma who were either vemurafenib naïve or had progressed on vemurafenib treatment.

All patients in the dose-escalation stage received vemurafenib (720 mg or 960 mg) twice daily in combination with cobimetinib (60 mg, 80 mg, or 100 mg) administered daily according to one of the following 28-day schedules:

- 14 consecutive days of study drug followed by a 14-day drug holiday (14/14)
- 21 consecutive days of study drug followed by a 7-day drug holiday (21/7)
- Continuous daily dose (28/0).

Each treatment cycle was 28 days.

There were 10 dose-escalation cohorts of 3−6 patients per cohort. Patients in Cohort 1 received vemurafenib at a dose of 720 mg BID continuously and cobimetinib 60 mg once daily (QD) for 14 consecutive days of each 28-day cycle of combination dosing (14/14). Dose-escalation used the standard 3 + 3 design and proceeded in increments, taking into account the safety and tolerability of the combination.

Cohorts 1A and 1B were expansion cohorts because both cohorts were declared safe and tolerable after dose escalation; furthermore, they delivered the single-agent maximum tolerated dose and schedule of cobimetinib and, in the case of cohort 1B, the approved dose and schedule of vemurafenib (Ribas et al. 2014a; Ribas et al. 2014b; Pavlick et al. 2015).

1.4.1.2 Randomized Phase III Study of Vemurafenib and Cobimetinib (GO28141; CoBRIM)

This randomized, open-label, multicenter, Phase III study assessed previously untreated patients with metastatic melanoma confirmed by histopathology (unresectable stage IIIC or stage IV) and with a $BRAF^{V600}$ mutation by the cobas□ 4800 $BRAF^{V600}$ Mutation Test (Larkin et al. 2014). In this study, 495 patients were randomly assigned to receive vemurafenib (960 mg BID PO) and cobimetinib (60 mg QD PO) for the first 21 days of each 28 day cycle (combination group) or vemurafenib and placebo (control group). The primary end point was investigator-assessed PFS according to RECIST Version 19.1. The secondary endpoints included OS, rate of confirmed objective response (RECIST v1.1), DOR, IRC-assessed PFS, and safety. The final analysis was planned to occur after 206 events, which was reached in May 2014.

1.4.2 <u>Clinical Efficacy of Cobimetinib and Vemurafenib in Melanoma</u>

1.4.2.1 Study NO25395 (BRIM-7)

Patients were evaluable for efficacy if they had received combination treatment, had measurable disease at baseline, and had either at least one post -baseline tumor assessment or evidence of progression prior to tumor assessment. A total of 129 patients were enrolled and received vemurafenib plus cobimetinib; 66 patients had recently progressed on vemurafenib monotherapy and 63 had never received a RAF inhibitor. In the recently progressed patients, there was no CR; however, the remaining response rates were as follows: PR = 10 (15%) patients, SD = 28 (42%) patients,

PD = 24 (36%) patients, and 4 (6%) were not assessed. In the BRAF-inhibitor naïve patients, the response rates were as follows: CR = 6 (10%) patients, PR = 49 (78%) patients, SD = 6 (10%) patients, and PD = 2 (3%) had PD (Ribas et al. 2014a).

1.4.2.2 Study GO28141 (CoBRIM)

The initial results were reported based on data analyses from July 2014 after the prespecified number of progression events was reached in May 2014 (Larkin et al. 2014). Updated results were presented at ASCO 2015 (Larkin et al. 2015) based on data cut-off date of 16 January 2015.

Vemurafenib plus cobimetinib was significantly superior to vemurafenib alone: median PFS was 12.3 months in the combination group and 7.2 months in the vemurafenib group (HR 0.58 [95% CI: 0.46-0.72]). The PFS benefit was observed across key subgroups including LDH levels and BRAF mutation type. BORR was 69.9% (95% CI: 63.5-75.3) with 15.8% CRs in the combination arm and 50% (95% CI: 43.6-56.4) with 10.5% CRs in the single-agent arm (Larkin et al. 2015).

OS data are still not mature but interim analysis (data cut-off date of May 2014; Larkin et al. 2014) showed 9-month OS rate of 81% (95% CI: 75-87) for the combination group and 73% (95% CI: 65-80) for the monotherapy group and a HR of 0.65 (95% CI: 0.42 - 1.00). The study continues to follow patients for OS; the final OS analysis is expected by the end of 2015.

1.4.3 <u>Safety Profile: Combined Vemurafenib and Cobimetinib Safety Summary</u>

1.4.3.1 Study NO25395 (BRIM-7)

In Study NO25395, similar types of AEs occurred among the BRAF inhibitor-naïve (BRAFi-naïve) patients and vemurafenib-PD patients however, AEs were less frequently reported in vemurafenib-PD than in BRAFi-naïve patients. This difference was also apparent for Grade □ 3 AEs and SAEs with lower rates of incidence in vemurafenib-treated patients with PD, and may reflect that vemurafenib-treated patients with PD had previously demonstrated ability to tolerate vemurafenib, as well as had shorter duration of exposure to study drugs.

The most common AEs (□ 10%) are summarized by group in Table 1.

Table 1 Treatment-Emergent Adverse Events That Occurred in 10% or More of Patients in Study NO25395 (BRIM-7)

MedDRA System Organ Class<br>MedDRA Preferred Term	BRAFi-naive<br>n = 63	Vemurafenib-PD<br>n = 66	All patients<br>n = 129
Any Adverse Events	63 (100.0%)	61 (92.4%)	124 (96.1%)
**Gastrointestinal Disorders**	56 (88.9%)	44 (66.7%)	100 (77.5%)

MedDRA System Organ Class<br>MedDRA Preferred Term	BRAFi-naive<br>n = 63	Vemurafenib-PD<br>n = 66	All patients<br>n = 129
Diarrhea	52 (82.5%)	31 (47.0%)	83 (64.3%)
Nausea	36 (57.1%)	22 (33.3%)	58 (45.0%)
Vomiting	27 (42.9%)	13 (19.7%)	40 (31.0%)
Abdominal Pain	12 (19.0%)	10 (15.2%)	22 (17.1%)
Constipation	10 (15.9%)	9 (13.6%)	19 (14.7%)
**Skin and Subcutaneous Tissue Disorders**	62 (98.4%)	33 (50.0%)	95 (73.6%)
Photosensitivity Reaction	42 (66.7%)	10 (15.2%)	52 (40.3%)
Rash	32 (50.8%)	18 (27.3%)	50 (38.8%)
Pruritus	18 (28.6%)	7 (10.6%)	25 (19.4%)
Dermatitis Acneiform	19 (30.2%)	5 (7.6%)	24 (18.6%)
Rash Maculopapular	19 (30.2%)	2 (3.0%)	21 (16.3%)
Dry Skin	15 (23.8%)	2 (3.0%)	17 (13.2%)
Alopecia	9 (14.3%)	7 (10.6%)	16 (12.4%)
Erythema	14 (22.2%)	2 (3.0%)	16 (12.4%)
Actinic Keratosis	11 (17.5%)	2 (3.0%)	13 (10.1%)
**General Disorders and Administration Site Conditions**	53 (84.1%)	32 (48.5%)	85 (65.9%)
Fatigue	44 (69.8%)	18 (27.3%)	62 (48.1%)
Pyrexia	27 (42.9%)	11 (16.7%)	38 (29.5%)
Edema Peripheral	26 (41.3%)	11 (16.7%)	37 (28.7%)
Chills	17 (27.0%)	10 (15.2%)	27 (20.9%)
**Investigations**	48 (76.2%)	22 (33.3%)	70 (54.3%)
Blood Creatine Phosphokinase Increased	27 (42.9%)	10 (15.2%)	37 (28.7%)
Blood Alkaline Phosphatase Increased	21 (33.3%)	10 (15.2%)	31 (24.0%)
Aspartate Aminotransferase Increased	22 (34.9%)	6 (9.1%)	28 (21.7%)
Alanine Aminotransferase Increased	23 (36.5%)	4 (6.1%)	27 (20.9%)
Blood Creatinine Increased	20 (31.7%)	6 (9.1%)	26 (20.2%)
Blood Bilirubin Increased	8 (12.7%)	6 (9.1%)	14 (10.9%)
**Musculoskeletal and Connective Tissue Disorders**	36 (57.1%)	23 (34.8%)	59 (45.7%)
Arthralgia	30 (47.6%)	8 (12.1%)	38 (29.5%)
Myalgia	15 (23.8%)	4 (6.1%)	19 (14.7%)
Back Pain	7 (11.1%)	10 (15.2%)	17 (13.2%)

MedDRA System Organ Class<br>MedDRA Preferred Term	BRAFi-naive<br>n = 63	Vemurafenib-PD<br>n = 66	All patients<br>n = 129
Pain In Extremity	10 (15.9%)	4 (6.1%)	14 (10.9%)
**Nervous System Disorders**	22 (34.9%)	18 (27.3%)	40 (31.0%)
Headache	17 (27.0%)	13 (19.7%)	30 (23.3%)
Dizziness	8 (12.7%)	9 (13.6%)	17 (13.2%)
**Metabolism and Nutrition Disorders**	22 (34.9%)	17 (25.8%)	39 (30.2%)
Decreased Appetite	15 (23.8%)	14 (21.2%)	29 (22.5%)
Hypokalemia	10 (15.9%)	4 (6.1%)	14 (10.9%)
**Injury, Poisoning and Procedural Complications**	20 (31.7%)	15 (22.7%)	35 (27.1%)
Sunburn	20 (31.7%)	15 (22.7%)	35 (27.1%)
**Blood and Lymphatic System Disorders**	20 (31.7%)	10 (15.2%)	30 (23.3%)
Anemia	20 (31.7%)	10 (15.2%)	30 (23.3%)
Vascular Disorders	17 (27.0%)	6 (9.1%)	23 (17.8%)
Hypertension	17 (27.0%)	6 (9.1%)	23 (17.8%)
**Infections and Infestations**	14 (22.2%)	6 (9.1%)	20 (15.5%)
Upper Respiratory Tract Infection	14 (22.2%)	6 (9.1%)	20 (15.5%)
Respiratory, Thoracic and Mediastinal Disorders	12 (19.0%)	6 (9.1%)	18 (14.0%)
Cough	12 (19.0%)	6 (9.1%)	18 (14.0%)
Eye Disorders	11 (17.5%)	2 (3.0%)	13 (10.1%)
Vision Blurred	11 (17.5%)	2 (3.0%)	13 (10.1%)

Multiple occurrences of a specific adverse event for a patient were counted once at the highest NCI CTCAE grade of these occurrences. For example, if a patient experienced two events with a specific preferred term, one Grade 3 and one Grade 4, the patient would be counted only once in the Grade 4 row. Similarly, in the ’Any adverse events’ and the ’Overall’ rows, if a patient experienced, for example, three separate events of Grade 1, 3, and 4, the patient would be counted only once in the Grade 4 row.

Source: Table 21 in CSR NO25395.Data cutoff: 1 October 2013.

In Study NO25395, 62.0% of patients experienced at least one Grade □ 3 AE; 52.7% experienced a Grade 3 AE, 9.3% a Grade 4 AE, and there were no AEs of Grade 5.

Among the BRAFi-naive patients, the most common Grade □ 3 (occurring in □ 5% of patients) were anemia, diarrhea, fatigue, increased blood ALP, increased ALT, increased AST, hypophosphatemia, arthralgia, SCC, maculopapular rash, and hypertension. Among the vemurafenib-PD patients, the most common Grade □ 3 AEs (occurring in □ 5% of patients) were SCC and anemia.

SAEs were reported in 33.3% of all patients. The majority (55 of 77 patients) were considered not related to either study drug based on investigator assessment. SCC of the skin was reported as an SAE in 9.5% of BRAFi-naive patients versus 4.5% of vemurafenib-PD patients. No other SAE was reported in more than 3% of patients in either group.

Twelve BRAFi-naive patients (19%) and 45 vemurafenib-PD patients (68.2%) had died as of the clinical cutoff date.

All 12 deaths in BRAFi-naive patients and 43 of the 45 deaths in the vemurafenib -PD population were attributed to disease progression, and the majority of deaths occurred □ 30 days after last study drug administration. Two vemurafenib-PD patients (3%) died from unknown causes after being off the study for disease progression. No deaths in Study NO25395 were attributed to Grade 5 AEs.

Patients experiencing AESIs requiring expedited reporting that were pre-specified for Study NO25395 are summarized in Table 2.

Table 2 Summary of Patients Experiencing Protocol-Defined AESIs Irrespective of Relationship to Study Drug in Study NO25395

AESI	Vemurafenib-PD patients (n = 66)	BRAFi-naive patients (n = 63)	All Patients in Dose-Escalation and Expansion Stages (n = 129)
Grade □ 1 retinal vein occlusion	0 (0%)	0 (0%)	0 (0%)
Grade □ 2 Visual disturbances			
Grade □ 2 Retinal detachment/retinopathy	0 (0%)	2 (3.2%)	2 (1.6%)
Grade □ 2 Other visual disturbances	3 (4.5%)	9 (14.3%)	12 (9.3%)
cuSCC/keratoacanthoma	5 (7.6%)	7 (11.1%)	12 (9.3%)
Grade □ 3 QTc interval prolongation	2 (3.0%)	4 (6.3%)	6 (4.7%)
Grade □ 3 photosensitivity	1 (1.5%)	2 (3.2%)	3 (2.3%)
Grade 4 Liver Function Test (ALT, AST and/or bilirubin) Abnormality	0 (0%)	0 (0%)	0 (0%)

ALT= alanine aminotransferase; AST= aspartate aminotransferase.

1.4.3.2 Study GO28141 (CoBRIM)

Most patients in both treatment arms (□ 97.5%) experienced at least one AE. The most common AEs, by system organ class, reported in the cobimetinib + vemurafenib arm were: skin and subcutaneous tissue disorders (83.3%), GI disorders (78%), investigations (68.1%), and general disorders and administrative site conditions (66.9%).

Table 3 tabulates the most common AEs occurring in 20% or more of patients in either study arm by preferred term. The most common AEs that occurred with greater frequency in the cobimetinib plus vemurafenib arm were diarrhea (56.7% vs. 28%), rash (39% vs. 35.6%), nausea (39% vs. 23.8%), blood creatine phosphokinase increased (29.9% vs. 2.9%), photosensitivity reaction (28.3% vs. 15.9%), pyrexia (26% vs. 22.2%), alanine aminotransferase (ALT) increased (23.6% vs. 18%), aspartate aminotransferase (AST) increased (22% vs. 12.6%), and vomiting (21.3% vs. 12.1%).

Table 3 Most Common Adverse Events (Occurring in ≥ 20% of Patients) in Study GO28141

	Vemurafenib +placebo n = 239	Vemurafenib +cobimetinib n = 254
Diarrhea	67 (28%)	144 (56.7%)
Rash	85 (35.6%)	99 (39%)
Arthralgia	96 (40.2%)	83 (32.7%)
Fatigue	74 (31%)	82 (32.3%)
Nausea	57 (23.8%)	99 (39%)
Pyrexia	53 (22.2%)	66 (26%)
Photosensitivity	38 (15.9%)	72 (28.3%)
Alopecia	70 (29.3%)	35 (13.8%)
ALT increase	43 (18%)	60 (23.6%)
Hyperkeratosis	68 (28.5%)	26 (10.2%)
AST increase	30 (12.6%)	56 (22%)
CPK increase	7 (2.9%)	76 (29.9%)
Vomiting	29 (12.1%)	54 (21.3%)

ALT=alanine aminotransferase; AST=aspartate aminotransferase; CPK=creatine phosphokinase.

At least one Grade ≥ 3 AE was reported in 65% of patients treated with cobimetinib + vemurafenib and 59% of patients treated with placebo + vemurafenib. Table 4 tabulates the Grade ≥ 3 AEs occurring in at least 2% of patients in either arm.

The most common Grade ≥ 3 AEs reported at a higher frequency (2% difference) in patients treated with cobimetinib + vemurafenib than in patients treated with placebo + vemurafenib were ALT increased (11.4% vs. 6.3% of patients), blood creatine phosphokinase increased (10.2% vs. 0%), AST increased (8.3% vs. 2.1%), diarrhea (6.3% vs. 0%), blood alkaline phosphatase increased (4.3% vs. 1.7%), hyponatremia (2.4% vs. 0.4%), photosensitivity reaction (2.4% vs. 0%), and retinal detachment (2.4% vs. 0%).

Table 4 Grade □ 3 Adverse Events Occurring in at Least 2% of Patients in Either Arm (Safety-Evaluable Population)

MedDRA Preferred Term for Grade 3 events	Vemurafenib + placebo (n = 239)	Vemurafenib + cobimetinib (n = 254)	All patients (n = 493)
□Glutamyltransferase increased	25 (10.5%)	30(11.8%)	55 (11.2%)
ALT increase	15 (6.3%)	29 (11.4%)	44 (8.9%)
Squamous cell carcinoma of skin	27 (11.3%)	6 (2.4%)	44 (8.9%)
Rash maculo-papular	13 (5.4%)	16 (6.3%)	29 (5.9%)
Rash	12 (5.0%)	15 (5.9%)	27 (5.5%)
AST increase	27 (11.3%)	6 (2.4%)	33 (6.7%)
CPK increase	0	26 (10.2%)	26 (5.3%)
Keratoacanthoma	18 (7.5%)	2 (0.8%)	20 (4.1%)
Arthralgia	12 (5.0%)	6 (2.4%)	18 (3.7%)
Fatigue	8 (3.3%)	10 (3.9%)	18 (3.7%)
Diarrhea	0	16 (6.3%)	16 (3.2%)
Hypertension	6 (2.5%)	10 (3.9%)	16 (3.2%)
Basal cell carcinoma	5 (2.1%)	10 (3.9%)	15 (3.0%)
Blood alkaline phosphatase increased	4 (1.7%)	11 (4.3%)	15 (3.0%)
Dermatitis acneiform	4 (1.7%)	6 (2.4%)	10 (2.0%)
Pain in extremity	6 (2.5%)	3 (1.2%)	9 (1.8%)
Hyponatremia	1 (0.4%)	6 (2.4%)	7 (1.4%)
Myalgia	6 (2.5%)	1 (0.4%)	7 (1.4%)
Photosensitivity reaction	0	6 (2.4%)	6 (1.2%)
Retinal detachment	0	6 (2.4%)	6 (1.2%)
Hyperkeratosis	5 (2.1%)	0	5 (1.0%)

ALT=alanine aminotransferase; AST=aspartate aminotransferase; CPK=creatine phosphokinase.

Serious adverse events (SAEs) occurred in 75 patients (29.5%) treated with cobimetinib + vemurafenib, of which, 46 SAEs were considered related to study treatment. SAEs occurred in 60 patients (25.1%) treated with placebo + vemurafenib, of which, 35 SAEs were considered related to study treatment.

The three most common SAEs in patients treated with cobimetinib + vemurafenib were pyrexia (2.4% of patients), dehydration (2.0%), and rash (1.6%). The three most

common SAEs in patients treated with placebo + vemurafenib were pyrexia, keratoacanthoma, and pleural effusion (1.3% each).

As of the clinical cutoff date, 36 of 254 patients (14.2%) who received cobimetinib + vemurafenib and 48 of 239 patients (20.1%) who received placebo + vemurafenib had died. In most cases, the primary cause of death was attributed to disease progression.

Six patients in the cobimetinib + vemurafenib arm and 3 patients in the placebo ▪ vemurafenib arm were reported to have Grade 5 AEs. There were no patterns with respect to the type of Grade 5 events reported, and for most events the patient's underlying disease was considered to contribute to the cause of the event.

Of the 6 patients with Grade 5 AEs in the cobimetinib ▪ vemurafenib arm, the AE was recorded as the primary cause of death for 2 patients ("cardiac arrest" and "pneumonia", respectively). An additional 2 patients with reported Grade 5 events ("unexplained death", and "asthenia and fatigue") had primary cause of death recorded as "other." The remaining 2 patients with reported Grade 5 events ("hemiparesis" and "cerebral hemorrhage") had disease progression recorded as the primary cause of death.

Of the 3 patients with AEs graded 5 in the placebo plus vemurafenib arm, 1 patient was reported to have died from "cardiac failure" while 2 patients with Grade 5 AEs of "fatigue" and "pulmonary embolism", respectively were reported to have died from PD.

The AESIs, as defined in the study protocol comprised any RVO, serous retinopathy, Grade ▪ 3 photosensitivity, Grade ▪ 2 reduction in LVEF, Grade ▪ 3 elevations in liver function tests, Grade ▪ 3 QTc prolongation, any cutaneous primary malignancy, and Grade ▪ 3 rash. A summary of AESI observed in the study is shown in Table 5.

Table 5 Summary of Adverse Events of Special Interest (Safety-Evaluable Population)

AE Special Interest	Vemurafenib+ placebo n = 239	Vemurafenib+ cobimetinib n = 254	All patients n = 493
Retinal Vein Occlusion	0	0	0
Retinal Detachment or Central Serous Retinopathy	5 (2.1%)	61 (24.0%)	66 (13.4%)
Photosensitivity Grade ≥ 3	0	8 (3.1%)	8 (1.6%)
LVEF Reduction Grade ≥ 2	7 (2.9%)	17 (6.7%)	24 (4.9%)
Liver Function Tests Grade ≥ 3	36 (15.1%)	52 (20.5%)	88 (17.8%)
QTc Interval Prolongation Grade ≥3	4 (1.7%)	4 (1.6%)	8 (1.6%)
Cutaneous Primary Malignancy	47 (19.7%)	21 (8.3%)	68 (13.8%)
Rash Grade ≥ 3	38 (15.9%)	41 (16.1%)	79 (16.0%)

1.5 SAFETY PROFILE: VEMURAFENIB MONOTHERAPY

This section describes the safety profile of vemurafenib as monotherapy. This section summarizes safety findings, such as the incidence of AEs (including AEs leading to death, Grade 3-4 AEs, SAEs, vemurafenib-related AEs, and AEs leading to withdrawal of vemurafenib) and notable laboratory test results. Overall, most of the safety experience presented in the IB is based on patients undergoing treatment dosed at 960 mg BID vemurafenib. The details about safety in combination therapy studies are available in respective IBs.

1.5.1 Safety in Healthy Subjects

Single doses of vemurafenib were well tolerated in healthy subjects (n = 18) enrolled in a relative bioavailability study (PLX102-01). No serious AEs and no study treatment discontinuations due to AEs were reported. All AEs were considered mild or moderate in intensity, and approximately half were considered by the investigator to be possibly related. The AEs most commonly reported were headache (22.2%), dizziness, somnolence, rash, nausea, and decreased white blood cells (11.1% each). No clinically significant changes or findings were noted from clinical laboratory evaluations, vital sign measurements, physical examinations, or 12-lead ECGs for this study.

1.5.2 Safety in Patients

1.5.2.1 Overview of Adverse Events

The following four clinical trials are the source of the vast majority of clinical safety data (AEs, serious AEs [SAEs], AEs that led to treatment discontinuation, deaths, and events to monitor) to support the use of vemurafenib for the treatment of patients with *BRAF V600* mutation-positive unresectable or metastatic melanoma:

- NO25026, Phase III: Results of the final analysis are provided on the basis of the 8 July 2015 clinical cutoff date.
- NP22657, Phase II: Results iof the final analysis are provided on the basis of the 3 June 2014 clinical cutoff date.
- NP25163, Phase I clinical pharmacology study: clinical cut-off: 30 May 2013; n = 52
- MO25515, Post approval safety study: Interim Analysis: Feburary 2012, n = 2398.

A summary of safety data is presented below.

1.5.2.1.1 Study NO25026

The Final CSR (LPLV: 8 July 2015) provides cumulative safety results from the start of the study until all patients have discontinued from or completed the study. All AEs reported during the study were included. For dacarbazine patients who crossed over to receive vemurafenib, AEs with an onset date before the date of crossover are summarized separately. An overview of AEs reported in this study prior to cross over is provided in Table 6.

Almost all patients (99% in vemurafenib group, 93% in dacarbazine group) in this study experienced at least one AE. Ten patients experienced AEs that resulted in death, 6 in the vemurafenib arm and 4 in the dacarbazine arm (prior to crossover). Two of these events were considered related to study treatment (one to vemurafenib [intracranial tumor hemorrhage] and one to dacarbazine [shock]). After crossover, an additional patient (randomized to dacarbazine treatment group prior to crossover) experienced an AE that resulted in death (pneumonia), which was considered not related to study treatment.

In the vemurafenib treatment group, the most commonly reported (≥ 20% of patients) AEs were arthralgia (56%); alopecia (48%); fatigue (47%); rash (43%); photosensitivity reaction (40%); nausea (39%); diarrhea (37%); headache (34%); hyperkeratosis and skin papilloma (29% each); pruritus (26%); dry skin (24%); pain in extremity and decreased appetite (23% each); vomiting and pyrexia (22% each); and SCC of skin (20%). In the dacarbazine treatment group, the most commonly reported AEs (≥ 20% of patients) were nausea (45%), fatigue (35%), vomiting (27%), and constipation (25%). Most patients had at least 1 AE that was considered by the investigator to be related to

vemurafenib (328 patients [98%]) or dacarbazine (204 patients [71%]).

Table 6 Study NO25026: Overview of AEs (Safety Population)

	Vemurafenib (N = 336) n (%)	Dacarbazine (N = 287) n (%)
Patients with any AE	334 (99)	266 (93)
Grade ≥ 3 AEs	236 (70)	106 (37)
AEs resulting in death	6 (2)	4 (1)
Deaths [a]	271 (81) [b]	170 (58) [b]
Deaths within 28 days of last dose of study treatment [a]	44 (13) [c]	17 (6) [c]
SAEs	165 (49)	52 (18)
AEs that led to study or study treatment discontinuation	25 (7)	5 (2)

AE = adverse event; SAE = serious adverse event.

Notes: Percentages were based on N. Only the most severe intensity was counted for multiple occurrences of the same AE in 1 individual. For dacarbazine-treated patients who crossed over to receive vemurafenib, only AEs with an onset date before the date of crossover were summarized.

[a] Deaths were based on the all treated population, where N = 336 in the vemurafenib and N = 293 in the dacarbazine treatment groups. Only deaths in dacarbazine-treated patients that occurred before the date of crossover were included.

[b] In the vemurafenib and dacarbazine treatment groups, 255 and 163 deaths, respectively, were due to disease progression.

[c] In the vemurafenib and dacarbazine treatment groups, 38 and 13 deaths, respectively, were due to disease progression.

Source: Study NO25026, Final CSR 1067390.

1.5.2.1.2 Study NP22657

An overview of AEs and deaths using a clinical cutoff date, 3 June 2014, in Study NP22657 is presented in Table 7. All 132 (100%) patients had at least one AE. Treatment-related AEs occurred in 130 (98%) patients. The majority of AEs were of mild or moderate intensity. The most common AEs (reported in ≥ 50% of patients) were arthralgia (70%), fatigue (60%), and photosensitivity reaction (54%).

Table 7 Study NP22657: Overview of AEs and Deaths (Safety Population)

	n (%)
No. of Patients	132
Patients with at least one AE	132 (100)
Treatment-related AEs	130 (98)
AEs of Grade ≥ 3	102 (77)
Treatment-related AEs of Grade ≥ 3	85 (64)
Deaths [a]	99 (75)
Deaths within 28 days of last vemurafenib dose	20 (15)
SAEs	79 (60)
Treatment-related SAEs	57 (43)
AEs that led to treatment discontinuation	4 (3)

Clinical cutoff: 3 June 2014.

[a] 94 of these 99 deaths were due to disease progression.

1.5.2.1.3 Study NP25163

An overview of AEs and deaths in Study NP25163 is presented in Table 6 and Table 7. All 52 (100%) patients had at least one AE. Fifty patients (96%) had at least one treatment-related AE. The majority of AEs were of mild or moderate intensity. The most

common AEs overall (≥ 20%) were fatigue, which was reported by 32 patients (62%), followed by (in order of decreasing incidence) arthralgia (31 patients, 60%), nausea (26 patients, 50%), rash (21 patients, 40%), diarrhea (20 patients, 38%), hyperkeratosis and decreased appetite (17 patients each, 33%), alopecia (16 patients, 31%), constipation, vomiting, pain in extremity, and sunburn (15 patients each, 29%), erythema (14 patients, 27%), photosensitivity reaction and cough (13 patients each, 25%), actinic keratosis, myalgia, seborrheic keratosis, weight decreased, and headache (12 patients each, 23%), and edema peripheral and skin papilloma (11 patients each, 21%). All other AEs were reported in fewer than 20% of patients, overall.

Table 8 NP25163: Overview of AEs and Deaths (Safety Population)

	n (%)
**No. of Patients**	**52**
Patients with at least one AE	52 (100)
Treatment-related AEs	50 (96)
AEs of Grade ≥ 3	32 (62)
Treatment-related AEs of Grade ≥ 3	25 (48)
Deaths	25 (48)
Deaths within 28 days of last vemurafenib dose	10 (19)
SAEs	21 (40)
Treatment-related SAEs	15 (29)
AEs that led to treatment discontinuation	3 (6) [a]

Clinical cutoff: LPLV, 19 February 2013.
[a] A fourth patient was discontinued from the study with a reason for withdrawal of 'Other,' although the investigator then specifically identified an AE of hyponatremia (Grade 4) as the reason for withdrawal.

1.5.2.1.4 Study MO25515

Table 25 Overall Summary of Adverse Events and Deaths (Safety Population)

	Vemurafenib (N=3219) n (%)
Number of patients with at least 1:	
Any AE	3121 (97.0)
SAE	1114 (34.6)
Grade 3 or 4 AE	1701 (52.8)
Grade 5 AE	88 (2.7)
AE leading to Study Drug Discontinuation	225 (7.0)
AE of Special Interest:	2723 (84.6)
Arthralgia	1363 (42.3)
Rash	1543 (47.9)
Photosensitivity	915 (28.4)
Fatigue	1186 (36.8)
Cutaneous SCC	471 (14.6)
Non Cutaneous SCC	3 (0.1)
New Primary Melanoma	54 (1.7)
GI polyps	1 (<0.1)
Pancreatitis	8 (0.2)
Potentiation of radiation toxicity	44 (1.4)
AEs Potentially Associated with Prolongation of Cardiac Repolarization or Arrhythmia	548 (17.0)
Other primary malignancy (other than cSCC or new primary melanoma)	102 (3.2)
Liver Injury	453 (14.1)
Number of deaths by primary cause for:	
Deaths within 30 days after First Dose	39 (1.2)
Disease Progression	25 (0.8)
Adverse Event	12 (0.4)
Other	2 (0.1)
Deaths within 28 days after Last Dose	807 (25.1) ~
Disease Progression	716 (22.2) ~
Adverse Event	80 (2.5)
Unknown	1 (<0.1)
Other	10 (0.3)
All Deaths	2054 (63.8)
Disease Progression	1932 (60.0)
Adverse Event *	85 (2.6)
Unknown	12 (0.4)
Other	25 (0.8)

Percentages are based on the number of patients in the population.
~ Patients 2096350026, 2102200029 and 2367130028 died but had their date of last dose missing, so it is not known how long after the last dose the deaths occurred. Following a conservative approach these patients were included in this assessment.
* Three patients had Grade 5 AEs but their primary cause of death was incorrectly recorded as 'other' in the eCRF and not as an AE (see Section 6.5.1).
Source: Adapted from Post-text Table 14.3.1, Section 11.5.1 and Table 14.3.2.4, Section 11.5.2.

1.5.2.2 Grade 3 and Grade 4 Adverse Events

1.5.2.2.1 Study NO25026

In the Phase III study NO25026, a greater percentage of patients had Grade ≥ 3 AEs in the vemurafenib treatment group (70%) than the dacarbazine treatment group (37%). The most commonly reported (≥ 5% of patients) Grade ≥ 3 AEs were SCC of skin (20%), keratoacanthoma (11%), rash (9%), and arthralgia (6%) in the vemurafenib treatment group and neutropenia (9%) in the dacarbazine treatment group.

The overall incidence of Grade 4 AEs with onset dates prior to crossover was similar in the vemurafenib (23 patients [7%] with 26 AEs) and dacarbazine (27 patients [9%] with 34 AEs) treatment groups. Grade 4 AEs in the vemurafenib treatment group included pulmonary embolism (4 patients); increased blood creatine phosphokinase, increased GGT, and pneumonia (2 patients each); and acute kidney injury, confusional state, coronary artery disease, general physical health deterioration, hyperkalemia, hyponatremia, ileus, increased blood bilirubin, intraventricular hemorrhage, myocardial infarction, neutropenia, pneumothorax, respiratory distress, spinal cord compression, stridor, and tonsil cancer (1 patient each). In the dacarbazine treatment group, Grade 4 AEs included neutropenia (9 patients); decreased neutrophil count (4 patients); thrombocytopenia (3 patients); dyspnea (2 patients); and brain edema, cardiac tamponade, cerebral hemorrhage, decreased platelet count, deep vein thrombosis, hematochezia, hydronephrosis, hypercalcemia, hyperkalemia, hyperuricemia, hyponatremia, intraventricular hemorrhage, lower abdominal pain, lung infection, pleural effusion, and pulmonary embolism (1 patient each).

The incidence of Grade 5 events (AEs resulting in death) was similar in the vemurafenib and dacarbazine treatment groups (2% and 1%, respectively). During the total study period, 14 patients with Grade 3 treatment-related AEs discontinued treatment with vemurafenib (12 patients) and dacarbazine (2 patients). A total of 6 patients (2%) in the vemurafenib treatment group had 7 Grade 4 AEs that were considered by the investigator to be related to vemurafenib including 3 AEs of elevations in liver function tests: increased blood bilirubin (1 patient) and increased GGT (2 patients). The other Grade 4 AEs considered related to vemurafenib were increased blood creatine phosphokinase (2 patients), tonsil cancer (1 patient), and neutropenia (1 patient). Of the Grade 4 AEs detailed in the dacarbazine treatment group above, all cases of neutropenia and decreased neutrophil count were considered by the investigator to be related to dacarbazine. Other Grade 4 AEs considered related to dacarbazine included thrombocytopenia (3 patients) and decreased platelet count, deep vein thrombosis, and pulmonary embolism (1 patient each).

1.5.2.2.2 Study NP22657

In Study NP22657, majority of patients (102/132; 77.3%) experienced at least one Grade 3 AE. AEs of Grade 3 that were considered by the investigator to be related to vemurafenib treatment were reported for 85 (64.4%) patients, with cutaneous squamous cell carcinoma ([cuSCC] cuSCCs were protocol-specified to be reported as Grade 3 AEs) most commonly reported (34 patients [25.8%]).

During the total study period, 13 (9.8%) patients experienced at least one AE of Grade 4 intensity. Five (3.8%) patients reported six Grade 4 AEs that were considered by the investigator to be related to vemurafenib treatment. These five patients included three patients with increased GGT, one patient with both increased GGT and increased

conjugated bilirubin, and one patient with hyperuricemia.

With the exception of the increase in GGT and conjugated bilirubin in the one patient, none of the other Grade 4 AEs were reported as serious AEs, and none led to discontinuation of vemurafenib.

1.5.2.2.3 Study NP25163

In the NP25163 study, 32 patients (62%) had at least one Grade 3 AE, and 5 patients (10%) had at least one Grade 4 AE. There were no Grade 5 (fatal) AEs. Twenty-five patients (48%) had at least one related Grade 3 AE, and 2 patients (4%) had at least one related Grade 4 AE. The SOC associated with the most Grade 3 -4 AEs was ‘Neoplasms benign, malignant, and unspecified (including cysts and polyps),’ in which 13 events were reported in 12 patients (23%): cuSCC (7 patients, 13%), keratoacanthoma (4 patients, 8%), and malignant melanoma and SCC of the oral cavity (1 patient each, 2%). Two of the 5 patients that had Grade 4 AEs of GGT increased and shock were assessed as treatment-related.

1.5.2.2.4 Study MO25515

Table 32 Adverse Events of Grade 3 and 4 Occurring in ≥ 1% of Patients (Safety Population)

System Organ Class<br>Preferred Term	Vemurafenib (N=3219)<br>n	(%)	E	95% CI
Number of patients with at least 1 Grade 3 or 4 AE	1701	(52.8)	3583	(51.1, 54.6)
Neoplasms Benign, Malignant And Unspecified (Incl Cysts And Polyps)	578	(18.0)	925	(16.6, 19.3)
Squamous Cell Carcinoma Of Skin	261	(8.1)	359	(7.2, 9.1)
Keratoacanthoma	260	(8.1)	360	(7.2, 9.1)
Basal Cell Carcinoma	65	(2.0)	75	(1.6, 2.6)
Malignant Melanoma	43	(1.3)	46	(1.0, 1.8)
Investigations	315	(9.8)	467	(8.8, 10.9)
Alanine Aminotransferase Increased	61	(1.9)	65	(1.5, 2.4)
Gamma-Glutamyltransferase Increased	59	(1.8)	70	(1.4, 2.4)
Electrocardiogram Qt Prolonged	54	(1.7)	64	(1.3, 2.2)
Blood Alkaline Phosphatase Increased	46	(1.4)	49	(1.0, 1.9)
Aspartate Aminotransferase Increased	37	(1.1)	40	(0.8, 1.6)
Blood Bilirubin Increased	32	(1.0)	38	(0.7, 1.4)
Skin And Subcutaneous Tissue Disorders	262	(8.1)	306	(7.2, 9.1)
Rash	44	(1.4)	46	(1.0, 1.8)
Photosensitivity Reaction	41	(1.3)	43	(0.9, 1.7)
Musculoskeletal And Connective Tissue Disorders	198	(6.2)	245	(5.3, 7.0)
Arthralgia	116	(3.6)	141	(3.0, 4.3)
Gastrointestinal Disorders	190	(5.9)	231	(5.1, 6.8)
Vomiting	38	(1.2)	41	(0.8, 1.6)
Nausea	36	(1.1)	39	(0.8, 1.5)
Diarrhoea	35	(1.1)	37	(0.8, 1.5)
General Disorders And Administration Site Conditions	173	(5.4)	208	(4.6, 6.2)
Fatigue	68	(2.1)	81	(1.6, 2.7)
Asthenia	48	(1.5)	50	(1.1, 2.0)
Vascular Disorders	159	(4.9)	187	(4.2, 5.7)
Hypertension	141	(4.4)	168	(3.7, 5.1)
Blood And Lymphatic System Disorders	128	(4.0)	154	(3.3, 4.7)
Anaemia	85	(2.6)	101	(2.1, 3.3)
Metabolism And Nutrition Disorders	123	(3.8)	140	(3.2, 4.5)
Hyponatraemia	32	(1.0)	32	(0.7, 1.4)

All Grade 3 and Grade 4 Adverse Events including Serious Adverse Events are included in summary statistics.
If a patient had multiple occurrences of an AE, the patient is presented only once in the patient count.
Table presents number and percentage of patients (n (%)) and number of events (E).
Percentages are based on the number of patients in the population.

1.5.3 <u>Deaths</u>

Across all studies, the majority of deaths were attributed to progressive disease.

1.5.3.1 Study NO25026

In the pivotal, Phase III Study NO25026, 81% of patients in the vemurafenib treatment group died with censoring at crossover. Of the patients in the dacarbazine treatment group, 58% died with censoring at crossover, and 240 patients (82%) died without censoring at crossover.

There were 44 patients (13%) and 17 patients (6%) who died within 28 days of their last dose of vemurafenib and dacarbazine, respectively. The majority of the patients died due to disease progression (255 patients [76%] and 163 patients [56%] in the vemurafenib and dacarbazine treatment groups, respectively). A total of 70 patients in the dacarbazine treatment group who crossed over to vemurafenib died.

The incidence of AEs with an outcome of death (Grade 5 AEs) reported prior to crossover was similar in both treatment groups (6 patients [2%] in the vemurafenib and 4 patients [1%] in the dacarbazine treatment groups, respectively). In the vemurafenib treatment group, Grade 5 AEs included cerebrovascular accident, intracranial tumor hemorrhage, pneumonia, multiple injuries, completed suicide, and cardiac tamponade. All deaths except for one case (intracranial tumor hemorrhage) were reported as not related to study treatment.

In the dacarbazine treatment group, prior to crossover, Grade 5 AEs included dyspnea, shock, cardiac arrest, and cardiopulmonary failure. Only 1 death was considered to be related to dacarbazine (shock).
After crossover, an additional of 1 patient experienced an AE that led to death (pneumonia), which was reported as not related to study treatment.

1.5.3.2 Study NP22657

In Study NP22657, 99 (75.0%) patients died and 20 (15.2%) of these patients died within 28 days of their last vemurafenib dose. With the exception of 5 deaths (septic shock, unknown cause, death on study treatment, pneumonia, and acute renal failure within 28 days of last dose), all deaths resulted from disease progression.

Only 3 of the 5 deaths were reported as Grade 5 AEs. The reported events were pneumonia, acute renal failure, and cerebral hemorrhage, of which only the event of acute renail failure was considered related to the study drug by the investigator. In the event of the cerebral hemorrhage, according to the Investigator, cause of death was cerebral hemorrhage (underlying cause − disease progression).

1.5.3.3 Study NP25163

As of end of the Study NP25163, 25 of the 52 (48%) patients had died. Twenty-two (42%) died as a result of progressive malignant melanoma and 3 patients (6%) died of unknown cases (one was reported as ‘Unknown’ and two were reported as ‘Death).

1.5.3.4 Study MO25515

Table 33 Deaths (Safety Population)

	Vemurafenib (N=3219) n (%)	
Total Number of Deaths (n %)	2054	(63.8)
Primary Cause of Death		
Disease Progression	1932	(94.1)
Adverse Event *	85	(4.1)
Unknown	12	(0.6)
Other	25	(1.2)
Underlying Cause of Death		
Disease Progression	1853	(90.2)
Adverse Event	39	(1.9)
Unknown	124	(6.0)
Other	30	(1.5)
Not Applicable	8	(0.4)
Reasonable suspected relation between cause of death and study drug?		
Yes	25	(1.2)
No	2028	(98.7)
Unknown ~	1	(<0.1)

Table presents number and percentage of patients (n (%)).
Percentages for number of deaths are based on the number of patients in population. Other percentages are based on the number of deaths.
Survival data were only collected for patients who gave consent to collect these data.
* Three patients had Grade 5 AEs but the Primary Cause of Death was not recorded as AE:
Patient 2311860039: AE=Death, Primary Cause of Death=Other, Underlying Cause=Unknown;
Patient 2365810003: AE=Euthanasia, Primary Cause of Death=Other, Underlying Cause=Disease Progression;
Patient 2427490011: AE=Death, Primary Cause of Death=Other, Underlying Cause=Adverse event).
~ For Patient 2098010023 the reason was unknown.

1.5.4 <u>Serious Adverse Events</u>

Across all three studies (NO25026, NP22657, and NP25163), the most commonly reported vemurafenib-related SAE was cuSCC.

1.5.4.1 Study NO25026

SAEs were reported by 165 patients (49%) and 52 patients (18%) in the vemurafenib and dacarbazine treatment groups respectively. Of these, 119 patients (35%) and 17 patients (6%) in the vemurafenib and dacarbazine treatment groups, respectively, reported at least 1 SAE that was considered related to treatment.

Overall, 266 SAEs were reported in the vemurafenib treatment group, of which SCC of skin (20%); keratoacanthoma (11%); basal cell carcinoma (3%); malignant melanoma (2%); and pyrexia, pneumonia, and pulmonary embolism (1% each) were the most commonly reported. In the dacarbazine treatment group, 60 SAEs were reported, of which keratoacanthoma and pyrexia (1% each) were the most commonly reported.

1.5.4.2 Study NP22657

In NP22657, 79 (59.8%) patients experienced a total of 143 SAEs. Fifty-seven (43.2%) patients reported 88 SAEs that were considered by the investigator to be related to

vemurafenib treatment, most commonly cuSCC (34 patients, 25.8%). Other common (≥ 2%) vemurafenib-related SAEs included BCCs (7.6%), KA (3.8%), pyrexia (2.3%), and VIIth nerve paralysis (2.3%).

1.5.4.3 Study NP25163

In NP25163, 21 patients (40%) experienced one or more SAE, and 15 patients (29%) experienced one or more treatment-related SAE. The most common treatment-related SAE was cutaneous SCC in 7 patients (13%) and keratoacanthoma in 4 patients (8%).

1.5.4.4 Study MO25515

Table 35 Serious Adverse Events in ≥ 5 (≥ 0.2%) Patients (Safety Population)

System Organ Class<br>Preferred Term	Vemurafenib (N=3219)<br>n	(%)	E	95% CI
Number of patients with at least 1 SAE	1114	(34.6)	1923	(33.0, 36.3)
Neoplasms Benign, Malignant And Unspecified (Incl Cysts And Polyps)	572	(17.8)	917	(16.5, 19.1)
Keratoacanthoma	260	(8.1)	361	(7.2, 9.1)
Squamous Cell Carcinoma Of Skin	259	(8.0)	352	(7.1, 9.0)
Basal Cell Carcinoma	68	(2.1)	79	(1.6, 2.7)
Malignant Melanoma	43	(1.3)	46	(1.0, 1.8)
Malignant Melanoma In Situ	10	(0.3)	10	(0.1, 0.6)
Intracranial Tumour Haemorrhage	7	(0.2)	7	(0.1, 0.4)
Bowen's Disease	5	(0.2)	7	(0.1, 0.4)
Infections And Infestations	156	(4.8)	191	(4.1, 5.6)
Pneumonia	31	(1.0)	39	(0.7, 1.4)
Urinary Tract Infection	11	(0.3)	11	(0.2, 0.6)
Erysipelas	9	(0.3)	10	(0.1, 0.5)
Lower Respiratory Tract Infection	7	(0.2)	7	(0.1, 0.4)
Respiratory Tract Infection	7	(0.2)	7	(0.1, 0.4)
Sepsis	7	(0.2)	7	(0.1, 0.4)
Lung Infection	6	(0.2)	6	(0.1, 0.4)
Anal Abscess	5	(0.2)	6	(0.1, 0.4)
Cellulitis	5	(0.2)	6	(0.1, 0.4)
Groin Abscess	5	(0.2)	5	(0.1, 0.4)
Nervous System Disorders	107	(3.3)	118	(2.7, 4.0)
Cerebral Haemorrhage	14	(0.4)	14	(0.2, 0.7)
Seizure	12	(0.4)	13	(0.2, 0.7)
Epilepsy	10	(0.3)	14	(0.1, 0.6)
Cerebrovascular Accident	10	(0.3)	10	(0.1, 0.6)
Gastrointestinal Disorders	104	(3.2)	119	(2.6, 3.9)
Vomiting	17	(0.5)	18	(0.3, 0.8)
Diarrhoea	16	(0.5)	16	(0.3, 0.8)
Nausea	13	(0.4)	15	(0.2, 0.7)
Constipation	6	(0.2)	6	(0.1, 0.4)
Cardiac Disorders	80	(2.5)	91	(2.0, 3.1)
Atrial Fibrillation	15	(0.5)	15	(0.3, 0.8)
Acute Myocardial Infarction	10	(0.3)	10	(0.1, 0.6)
Myocardial Infarction	7	(0.2)	8	(0.1, 0.4)
Pericarditis	7	(0.2)	8	(0.1, 0.4)
Pericardial Effusion	7	(0.2)	7	(0.1, 0.4)
Cardiac Failure	6	(0.2)	6	(0.1, 0.4)
Angina Pectoris	5	(0.2)	5	(0.1, 0.4)
General Disorders And Administration Site Conditions	76	(2.4)	81	(1.9, 2.9)
Pyrexia	32	(1.0)	34	(0.7, 1.4)
General Physical Health Deterioration	9	(0.3)	9	(0.1, 0.5)
Death	7	(0.2)	7	(0.1, 0.4)
Fatigue	6	(0.2)	8	(0.1, 0.4)
Skin And Subcutaneous Tissue Disorders	59	(1.8)	63	(1.4, 2.4)
Actinic Keratosis	9	(0.3)	10	(0.1, 0.5)
Rash Maculo-Papular	7	(0.2)	7	(0.1, 0.4)
Photosensitivity Reaction	5	(0.2)	5	(0.1, 0.4)
Rash	5	(0.2)	5	(0.1, 0.4)
Respiratory, Thoracic And Mediastinal Disorders	45	(1.4)	47	(1.0, 1.9)
Pulmonary Embolism	13	(0.4)	13	(0.2, 0.7)
Pleural Effusion	6	(0.2)	6	(0.1, 0.4)

System Organ Class<br>Preferred Term	Vemurafenib (N=3219) n	(%)	E	95% CI
Musculoskeletal And Connective Tissue Disorders	37	(1.1)	42	(0.8, 1.6)
Arthralgia	12	(0.4)	13	(0.2, 0.7)
Back Pain	6	(0.2)	6	(0.1, 0.4)
Blood And Lymphatic System Disorders	33	(1.0)	37	(0.7, 1.4)
Anaemia	22	(0.7)	25	(0.4, 1.0)
Eye Disorders	24	(0.7)	26	(0.5, 1.1)
Uveitis	6	(0.2)	6	(0.1, 0.4)
Renal And Urinary Disorders	21	(0.7)	25	(0.4, 1.0)
Acute Kidney Injury	8	(0.2)	9	(0.1, 0.5)
Investigations	20	(0.6)	22	(0.4, 1.0)
Blood Creatinine Increased	7	(0.2)	7	(0.1, 0.4)
Psychiatric Disorders	20	(0.6)	20	(0.4, 1.0)
Confusional State	5	(0.2)	5	(0.1, 0.4)

If a patient has multiple occurrences of an AE, the patient is presented only once in the patient count.
Table presents number and percentage of patients (n (%)) and number of events (E).
Percentages are based on the number of patients in the population.
Exact binomial confidence intervals are presented based on the number of patients in the population.
Source: Adapted from Post-text Table 14.3.1.4, Section 11.5.1

1.5.5 Discontinuations due to Adverse Events

Across all studies, a small proportion of vemurafenib-treated patients experienced AEs that led to treatment discontinuation.

In the pivotal Phase III Study NO25026, AEs that led to study treatment discontinuation were reported by 25 patients (7%) and 5 patients (2%) in the vemurafenib and dacarbazine treatment groups, respectively. Overall, specific AEs leading to study treatment discontinuation occurred in ・ 3 patients in each treatment group. The most commonly reported AEs that led to study treatment discontinuation included arthralgia (3 patients) and dysphagia, increased blood bilirubin, and rash (2 patients) in the vemurafenib treatment group. No AE that led to study treatment discontinuation was reported in more than 1 patient in the dacarbazine treatment group. In Studies NP22657 and NP25163, 3% and 6% of patients had AEs that led to treatment discontinuation, respectively.

1.5.6 Adverse Events of Special Interest

AEs of special interest (AESIs) were prospectively defined based on safety signals identified during preclinical and early clinical studies or because they were reported as dose-limiting toxicities (arthralgia, fatigue, rash) during the dose escalation stage of the Phase I study (Study PLX06-02). AESIs included cuSCC, rash, photosensitivity, fatigue, arthralgia, liver function abnormalities, and events potentially related to QT interval prolongation. Each event was defined by medical concept and represented by a grouping of individual, related MedDRA preferred terms.

The event of QT interval prolongation was an AESI and defined as the group of preferred terms contained in the MedDRA Standard Medical Query (SMQ) of “torsades de pointes/QT prolongation”. There was a singular fatal case of torsades de pointes in

vemurafenib-treated patients in Study MO25515.

In Study NO25026, AEs of special interest were reported by 329 patients (98%) and 154 patients (54%) in the vemurafenib and dacarbazine treatment groups, respectively. AEs of cuSCC occurred in 93 patients (28%) and 5 patients (2%) in the vemurafenib and dacarbazine treatment groups, respectively. Most of the cuSCC events were SCC of skin and keratoacanthoma, all of which were serious and Grade 3 in intensity, as defined per protocol.

AEs of liver function abnormalities occurred in 94 patients (28%) and 17 patients (6%) in the vemurafenib and dacarbazine treatment groups, respectively. The most common liver function abnormality events reported in the vemurafenib and dacarbazine treatment groups were increased blood alkaline phosphatase and increased ALT, respectively. AEs of torsade de pointes/QT prolongation, which were potentially associated with prolongation of cardiac repolarization or arrhythmia, were reported by 13 patients (4%) and 1 patient (▪ 1%) in the vemurafenib and dacarbazine treatment groups, respectively.

The majority of occurrences of rash, arthralgia, fatigue, photosensitivity, liver function abnormalities and QT interval prolongation were of Grade 1 or Grade 2 intensity.

1.5.6.1 Cutaneous Squamous Cell Carcinoma (cuSCC)

In studies NO25026 and NP22657, cuSCC were reported as Grade 3 SAEs, as defined per protocol.

- In Study NO25026, ninty-three patients reported a total of 105 events of cuSCC, composed of cuSCC (66 events), keratoacanthoma (36 events), and Bowen's disease (3 events).
- In Study NP22657, 38 patients reported a total of 42 events of cuSCC composed of cuSCC (34 events, keratoacanthoma (7 events), and Bowen's disease (1 event).
- In Study NP25163, ten patients reported a total of 11 events of cuSCC composed of cuSCC (7 events), and keratoacanthoma (4 events).

The incidence of patients developing cuSCC was the same between the NO25026 (28%) and the NP22657 (28.8%), and similar to the NP25163 study (19%).

1.5.6.2 Other Neoplasms

1.5.6.2.1 Non-Cutaneous Squamous Cell Carcinoma

Rare cases of SCC of the head and neck have been reported in clinical trials where patients were treated with vemurafenib. One case in study NO25026 involved a patient who had a confirmed tonsilar SCC after receiving vemurafenib for greater than 200 days. The patient had a 30 pack-yr history of tobacco use. The patient's biopsy tested strongly

positive for p16 by immunohistochemistry, but no evidence of a RAS mutation or epidermal growth factor receptor (EGFR) amplification or mutation was present. A second case occurred in study NP25163 (PK/PD study). This patient had an invasive SCC of the tongue. He was previously treated for metastatic melanoma with ipilimumab, therapeutic vaccine (type not specified), and high dose interleukin -2, before enrolling on NP25163. The patient had no known risk factors for head-and-neck SCC and preliminary testing of the tumor was negative for the presence of HPV genome. Patients in clinical trials will undergo monitoring including head and neck examination (consisting of at least a visual inspection of oral mucosa and lymph node palpation), chest CT, and in relevant cases anal examinations and pelvic examinations (for women).

1.5.6.2.2 Adenomatous Colonic Polyps

Rare cases of adenomatous colonic polyps have been reported in patients treated with vemurafenib for two or more years while enrolled in a clinical trial. The severity and nature of this risk are currently unknown and under investigation . The clinical significance of colonic polyps is uncertain but physicians should be aware that they may occur in patients treated with vemurafenib.

1.5.6.3

1.5.6.4 Liver Injury

An analysis of liver-related adverse events reported with vemurafenib use showed that 63 cases from both clinical trials and postmarketing reports (out of an estimated 19,926 patients exposed) of medically confirmed serious adverse events were consistent with drug induced liver injury based on clinical chemistry criteria from the DILI Expert Working Group [126]. Of the 63 cases, two were assessed as severe, both reported as hepatic failure. There were no reported deaths among the 63 cases of liver injury; the outcome of both cases of severe liver injury is considered resolved. The median time to on set of the adverse events was 44 days after initial dose. The median ALT to ALP ratio was calculated as 1.5, suggesting a trend towards cholestatic pattern of liver injury. The analysis did not reveal any risk factors or populations at risk.

1.5.6.5 QT Prolongation

The effects of single and multiple doses of vemurafenib (960 mg bid) on ECG measurement, including the QT interval, were evaluated in 132 adult patients with metastatic melanoma in the Phase II study, NP22657. Centralized measurement of ECG intervals and T/U wave morphology was conducted by the core ECG laboratory on the robust schedule of serial time matched 12-lead ECGs obtained for up to 16 cycles. For each of the time points, the means from the available triplicate asse ssments were used as a single observation for each numeric ECG parameter. The T-wave and Uwave morphology and ECG normality were assessed on each triplicate ECG.

Vemurafenib treatment at 960 mg bid did not appear to have a clinically meaningful effect on heart rate (HR). The study population-specific correction (QTcP) eliminated

most of the bias from the QT-RR relationship and was therefore used for the primary statistical analyses of variables related to the QTc interval.

Forty-one patients (31.1%) exhibited new ECG changes considered to be abnormal and potentially significant. No patients developed new abnormal U waves, and 19 patients (14.4%) had new abnormal T-waves. Vemurafenib did not cause a meaningful change from the time-matched baseline ECG in either the QRS or the PR (PQ) interval. Two patients (1.5%) developed treatment-emergent QTcP values >500 msec (CTC Grade 3), while 49 (37.1%) and 6 (4.5%) of patients exhibited treatment-emergent, QTcP values > 450 msec and > 480 msec, respectively. No patients had treatment emergent uncorrected QT values > 500 msec. Maximal, treatment-emergent, individual QTcP changes >30 msec from baseline were observed in 58 (43.9%) patients, but only one patient (0.8%) exhibited a QTcP change >60 msec from baseline.

The pattern of increasing vemurafenib concentration from Days 1 to 15 of vemurafenib treatment appeared to correlate with the increased mean QTcP change observed from Days 1 to 15 and the constant vemurafenib exposures observed in later cycles appeared to correlate with the maintenance of the effect on QTc interval.

1.5.7 Safety on the Basis of BRAF V600 Mutation Status

1.5.7.1 Study NO25026

The safety-evaluable patient population for the BRAF V600 mutation status subgroup analyses comprised those patients who were treated and had at least one on-study assessment. On the basis of two safety clinical cutoff dates (30 December 2010 and 1 February 2012), the safety profiles of patient subgroups with BRAF V600 non-E mutations and/or V600K mutations within each treatment group were similar to the safety profiles for the overall safety populations within each respective treatment group (vemurafenib or DTIC) [117,124]. Data on safety by mutation status subgroups should be interpreted with caution because of the low numbers of patients in the non-V600E and/or V600K subgroups. In addition, there is no biological reason to suspect any differences in safety would be observed on the basis of the type of BRAF V600 mutation.

1.5.7.2 Study ML27763

In Study ML27763 (LPLV: 22 April 2015), the vemurafenib safety profile was consistent with the reported experience in melanoma. All patients had discontinued study treateament, primarily because of disease progression (n=20, 65%). The median duration of treatment with vemurafenib was 22.1 weeks. The safety population was defined as all enrolled patients who received any amount of vemurafenib. In this study, the ITT population is the same as the safety population. Overall, the safety and tolerability profile of vemurafenib observed in this study was similar across the mutation

subgroups and is consistent with what has been reported in published studies for patients with advanced BRAF V600E positive melanoma [118 - 120]. In the ITT population, 30 (97%) of the patients had at least on treatment-emergent AE; the most common AEs were fatigue (n=19, 61%), rash (n=13, 41.9%), arthralgia (n=12; 38.7%), and squamous cell carcinoma of the skin (10; 32.3%). These were also the most common AEs in each of the mutation subgroups. Twenty-eight patients (90%) experienced an AE that was considered related to study treatment; the most common of these tretatment-related AEs were fatigue (n=15; 48%), rash (n=12, 39%); arthralgia (n=11; 35%) and SCC of the skin (n=10; 32%). Grade 3, 4, and 5 AEs were reported by 16 (51.6%) patients, and 1 patient experienced a Grade 5 cerebrovascular accident, which was not considered related to vemurafenib. Eight (25.8%) patients required dose reduction because of AEs. One patient experienced cardiac failure, which resulted in the discontinuation of study drug. There were 9 (29%) deaths during this study: 4 patients died during treatment (2 each died from disease progression and AEs considered unrelated to study drug) and 5 patients died during the post-treatment followup period as a result of disease progression.

1.5.8 Safety in Patients with Brain Metastases

1.5.8.1 Study MO25653

The open-label study MO25653 (LPLV: 14 March 2012) evaluated the safety and tolerability of vemurafenib in patients with metastatic melanoma with brain metastases (n = 24) [121].

A total of 96% of patients (23/24) experienced at least 1 AE of any grade. One patient (Patient 207738001) did not experience any AEs, but died early in the study, on Day 4, due to disease progression. Adverse events most commonly reported in patients were in the SOCs of nervous system disorders (17/24; 71%), and skin and subcutaneous tissue disorders (16/24 patients; 67%). A total of 83% of patients (20/24) experienced at least 1 drug-related AE of any grade. Drug-related AEs most commonly reported in patients were in the SOCs of skin and subcutaneous tissue disorders (16/24 patients; 67%) and musculoskeletal and connective tissue disorders (9/24 patients; 38%). The majority of patients (22/24; 92%) had Grade 1/mild or Grade 2/moderate AEs. The most commonly reported AEs (≥ 5 patients) were arthralgia (9/24 patients; 38%) and epilepsy (6/24 patients; 25%); alopecia, solar dermatitis, rash maculopapular, dizziness, paraesthesia, vomiting, diarrhea and muscular weakness (each in 5/24 patients; 21%). A total of 4/24 patients (17%) experienced at least 1 Grade 3/severe AE, these included cSCC reported by all 4 patients (17%) and also AEs of amylase increased and GGT increased by 1 of these patients (4%). There were no Grade 4 adverse events reported during the study.

In the MO25653 study, a total of 23 SAEs were reported by 14/24 patients (60%). The most common SAEs were epilepsy (5/24 patients; 21%) and SCC of skin (4/24 patients; 17%). The SAE of ileus in 1 patient resulted in the discontinuation of study drug and death. This event was considered unrelated to the vemurafenib treatment. A total of 9 SAEs reported by 4/24 patients (17%) were considered related to vemurafenib treatment.

These were all events of SCC of the skin which occurred on treatment.

1.5.8.2 Study MO25743

In Study MO25743 (clinical cutoff: 16 April 2015), 141 patients (96.6%) in the SP experienced at least one AE: 88 (97.8%) in the previously untreated cohort and 53 (94.6%) in the previously treated cohort). The overall incidence of AEs reported among ≥20% of patients were arthralgia (37.0%), rash (32.2%), fatigue (28.1%), hyperkeratosis (28.1%), photosensitivity reaction (24.0%), and electrocardiogram-QT prolonged (20.5%). A total of 95 (65.1%) patients in the SP experienced at least one AE with severity Grades 3 or 4: including 59 [65.5%] in the previously untreated cohort and 36 [64.3%] in the previously treated cohort, regardless of relatedness to study drug. Per the protocol, all cases of cuSCC were reported as SAEs. Two AEs (cuSCC in 17 patients [11 (12.2%) in the previously untreated cohort and 6 (10.7%) in the previously treated cohort] and keratoacanthoma in 15 patients [11 (12.2%) in the previously untreated cohort and 4 (7.1%) in the previously treated cohort]) with severity Grades 3 or 4 were reported among ≥5% of patients in either cohort and were classified as study drug related.

A total of 6 patients (4.1%) had Grade 3/4 events of central nervous system hemorrhages, 3 (3.3%) in the previously untreated cohort and 3 (6.4%) in the previously treated cohort.

Overall, 3 patients (2.1%) were reported with an AE leading to death, with 2 patients (2.2%) in the previously untreated cohort and 1 patient (1.8%) in the previously treated cohort. Overall, 64 of 146 patients (43.8%) experienced at least one SAE, including 37 of 90 patients (41.1%) in the previously untreated cohort and 27 of 56 patients (48.2%) in the previously treated cohort. The most common SAEs were squamous cell carcinoma of skin (17 patients, 11.6%) and keratoacanthoma (15 patients, 10.3%). Ten AEs that led to treatment discontinuation were reported in 8 patients (5.5%), including 5 patients (5.6%) for the previously untreated cohort (7 AEs) and 3 patients (5.4%) in the previously treated cohort (3 AEs).

A total of 122 patients (83.6%) were reported with at least one AESI, including 76 (84.4%) in the previously untreated cohort and 46 (82.1%) in the previously treated cohort. A total of 333 AESIs were reported (202 in the previously untreated cohort and 131 in the previously treated cohort). The following AESIs were reported, rash (79

patients, 54.1%), AEs potentially associated with prolongation of cardiac repolarization or arrhythmia (30 patients, 20.5%), cuSCC (29 patients, 19.9%), liver function abnormalities (29 patients, 19.9%), photosensitivity (43 patients, 29.5%), other primary malignancy (6 patients, 4.1%), new primary melanoma (4 patients, 2.7%), and potentiation of radiation toxicity (1 patient, 1.1 %).

No cases of non-cuSCC were reported in this study. One patient (1.8%) in the previously treated cohort was reported with a Grade 3 unspecified SCC. Upon further review, it was noted that this lesion was a Grade 3 cuSCC of the right anterior thigh. Overall, treatment with vemurafenib was generally well-tolerated in patients with symptomatic and asymptomatic brain metastases. The safety profile was largely consistent with previous findings, and no new unexpected safety signals were identified from the review of these data.

1.5.9 Safety in Post-approval Studies

1.5.9.1 Global Safety Study MO25515

In analyses for the MO25515 post-approval safety study (clinical cutoff: February 2012), out of 5440 screened patients, 2398 (44%) were enrolled and 2207 had received at least one dose of vemurafenib [112]. Of 2207 patients, 1913 (87%) have reported AEs, 78% of which were related to vemurafenib, with 32% Grade 3 AEs and 2% Grade 4 AEs. The most common AEs (>10%) of any grade were arthralgia (31.2%), rash (26.2%), fatigue (20.7%), alopecia (16.9%), nausea (15.8%), photosensitivity reaction (11.3%), dry skin (11%), pruritus (10.6%), hyperkeratosis (10.5%) and headache (10%), and were similar irrespective of presence or absence of brain metastases and ECOG PS. The most common grade 3/4 AEs (≥1%) were rash (2.6%), arthralgia (2.5%), fatigue (1.5%), anaemia (1.0%) and cutaneous squamous cell carcinoma/ keratoacanthoma (4.7%/3.3%). Of 1282 pts (58%) who discontinued treatment, most withdrew due to progressive disease (42.8%) or death (6.7%) but 5.1% had withdrew due to AEs (most commonly general physical deterioration). AEs resulted in treatment interruption in 512 pts (23.2%).

Therefore, in a setting representative of routine clinical practice, vemurafenib is well tolerated for the treatment of BRAF V600-mutated metastatic melanoma, with a safety profile that resembles the safety profiles in the Phase I to III data, although this analysis is limited by the study duration.

1.5.9.2 Expanded Access Study ML25597

The primary objective of the ML25597 study was to provide vemurafenib to patients with BRAF V600E-positive metastatic melanoma who were otherwise without satisfactory treatment options (prior to marketing approval of vemurafenib). Safety of vemurafenib in patients with metastatic melanoma was a secondary objective. The most common

treatment-emergent AEs by system organ class included skin disorders (rash, photosensitivity reactions, etc.), musculoskeletal disorders (arthralgia, myalgia, etc.), general disorders (fatigue, pyrexia, etc.), and gastrointestinal disorders (nausea, diarrhea, etc.) [112]. In total, 308 patients reported 1353 treatment-emergent AEs related to vemurafenib that were primarily Grade 1 and 2, the most common of which were rash (all forms; 36.9%), arthralgia (34.8%), photosensitivity reaction (including sunburn;26.4%), fatigue (including asthenia, lethargy, and malaise; 19.9%), and nausea (10%),consistent with the previously reported safety profile of vemurafenib. Two Grade 5 treatment-emergent SAEs related to vemurafenib administration were reported, including one case each of multi-organ failure and cerebral hemorrhage. Seven Grade 4 treatment-emergent SAEs related to vemurafenib administration were reported, including one case each of respiratory failure, electrocardiogram (ECG) QT prolonged, neutropenia, hyperbilirubinemia, blood creatinine phosphokinase increased, neutrophil count decreased, and confusional state. Sixteen Grade 3 treatment-emergent SAEs related to vemurafenib administration were reported and nine patients experienced 10 AEs that led to vemurafenib withdrawal. Notably, the study contained a high proportion of heavily pre-treated patients, including those who received radiation for brain metastases, indicating a large fraction with highly progressed and intractable disease.

1.5.10 <u>Safety in Special Populations</u>

1.5.10.1 Study YO28390

Study YO28390 is a Phase I open label, multicenter, multiple dose study to investigate the pharmacokinetics, safety, and efficacy of vemurafenib in Chinese patients with BRAFv600 mutation positive unresectable or metastatic melanoma.
As of 15 December 2014, all 46 patients (100%) treated with vemurafenib reported a total of 719 AEs. The most common AEs occurring in ≥ 10% of patients were dermatitis acneiform and arthralgia (30 patients each, 65.2%), followed by blood cholesterol increased and diarrhea (27 patients each, 58.7%), blood bilirubin increased (25 patients, 54.3%), melanocytic nevus (24 patients, 52.2%), alopecia (23 patients, 50.0%), palmar-plantarerythrodysesthesia syndrome (22 patients, 47.8%), photosensitivity reaction (17 patients, 37.0%), fatigue (14 patients, 30.4%), pyrexia (13 patients, 28.3%), rash maculo-papular (12 patients, 26.1%), gamma-glutamyltransferase (GGT) increased and proteinuria (11 patients each, 23.9%), and total bile acids increased, hypertriglyceridemia, and leukopenia (10 patients each, 21.7%).

Twenty patients died during the study: causes of death were disease progression (18 patients, 90%), AE (1 patient), and ‘Unknown’ (1 patient). As reported by the investigator, the death due to an AE was a case of unconfirmed progressive disease, and was instead reported as an unrelated AE even though it occurred outside the reporting period for AEs.

Two patients had 2 serious adverse events (Grade 3 chest discomfort [serious criteria: hospitalized], and Grade 3 uveitis [serious criteria: disabling]) within the protocol -defined AE reporting period. Both of these events were considered by the investigator to be related to vemurafenib. For the event of chest discomfort, treatment with vemurafenib was interrupted. For the event of uveitis, the dose of vemurafenib was reduced.
No patient had an AE that led to withdrawal of treatment. Thirteen patients (28.3%) had AEs that led to dose modification or interruption. Seven patients (15.2%) had 9 AEs that were ≥ Grade 3. The AEs were: GGT increased (Grade 4; not related), anemia (Grade 3; not related), lymphopenia (Grade 3; not related), blood alkaline phosphatase increased (Grade 3; not related), hypokalemia (Grade 3; related), chest discomfort (Grade 3; related), blood cholesterol increased (Grade 3; related), uveitis (Grade 3; related), and anemia (Grade 3; related).

Vemurafenib was well tolerated in this study. A higher incidence of mild to moderate grade clinical AEs of blood cholesterol increased, hypertriglyceridemia, blood bilirubin increased, total bile acids increased, hyperuricemia, leukopenia, proteinuria, and melanocytic nevus was observed in this study compared with global pivotal Study NO25026. No cuSCC, keratoacanthoma, or Bowen’s disease has been reported in this study.

1.5.10.2 Study JO28178

Study JO28178 is a multi-center, open-label study evaluating the initial safety as well as the safety, tolerability, efficacy, pharmacokinetic, and dose intensity of vemurafenib in Japanese patients with unresectable or recurrent melanoma with BRAFV600 mutations. Based on the final CSR (LPLV: I8 April 2015), in the safety population, a total of 161 AEs occurred among all 11 patients. AEs experienced by ≥4 patients were arthralgia (10 patients), alopecia (7 patients), myalgia (8 patients), rash, maculopapular rash, fatigue, and decreased appetite (5 patients each).

AEs classified as ≥Grade 3 occurred 6 times in 3 patients. The ≥Grade 3 AEs were liver disorder, multiforme erythema, decreased neutrophil count, decreased white blood cell count, uveitis, and maculopapular rash (1 patient each). A causal relationship with vemurafenib could not be ruled out for any of these AEs.
Five patients died during the study due to disease progression but no deaths were caused by AEs.

One SAE occurred in 1 patient in the form of abnormal hepatic function, and a causal relationship with vemurafenib could not be ruled out.

AEs of interest caused by vemurafenib were as f ollows: AEs associated with hepatic function disorder (5 patients); photosensitivity (4 patients); arthralgia (10 patients); fatigue (7 patients); QTc Prologation (3 patients); hypersensitivity and severe cutaneous reactions (10 patients); and bone cell count and muscle spasticity (1 patient each).

No patient experienced the following AEs: cuSCC, non-cutaneous second primary malignancy, progression of RAS mutant malignancy, facial paralysis, drug-drug interactions, and GI polyp.

Abnormalities or abnormal changes in laboratory test values were as follows: decreased lymphocyte count (8 patients); decreased neutrophil count, increased ALT, and hematuria (4 patients each), decreased hematocrit, decreased hemoglobin, decreased white blood cell count, increased AST, increased GGT, increased cholesterol, increased triglycerides, and proteinuria (2 patients each), decreased platelet count, decreased red blood cell count, increased eosinophil count, increased ALP, decreased albumin, and decreased phosphorus (1 patient each). There were no clinically significant changes in vital signs.

1.5.10.3 Study NO25390

All 6 enrolled patients were treated with at least 1 dose of study drug, so all 6 patients were included in the safety population. No DLTs were observed in the first cohort of 3 patients treated at 720 mg BID. No DLTs were observed in the second cohort of 3 patients treated at 960 mg BID, which is equal to the adult recommended dose. An MTD could not be determined in this study because of the low number of patients enrolled. In lieu of establishing an MTD, Genentech has instead concluded, based on the limited data available, that the recommended dose of vemurafenib in adolescent patients should be expressed as an “upper limit”, or “not to exceed” daily dose, of 960 mg BID of vemurafenib.

All 6 patients enrolled in the study had at least 1 AE. Common AEs (those that were reported in □ 3 patients [□ 50%] overall) were: diarrhea and headache (4 patients each, 66.7%), followed by photosensitivity reaction, rash, nausea, and fatigue (3 patients each, 50.0%).

Three patients (50%) experienced an SAE during the study. One SAE was fatal (an event of intracranial tumor hemorrhage subsequent to study drug discontinuation for progressive disease confirmed by a brain computed tomography scan; in the investigator’s opinion the SAE was not related to study drug).

Two patients in Cohort 1 (66.7%) and 2 patients in Cohort 2 (66.7%) had at least 1 □ Grade 3 AE. Of the12 reported □ Grade 3 events, 10 were Grade 3, 1 was Grade 4 (scrotal abscess), and 1 was Grade 5 (intracranial tumor hemorrhage).
No patient had an AE that led to discontinuation of study drug, and no patient had an AE that led to a dose reduction of study drug. Two patients (33.3%) had an AE that led to study drug being temporarily withheld (a Grade 2 event of nephrolithiasis and a Grade 1 event of skin infection).

One patient (16.7%) had an AESI of cuSCC (Grade 3; related to study drug). This event was also reported as an SAE.

Five of the 6 patients (83.3%) died subsequent to disease progression. Four patient deaths (66.7%) were attributed to progression of disease, and 1 patient (16.7%) died due to an AE of intracranial tumor hemorrhage that, in the opinion of the Investigator,

was not related to study drug. One patient was alive at time of study termination by the Sponsor.

No new safety signals were observed in the 6 adolescents treated with vemurafe nib

1.5.11 Safety in the Post Marketing Setting

1.5.11.1 Drug Reaction with Eosinophilia and Systemic Symptoms (DRESS)

Eight cases of drug reaction with eosinophilia and systemic symptoms (DRESS) syndrome have been observed with vemurafenib treatment in the company's global safety database through March 2013, with four additional likely DRESS syndrome cases based on assessment of clinical manifestations. Of the eight diagnosed cases, four occurred within 15 days of vemurafenib treatment and four cases had no information on onset latency. Of the four likely DRESS cases, all occurred within 25 days of vemurafenib treatment. Of the twelve total cases, five were treated with steroids. No cases were reported to result in death. The majority (seven) patients had discontinued from vemurafenib treatment. Of the twelve cases, seven reported resolution. In addition, an abstract on hypersensitivity skin reactions in patients treated with vemurafenib after ipilimumab at the American Society of Clinical Oncology (ASCO) meeting in 2012 noted that the biopsies of two patients with Grade 3 rash revealed pathology that was consistent with a drug hypersensitivity reaction.

1.5.11.2 Progression of Existing Malignancy

In late 2012, Callahan et al. published an article in the New England Journal of Medicine that summarized the case of a patient who developed progression of pre-existing NRAS-mutated chronic myelomonocytic leukemia (CMML) shortly after the initiation of vemurafenib therapy for metastatic BRAF-mutant melanoma [127]. The patient, a 76 year old male, had no concurrent conditions, medical history, or concomitant medications reported. The patient had leukocytosis of 18,100 cells /mm3 with monocytosis of 3,000 cells/mm3 prior to treatment, which, at the time, was attributed to pneumonia. The patient was initially started on ipilimumab; however his melanoma progressed within 12 weeks, and thereafter the patient commenced treatment with vemurafenib. At the time vemurafenib was initiated, his white-cell count (WBC) was 25,600 cells/mm3. Eleven days after the first dose of vemurafenib, laboratory results showed marked leukocytosis of 80,900/mm3 with 27,600 monocytic cells/mm3 and vemurafenib treatment was stopped. Two weeks after vemurafenib cessation the patient's leukocyte counts dropped to 26,900/mm3. The patient was rechallenged with vemurafenib several times, each time resulting in elevated leukocyte counts, followed by a decrease in counts several days after cessation of the drug. Sanger sequencing of the nucleated cells in whole bone marrow and peripheral blood mononuclear cells (obtained prior to vemurafenib therapy) revealed an NRAS mutant leukemic clone. In vitro studies demonstrated proliferation of the leukemic cell population, an effect that was reversed

upon drug withdrawal. Further, the cells exhibited dose-dependent and reversible activation of ERK in the NRAS-mutated leukemic clone. Treatment with vemurafenib caused regression of the patient's BRAF V600K–mutant melanoma.

Another case of progression of pre-existing RAS-mutated malignancy was reported with vemurafenib use in 2014. This is a case report from the literature [136] about a 44 year-old male patient receiving vemurafenib for malignant melanoma who had a 2.6 cm pancreatic mass in the uncinate process of the pancreas with lymphadenopathy and biliary obstruction, prior to the administration of vemurafenib. Two weeks after the start of vemurafenib therapy, an abdominal PET CT demonstrated decrease in size of lymph nodes suggestive of a response to vemurafenib, but increase in the size and signal intensity of the pancreatic mass to 6 cm. FNA of the mass revealed malignant cells consistent with pancreatic adenocarcinoma, and vemurafenib was discontinued while the patient was started on capecitabine-based chemoradiotherapy. Following 4 weeks of combined chemoradiotherapy, CT scan revealed development of multiple hepatic lesions suspicious for metastases. Percutaneous liver biopsy demonstrated metastatic pancreatic adenocarcinoma, which was negative for BRAF mutation but positive for KRAS mutation. The patient was switched to gemcitabine and nab-paclitaxel treatment but died 7 months after he presented with scan findings of infiltrative mass in the pancreatic uncinate process. The authors concluded the event as related to vemurafenib.

Based on mechanism of action, vemurafenib may cause progression of cancers associated with RAS mutations through paradoxical activation of the MAPK pathway.

1.5.11.3 Neutropenia

A review of the Roche safety database in September 2013 found neutropenia to be an uncommon (6 cases per 1000 person-years, 0.6%) adverse drug reaction associated with the use of vemurafenib, typically occurring during the first 6-12 weeks of treatment. It appeared to be reversible usually within 2 weeks, with either temporary interruption, dose reduction or discontinuation, and in some cases was managed with GCSF.

1.5.11.4 Panniculitis

Twenty-six cases of medically confirmed panniculitis cases, out of an estimated 14,926 vemurafenib patients, have been reported in the company's global safety database until June 2013; 85% of the cases were assessed as causally associated with vemurafenib treatment. The majority of the cases are in females, and in most cases the latency is 10 to 20 days after the initial dose.

1.5.11.5 Pancreatitis

A review of the Roche safety database in December 2013 concluded that pancreatitis is an adverse drug reaction (ADR) in patients treated with vemurafenib, with cases originating from different tumor types (metastatic malignant melanoma, hairy cell

leukemia) and different reporting sources (spontaneous, literature, and clinical trials). There were no strong risk factors or alternative explanations to account for the occurrence of pancreatitis in 17 cases retrieved from the global safety database. In 13/17 cases (76%), a consistent latency of one month was seen which, according to Badalov et al. [128] categorized the drug as Class II in the drug-induced pancreatitis categorization based on the level of evidence (consistent latency in 75% or more of the reported cases). In addition, 8 of these 17 cases were assessed as more likely associated with vemurafenib, as all cases except one occurred within a week after vemurafenib therapy and all cases demonstrated positive de-challenge. The presentation (mild to moderate) of these cases in terms of severity was also consistent with the clinical picture of drug-induced pancreatitis as described in the scientific literature [129]. Finally, a positive re-challenge (although poorly documented) was reported in 2 out of these 8 cases.

1.5.11.6 Risk of Radiation Recall and Radiation Sensitization

A review of the Roche safety database of radiation-related adverse events reported with vemurafenib use in June 2014 concluded that potentiation of radiation treatment toxicity constitutes an adverse drug reaction for vemurafenib. This conclusion is based on 20 cases of radiation-related injuries adjudicated as radiation recall (n=8) and radiation sensitization (n=12 cases). The nature and severity of the events in all 20 cases were evaluated as worse than expected for the normal tissue tolerace to therapeutic radiation [133-135]. Most cases were cutaneous in nature but some cases involving visceral organs had fatal outcomes. In addition, the incidence of radiation-related injuries seen in the vemurafenib Phase III and Phase IV clinical trials was relatively higher at 5.2% and 6 % respectively (CI 1.71-11.74, 3.14 – 10.25) than the background rate among melanoma patients receiving radiation treatment (2.7%, CI 2.21 – 3.39).

Radiation Recall:
The cases of radiation recall showed acute inflammation confined to the previously irradiated area, triggered by vemurafenib administration ≥ 7 days after completion of radiotherapy. Five of the 8 cases (62%) affected the skin while the remaining cases involved the lung (n=2), and urinary bladder (n=1). The cutaneous reactions included: erythema, hyperkeratosis, eczematous, vesicular, and ulcerative lesions.
The mean time interval between the end of radiotherapy to the start of vemurafenib treatment was 28 days for cutaneous reactions (range 21- 42) while for non-cutaneous recall reactions the interval was 26-28 days for lung and 1460 days for the urinary bladder. The mean time to onset of radiation recall skin reaction after vemurafenib initial dose is 12 days (range 7-21 d), 24 days for pneumonitis, and 1 day for cystitis.
In all cases, patients were dosed with 960 mg BID of vemurafenib, while the cumulative radiation dose for the skin cases ranged from 20-71 Gy (mean 38.2 Gy, median 35 Gy) with radiation fraction size ranging from 1.8 to 4 Gy (mean 2.9 Gy, median 3 Gy). For the non-cutaneous cases of radiation recall, the cumulative radiation dose for the 2 lung cases was similar at 50 Gy (fraction size of 2 Gy) and for the urinary bladder case the

cumulative radiation dose was 66 Gy (fraction size not reported). Treatment with vemurafenib was maintained in 6 out of 8 cases, of which 5 cases received steroid treatment (topical/systemic); and in all 6 cases, the outcome was reported as resolved. In one case, vemurafenib was discontinued and the patient was treated with topical steroid and the event improved; the dose was reduced in the remaining case and the event persisted but did not worsen.

Radiation Sensitization:
The radiation sensitization cases showed potentiation of radiation reaction evidenced by the severity of the reaction, which was greater than what is expected for local radiation injury. Of the 12 cases, 9 events involved the skin, 3 events involved the esophagus, and one event each involved the liver and rectum. The nature of skin radiation sensitization is similar to that seen in radiation recall skin reactions. Except for one case, all cases were either concurrently/simultaneously on radiation and vemurafenib treatments or were started on vemurafenib within 3 days after completing radiotherapy. When reported, the time to onset of the reaction following initiation of radiation therapy or vemurafenib treatment ranged from 3 to 27 days (mean=10 d, median=8.5 d).

All patients with cases of radiation sensitization were dosed with 960 mg BID vemurafenib and the cumulative radiation dose received among cases involving the skin ranged from 9 to 36 Gy (mean=22.8 Gy, median=21 Gy), with radiation fraction size ranging from 3 to 6 Gy (mean=3.75 Gy, median=3 Gy). The cumulative radiation dose for cases of esophagitis ranged from 20-30 Gy with fraction size unreported in 2 cases and 4 Gy in one case. The cumulative radiation dose for proctitis was 30 Gy and 20 Gy for the liver radiation sensitization case (fraction size of 4 Gy). Vemurafenib was maintained at the same dose in 5 cases. Of these 5, 2 cases were treated with topical steroid and 2 cases had premature termination of radiotherapy; all 5 cases had a resolved or improved outcome. There was one case where vemurafenib was discontinued and patient was treated with steroid, which also resulted in resolution of the event. In one case, vemurafenib was temporarily interrupted but the event did not resolve. There was no information on vemurafenib treatment decision with corresponding event outcome for the rest of the cases.

There were 3 cases with fatal outcome: 1 case of a 15 year old female who devel oped radiation necrosis of the liver 10 weeks after receiving 20 Gy of fractionated radiation (fraction size 4 Gy) over the thoracic spine while on vemurafenib, and 2 cases of radiation esophagitis on adult patients, one of whom was reported to have worse ning of a Grade 1 esophagitis to Grade 4, 10 days after she was started on vemurafenib. The other case of fatal esophagitis had limited information other than that the patient was receiving concurrent vemurafenib and radiotherapy and reported radiation injury with a fatal outcome.

1.5.11.7 Acute Kidney Injury (AKI)

A review of the Roche safety database (with an estimated exposure to vemurafenib of 28,809 patients) in May 2015 concluded that acute kidney injury is an adverse drug reaction (ADR) in patients treated with vemurafenib. This was based on 145 AKI cases with some evidence of vemurafenib causality, of which 102 cases were assessed to have strong evidence. The cases ranged within a broad spectrum, majority were mild (> 1-1.5 x ULN) to moderate (> 1.5 – 3 x ULN) creatinine elevations, that appear to be reversible in nature. There were 3 cases of biopsy-proven interstitial nephritis, 4 cases of biopsy-proven tubular injury/acute tubular necrosis (ATN), and 20 cases presented with DRESS or DRESS-like symptoms. In the NO25026 study, the incidence of AKI cases in vemurafenib-treated patients was 10% compared to the dacarbazine arm, at 1.4%. The incidence of serum creatinine elevation during the course of study follow-up was also disproportionally higher in the vemurafenib arm, 40% compared to 6% in the control arm.

Association of AKI cases with male gender, hypertension, diabetes, chronic kidney disease, and at-risk concomitant medications such as diuretics, ACE-I, and NSAIDs was seen both in the clinical and in the postmarketing settings.

1.5.11.8 Dupuytren’s Contracture and Plantar Fibromatosis

A review of the Roche safety database (with an estimated exposure to vemurafenib of 26,560 patient-years) in September 2016 concluded that Dupuytren’s contracture and plantar fibromatosis are ADRs in patients treated with vemurafenib. This was based on 11 cases of Dupuytren’s contracture and 4 cases of plantar fibromatosis, which were assessed to be likely related to vemurafenib. Cases have been described as serious in 5 out of 11 Dupuytren’s cases whereby 1 case was described as disabling

1.5.11.9 Safety in Patients with Papillary Thyroid Cancer

The Phase II study NO25530 was conducted in n = 51 patients with BRAF V600 mutation positive papillary thyroid cancer whose tumors were refractory to radioactive iodine treatment. A total of 26 patients were enrolled in Cohort 1 (TKI naïve) and 25 patients were enrolled in Cohort 2 (TKI pretreated). Based on the final CSR (LPLV: 29 May 2015), all patientsin both cohorts (Cohort 1 = 26 patients; Cohort 2 = 25 patients) had at least 1 AE with a total of 939 AEs and 724 AEs reported in patients from Cohorts 1 and 2, respectively (multiple occurrences of the same AE were counted separately). Sixteen patients (61.5%) in Cohort 1 and 18 patients (72.0%) in Cohort 2 had at least 1 SAE. The most common AE in Cohort 1 was fatigue (18 patients, 69%), followed by weight decreased, alopecia, and dysgeusia (14 patients each, 54%); decreased appetite, skin papilloma, nausea, and blood creatinine increased (13 patients each, 50%); arthralgia and diarrhea (12 patients each, 46%); and photosensitivity (sunburn), rash, and hyperkeratosis (11 patients each, 42%).

The most common AEs in Cohort 2 were fatigue and weight decreased (14 patients each, 56%), followed by anemia (13 patients, 52%), decreased appetite (11 patients,

44%), and photosensitivity (sunburn) and blood bilirubin increased (10 patients each, 40%). The higher incidence of anemia in Cohort 2 patients was thought to be due to prior therapy with sorafenib or other agents.

Most of the reported AEs were Grade 1 or Grade 2 in intensity. Of the 939 reported AEs in Cohort 1, 64 AEs (6.8%) were ≥ Grade 3 in intensity. Of the 724 reported AEs in Cohort 2, 63 AEs (8.7%) were ≥ Grade 3 in intensity.

Eight patients (30.8%) in Cohort 1 and 16 patients (64.0%) in Cohort 2 died during the study. Two of the deaths (both in Cohort 2) were due to AEs that, in the opinion of the Investigators, were not related to treatment with vemurafenib: multi-organ failure (Day 12) and gastric adenocarcinoma (Day 512). The higher incidence in deaths on study observed in Cohort 2 (compared to Cohort 1) is likely attributable to more advanced disease.

In Cohort 1, 16 patients (61.5%) had at least 1 SAE during the study, of which at least 1 was a related SAE. In Cohort 2, 18 patients (72.0%) had at least 1 SAE during the study, and 11 of these patients (44.0%) had at least 1 related SAE.

In Cohort 1, 7 patients (26.9%) had at least 1 AE that led to withdrawal of treatment; in 6 of these patients (23.1%) the AE was related to treatment with vemurafenib, and in 4 of these patients (15.4%) the AE was serious. In the remaining 1 patient, the AE was unrelated to study treatment and was due to a pathological fracture that was considered serious. In Cohort 2, 6 patients (24.0%) had at least 1 AE that led to withdrawal of treatment; in 2 of these patients (8.0%) the AE was related to treatment with vemurafenib, and in 3 of these patients (12.0%) the AE was serious. In the remaining 4 patients, the AEs were unrelated to study treatment, and in 3 of these patients the AEs were considered serious. Three patients (11.5%) in Cohort 1 and 2 patients (8.0%) in Cohort 2 had an AESI. In Cohort 1 the AESIs were 1 event each of Grade 3 QTc prolongation, Grade 4 hepatotoxicity, and Grade 3 melanoma. In Cohort 2 the AESIs were events of Grade 2 malignant melanoma *in situ* and Grade 3 basal cell carcinoma. Overall, the vemurafenib safety profile in patients with PTC was found to be similar to the safety profile observed in other clinical trials. No new safety concerns were identified (Final CSR, RDR 1067698).

1.5.11.10 Safety in Patients with Metastatic CRC

The AE profile of vemurafenib in 21 patients with metastatic CRC treated in the extension phase of PLX06-02 is similar to that observed in patients with metastatic melanoma. All patients experienced at least one AE, the most common (reported in ≥30% of patients) of which were fatigue (57.1%), hyperglycemia (42.9%), arthralgia (38.1%), diarrhea (38.1%), hyperbilirubinemia (33.3%), nausea (33.3%), photosensitivity reaction (33.3%), rash (33.3%), and vomiting (33.3%).

Grade ≥ 3 study-drug-related AEs were reported in 61.9% of patients, the most common of which was cuSCC (23.8%). Seven patients (33.3%) experienced drug-related SAEs, the most common of which was cuSCC.

All deaths reported during treatment or within 28 days of last dose of study drug were attributed to disease progression.

Eleven (52.4%) patients had their study treatment interrupted and 4 (19.0%) had at least one dose reduction. AEs that led to dose interruption and/or modification for the CRC patients were similar to those reported for patients in the melanoma extension cohort. No CRC patients experienced an AE that led to vemurafenib treatment discontinuation.

1.6 SAFETY PROFILE: COBIMETINIB MONOTHERAPY

As of the data cutoff date of 11 June 2013, 115 patients were treated across all study stages in Study MEK4592g, including 74 patients treated with cobimetinib 60 mg 21/7

All patients in Study MEK4592g experienced an AE. The most frequent AEs were diarrhea (67.0%), fatigue (50.4%), rash (49.6%), nausea, vomiting (33 .9% each), and edema peripheral (28.7%). Other events that occurred in □ 10% of patients included anemia, abdominal pain, constipation, hypokalemia, decreased appetite, headache, dizziness, back pain, increased AST, dermatitis acneiform, pruritus, and dry skin.

Amongst the patients who received cobimetinib 60 mg 21/7, the most frequent treatment-emergent AEs in the cobimetinib 60 mg QD 21/7 group were diarrhea (64.4%), rash (53.3%), fatigue (48.9%), nausea, edema peripheral (31.1% each), and vomiting (28.9%).

Amongst all cobimetinib-treated patients, 5 patients (4.3%) experienced a Grade 4 AE, and 53 patients (46.1%) experienced a Grade 3 AE. The most frequent Grade 3 and Grade 4 AEs were hyponatremia (9.6%), fatigue (8.7%), anemia (7.8%), diarrhea, and hypokalemia (6.1% each). Grade 5 AEs, which in Study MEK4592g included disease progression reported as an adverse event, are discussed se parately below.

A total of 49 patients (42.6%) experienced an SAE. The most common types of SAE were GI disorders (n = 17), but there were no trends in specific preferred terms. The GI SAEs, such as intestinal obstructions and gastrointestinal hemorrhages, occurred in patients with gastrointestinal malignancies. SAEs reported for more than two patients among all patients in the study were anemia, bile duct obstruction, dehydration, syncope, and respiratory arrest (3 patients each [2.6%]).

As of the clinical-data cutoff date (20 September 2013), a total of 29 patients (25.2%) had died, including 11 patients in the cobimetinib 60 mg QD 21/7 group.

A total of 14 deaths were reported for patients treated in Stage I of the study. With the exception of 1 patient who died of cardiopulmonary arrest secondary to PD, all Stage I deaths occurred because of PD and no death was considered by the investigator to be related to the study drug.

During Stages IA, II, and IIA of the study, 12 deaths were reported, all of which occurred □ 30 days after the last dose of study drug. Of these, 2 deaths were considered by the investigator to be possibly related to study drug. In both cases, the investigator

considered the metastatic cancer to be a contributing etiologic factor to the patient's death.

Three deaths were reported in Stage III of this study. None was assessed by the investigator as treatment related. Other contributing etiologic factors to the deaths included the patients' underlying diseases and malignant tumor progression

1.7 BEVACIZUMAB CLINICAL EXPERIENCE

Bevacizumab has been studied in a multitude of Phase I, II, and III clinical trials in more than 22,000 patients and in multiple tumor types. Approximately 1,720, 000 patients have been exposed to bevacizumab as a marketed product or in clinical trials. T he following discussion summarizes bevacizumab's safety profile and presents some of the efficacy results pertinent to this particular trial. Please refer to the bevacizumab Investigator Brochure for descriptions of all completed Phase I, II, and III trials reported to date.

In a large phase III study (AVF2107g) in patients with metastatic colorectal cancer, the addition of bevacizumab, a monoclonal antibody directed against vascular endothelial growth factor (VEGF), to irinotecan/5-fluorouracil/leucovorin (IFL) chemotherapy resulted in a clinically and statistically significant increase in duration of survival, with a hazard ratio of death of 0.66 ($p < 0.001$) and a median survival of 20.3 vs. 15.6 months. Similar increases were seen in progression-free survival (10.6 vs. 6.2 months; HR 0.54, $p < 0.001$), overall response rate (34.8% vs. 44.8%; $p = 0.004$) and duration of response (10.4 vs. 7.1 months; HR 0.62, $p = 0.001$) for the combination arm versus the chemotherapy only arm (bevacizumab Investigator Brochure, November 2012). Based on the survival advantage demonstrated in Study AVF2107g, bevacizumab was designated for priority review and was approved on 26 February 2004 in the United States for first-line treatment in combination with IV 5-FU▪based chemotherapy for subjects with metastatic colorectal cancer.

Bevacizumab has also been approved based on additional Phase III trials in metastatic CRC (E3200 and ML18147) non small cell lung cancer (NSCLC; E4599), and renal cell carcinoma (RCC; AVOREN) which also demonstrated clinical benefit from bevacizumab. Furthermore, Phase II studies in glioblastoma (GBM; AVF3708g and NCI-06-C0064) showed an improvement in objective response rate. These studies led to accelerated approval by the FDA for recurrent GBM.

In Study E3200, the addition of bevacizumab to FOLFOX chemotherapy resulted in improved overall survival compared with FOLFOX alone (13.0 vs. 10.8 months, respectively, HR ▪ 0.75; p ▪ 0.01) in a population of previously treated, Avastin naive metastatic CRC patients. In Study ML18147, bevacizumab in combination with oxaliplatin- or irinotecan-based chemotherapy regimens demonstrated a statistically

significant increase in OS compared to oxaliplatin- or irinotecan-based chemotherapy alone (11.2 vs. 9.8 months, respectively, HR = 0.81; p=0.0062) in metastatic CRC patients who had previously received bevacizumab as a part of their 1st line treatment (Bennouna et al. 2013). These two studies led to FDA approvals for bevacizumab for previously treated metastatic CRC patients, in 2006 and 2013, respectively.

There was also improved overall survival in first-line NSCLC patients (E4599) treated with carboplatin/paclitaxel ▪ bevacizumab compared with chemotherapy alone (12.3 vs. 10.3 months, respectively; HR ▪ 0.80; p = 0.003). The results from this trial were the basis for FDA approval of bevacizumab for use in combination with carboplatin ▪ paclitaxel as first-line treatment of patients with unresectable, locally advanced, recurrent or metastatic, non-squamous NSCLC in October 2006.

In previously untreated metastatic RCC patients, bevacizumab in combination with interferon-alfa showed an improved progression free survival compared to interferon-alfa alone (10.2 vs. 5.4 months, respectively; HR=0.63; p=0.0001). These results supported the FDA approval of bevacizumab with interferon-alfa in metastatic RCC in July 2009.

Two Phase II trials investigated bevacizumab as a single agent in patients with recurrent GBM. In AVF3708g, patients with recurrent GBM were randomize d to bevacizumab or bevacizumab plus irinotecan and demonstrated an improvement in objective response rate (28.2% vs. 37.8%, respectively). The NCI-06-C0064 study was single arm Phase II study in recurrent GBM patients treated with bevacizumab alone and showed an objective response rate of 19.6%. This study supported the results from AVF3708g, and based on the objective response rate in these two trials, the FDA granted accelerated approval for bevacizumab as a single agent in GBM patients with progressiv e disease following prior therapy. In 2013, results from two phase III randomized controlled trials for newly diagnosed GBM were presented, one Roche-sponsored trial (AVAglio) and one cooperative group trial (RTOG 0825). In AVAglio, progression free survi val was significantly longer with bevacizumab when added to radiation therapy/temozolomide (HR 0.64, mPFS 10.6 vs 6.2 months). Health-related quality of life (HRQoL) and Karnofsky performance score (KPS) were stable/improved during PFS (both arms). Patients receiving bevacizumab plus radiation therapy/temozolomide had diminished corticosteroid requirement, but reported more adverse events (AEs) compared with placebo plus radiation therapy/temozolomide (serious AEs: 36.6% vs 25.7%; grade ≥3: 62.7% vs 50.1%; grade ≥3 AEs of special interest to bevacizumab: 28.7% vs 15.2%). In RTOG 0825, PFS was extended for bevacizumab (7.3 vs.10.7 months, HR 0.79) but did not meet the prespecified endpoint for significance. There was no difference between arms for overall survival (median 16.1 vs.15.7 months, HR 1.13).

Lastly, in the E2100 study, patients with untreated metastatic breast cancer who received bevacizumab in combination with weekly paclitaxel had a marked improvement in PFS compared with chemotherapy alone (13.3 vs. 6.7 months, respectively, HR 0.48; p<0.0001) and this led to the accelerated approval of bevacizumab in metastatic breast

cancer. Unfortunately, the clinical benefit was not confirmed in subsequent trials and the FDA ultimately removed the label for the breast cancer indication. (See the Bevacizumab Investigator Brochure for additional details).

a. Safety Profile

Hypertension: An increased incidence of hypertension (all grades) of up to 42.1% has been observed in patients treated with bevacizumab compared to up to 14% in the comparator arm. In clinical trials across all indications the overall incidence of NCI-CTC Grade 3 and 4 hypertension in patients receiving bevacizumab ranged from 0.4% to 17.9%. Grade 4 hypertension (hypertensive crisis) occurred in up to 1.0% of bevacizumab-treated patients, compared to up to 0.2% of patients treated with the same chemotherapy alone. Very rare cases of hypertensive encephalopathy have been reported, some of which were fatal. The risk of bevacizumab -associated hypertension did not correlate with the patients' baseline characteristics, underlying disease or concomitant therapy.

Analyses of the clinical safety data suggest that the occurrence of hypertension with Avastin therapy is likely to be dose-dependent. Monitor blood pressure every two to three weeks during treatment with bevacizumab. Treat with appropriate anti-hypertensives such as angiotensin-converting enzyme inhibitors, diuretics and calcium-channel blockers and monitor blood pressure regularly. Continue to monitor blood pressure at regular intervals in patients with bevacizumab -induced or –exacerbated hypertension after discontinuation of bevacizumab.

Temporary interruption of bevacizumab therapy is recommended in patients with hypertension requiring medical therapy until adequate control is achieved. Avastin should be permanently discontinued if medically significant hypertension cannot be adequately controlled with antihypertensive therapy. Bevacizumab should be permanently discontinued in patients who develop hypertensive crisis or hypertensive encephalopathy.

Proteinuria: In clinical trials, proteinuria has been reported within the range of 0.7% to 38% of patients receiving bevacizumab. Proteinuria ranged in severity from clinically asymptomatic, transient, trace proteinuria to nephrotic syndrome. Grade 3 proteinuria was reported in up to 8.1% of treated patients. Grade 4 proteinuria (nephrotic syndrome) was seen in up to 1.4% of treated patients. In the event of Grade 4 proteinuria Avastin treatment should be permanently discontinued. The proteinuria seen in bevacizumab clinical trials was not associated with renal impairment and rarely required permanent discontinuation of bevacizumab therapy.

Analyses of the clinical safety data suggest that the occurrence of proteinuria with Avastin therapy is likely to be dose-dependent. Patients with a history of hypertension may be at increased risk for the development of proteinuria when treated with

bevacizumab. There is evidence suggesting that Grade 1 proteinuria may be related to bevacizumab dose. Testing for proteinuria is recommended prior to start of bevacizumab therapy. In most clinical studies urine protein levels of ≥2g/24h led to the holding of bevacizumab until recovery to <2g/24h.

Venous thromboembolism (including deep venous thrombosis, pulmonary embolism, and thrombophlebitis:Patients may be at risk of developing venous thromboembolic events, including pulmonary embolism under Avastin treatment. In clinical trials across all indications the overall incidence of VTE events was 2.8% –17.3% in the bevacizumab-containing arms compared with 3.2% 15.6% in the chemotherapy control arms. Venous thromboembolic events include deep venous thrombosis and pulmonary embolism.

Grade 3-5 venous thromboembolic events have been reported in up to 7.8% of patients treated with chemotherapy plus bevacizumab compared with up to 4.9 % in patients with chemotherapy alone. Patients who have experienced a venous thromboembolic event may be at higher risk for a recurrence if they receive bevacizumab in combination with chemotherapy versus chemotherapy alone.

From a clinical trial in patients with persistent, recurrent, or metastatic cervical cancer (study GOG-0240), grade 3-5 venous thromboembolic events have been reported in up to 10.6% of patients treated with chemotherapy and bevacizumab compared with up to 5.4% in patients with chemotherapy alone.

In clinical trial BO21990, Grade 3-5 venous thromboembolic events were observed in 7.6% of patients with newly diagnosed glioblastoma treated with Avastin in combination with chemotherapy and radiotherapy, compared to 8.0 % of patients treated with chemotherapy and radiotherapy alone.

Avastin should be discontinued in patients with life-threatening (Grade 4) venous thromboembolic events, including pulmonary embolism. Patients with thromboembolic events ≤Grade 3 need to be closely monitored.

Arterial Thromboembolism: An increased incidence of ATE events was observed in patients treated with bevacizumab across indications including cerebrovascular accidents, myocardial infarction, transient ischemic attacks, and other arterial thromboembolic events. Avastin should be permanently discontinued in patients who develop arterial thromboembolic events.

In clinical trials, the overall incidence ranged up to 5.9% in the bevacizumab - containing arms compared up to 1.7% in the chemotherapy control arms. Fatal outcome was reported in 0.8% of patients receiving bevacizumab in combination with chemotherapy compared to 0.5% of patients receiving chemotherapy alone. Cerebrovascular accidents (including transient ischemic attacks) were reported in up to 2.3% of be vacizumab

treated patients versus 0.5% of patients in the control group: myocardial infarction was reported in 1.4% of bevacizumab treated versus 0.7% of patients in the observed control group.

In one clinical trial, AVF2192g, patients with metastatic colorectal cancer who were not candidates for treatment with irinotecan were included. In this trial arterial thromboembolic events were observed in 11% (11/100) of bevacizumab patients compared to 5.8% (6/104) in the chemotherapy control group. In an uncontro lled clinical trial, AVF3708g, in patients with relapsed glioblastoma, arterial thromboembolic events were observed in 6.3% (5/79) of patients who received Avastin in combination with irinotecan compared to 4.8% (4/84) of patients who received Avastin alone.

Patients receiving Avastin plus chemotherapy with a history of arterial thromboembolism, diabetes or age greater than 65 years have an increased risk of developing arterial thromboembolic events during Avastin therapy. Caution should be taken when treating such patients with Avastin.

Aspirin is a standard therapy for primary and secondary prophylaxis of arterial thromboembolic events in patients at high risk of such events, and the use of aspirin ≤ 325 mg daily was allowed in the five randomized studies discussed above. Use of aspirin was assessed routinely as a baseline or concomitant medication in these trials, though safety analyses specifically regarding aspirin use were not preplanned. Due to the relatively small numbers of aspirin users and arterial thromboembolic events, retrospective analyses of the ability of aspirin to affect the risk of such events were inconclusive. However, similarly retrospective analyses suggested that the use of up to 325 mg of aspirin daily does not increase the risk of grade 1-2 or grade 3-4 bleeding events, and similar data with respect to metastatic colorectal cancer patients were presented at ASCO 2005 (Hambleton et al., 2005).

Gastrointestinal perforation: Bevacizumab has been associated with serious cases of gastrointestinal perforation. Gastrointestinal perforations have been reported in clinical trials with an incidence of <1% in patients with metastatic breast cancer or non-squamous NSCLC, up to 2% in metastatic renal cell cancer, newly diagnosed glioblastoma, or in patients with ovarian cancer receiving front-line treatment, and up to 2.7% (including gastrointestinal fistula and abscess) in patients with metastatic colorectal cancer. Cases of GI perforations have also been observed in patients with relapsed glioblastoma. Fatal outcome was reported in approximately a third of serious cases of gastrointestinal perforations, which represents between 0.2 -1% of all bevacizumab treated patients.

In Avastin clinical trials, gastrointestinal fistulae (all grade) have been reported with an incidence of up to 2% in patients with metastatic colorectal cancer and ovarian cancer, but were also reported less commonly in patients with other types of cancer.

From a clinical trial in patients with persistent, recurrent, or metastatic cervical cancer (study GOG-0240), GI perforations, including gastrointestinal fistulae and abscess (all grade) were reported in 10.1% of Avastin treated patients, all of whom had a history of prior pelvic radiation. Fatal outcome was reported in 0.9% of Avastin-treated patients. Most patients reported as having GI perforations in this study (15 out of 22) had GI-vaginal fistulae.

The presentation of these events varied in type and severity, ranging from free air seen on the plain abdominal X ray, which resolved without treatment, to intestinal perforation with abdominal abscess and fatal outcome. In some cases underlying intra-abdominal inflammation was present, either from gastric ulcer disease, tumor necrosis, diverticulitis or chemotherapy-associated colitis. A causal association of intra-abdominal inflammatory process and gastrointestinal perforation to bevacizumab has not been established.

Patients may be at increased risk for the development of gastrointestinal perforation and gallbladder perforation when treated with Avastin. Avastin should be permanently discontinued in patients who develop gastrointestinal perforation.

Fistula: Bevacizumab use has been associated with serious cases of fistulae including events resulting in death. From a clinical trial in patients with persistent, recurrent, or metastatic cervical cancer (study GOG-0240), 4.1% of bevacizumab-treated patients and 2.3% of control patients were reported to have had vaginal, vesical or female genital tract fistulae (all grade), some of which were GI vaginal fistulae. The overall rate of GI-vaginal fistulae (all grade), combining both those reported as GI perforations and those reported as fistulae and abscess (as stated above) was 8.2% in bevacizumab -treated patients and 0.9% in control patients. Uncommon (≥0.1% to <1%) reports of other types of fistulae that involve areas of the body other than the gastroint estinal tract (eg, bronchopleural, urogenital, biliary fistulae) were observed across various indications. Fistulae have also been reported in post-marketing experience. Events were reported at various time points during treatment ranging from one week to greater than 1 year from initiation of bevacizumab, with most events occurring within the first 6 months of therapy.

Permanently discontinue bevacizumab in patients with tracheoesophageal fistulae or any Grade 4 fistula. Limited information is available on the continued use of bevacizumab in patients with other fistulae. In cases of internal fistula not arising in the GI tract, discontinuation of bevacizumab should be considered.

Wound healing complications: Avastin may adversely affect the wound healing process. Serious wound healing complications with a fatal outcome have been reported.

Necrotising fasciitis including fatal cases, has rarely been reported in patients treated with Avastin; usually secondary to wound healing complications, gastrointestinal

perforation or fistula formation. Avastin therapy should be discontinued in patients who develop necrotising fasciitis, and appropriate treatment should be promptly initiated.

As bevacizumab may adversely impact wound healing, patients who had major surgery within the last 28 days prior to starting bevacizumab treatment were excluded from participation in Phase III trials.

Across mCRC clinical trials there was no increased risk of post-operative bleeding or wound healing complications observed in patients who underwent major surgery between 28-60 days prior to starting bevacizumab therapy. An increased incidence of post-operative bleeding or wound healing complications occurring within 60 days of major surgery was observed if the patient was being treated with bevacizumab at the time of surgery. The incidence varied between 10% (4/40) and 20% (3/15).

In locally recurrent and metastatic breast and ovarian cancer trials, Grade 3-5 wound healing complications were observed in up to 1.1% of patients receiving bevacizumab compared with up to 0.9 % of patients in the control arms.

In the study of patients with relapsed glioblastoma (study AVF3708g), the incidence of post-operative wound healing complications (craniotomy site wound dehiscence and cerebrospinal fluid leak) was 3.6% in patients treated with single - agent bevacizumab and 1.3% in patients treated with bevacizumab plus irinotecan.

In patients with newly diagnosed glioblastoma (study BO21990) the incidence of Grade 3-5 post-operative wound healing complications (including complications following craniotomy) was 3.3% when treated with Avastin in combination with chemotherapy and radiotherapy, compared with 1.6 % when treated with chemotherapy and radiotherapy alone.

Bevacizumab should not be initiated for at least 28 days following surgery and until the surgical wound is fully healed. In patients who experience wound healing complications during Avastin treatment, Avastin should be withheld until the wound is fully healed. Avastin therapy should be withheld for elective surgery. The appropriate interval between the last dose of bevacizumab and elective surgery is unknown; however, the half-life of bevacizumab is estimated to be 20 days. Suspend bevacizumab for at least 28 days prior to elective surgery. Do no administer bevacizumab until the wound is fully healed.

Hemorrhage: In clinical trials across all indications the overall incidence of NCI-CTC Grade 3-5 bleeding events ranged from 0.4% to 6.9% in bevacizumab -treated patients, compared to 0 to 4.5% of patients in the chemotherapy control group. The hemorrhagic events that have been observed in bevacizumab clinical studies were predominantly tumor- associated hemorrhage (see below) and minor mucocutaneous hemorrhage

(e.g., epistaxis). Avastin should be permanently discontinued in patients who experience Grade 3 or 4 bleeding during Avastin therapy.

Tumor-Associated Hemorrhage: Major or massive pulmonary hemorrhage or hemoptysis has been observed primarily in patients with NSCLC. Possible risk factors include squamous cell histology, treatment with anti-rheumatic/anti-inflammatory drugs, treatment with anticoagulants, prior radiotherapy, bevacizumab therapy, previous medical history of atherosclerosis, central tumor location and cavitation of tumors prior to or during therapy. The only variables that showed statistically significant correlations with bleeding were bevacizumab therapy and squamous cell histology. Patients with NSCLC of known squamous cell histology or mixed cell type with predominant squamous cell histology were excluded from subsequent studies, while patients with unknown tumor histology were included.

In patients with NSCLC excluding predominant squamous histology, all Grade events were seen with a frequency of up to 9% when treated with bevacizumab plus chemotherapy compared with 5% in the patients treated with chemotherapy alone. Grade 3-5 events have been observed in up to 2.3% of patients treated with bevacizumab plus chemotherapy as compared with <1% with chemotherapy alone. Major or massive pulmonary hemorrhage/hemoptysis can occur suddenly and up to two thirds of the serious pulmonary hemorrhages resulted in a fatal outcome.

Patients with non–small cell lung cancer treated with Avastin may be at risk for serious, and in some cases fatal, pulmonary haemorrhage/haemoptysis. Patients with recent pulmonary haemorrhage/ haemoptysis (>1/2 teaspoon red blood) should not be treated with Avastin.

Mucocutaneus Hemorrage: Across all bevacizumab clinical trials, mucocutaneous hemorrhage has been seen in 50% of patients treated with bevacizumab. These were most commonly NCI-CTC Grade 1 epistaxis that lasted less than 5 minutes, resolved without medical intervention and did not require any changes in bevacizumab treatment regimen. Clinical safety data suggest that the incidence of minor mucocutaneous hemorrhage (e.g. epistaxis) may be dose-dependent.

There have also been less common events of minor mucocutaneous hemorrhage in other locations, such as gingival bleeding and vaginal bleeding.

Posterior Reversible Encephalopathy Syndrome (PRES): PRES is a rare neurologic disorder that can present with the following signs and symptoms (among others): seizures, headache, altered mental status, visual disturbance, or cortical blindness, with or without associated hypertension. Brain imaging is mandatory to confirm the diagnosis of PRES. Two confirmed cases (0.8%) of PRES have been reported in one clinical study. Symptoms usually resolve or improve within days, although some patients have experienced neurologic sequelae.

In patients who develop PRES, treatment of specific symptoms, including control of hypertension, is recommended along with discontinuation of bevacizumab. The safety of reinitiating bevacizumab therapy in patients previously experiencing PRES is not known (Glusker et al. 2006; Ozcan et al. 2006).

Congestive heart failure: In clinical trials CHF was observed in all cancer indications studied to date, but predominantly in patients with metastatic breast cancer. In five Phase III studies (AVF2119g, E2100, BO17708, AVF3694g and AVF3693g) in patients with metastatic breast cancer, Grade ≥ 3 CHF was reported in up to 3.5% of patients treated with bevacizumab in combination with chemotherapy compared with up to 0.9% in the control arms. For patients in study AVF3694g who received anthracyclines concomitantly with bevacizumab, the incidences of Grade ≥ 3 CHF for the respective bevacizumab and control arms were similar to those in the other studies in mBC: 2.9% in the anthracycline+Bv arm and 0% in the anthracycline+placebo arm. In addition, in study AVF3694g the incidence of any grade CHF was similar between the anthracycline+Bv (6.2%) and the anthracycline+placebo arms (6.0%). Most patients who developed CHF during mBC trials showed improved symptoms and/or left ventricular function following appropriate medical therapy.

In most clinical trials of bevacizumab, patients with pre-existing CHF of NYHA II – IV were excluded, therefore, no information is available on the risk of CHF in this population.

Prior anthracyclines exposure and/or prior radiotherapy to the chest wall may be possible risk factors for the development of CHF. Caution should be exercised before initiating bevacizumab therapy in patients with these risk factors.

An increased incidence of CHF has been observed in a clinical trial of patients with diffuse large B-cell lymphoma (BO20603) when receiving bevacizumab with a cumulative doxorubicin dose greater than 300 mg/m2. This phase III clinical trial compared rituximab/cyclophosphamide/doxorubicin/vincristine/prednisone (R-CHOP) plus bevacizumab to R-CHOP without bevacizumab. While the incidence of CHF in both arms was above that previously observed for doxorubicin therapy the rate was higher in the R-CHOP plus bevacizumab arm.

Events consistent with congestive heart failure (CHF) were reported in clinical trials. The findings ranged from asymptomatic declines in left ventricular ejection fraction to symptomatic CHF, requiring treatment or hospitalisation.

Caution should be exercised when treating patients with clinically significant cardiovascular disease such as pre-existing coronary artery disease, or congestive heart failure with bevacizumab.Patients receiving concomitant anthracyclines or with prior exposure to anthracyclines should have a baseline MUGA scans or echocardiograms (ECHOs) with a normal LVEF.

Ovarian Failure/Fertility: The incidence of new cases of ovarian failure, defined as amenorrhoea lasting 3 or more months, FSH level ≥ 30mIU/ml and a negative serum β-HCG pregnancy test, has been evaluated. New cases of ovarian failure were reported more frequently in patients receiving bevacizumab. After discontinuation of bevacizumab treatment, ovarian function recovered in the majority of women. Long term effects of treatment with bevacizumab on fertility are unknown.

Neutropenia: Increased rates of severe neutropenia, febrile neutropenia, or infection with severe neutropenia (including some fatalities) have been observed in patients treated with some myelotoxic chemotherapy regimens plus Avastin in comparison to chemotherapy alone.

Hypersensitivity reactions, infusion reactions: Patients may be at risk of developing infusion / hypersensitivity reactions. Close observation of the patient during and following the administration of bevacizumab is recommended as expected for any infusion of a therapeutic humanized monoclonal antibody. If a reaction occurs, the inf usion should be discontinued and appropriate medical therapies should be administered. A systematic premedication is not warranted.

In some clinical trials anaphylactic and anaphylactoid-type reactions were reported more frequently in patients receiving Avastin in combination with chemotherapies than with chemotherapy alone. The incidence of these reactions in some clinical trials of Avastin is common (up to 5% in bevacizumab-treated patients).

Laboratory Abnormalities: Decreased neutrophil count, decreased white blood count and presence of urine protein may be associated with bevacizumab treatment. Across clinical trials, the following Grade 3 and 4 laboratory abnormalities were seen with an increased (≥2%) incidence in patients treated with bevacizumab compared to those in the control groups: hyperglycemia, decreased hemoglobin, hypokalemia, hyponatremia, decreased white blood cell count, increased PT (prothrombin time), normalized ratio.

Additional Adverse Events: See the bevacizumab Investigator Brochure for additional details regarding the safety experience with bevacizumab.

1.8 STUDY RATIONALE

There is a strong rationale for combining targeted therapy with immunotherapy in ATC. First, targeted therapies with BRAF/MEK inhibitors and potent antiangiogenics have efficacy in ATC. Responses and stabilization of disease, although longer than cytotoxic chemotherapy, are still relatively short and can be improved upon. Second, ATC appears to be a very good candidate for immunotherapy due to the high expression of PD-L1 and infiltration by inflammatory and immune cells. However, immunotherapy alone is unlikely to control a disease that is rapidly progressive and fatal because it may

take several months to take effect. Third, there are data implicating the MAPK sign aling in the regulation of immune microenvironment. Thus, we hypothesize that combining targeted therapies with immunotherapy will prolong overall survival in patients with ATC.

2. OBJECTIVES

2.1 PRIMARY

To determine if targeted therapy + atezolizumab (cohorts 1-3) will lead to improved overall survival (OS) in patients with anaplastic thyroid carcinoma (ATC).

2.2 SECONDARY

1. To evaluate the safety and efficacy (RECIST/irRC response rate, progression-free survival [PFS]) of targeted therapy + atezolizumab (cohorts 1-3) in ATC and PDTC.
2. To determine the OS in patients with PDTC treated with targeted therapy + atezolizumab (cohorts 1-3).
3. To determine the efficacy (RECIST/irRC response rate, progression-free survival [PFS]) and OS of ATC and PDTC patients treated with taxanes + atezolizumab (cohort 4)

2.3 EXPLORATORY

1. To evaluate changes in the tumor-associated and systemic immune system biomarkers in ATC and PDTC patients treated with immunotherapy (See Appendix 12 for translational study plans including details on biospecimen collection, processing and analyses,).

2. To report RAI uptake in patients who have a diagnostic whole body scan and therapeutic I-131 in cohort 2.

3. STUDY DESIGN

3.1 DESCRIPTION OF THE STUDY

This study is a non-randomized, open-label single institution study of the combination of different targeted agents with atezolizumab in ATC and PDTC. See figure 1 for schematic trial design. Patients will be assigned to cohorts based on themolecular profileof their tumor. Due to the rapid progression seen in patients with ATC, all patients that meet general study eligibility criteria will have the option to be treated with either weekly nab-paclitaxel (preferred) or paclitaxel until molecular testing results become available. When these results become available, patients will be restaged (if >15 days on taxane ch emotherapy), re-consented and then stratified to a treatment arm based on the somatic mutations found in their tumor (see Figure 1).

- Patients will start targeted agents after a 7 day wash-out period if treated with induction taxanes (if induction taxane treatment is given).
- Those with BRAF mutation will be screened for cohort 1. If eligible for cohort 1 vemurafenib + cobimetinib will be taken, for arun-inperiod of 28 days. If liver function tests (LFTs: AST, ALT, total bilirubin) are < grade 3 then patients will start atezolizumab in combination with vemurafenib + cobimetinib.

- Those with RAS, NF1, or NF2 mutations will be screened for cohort 2, cobimetinib in combination with atezolizumab. In addition patients with tumors with MAPK activating mutations at or above MEK will be considered for cohort 2, on a case-by-case basis, after discussion with medical monitor to discard the possibility of a non-activating mutation.
- Patients without BRAF,RAS, NF1, NF2, or other Cohort 2-qualifying mutations will be screened for cohort 3, bevacizumab + atezolizumab.
- Any patient who does not meet entry criteria for a particular targeted therapy cohort may be screened for any other of the remaining cohorts, preferably for targeted therapy cohorts per figure 1.
- Patients who are not eligible for any of the targeted therapy cohorts (cohorts 1-3) may be enrolled in cohort 4, nab-paclitaxel (or paclitaxel if nab-paclitaxel cannot be obtained) + atezolizumab.

Patients will be evaluated by an investigator prior to each dose, including history and physical examination, vital signs, and laboratory evaluation, including chemistries, liver function, complete blood count, and urinalysis. Radiographic evaluation for response assessment will occur every 8 weeks for cohorts 1 and 2, and every 6 weeks for cohorts 3 and 4. Treatment will continue until disease progression, unacceptable toxicity, or withdrawal of consent.

In the absence of unacceptable toxicity, patients who meet criteria for disease progression per RECIST v1.1 while receiving atezolizumab will be permitted to continue study treatment if they meet all of the following criteria:

- Evidence of clinical benefit, as determined by the investigator following a review of all available data
- Absence of symptoms and signs (including laboratory values, such as new or worsening hypercalcemia) indicating unequivocal progression of disease
- Absence of decline in ECOG Performance Status that can be attributed to disease progression
- Absence of tumor progression at critical anatomical sites (e.g., leptomeningeal disease) that cannot be managed by protocol-allowed medical interventions”

All patients will be followed until death or withdrawal of consent. We expect completion of the trial 18 months after the last patient is enrolled.

Sample size and anticipated duration:

	Targeted enrollment	Duration (months)
Anaplastic Thyroid Carcinoma	36-60	33
Poorly Differentiated Thyroid Carcinoma	0-14	33
Total	66	

The rate of accrual is expected to be 1.5 patients/month. A minimum of 10 patients must be enrolled in cohorts 1-3. In order to achieve needed accrual for the primary endpoint, no more than 14 patients will be accrued to cohort 4. Exploratory endpoint: Patients will undergo optional biopsy at screening, and mandatory biopsy at cycle 2, day 1 (-5 days), and at progression for evaluation of immune infiltration.

Patients will be allowed to undergo elective surgery and/or radiation to control their disease, throughout the duration of the trial. However, in patients who are on bevacizumab this drug will be held for 4 weeks prior to surgery. Atezolizumab does not need to be held unless otherwise required, in the opinion of the treating physician.

Neoadjuvant treatment with atezolizumab combinations prior to surgery +/- adjuvant radiation is an exploratory endpoint of this study. The standard of care for adjuvant radiation includes concurrent cytotoxic chemotherapy if tolerated by the patient. Thus, concurrent cytotoxic chemotherapy in this setting will be permitted. During adjuvant radiation +/- cytotoxic chemotherapy, all study drugs will be held, but may be resumed as soon as 2 weeks after completion of adjuvant radiation +/- cytotoxic chemotherapy.

Figure 1: Study Design

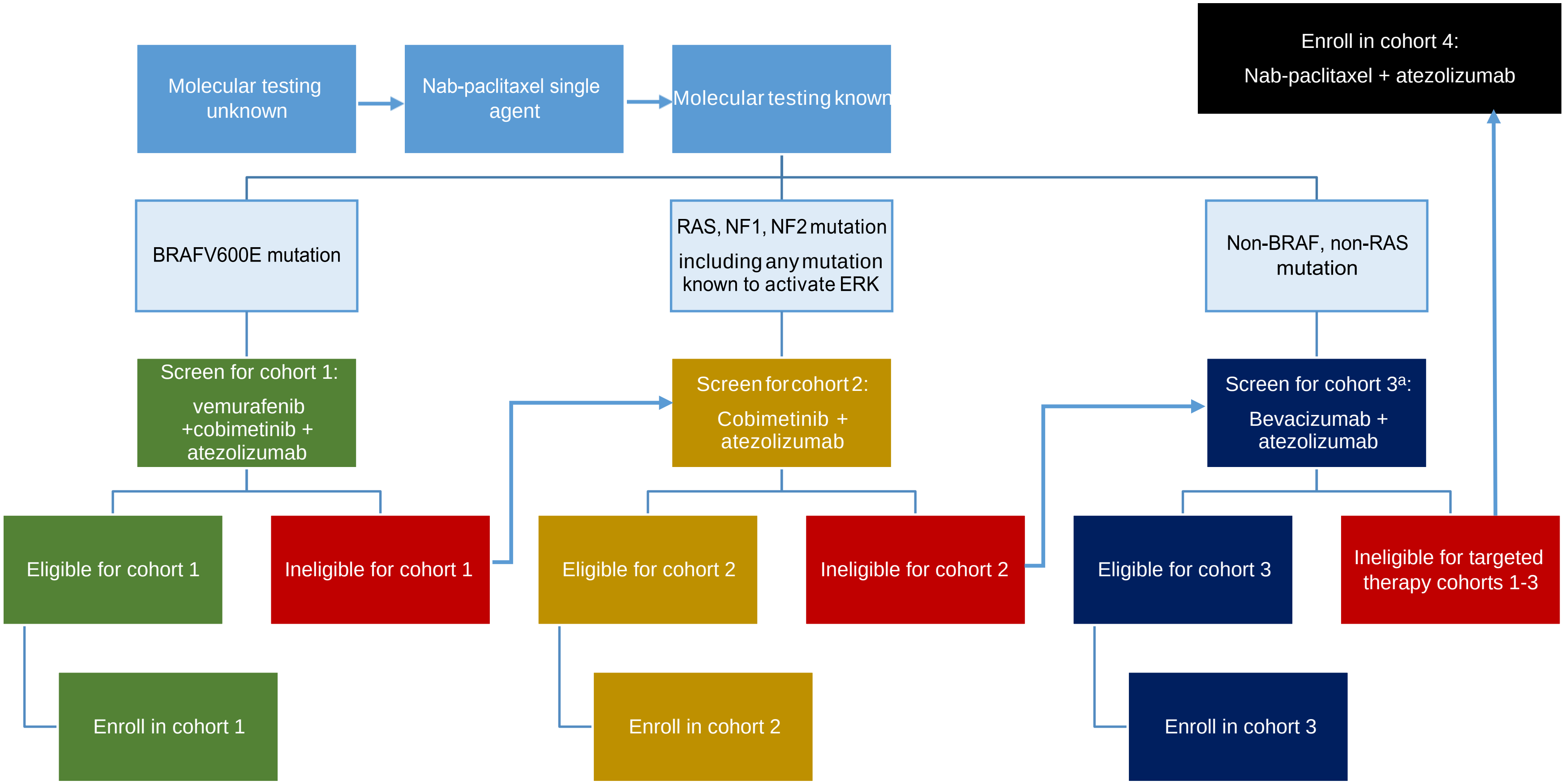

Figure 1. Treatment arm assignment based on molecular testing. Patients with unknown molecular testing status may be treated with nab-paclitaxel (paclitaxel is acceptable if nab-paclitaxel cannot be used) until molecular testing results are known. Patients will then be assigned a treatment cohort based on mutation profile. A wash-out period of 7 days is requiredafter treatment with taxanes. [a] Those patients unable to travel every 2 weeks for atezolizumab administration in cohorts 1 and 2 may be screened for cohort 3 consideration.

3.2 END OF STUDY

The end of this study is defined as the date when the last patient, last visit (LPLV) occurs or the date at which the last data point required for statistical analysis (i.e. all patients have died or withdrawn from the study) or safety follow-up is received from the last patient, whichever occurs later. LPLV is expected to occur 18 months after the last patient is enrolled.

3.3 RATIONALE FOR STUDY DESIGN

There is a critical need to identify new, more effective approaches to treating ATC. We believe that the current approach to ATC must be completely revised in the age of such promising, emerging targeted and immunotherapies. However, due to the highly aggressive nature of this disease, cytotoxic chemotherapy needs to be used as a bridge while molecular testing is being processed. While responses are reasonable with cytotoxic chemotherapy, they are short-lived (median OS=4 months and median PFS=3.1 months1). On the other hand, responses to targeted therapies (i.e., vemurafenib, dabrafenib+trametinib, and the antiangiogenic drug, lenvatinib) are more durable (PFS=7.4 months with lenvatinib) and quite promising. ATC and PDTC have florid immune infiltrates that highly express PD-L1. Due to the rapid growth of anaplastic tumors, single agent immunotherapy is not a feasible approach, as these patients need responses to occur within weeks. Our extensive experience with ATC leads us to hypothesize that an approach with genotyped-directed targeted agents in combination with immunotherapy will prolong survival.

3.4 OUTCOME MEASURES

3.4.1 <u>Primary Efficacy Outcome Measure</u>

To determine if targeted therapy + atezolizumab (cohorts 1-3) will lead to improved overall survival (OS) in patients with anaplastic thyroid carcinoma (ATC).

3.4.2 <u>Secondary Efficacy Outcome Measures</u>

1. To evaluate the efficacy (RECISTv1.1, immune modified RECIST (irRC) response rate, progression-free survival [PFS]) of targeted therapy + atezolizumab (cohorts 1-3) in ATC and PDTC.
2. To determine the OS in patients with PDTC treatedwith targeted therapy + atezolizumab (cohorts 1-3).
3. To determine the efficacy and OS of ATC and PDTC patients treated with taxanes + atezolizumab (cohort 4)
4. To evaluate the efficacy and OS of each targeted therapy cohort (1-3) separately and in each disease group (ATC and PDTC).

3.4.3 <u>Safety Outcome Measures</u>

1. To evaluate the safety of targeted therapy + atezolizumab in cohorts 1-3.

2. To evaluate the safety of taxanes + atezolizumab in cohort 4.

All patients who receive at least 1 dose of study drug (vemurafenib, cobimetinib, bevacizumab, atezolizumab) in any cohort will be eligible for safety outcome.

4. MATERIALS AND METHODS

4.1 STUDY POPULATION

4.1.1 Inclusion Criteria

1. Histologically confirmed anaplastic thyroid or poorly differentiated thyroid carcinomas.

2. Patients deemed to have unresectable locoregional disease or metastatic disease. Patients who are unwilling to undergo surgery or external beam radiation are also eligible.

3. Patients with PDTC must have at least one target lesion by RECIST v1.1. This is not a requirement for ATC patients.

4. Total bilirubin ≤1.5 x upper limit of normal (ULN). Total bilirubin≤ 3 x ULN for patients with Gilbert's syndrome. AST (SGOT)/ALT (SGPT) ≤2.5 x ULN, (5 x ULN for patients with concurrent liver metastases). Serum creatinine ≤ within 1.5 x ULN. ANC ≥ 1.0 x 10 ^9/L; PLT ≥ 100 x 10^9/L.

5. For patients receiving therapeutic anticoagulation: stable anticoagulant regimen and stable INR during the 28 days immediately preceding initiation of study treatment

6. Subjects must be willing to undergo tumor biopsy after treatment with atezolizumab, unless in the opinion of the treating physician, a biopsy is not feasible or safe.

7. ECOG PS</=2

8. Age >/= 18 years.

9. Age and Reproductive Status a) Males and Females, >/=18 years. Women of childbearing potential (WOCBP)* must have a negative serum or urine pregnancy test within 14 days prior to the start of study drug and must use effective contraceptives throughout the duration of the study. Males who are sexually active with WOCBP must agree to use effective contraception throughout the duration of the study. Azoospermic males and WOCBP who are continuously not heterosexually active are exempt from contraceptive requirements.

*A Women of childbearing potential (WOCBP) is defined as any female who has experienced menarche and who has not undergone surgical sterilization (hysterectomy or bilateral oophorectomy) and is not postmenopausal. Menopause is defined as 12 months of amenorrhea in a woman over age 45 years in the absence of other biological or physiological causes.

10. Negative hepatitis B surface antigen (HBsAg) test at screening

11. Ability to provide informed consent.

12. **ADDITIONAL INCLUSION CRITERIA FOR BRAF MUTATION (COHORT 1):** Patients with a BRAFV600E mutation being considered for the triplet combination (vemurafenib + cobimetinib + atezolizumab) must meet the following endorgan function criteria: ANC ≥ 1.5 × 10^9/L without granulocyte colony-stimulating factor support, WBC count ≥ 2.5 × 10^9/L, Lymphocyte count ≥ 0.5 × 10^9/L, Platelet count ≥ 100 × 10^9/L without transfusion, Hemoglobin ≥ 9.0 g/L without transfusion. Serum albumin ≥ 2.5 g/L, Total bilirubin ≤ 1.5 × ULN, AST and ALT ≤ 2.0 × ULN, Alkaline phosphatase (ALP) ≤ 2.5 × ULN or, for patients

with documented liver or bone metastases, ALP ≤ 5 × ULN, Serum creatinine ≤ 1.5 × ULN

or creatinine clearance (CrCl) ≥ 40 mL/min on the basis of measured CrCl from a 24 -hour urine collection or Cockcroft-Gault glomerular filtration rate estimation:
CrCl = ((140 − age) / (serum creatinine in mg/dL)) × (weight in kg) (× 0.85 if female)/ 72).
Patients with BRAF mutation may be screened for eligibility in cohorts 2, 3, or 4 (in this order of preference) if they do not meet the entry criteria for cohort 1.

4.1.2 Exclusion Criteria

1. Subjects with an active, known or suspected autoimmune disease. Subjects with type I diabetes mellitus on stable insulin regimen, hypothyroidism only requiring hormone replacement, skin disorders (such as vitiligo, psoriasis, or alopecia) not requiring systemic treatment, or conditions not expected to recur in the absence of an external trigger are permitted to enroll.

2. For patients not receiving therapeutic anticoagulation: INR or aPTT >1.5 x ULN within 28 days prior to initiation of study treatment

3. Prior treatment with anti-PD-1, or anti-PD-L1 therapeutic antibody or pathway targeting agents. Patients who have received prior treatment with anti-CTLA-4 may be enrolled, provided the following requirements are met:
Minimum of 12 weeks from the first dose of anti-CTLA-4 and > 6 weeks from the last dose
No history of severe immune-related adverse effects from anti-CTLA 4 (NCI CTCAE Grade 3 and 4)

4. Known clinically significant liver disease, including active viral, alcoholic, or other hepatitis; cirrhosis; fatty liver; and inherited liver disease

5. History of HIV infection or active hepatitis B (chronic or acute) or hepatitis C infection
Patients with past or resolved hepatitis B infection (defined as having a negative hepatitis B surface antigen[HBsAg] test and apositive anti-HBc[antibody to hepatitis B core antigen] antibody test) are eligible. However, patients with past or resolved HBV should be monitored for reactivation by a specialist.
Patients positive for hepatitis C virus (HCV) antibody are eligible only if polymerase chain reaction (PCR) is negative for HCV RNA.. *Current treatment with anti-viral therapy for HBV.*

6. Pregnant or lactating women. All pre-menopausal women being screened must have a negative serum pregnancy test within 14 days prior to commencement of dosing. Women of non-childbearing potential may be included if they are either surgically sterile or have been postmenopausal for >/= 1 year. Fertile men and women must use an effective method of contraception during treatment and for at least 6 months after completion of treatment as directed by their physician.

7. Untreated brain metastases.

8. Chemotherapy within 21 days of enrollment with the exception of paclitaxel or nab-paclitaxel (Abraxane). Patients who have received one course of these agents prior to study entry are eligible. (One course of weekly paclitaxel or nab-paclitaxel is 3 doses. One

course of every 3 week dosing of paclitaxel or nab-paclitaxel is 1 dose). Patients who have received prior radiosensitizing chemotherapy are eligible.

9. The use of corticosteroids is not allowed for 10 days prior to initiation of atezolizumab except patients who are taking steroids for physiological replacement. Inhaled or topical steroids, and adrenal replacement steroid doses are permitted in the absence of active autoimmune disease. This does not apply to patients receiving steroids as pre-medications for paclitaxel administration.

10. Grade ≥ 2 uncontrolled hypertension (patients with a history of hypertension controlled with anti-hypertensive medication to Grade ≤ 1 are eligible)

11. Cobi Specific Exclusion Criteria

- Prior treatment with a MEK inhibitor
- Poorly controlled hypertension, defined as sustained, uncontrolled, non-episodic baseline hypertension (blood pressure [BP] consistently above 159/99 mmHg) despite optimal medical management
- History or presence of an abnormal ECG that is deemed clinically significant by the investigator, including complete left bundle branch block, second- or third-degree atrioventricular heart block, or evidence of prior myocardial infarction
- Known allergy or hypersensitivity to any component of the cobimetinib formulation
- History of malabsorption syndrome or other condition that would interfere with enteral absorption or results in the inability or unwillingness to swallow pills
- History or evidence of inherited bleeding diathesis or significant coagulopathy at risk for bleeding
- Any Grade ≥ 3 hemorrhage or bleeding event within 28 days prior to initiation of study treatment
- History of stroke, reversible ischemic neurological defect, or transient ischemic attack within 6 months prior to initiation of study treatment
- Consumption of foods, supplements, or drugs that are strong or moderate CYP3A4 enzyme inducers or inhibitors (e.g., St. John's wort, hyperforin, grapefruit juice) within 2 weeks prior to initiation of study treatment

12. ADDITIONAL EXCLUSION CRITERIA FOR non-BRAF/non-RAS MUTATION (COHORT 3):

a. Patients with clinically significant hemoptysis or tumor bleeding within two weeks prior to first dose of targeted therapy.

b. Patients with suspected tracheal or esophageal invasion are excluded from cohort3 due to the high risk of trachealesophageal fistula.

Patients excluded from cohort 3 may be enrolled on taxane + atezolizumab cohort (cohort 4).

13. **ADDITIONAL EXCLUSION CRITERIA FOR COHORTS 1 and 2:** Ocular Exclusion Criteria for cobimetinib containing cohorts—cohorts 1 and 2. (However, these patients may be assigned other cohorts if they do not meet the ocular exclusion criteria):

History of or evidence of retinal pathology on ophthalmologic examination that is considered a risk factor for neurosensory retinal detachment, central serous chorioretinopathy, retinal vein occlusion (RVO), or neovascular macular degeneration Patients will be excluded from participation in cohorts 1 and 2 if they currently are known to have any of the following risk factors for RVO, unless a retinal specialist has determined that the risk of retinal detachment is low:

a History of serous retinopathy
b. History of retinal vein occlusion
c. History of ongoing serous retinopathy or RVO at baseline

14. **ADDITIONAL EXCLUSION CRITERIA FOR PATIENTS IN COHORT 1 (vemurafenib+cobimetinib+atezolizumab):** Cardiac Exclusion Criteria:
History of clinically significant cardiac dysfunction, including the following:

a. Mean (average of triplicate measurements) QTc interval corrected using Fridericia’s method (QTcF) >= 480 ms at screening, or uncorrectable abnormalities in serum electrolytes (sodium, potassium, calcium, magnesium, and phosphorus)

15. **ADDITIONAL EXCLUSION CRITERIA FOR PATIENTS IN COHORTS 1 and 2 (cobimetinib-containing cohorts):** Cardiac Exclusion Criteria:

a. Unstable angina, or new-onset angina within 3 months prior to initiation of study treatment
b. Symptomatic congestive heart failure, defined as New York Heart Association Class II or higher
c. Myocardial infarction within 3 months prior to initiation of study treatment

d. Left ventricular ejection fraction below the institutional lower limit of normal or below 50%, whichever is lower

4.2 STUDY TREATMENT

4.2.1 <u>Study Treatment Plan:</u>

Induction phase with single agent taxanes: This phase is optional and serves only as a bridge to mutation-driven treatment assignment. For example, if molecular testing is readily available, patients may be assigned to the mutation-driven cohorts and do not require induction phase.

- nab-paclitaxel 100 mg/m2 weekly for up to 3 doses (preferred)

OR

-nab-paclitaxel 260 mg/m2 q 3 weeks (1 dose)

OR

- Paclitaxel 80 mg/m2 weekly for up to 3 doses

OR

-Paclitaxel 175 mg/m2 q 3 weeks (1 dose)

Mutation-driven, targeted treatments:

• Cohort 1, BRAF mutant: vemurafenib 960mg bid (day 1-21) + cobimetinib 60mg qday (day 1-21) run-in before starting atezolizumab. Vemurafenib will be dose reduced to 720mg bid (at cycle 1 day 21) and cobimetinib 60mg will be taken on days 1-21. Atezolizumab 840 mg IV on Day 1 and Day 15 in a 28-day cycle, will be started on Cycle 1, Day 1. Patients who have > grade 3 LFTs (AST, ALT or total bilirubin) will not receive atezolizumab but may continue on vemurafenib + cobimetinib with dose reduction. If after dose reductions, the LFTs are below grade 3, patient may start atezolizumab. See figure 2.

• Cohort 2, RAS, NF1, or NF2 mutant, including patients with tumors with MAPK activating mutations at or above MEK: cobimetinib 60 mg orally on Days 1−21 plus atezolizumab 840 mg IV on Day 1 and Day 15 in a 28-day cycle.

• Cohort 3, Non-BRAF/non-RAS mutant: atezolizumab 1200 mg IV every 21 days plus bevacizumab 15 mg/kg IV every 21 days in a 21-day cycle.

• Cohort 4, Taxane + atezolizumab: Nab-paclitaxel 100mg/m2 on days 1, 8, and 15. Atezolizumab 1200mg IV every 21 days. Paclitaxel may be substituted for nab-paclitaxel but nab-paclitaxel is preferred. The dose of paclitaxel is 175mg/m2 every 21 days. Any patient assigned to cohort 4 is eligible to receive nab-paclitaxel at a local facility.

Figure 2: Triplet dosing schedule with run-in for vemurafenib + cobimetinib + atezolizumab

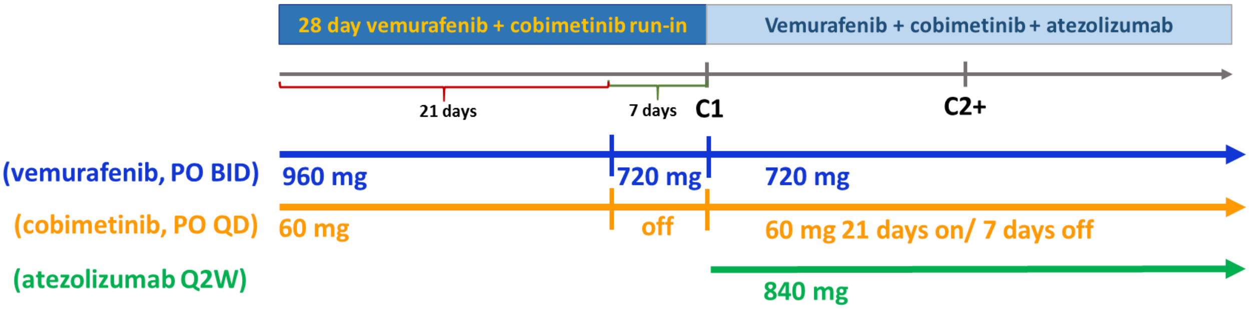

4.2.2 Study Drug: atezolizumab

4.2.1.1 Formulation

The atezolizumab drug product is provided in a single-use, 20-cc USP/Ph. Eur. Type 1 glass vial as a colorless-to-slightly-yellow, sterile, preservative-free clear liquid solution intended for IV administration. The vials are designed to deliver either 20 mL (1200 mg) or 14 mL (840 mg) of atezolizumab solution but may contain more than the stated volume to enable delivery of the entire 20 mL or 14 mL volume. The atezolizumab drug product is formulated as 60 mg/mL atezolizumab in 20 mM histidine acetate, 120 mM sucrose, 0.04% polysorbate 20, pH 5.8.

Atezolizumab must be refrigerated at 2°C − 8°C (36°F − 46°F) upon receipt until use. Atezolizumab vials should not be used beyond the expiration date provided by the manufacturer. No preservative is used in the atezolizumab drug product; therefore, each vial is intended for single use only. Discard any unused portion of drug left in a vial. Vial contents should not be frozen or shaken and should be protected from direct sunlight.

For further details, see the atezolizumab Investigator's Brochure.

4.2.1.2 Dosage, Administration, and Storage

The dose level of atezolizumab to be tested in this study is 840mg administered by IV infusion on Day 1 and Day 15 in a 28-day cycle in cohorts 1 and 2, and 1200 mg (equivalent to an average body weight−based dose of 15 mg/kg) administered by IV infusion every 3 weeks (21 [□ 2] days) in cohorts 3 and 4. Atezolizumab will be delivered in infusion bags with IV infusion lines that have product contacting surfaces of polyvinyl chloride (PVC) or polyolefin and 0.2 □m in-line filters (filter membrane of

polyethersulfone [PES]). No incompatibilities have been observed between atezolizumab and PVC or polyolefin infusion materials (bags or infusion lines).

Administration of atezolizumab will be performed in a setting with emergency medical facilities and staff who are trained to monitor for and respond to medical emergencies.

The initial dose of atezolizumab will be delivered over 60 (□ 15) minutes. If the first infusion is tolerated without infusion-associated AEs, the second infusion may be delivered over 30 (□ 10) minutes. If the 30-minute infusion is well tolerated, all subsequent infusions may be delivered over 30 (□ 10) minutes. For the first infusion, the patient's vital signs (heart rate, respiratory rate, blood pressure, and temperature) should be determined within 60 minutes before, during (every 15 [□ 5] minutes), and 30 (□ 10) minutes after the infusion. For subsequent infusions, vital signs will be collected within 60 minutes before and within 30 (□ 10) minutes after the infusion. Vital signs should be collected during the infusion only if clinically indicated. Patients will be informed about the possibility of delayed post-infusion symptoms and instructed to contact their study physician if they develop such symptoms.

No premedication will be allowed for the first dose of atezolizumab. Premedication may be administered for Cycles □ 2 at the discretion of the treating physician. The management of IRRs will be according to severity asfollows:

- In the event that a patient experiences a mild (NCI CTCAE Grade 1) IRR, the infusion rate should be reduced to half the rate being given at the time of event onset. Once the event has resolved, the investigator should wait for 30 minutes while delivering the infusion at the reduced rate. If tolerated, the infusion rate may then be increased to the original rate.
- In the event that a patient experiences a moderate IRR (NCI CTCAE Grade 2) or flushing, fever, or throat pain, the infusion should be immediately interrupted and the patient should receive aggressive symptomatic treatment. The infusion should be restarted only after the symptoms have adequately resolved to baseline grade. The infusion rate at restart should be half of the infusion rate that was in progress at the time of the onset of the IRR.
- For severe or life-threatening IRRs (NCI CTCAE Grade 3 or 4), the infusion should be stopped immediately, and aggressive resuscitation and supportive measures should be initiated. Patients experiencing severe or life-threatening IRRs will not receive further infusion and will be further managed as clinically indicated until the event resolves.

For anaphylaxis precautions, see Appendix 7.

Guidelines for dosage modification, treatment interruption, or discontinuation and the management of specific adverse events are provided in Section 0 and Section 4.3.1, respectively.

4.2.2 Other Study Drug(s): Vemurafenib, cobimetinib, bevacizumab

4.2.3 Vemurafenib Dosage, Storage, and Administration

The dose of vemurafenib at 960 mg BID was identified in the Phase I dose-finding study PLX 06-02 and is established as the recommended dosage for Phase II and III trials for the treatment of melanoma.

The formulated vemurafenib drug product is provided as 240 -mg film-coated tablets packed in bottles for oral administration. For additional batch -specific instructions and information, vemurafenib will be labeled in compliance with Good Manufacturing Procedures (GMP). The drug label will include the contents, protocol number, batch number, and storage conditions, as well as any required statements that the drug is: “For Clinical Trial Use Only.” Patients will be requested to store the vemurafenib at the recommended storage conditions noted on the label out of the reach of children or other cohabitants. For further details, please see the Investigator’s Brochure.

Vemurafenib may be crushed for those patients who are unable to swallow pills and will be administered orally or via PEG tube. Instructions will be provided to the patient. See Appendix 13

4.2.4 Cobimetinib Dosage, Storage, and Administration

The recommended dose for cobimetinib is 60 mg QD for 21 days on, then 7 days off, in a 28 day treatment cycle. The 20 mg cobimetinib drug product is a film-coated, immediate release tablet. The white tablet is round with the engraving “ROCHE” on one side. Cobimetinib will be packaged in blister packs. Cobimetinib should not be stored above 25°C (77°F).

Cobimetinib should be taken once daily at approximately the same time each day with the morning vemurafenib dose, and no later than 4 hours after the scheduled time. Cobimetinib can be taken with or without a meal. Cobimetinib tablets should never be chewed, cut, or crushed. At least 7 days off cobimetinib is required prior to starting a new treatment cycle.

Cobimetinib suspension will be used in patients who are unable to swallow pills and will be administered orally or via PEG tube. Reconstitution instructions will be provided to the patient. Suspension Box contains: One bottle cobimetinib 250 mg powder for reconstitution for oral suspension, one 50 ml measuring cup, and one bag with 2 press - in-bottle adapters.

There is a risk of overexposure from reconstitution of cobimetinib powder for oral suspension. If more than 10 reconstitutions of cobimetinib powder for oral suspension are made per day, wearing respiratory protection during the reconstitution or using an exhaust hood (e.g. ventilation booth) is recommended.

4.2.5 Bevacizumab Dosage, Storage, and Administration

BEVACIZUMAB DOSAGE AND FORMULATION

Bevacizumab 15 mg/kg q3 weeks IV is the dosage that will be used in conjunction with atezolizumab.

Bevacizumab is manufactured by recombinant DNA technology, u sing a genetically engineered Chinese hamster ovary (CHO) cell line. The protein is purified from the cell culture medium by routine methods of column chromatography and filtration. The final product is tested for quality, identity, safety, purity, potency, strength, and excipient/chemical composition according to International Conference on Harmonisation (ICH) guidelines. The purity of bevacizumab is > 95%.

Bevacizumab may be supplied in 6-cc (100-mg) and 20-cc (400-mg) glass vials containing 4 mL or 16 mL of bevacizumab, respectively (all at 25 mg/mL). Vials contain bevacizumab with phosphate, trehalose, polysorbate 20, and sterile water for injection (SWFI). Vials contain no preservative and are suitable for single use only.For further details and molecule characterization, see the bevacizumab Investigator Brochure.

Bevacizumab Administration

Bevacizumab should be prepared using aseptic technique. Withdraw the necessary amount of bevacizumab and dilute to the required administration volume with 0.9% Sodium Chloride Injection, USP. Administration will be as a continuous IV infusion. Anaphylaxis precautions should be observed during study drug administration.

The initial dose will be delivered over 90±15 minutes. If the first infusion is tolerated without infusion-associated adverse events (fever and/or chills), the second infusion may be delivered over 60±10 minutes. If the 60-minute infusion is well tolerated, all subsequent infusions may be delivered over 30 ±10 minutes.

If a subject experiences an infusion–associated adverse event, he or she may be premedicated for the next study drug infusion; however, the infusion time may not be decreased for the subsequent infusion. If the next infusion is well tolerated with premedication, the subsequent infusion time may then be decreased by 30±10 minutes as long as the subject continues to be premedicated. If a subject experiences an infusion-associated adverse event with the 60-minute infusion, all subsequent doses should be given over 90±15 minutes. Similarly, if a subject experiences an infusion-associated adverse event with the 30-minute infusion, all subsequent doses should be given over 60±10 minutes.

Bevacizumab Storage

Upon receipt of the study drug, vials are to be refrigerated at 2° C–8° C

(36°F–46°F) and should remain refrigerated until just prior to use. DO NOT FREEZE. DO NOT SHAKE. Keep vial in the outer carton due to light sensitivity.

VIALS ARE FOR SINGLE USE ONLY. Vials used for 1 subject may not be used for any other subject. Chemical and physical in-use stability has been demonstrated for 48 hours at 2°C–30°C in 0.9% Sodium Chloride solution. If not used immediately, in-use storage times and conditions are the responsibility of the user and would normally not be longer than 24 hours at 2°C–8°C, unless dilution has taken place in a controlled and validated aseptic conditions.

4.2.6 Protocol-Specified Chemotherapy (nab-paclitaxel or paclitaxel)

The recommended dose for nab-paclitaxel (preferred over paclitaxel) is 100mg/m2 IV on days 1, 8, and 15. The dose of paclitaxel is 175mg/m2 IV every 21 days. Standard steroid pre-medication should be avoided for nab-paclitaxel when possible.

4.2.7 Summary of Pharmacy Data

The study drugs may be prepared, handled, and administered per their respective current FDA-approved package inserts and will be provided by Genentech as shown in the table below:

Drug Name	Package Format
Avastin (bevacizumab)	400 mg/16 mL solution for injection
atezolizumab (MPDL3208A)	1200 mg/ 20 mL solution for injection
Vemurafenib	120 tablets/ bottle
Cobimetinib, oral<br><br>Cobimetinib, suspension	One kit includes 63 tablets (3 blister packs of 21 tablets each)<br>Suspension Bottle:<br>Cobimetinib powder 250 mg for reconstitution for oral suspension<br><br>Suspension Box contains: One bottle cobimetinib powder 250 mg for reconstitution for oral suspension, one 50 ml measuring cup and one bag with 2 press-in-bottle adapters.

4.2.8 Concomitant Therapy and Additional Restrictions

Concomitant therapy includes any medication (e.g., prescription drugs, over-the-counter drugs, vaccines, herbal or homeopathic remedies, nutritional supplements) used by a patient in addition to protocol-mandated study treatment from 7 days prior to signing of the Informed Consent Form through 30 days after the last dose of study treatment. All

such medications should be reported to the investigator and recorded on the Concomitant Medications eCRF.

4.2.8.1 Permitted Therapy

Patients who use oral contraceptives, hormone-replacement therapy, prophylactic anticoagulation therapy (such as low-molecular-weight heparin at a stable dose level), or other allowed maintenance therapy should continue their use. Male and female patients of reproductive potential should use highly effective means of contraception

Patients are permitted to use the following therapies during the study:

- Oral contraceptives
- Hormone-replacement therapy
- Prophylactic or therapeutic anticoagulation therapy (such as warfarin at a stable dose or low-molecular-weight heparin)
- Inactivated influenza vaccinations
- Megestrol administered as an appetite stimulant
- Inhaled corticosteroids
- Mineralocorticoids
- Low-dose corticosteroids administered for orthostatic hypotension or adrenocortical insufficiency
- Pain medications as indicated, as per standard practice

At the discretion of the investigator, anti-emetic medications, antidiarrheal medications, and may be administered prophylactically per standard local practice before the second and subsequent doses of study treatment. Anti-emetic medications, antidiarrheal medications, and hematopoietic growth factors are not to be administered prophylactically prior to initiation of study treatment.

Patients requiring treatment for toxicities or for co-existent conditions may be treated as clinically indicated. Planned use of other medications should be discussed with the Medical Monitor.

Premedication with antihistamines may be administered for the second and subsequ ent atezolizumab infusions only, at the discretion of the investigator.

Patients who experience infusion-associated symptoms may be treated symptomatically with ibuprofen, diphenhydramine, and/or H_2-receptor antagonists (e.g., famotidine, cimetidine), or equivalent medications per local standard practice (acetaminophen is prohibited for cohort 1; see Section 4.2.8.2). Serious infusion-associated events manifested by dyspnea, hypotension, wheezing, bronchospasm, tachycardia, reduced oxygen saturation, or re spiratory distress should

be managed with supportive therapies as clinically indicated (e.g., supplemental oxygen and □2-adrenergic agonists; see Appendix 7).

4.2.8.2 Prohibited Therapy

Use of the following concomitant therapies is prohibited as described below:

Prohibited therapies applicable to all patients

- Concomitant therapy intended for the treatment of cancer (including, but not limited to, chemotherapy, hormonal therapy, immunotherapy, and herbal therapy), whether health authority−approved or experimental, is prohibited for various time periods prior to starting study treatment, depending on the agent and during study treatment until disease progression is documented and the patient has discontinued study treatment. The only exception is cytotoxic chemotherapy used concomitantly with adjuvant radiation.
- Investigational therapy is prohibited during the study.
- Anti-emetic medications, antidiarrheal medications, and hematopoietic growth factors are not to be administered prophylactically prior to initiation of st udy treatment.
- Anti-arrhythmic agents and medications with a risk of torsades de pointes are prohibited within 7 days prior to initiation of study treatment, during study treatment, and for 30 days after the last dose of study treatment.

Prohibited therapies applicable to patients receiving atezolizumab (or atezolizumab placebo):

- Live, attenuated vaccines (e.g., FluMist□) are prohibited within 4 weeks prior to initiation of atezolizumab, during atezolizumab treatment, and for 5 months after the last dose of atezolizumab.
- Systemic immunostimulatory agents (including, but not limited to, interferons and IL-2) are prohibited within 4 weeks or five half -lives of the drug, whichever is longer, prior to initiation of atezolizumab and during atezolizumab treatment because these agents could potentially increase the risk for autoimmune conditions when given in combination with atezolizumab.
- Systemic immunosuppressive medications (including, but not limited to, cyclophosphamide, azathioprine, methotrexate, and thalidomide) are prohibited during atezolizumab treatment because these agents could potentially alter the efficacy and safety of atezolizumab. However, systemic corticosteroids can be given as premedication to patients for whom CT scans with contrast are contraindicated (i.e., patients with contrast allergy or impaired renal clearance).

Prohibited therapies applicable to patients receiving cobimetinib or vemurafenib:

- St. John's wort is prohibited during cobimetinib and vemurafenib treatment and for 2 weeks after the last dose of cobimetinib or vemurafenib (whichever is later)

<u>Prohibited therapies applicable to patients receiving vemurafenib:</u>

- Acetaminophen is prohibited within 7 days prior to initiation of study treatment, during study treatment, and for 30 days after the last dose of study treatment, unless the patient has an absolute contraindication to the use of non-steroidal anti-inflammatory drugs (NSAIDs) or aspirin (i.e., severe allergy or reactive airway disease sensitive to NSAIDs or aspirin)

Patients who require the use of any of the agents listed above will be discontinued from study treatment (unless use is agreed upon in consultation with the Medical Monitor) and followed for safety outcomes for 30 days after their last dose of study treatment or until they receive another anti-cancer therapy, whichever occurs first.

4.2.8.3 Prohibited Foods and Drinks

Use of grapefruit juice, a potent CYP3A4 enzyme inhibitor, is prohibited during the study and for 30 days after the last dose of study treatment. Patients must not drink alcohol while receiving study treatment.

4.2.8.4 Cautionary Medications

Antiplatelet agents should be used with caution during study treatment.

Systemic corticosteroids and TNF-□ inhibitors may attenuate potential beneficial immunologic effects of treatment with atezolizumab. For treatment of vemurafenib-related adverse events (other than hepatotoxicity), systemic corticosteroids may be administered at the lowest effective dose and for the shortest possible duration necessary. If feasible, alternatives to these agents should be considered. Topical corticosteroids are allowed for suspected cutaneous autoimmune reactions. Megestrol may be administered as an appetite stimulant during the study. In situations in which systemic corticosteroids or TNF-□ inhibitors would be routinely administered, alternatives, including antihistamines, should be considered. If the alternatives are not feasible, systemic corticosteroids and TNF-□ inhibitors may be administered at the discretion of the investigator.

4.3 SYSTEMIC CORTICOSTEROIDS ARE RECOMMENDED, AT THE DISCRETION OF THE INVESTIGATOR, FOR THE TREATMENT OF SPECIFIC ADVERSE EVENTS WHEN ASSOCIATED WITH ATEZOLIZUMAB THERAPY. GENERAL PLAN TO MANAGE SAFETY CONCERNS

Measures will be taken to ensure the safety of patients participating in this trial, including the use of stringent inclusion and exclusion criteria (see Section 4.1.1 and Section 4.1.2) and close monitoring (as indicated below and in Section 4.7.2). See Section 6.3.2 for complete details regarding safety reporting for this study.

Eligibility Criteria

Eligibility criteria were selected to guard the safety of patients in this trial. Results from the nonclinical toxicology studies with atezolizumab, as well as the nonclinical/clinical data from other PD-L1/PD-1 inhibitors, were taken into account (see Section 4.1.2).

Monitoring

Safety will be evaluated in this study through the monitoring of all serious and non-serious AEs, defined and graded according to NCI CTCAE v4.0. Patients will be assessed for safety (including laboratory values) according to the schedule in Appendix 1. Patients will be followed for safety for 90 days following the last dose of study treatment or until receipt of another anticancer therapy, whichever comes first.

General safety assessments will include serial interval histories, physical examinations, and specific laboratory studies, including serum chemistries and blood counts (see Appendix 1 for the list and timing of study assessments). All serious adverse events (SAEs) and protocol-defined events of special interest (see Section 6.2.2) will be reported in an expedited fashion (see Section 6.3). In addition, the investigators will review and evaluate observed AEs on a regular basis.

Patients who have an ongoing study treatment−related AE upon study completion or at discontinuation from the study will be followed until the event has resolved to baseline grade, the event is assessed by the investigator as stable, new anticancer treatment is initiated, the patient is lost to follow-up, the patient withdraws consent, or until it has been determined that study treatment or participation is not the cause of the AE.

4.3.1 Management of Specific Safety Concerns with atezolizumab

Toxicities associated or possibly associated with atezolizumab treatment should be managed according to standard medical practice. Additional tests, such as autoimmune serology or biopsies, may be used to determine a possible immunogenic etiology.

Although most immune-related adverse events (irAEs) observed with immunomodulatory agents have been mild and self -limiting, such events should be recognized early and treated promptly to avoid potential major complications. (Di Giacomo et al. 2010). Discontinuation of atezolizumab may not have an immediate therapeutic effect and, in severe cases, immune-related toxicities may require acute management with topical corticosteroids, systemic corticosteroids, or additional immunosuppressant (i.e., mycophenolate, or TNF□ inhibitors).

The primary approach to Grade 1 to 2 irAEs is supportive and symptomatic care with continued treatment with atezolizumab; for higher-grade irAEs, atezolizumab should be withheld and oral and/or parenteral steroids administered. Recurrent Grade 2 irAEs

may also mandate withholding atezolizumab or the use of steroids. Assessment of the benefit-risk balance should be made by the investigator, with consideration of the totality of information as it pertains to the nature of the toxicity and the degree of clinical benefit a given patient may be experiencing prior to further administration of atezolizumab. atezolizumab should be permanently discontinued in patients with life-threatening irAEs.

4.3.2 Guidelines for Dosage Modification and Treatment Interruption or Discontinuation of atezolizumab

Separate management guidelines have been developed for the cobimetinib plus atezolizumab (appendix 10) and cobimetinib plus vemurafenib plus atezolizumab (appendix 9) regimens.

Atezolizumab treatment will be given as long as the patient continues to experience clinical benefit in the opinion of the investigator until the earlier of unacceptable toxicity, symptomatic deterioration attributed to disease progression, or any of the other reasons for treatment discontinuation listed in Section 4.6.

There will be no dose reduction for atezolizumab in this study. Patients may temporarily suspend study treatment for up to 105 days beyond the scheduled date of delayed infusion if study drug-related toxicity requiring dose suspension is experienced. If atezolizumab is held because of AEs for □ 84 days beyond the scheduled date of infusion, the patient will be discontinued from atezolizumab and will be followed for safety and efficacy as specified in Section 6.3.1. Targeted therapies with cobimetinib plus or minus vemurafenib (for cohorts 1 and 2), may be continued despite atezolizumab drug hold or discontinuation. See appendices 9 and 10.

If a patient must be tapered off steroids used to treat AEs, atezolizumab may be held for additional time beyond 105 days from the scheduled dose until steroids are discontinued or reduced to a prednisone dose (or dose equivalent) of □ 10 mg/day. The acceptable length of interruption will be at the discretion of the investigator.

Dose interruptions for reasons other than toxicity, such as surgical procedures, may be allowed. The acceptable length of interruption will be at the discretion of Genentech. The source document confirming length of treatment interruption will be documented and filed in the regulatory binder.

Patients should be assessed clinically (including review of laboratory values) for toxicity prior to, during, and after each infusion. If unmanageable toxicity due to atezolizumab occurs at any time during the study, treatment with atezolizumab should be discontinued.

4.4 STUDY TREATMENT MODIFICATIONS

Guidelines for dose modifications, interruptions, or discontinuations of vemurafenib and/or cobimetinib provided below are not intended to replace the investigator's best clinical judgment with regard to the medical management of her/his individual patients.

4.4.1 <u>Dose Modifications for Vemurafenib</u>

4.4.1.1 For New Primary Cutaneous Malignancies

No dose modifications are recommended.

4.4.1.2 For Other Adverse Reactions

Permanently discontinue vemurafenib for any of the following:

- Grade 4 adverse reaction, first appearance (if clinically appropriate) or second appearance
- QTc prolongation □ 500 msec and increased by □ 60 msec from pre-treatment values. In addition, electrolytes (K, Mg, and Ca) should be monitored and any electrolyte abnormalities should be corrected prior to reinstitution of therapy. ECG should be monitored weekly until the QTc interval decreases to □ 500 msec or returns to baseline before reinstituting therapy at a reduced dose (see Table 9).

Table 9 Vemurafenib Dose Modification Schedules for QTc Prolongation

Dose Modification Schedule Based on Prolongation of the QT Interval - QTc Value	Recommended Dose Modification
QTc □ 500 msec at baseline	Treatment not recommended.
QTc increase meets values of both □ 500 msec and >60 msec change from pre-treatment values	Discontinue permanently.
1st occurrence of QTc □ 500 msec during treatment and change from pre-treatment value remains □ 60 msec	• Temporarily interrupt treatment until QTc decreases below 500 msec.<br>• See monitoring measures in the Investigator's Brochure (Section 4.4).<br>• Resume dosing at 720 mg twice daily (or 480 mg twice daily if the dose has already been lowered).
2nd occurrence of QTc □ 500 msec during treatment and change from pre-treatment value remains □ 60 msec	• Temporarily interrupt treatment until QTc decreases below 500 msec.<br>• See monitoring measures in the Investigator's Brochure (Section 4.4). Resume dosing at 480 mg twice daily (or discontinue permanently if the dose has already been lowered to 480 mg twice daily).
3rd occurrence of QTc □ 500 msec during treatment and change from pre-treatment value remains □ 60 msec	Discontinue permanently.

Table 10 Vemurafenib General Dose Modifications

Toxicity Grade (CTCAE v.4.0)	Recommended Dose Modification of vemurafenib
Grade 2 (Intolerable) or Grade 3 [a]	
1st occurrence	• Withhold until recovery to Grade 0-1<br>• Restart at a reduced dose of 720 mg twice daily
2nd occurrence	• Withhold until recovery to Grade 0-1<br>• Restart at a reduced dose of 480 mg twice daily
Grade 4	
1st occurrence	• Discontinue permanently or withhold until recovery to Grade 0-1<br>• Restart at reduced dose of 480 mg twice daily
2nd occurrence	• Discontinue permanently

[a] does not apply to intermittent grade 3 abnormal lymphocytes that have no clinical significance

4.4.2 Dose Modification Guidelines for Cobimetinib

Table 11 Cobimetinib General Dose Modifications

**Toxicity Grade (CTCAE v.4.0)[a]**	**Recommended Dose Modification of cobimetinib**
Grade 2 (Intolerable) or ≥ Grade 3	
1st occurrence	• Withhold until recovery to Grade 0-1<br>• Restart at a reduced dose of 40 mg
2nd occurrence	• Withhold until recovery to Grade 0-1<br>• Restart at a reduced dose of 20 mg
3rd occurrence	Permanently discontinue (unless benefits for the individual patient are deemed to outweigh the risks)

[a] does not apply to intermittent grade 3 abnormal lymphocytes that have no clinical significance

Table 12 Cobimetinib or Cobimetinib/Vemurafenib Dose Modification Guidelines for Left Ventricular Dysfunction

Toxicity	Description	Management
LVEF Decrease from Baseline	Asymptomatic	Withhold cobimetinib dosing for at least 2 weeks for either:<br>• A drop in LVEF to < 40% or<br>• LVEF of 40% to 49% with a □ 10% absolute decrease below pretreatment values<br>If LVEF improves to > 40% or to < 10% absolute decrease below pre-treatment values, resume cobimetinib at:<br>• 40 mg for first appearance of LVEF decline<br>• 20 mg for second appearance of LVEF decline<br>• Permanently discontinue cobimetinib for a third episode of LVEF decline<br>If LVEF remains < 40% or □ 10% absolute decline below pretreatment values after withholding cobimetinib for at least 2 weeks, consider permanently discontinuing cobimetinib
LVEF Decrease from Baseline	Symptomatic	Withhold cobimetinib dosing for at least 4 weeks.<br>Permanently discontinue cobimetinib if after 4 weeks patient continues to have symptomatic heart failure despite maximal supportive care or if LVEF has not recovered to > 40% or to < 10% absolute decrease below pretreatment values.<br>If heart failure symptoms have resolved following treatment break and LVEF has recovered to > 40% or to < 10% absolute decrease below pretreatment values, resume cobimetinib at:<br>• 40 mg for first appearance of LVEF decline<br>• 20 mg for second appearance of LVEF decline<br>• Permanently discontinue cobimetinib for a third episode of LVEF decline<br>Vemurafenib treatment can be continued if cobimetinib treatment is modified, if clinically indicated.

Table 13 Cobimetinib Dose Modification Guidelines for Specified Adverse Drug Reactions

Severity of Adverse Reaction[a]	Dose Modification for Cobimetinib
New Primary Malignancies (cutaneous and non-	No dose modification is required.
Hemorrhage	
Grade 3	Withhold cobimetinib for up to 4 weeks.<br>• If improved to Grade 0 or 1, resume at the next lower dose level.<br>• If not improved within 4 weeks, permanently discontinue.
Grade 4	Permanently discontinue.
Cardiomyopathy	
Asymptomatic, absolute decrease in LVEF from baseline of greater than 10% and less than institutional lower limit of normal (LLN)	Withhold cobimetinib for 2 weeks; repeat LVEF.<br><br>Resume at next lower dose if all of the following are present<br>• LVEF is at or above LLN and<br>• Absolute decrease from baseline LVEF is 10% or less.<br><br>Permanently discontinue if any of the following are present<br>• LVEF is less than LLN or<br>• Absolute decrease from baseline LVEF is more than 10%.
Symptomatic LVEF decrease from baseline	Withhold cobimetinib for up to 4 weeks, repeat LVEF.<br><br>Resume at next lower dose if all of the following are present:<br>• Symptoms resolve and<br>• LVEF is at or above LLN and<br>• Absolute decrease from baseline LVEF is 10% or less.<br><br>Permanently discontinue if any of the following are present<br>• Symptoms persist, or<br>• LVEF is less than LLN, or<br>• Absolute decrease from baseline LVEF is more than 10%.
Dermatologic Reactions	
Grade 2 (intolerable), Grade 3 or 4	Withhold or reduce dose.
Serous Retinopathy or Retinal Vein Occlusion	

Serous retinopathy	Withhold cobimetinib for up to 4 weeks.<br>• If signs and symptoms improve, resume at the next lower dose level.<br>• If not improved or symptoms recur at the lower dose within 4
Retinal vein occlusion	Permanently discontinue cobimetinib.

Severity of Adverse Reaction[a]	Dose Modification for cobimetinib
Liver Laboratory Abnormalities and Hepatotoxicity	
First occurrence Grade 4	Withhold cobimetinib for up to 4 weeks.<br>• If improved to Grade 0 or 1, then resume at the next lower dose level.<br>• If not improved to Grade 0 or 1 within 4 weeks,
Recurrent Grade 4	Permanently discontinue cobimetinib.
Rhabdomyolysis and Creatine Phosphokinase (CPK) elevations	
• Grade 4 CPK elevation<br>• Any CPK elevation and myalgia	Withhold cobimetinib for up to 4 weeks.<br>• If improved to Grade 3 or lower, resume at the next lower dose level.
Photosensitivity	
Grade 2 (intolerable), Grade 3 or Grade 4	Withhold cobimetinib for up to 4 weeks.<br>• If improved to Grade 0 or 1, resume at the next lower dose level.<br>• If not improved within 4 weeks, permanently discontinue.
Other	
• Grade 2 (intolerable) adverse reactions<br>• Any Grade 3 adverse reactions[b]	Withhold cobimetinib for up to 4 weeks.<br>• If improved to Grade 0 or 1, resume at the next lower dose level.<br>• If not improved within 4 weeks, permanently discontinue.
First occurrence of any Grade 4 adverse reaction	• Withhold cobimetinib until adverse reaction improves to Grade 0 or 1.<br>Then resume at the next lower dose level, *OR*
Recurrent Grade 4 adverse reaction	Permanently discontinue cobimetinib.

[a] National Cancer Institute Common Terminology Criteria for Advers e Events version 4.0 (NCI CTCAE v4.0)

[b] does not apply to intermittent grade 3 abnormal lymphocytes that have no clinical significance

4.4.3 Dose Modification Guidelines for bevacizumab

Bevacizumab dose will not be reduced for reasons other than a >10% change in weight from baseline. Bevacizumab treatment may be either temporarily or permanently suspended in the case of bevacizumab-related events such as fistulae, GI perforation, hypertension, proteinuria, thrombosis/embolism ,

hemorrhage, CHF, wound healing complications, PRES (or RPLS) and hypersensitivity/allergic reactions in addition to any other serious bevacizumab-related toxicity (grade 3 or 4).

In addition, bevacizumab should be temporarily withheld in the event of febrile grade 4 neutropenia and/or grade 4 thrombocytopenia (regardless of the relationship to treatment), since these conditions are predisposing factors for an increased bleeding tendency. To summarize:

Table 14 Bevacizumab Dose Modification Guidelines for Specified Adverse Drug Reactions

Toxicity	Description	Management
Febrile Neutropenia	Grade 4	Hold treatment temporarily, (regardless of relationship to treatment)
Thrombocytopenia	Grade 4	Hold treatment temporarily, (regardless of relationship to treatment)
Fistula	Grade ≥ 2	Hold temporarily or permanently discontinue
GI Perforation		Permanently discontinue
Major surgery or wound healing complications		Hold temporarily or permanently discontinue
Hypertension	Medically significant hypertension not controlled with antihypertensive therapy, hypertensive crisis or hypertensive encephalopathy	Permanently discontinue
Left ventricular dysfunction (CHF)	Grade ≥ 3	Permanently discontinue
Nephrotic syndrome		Permanently discontinue
Arterial thrombosis/embolism	Any Grade	Permanently discontinue
Venous thrombosis/embolism	Grade ≥ 3	Hold temporarily or permanently discontinue
CNS bleeding	Any Grade	Permanently discontinue
Bleeding	Grade ≥ 3	Permanently discontinue

Haemoptysis	Grade ≥ 2	Hold temporarily or permanently discontinue
PRES (or RPLS)		Permanently discontinue
Hypersensitivity/Allergic reactions		Permanently discontinue

Posterior Reversible Encephalopathy Syndrome (PRES/RPLS)

There have been rare reports of patients treated with bevacizumab developing signs and symptoms that are consistent with PRES, a rare neurological disorder. PRES is also known as reversible posterior leukoencephalopathy syndrome or RPLS). PRES can present with following signs and symptoms among others: seizures, headache, altered mental status, visual disturbance or cortical blindness, with or without associated hypertension. A diagnosis of PRES requires confirmation by brain imaging. In patients developing PRES, treatment of specific symptoms including control of hypertension is recommended along with discontinuation of bevacizumab. The safety of reinitiating bevacizumab therapy in patients previously experiencing PRES is not known. Adequate brain imaging using MRI must be performed as a follow-up measurement for patients with PRES.

Gastrointestinal Perforation and Fistula

Bevacizumab has been associated with serious cases of GI perforation in patients with mCRC and a few reports of gallbladder perforation have been reported from the post-marketing experience. The presentation of these events has varied in type and severity, ranging from free air seen only on the plain abdominal X-ray, which resolved without treatment, to a colonic perforation with abdominal abscess and fatal outcome. The common feature among these cases was intra abdominal inflammation, either from gastric ulcer disease, tumour necrosis, diverticulitis or chemotherapy-associated colitis. Nevertheless, a causal association of an intra-abdominal inflammatory process and GI perforation to treatment with bevacizumab has not been established. However, caution should be exercised when treating patients with intra-abdominal inflammatory process with bevacizumab.

Bevacizumab should be permanently discontinued in patients who develop GI perforation.

Bevacizumab use has been associated with serious cases of fistulae including events resulting in death. Fistulae within the GI tract or GI tract and skin are common in patients with mCRC and ovarian cancer, but are uncommon or rare in other indications. Other fistulae (e.g. tracheoesophageal, bronchopleural, urogenital, biliary) have been reported uncommonly in bevacizumab clinical trials patients and in post-marketing reports.

Temporarily discontinue bevacizumab in patients with grade 2 or 3 non-tracheoesophageal fistula until resolution to ≤ grade 1.

Permanently discontinue bevacizumab in patients with tracheoesophageal fistula or any grade 4 fistula. Limited information is available on the continued use of bevacizumab in patients with other fistulae. In cases of internal fistula not arising in the GI tract, discontinuation of bevacizumab should be considered.

Wound Healing Complications

Increased incidences of post-operative bleeding or wound healing complications have been observed in clinical trials of bevacizumab in relapsed glioma and mCRC and BC.

Bevacizumab therapy should not be initiated for at least 28 days following major surgery or until the surgical wound is fully healed as bevacizumab may adversely impact wound healing.

In patients who experience wound healing complications during bevacizumab treatment, bevacizumab should be withheld until the wound is fully healed. If the wound does not fully heal despite withholding treatment, should be permanently discontinued.

Bevacizumab therapy should be withheld for an interval of at least two half -lives (approximately six weeks) before conducting major elective surgery. Emergency surgery should be performed as appropriate without delay after a careful risk - benefit assessment.

Hypertension

An increased incidence of hypertension has been observed in patients treated with bevacizumab. Clinical safety data suggest the incidence of hypertension is likely to be dose-dependent.

Pre-existing hypertension should be adequately controlled before starting bevacizumab treatment. There is no information on the effect of bevacizumab in patients with uncontrolled hypertension at the time of initiating therapy.

Blood pressure must be assessed before each bevacizumab administration.

In most cases hypertension is controlled adequately using standard antihypertensive treatment appropriate for the individual situation of the affected patient. Bevacizumab should be permanently discontinued if medically significant hypertension cannot be adequately controlled with antihypertensive therapy, or if the patient develops hypertensive crisis or hypertensive encephalopathy.

Congestive Heart Failure (CHF)

Events consistent with CHF were reported in clinical trials. The findings ranged from asymptomatic declines in left ventricular ejection fraction (LVEF) to symptomatic CHF, requiring treatment or hospitalisation. Most of the patients who experienced CHF had metastatic breast cancer and had received previous treatment with anthracyclines, prior radiotherapy to the left chest wall or other risk factors for CHF were present.

Caution should be exercised when treating patients with clinically significant cardiovascular disease such as pre-existing coronary artery disease, concomitant cardiotoxic therapy or CHF with bevacizumab.

Bevacizumab should be permanently discontinued in patients with ≥ grade 3 CHF.

4.4.3.1 Proteinuria

In clinical studies, the incidence of proteinuria was higher in patients receiving bevacizumab in combination with chemotherapy compared to those who received chemotherapy alone. Proteinuria reported as an AE with bevacizumab treatment has ranged in severity from clinically asymptomatic, transient, trace proteinuria to nephrotic syndrome with the great majority as grade 1 proteinuria. The proteinuria seen in bevacizumab clinical trials was not associated with renal dysfunction and rarely required permanent discontinuation of bevacizumab therapy. Patients with

a history of hypertension may be at increased risk for the development of proteinuria when treated with bevacizumab.

Proteinuria must be assessed by urinalysis before each bevacizumab administration unless proteinuria has been determined by 24-hour urine collection.

Table 15 Bevacizumab Treatment Management for Proteinuria

NCI CTCAE v. 4.0	Urinalysis	Treatment Action
Grade 1	1+ proteinuria<br>urinary protein < 1.0 g/24 hrs	No bevacizumab dose modification
Grade 2	2+ proteinuria<br>urinary protein 1.0-3.4 g/24 hrs	For 2+ proteinuria: may administer bevacizumab without dose modification and collect 24-hour urine prior to subsequent bevacizumab administration.<br>For 3+ proteinuria: must obtain 24-hour urine prior to administering bevacizumab<br>Suspend bevacizumab for urinary protein ≥2 g/24 hrs. Resume bevacizumab when proteinuria is < 2 g/24 hrs.
Grade 3	urinary protein ≥3.5 g/24 hrs	Suspend bevacizumab.<br>Resume bevacizumab when proteinuria is < 2 g/24 hrs.
**Nephrotic syndrome**		Permanently discontinue bevacizumab

<u>Arterial thrombosis/embolism</u>

Bevacizumab should be discontinued in patients who develop arterial thromboembolic events.

A history of arterial thromboembolic events or age greater than 65 years has been associated with an increased risk of arterial thromboembolic events during bevacizumab therapy. Patients receiving bevacizumab plus chemotherapy with a history of arterial thromboembolism and age greater than 65 years have a higher risk. Caution should be taken when treating these patients with bevacizumab.

<u>Venous thrombosis/embolism</u>

Bevacizumab should be held in patients developing a grade 3 thrombosis/embolism. Bevacizumab may be resumed once the patient is adequately anti-coagulated and on a stable level of anticoagulation for at least 2 weeks prior to restarting study drug treatment. Patients on heparin treatment should have an aPTT between 1.5-2.5 x ULN (or patient value before starting heparin treatment). Patients on Coumadin derivatives should have an INR

between 2.0 and 3.0 assessed in two consecutive measurements 1-4 days apart. Patients on full dose low molecular weight heparins should receive the appropriate dose based on the weight of the patient according to package insert.

An increased risk of venous thromboembolic events and bleeding in patients receiving anti-coagulation therapy after first venous thromboembolic event while receiving bevacizumab has been observed.

In the event of recurrent grade 3 thrombosis/embolism, the patient should be discontinued from bevacizumab.

Bevacizumab should be discontinued in patients with life-threatening (grade 4) pulmonary embolism.

Hemorrhage

An increased incidence of bleeding events was observed in study patients treated with bevacizumab as compared to control treatment arms. The hemorrhagic events observed in bevacizumab studies were predominantly tumor-associated hemorrhage and minor mucocutaneous hemorrhage.

Patients with untreated CNS metastases were routinely excluded from clinical trials with bevacizumab, based on imaging procedures or signs and symptoms. Therefore, the risk of CNS hemorrhage in such patient has not been prospectively evaluated in randomized clinical studies.

Bevacizumab should be permanently discontinued for:

- grade 3 or 4 bleeding of any kind
- any grade of CNS bleeding. Patients should be monitored for signs and symptoms of CNS bleeding.

Bevacizumab should be permanently discontinued for grade ≥ 2 hemoptysis (defined as ≥ 2.5 mL bright red blood per episode). The safety of re-initiating bevacizumab in patients previously experiencing grade ≥ 2 hemoptysis has not been evaluated.

If hemorrhagic complications occur in patients on full dose anti-coagulation therapy, permanently discontinue bevacizumab treatment and follow guidelines of the treating institution. Standard procedures such as antagonisation with

protamin or vitamin K and infusion of vitamin K dependent factors should be considered dependent on the severity of the bleeding.

Hypersensitivity/Allergic Reactions and Infusion-Associated Reactions

Bevacizumab should be permanently discontinued in patients exhibiting hypersensitivity/allergic reactions.

The NCI CTCAE distinguish between hypersensitivity reactions and acute infusion reactions induced by cytokine release. Despite the different possible mechanisms underlying hypersensitivity and infusion reactions, the clinical signs and symptoms associated with these reactions overlap.

Patients may be at risk of developing infusion reactions to bevacizumab. Close observation of the patient during and following the administration of bevacizumab is recommended as expected for any infusion of a therapeutic humanised monoclonal antibody. If an infusion reaction occurs, the infusion should be discontinued and appropriate medical therapies should be administered. A systematic premedication is not warranted.

Osteonecrosis of the Jaw

Osteonecrosis of the jaw was reported in patients receiving bevacizumab mainly in combination with bisphosphonates in the post-marketing setting. The pathogenesis of the osteonecrosis is unclear. For further information, please refer to the Avastin® Investigator' Brochure.

Ovarian Failure

Ovarian failure has been reported more frequently in patients receiving bevacizumab. Ovarian function recovered in the majority of women after bevacizumab discontinuation. For further information, please refer to the Avastin® Investigator' Brochure.

4.4.4 Dose Modification Guidelines for nab-paclitaxel or paclitaxel (cohort 4)

Table 16 Dose Modification Guidelines for Nab-paclitaxel or paclitaxel (Cohort 4)

Toxicity Grade (CTCAE v.4.0)	Recommended Dose Modification
**Nab-paclitaxel** Grade 2 (Intolerable) or Grade ≥ 3	
1st occurrence	• Withhold until recovery to Grade 0-1<br>• Restart at a reduced dose of 80 mg/m^2
2nd occurrence	• Withhold until recovery to Grade 0-1<br>• Restart at a reduced dose of 60 mg/m^2
**Paclitaxel** Grade 2 (Intolerable) or Grade ≥ 3	
1st occurrence	• Withhold until recovery to Grade 0-1<br>• Restart at a reduced dose of 160 mg/m^2
2nd occurrence	• Withhold until recovery to Grade 0-1<br>• Restart at a reduced dose of 120 mg/m^2
3rd occurrence	• Withhold until recovery to Grade 0-1<br>• Restart at a reduced dose of 100 mg/m^2

4.5 STUDY TREATMENT DISCONTINUATION

Patients must discontinue study treatment if they experience any of the following:

- RPLS (reversible posterior leukoencephalopathy syndrome
- Intolerable toxicity related to study treatment, including development of an immune-mediated adverse event determined by the investigator to be unacceptable given the individual patient's potential response to therapy and severity of the event
- Any medical condition that may jeopardize the patient's safety if he or she continues study treatment
- Investigator or Sponsor determines it is in the best interest of the patient
- Use of another non-protocol anti-cancer therapy
- Pregnancy
- Symptomatic deterioration attributed to disease progression

  Radiographic disease progression per RECIST v1.1, (see Section 3.1 for exceptions

The primary reason for study treatment discontinuation should be documented on the appropriate CRF. This study will be utilizing the Prometheus database for data capture. Patients who discontinue study treatment prematurely will not be replaced. Patients will return to the clinic for a treatment discontinuation visit □ 30 days after the last dose of study treatment. The visit at which response assessment shows progressive disease may be used as the treatment discontinuation visit. Patients who discontinue study

treatment for any reason other than progressive disease or loss of clinical benefit will continue to undergo tumor response assessments as outlined in the schedule of activities.

Please refer to section 4.8.5 for survival follow-up assessments.

4.6 STUDY AND SITE DISCONTINUATION

Genentech has the right to terminate this study at any time. Reasons for terminating the study may include, but are not limited to, the following:

- The incidence or severity of AEs in this or other studies indicates a potential health hazard to patients.
- Patient enrollment is unsatisfactory.

Genentech will notify the investigator if Genentech decides to discontinue the study.

4.7 CLINICAL AND LABORATORY EVALUATIONS

4.7.1 <u>Pretreatment Evaluations</u>

The flowcharts of pretreatment study assessments are provided in Appendix 1.

4.7.2 <u>Study Assessments</u>

The flowcharts of scheduled study assessments are provided in Appendix 1. Patients will be closely monitored for safety and tolerability throughout the study. All assessments must be performed and documented for each patient.

Patients should be assessed for toxicity prior to each dose; dosing will occur only if the clinical assessment and local laboratory test values are acceptable.

If the timing of a protocol-mandated study visit coincides with a holiday and/or weekend that precludes the visit, the visit should be scheduled on the nearest following feasible date, with subsequent visits rescheduled accordingly.

4.7.2.1 Medical History

Medical history includes clinically significant diseases within the previous 5 years, smoking history, cancer history (including tumor characteristics such as hormone receptor status), prior cancer therapies and procedures, and all medications used by the patient within 7 days before the screening visit (including prescription, over-the-counter, and herbal/homeopathic remedies and therapies).

4.7.2.2 Vital Signs

Vital signs will include measurements of heart rate, respiratory rate, systolic and diastolic blood pressures while the patient is in a seated position, and temperature.

Please refer to Section 3.1 for details.

4.7.2.3 Physical Examination

A complete physical examination will be performed at screening and at the treatment discontinuation visit and should include the evaluation of head, eye, ear, nose, and throat and cardiovascular, dermatologic, musculoskeletal, respiratory, gastrointestinal , and neurologic systems.

A limited physical examination will be performed at other visits to assess changes from baseline abnormalities and any new abnormalities and to evaluate patient-reported symptoms. New or worsened abnormalities should be recorded as AEs if appropriate.

As part of tumor assessments, a physical examination should also include the evaluation of the presence and degree of enlarged lymph nodes, skin neoplasm, hepatomegaly, and splenomegaly.

All patients should be monitored for symptoms of brain metastases. Symptoms suggestive of new or worsening CNS metastases should prompt a full neurological examination. A CT or magnetic resonance imaging (MRI) scan of the head should be done as clinically indicated to confirm or refute new or worsening brain involvement.

These examinations may be done via telemedicine. A follow-up in person with a physician will be done if clinically indicated.

4.7.2.4 Tumor and Response Evaluation

Any evaluable or measurable disease must be documented at screening and reassessed at each subsequent tumor evaluation. Tumor response evaluations will occur every 8 weeks for cohorts 1 and 2 and every 6 weeks for cohort 3 and 4 . For patients with measurable disease, response will be assessed by the in vestigator per RECIST v1.1 (see Appendix 4) and immune-related response criteria (irRC; see Appendix 5).

All patients will be evaluated for overall survival and progression -free survival. ATC patients do not have to have target lesions therefore response evaluations may not be possible. In these patients, responses will not be recorded. Progression-free survival endpoint will be met in these patients when death occurs, there is unequivocal progression of non-target lesions or there is appearance of new lesions that are either biopsy-proven or that are unequivocally considered to be new, active disease (whichever occurs first). All patients with PDTC and ATC patients with target lesions in cohorts 1 and 2 will have responses recorded every 8 weeks, and those in cohorts 3 and 4 every 6 weeks.

4.7.2.5 Laboratory Assessments

Samples for hematology, serum chemistries, coagulation, urinalysis, and the pregnancy test will be analyzed at the study site’s local laboratory.

Local laboratory assessments will include the following:

- Hematology (CBC, including RBC count, hemoglobin, hematocrit, WBC count with differential [neutrophils, eosinophils, lymphocytes, monocytes, basophils, and other cells], and platelet count)
- Serum chemistries (glucose, BUN, creatinine, sodium, potassium, magnesium, chloride, bicarbonate, calcium, phosphorus, total and direct bilirubin, ALT, AST, alkaline phosphatase, creatinine phosphokinase, uric acid, LDH, total protein, and albumin)
- Coagulation (aPTT, PT/INR, fibrinogen, D-dimer)
- TSH, free T4, thyroglobulin
- Pregnancy test (for women of childbearing potential, including women who have had a tubal ligation)
- Urinalysis (specific gravity, pH, glucose, protein, ketones, and blood)
- Hepatitis B virus (HBV) serology (HBsAg, hepatitis B core antibody), and HCV serology (anti-HCV) as clinically indicated
  - HBV DNA test is required for patients who have known positive serology for anti-HBc

  HCV RNA test is required for patients who have known positive serology for anti-HCV
- Biomarker assays
- Archival tumor tissue sample
  - Archival tumor tissue samples obtained outside of this study for other purposes will be collected, if available, from all patients (paraffin blocks are preferred, and at least 4 unstained slides are acceptable). The tissue will be used for evaluating PD-L1 status by IHC.
- Tumor biopsy at screening (this timepoint is optional), on cycle 2 day 1(+2/-5 days) and at the time of initial radiographic progression, or at any surgical or biopsy intervention
  - Acceptable samples include core needle biopsies for deep tumor tissue or lymph nodes or excisional, incisional, punch, or forceps biopsies for cutaneous, subcutaneous, or mucosal lesions. For core needle biopsy specimens, at least five cores should be submitted for evaluation.
- Blood samples at screening, on cycle 2 day 1(+2/-5 days) and at the time of initial radiographic progression
  - 64 ml of blood will be collected at several time points for: a) isolation of germ-line DNA from peripheral mononuclear blood cells (PMBCs); b) isolation of plasma for cell free DNA analysis of genomic abnormalities using gene panels; c) potential flow cytometry analyses for phenotypic and functional studies , and potential liquid biopsy studies.

Blood samples (50cc) for PKs will be drawn on D15 of Run-in for cohort 1 patients or C1D15 for cohort 2 patients that are administered cobimetinib suspension and/or crushed vemurafenib. Along with documenting the timing of the sample collection, it is required to document the date and time of administration for the last dose of cobimetinib and/or vemurafenib prior to sample collection as well as the date and time of the administration of the initial dose on Day 1.

Refer to Appendix 12 for additional details on laboratory assessments and sample handling.

4.7.2.6 Outside Physician Participation During Treatment

- MDACC physician communication with the outside physician is required prior to the patient returning to the local physician. This will be documented in the patient record.
- A letter to the local physician outlining the patient's participation in a clinical trial will request local physician agreement to supervise the patient's care (Appendix G)
- Protocol required evaluations outside MDACC will be documented by telephone, fax, or email. Fax and/or e-mail will be dated and signed by the MDACC physician, indicating that they have reviewed it.
- Changes in drug dose and/or schedule must be discussed with and approved by the MDACC physician investigator, or their representative prior to initiation, and will documented in the patient record.
- A copy of the informed consent, protocol abstract, treatment schema and evaluation during treatment will be provided to the local physician.
- Documentation to be provided by the local physician will include drug administration records, progress notes, reports of protocol required laboratory and diagnostic studies and documentation of any hospitalizations.
- The home physician will be requested to report to the MDACC physician investigator all life threatening events within 24 hours of documented occurrence.
- Patients in Cohort 4 will return to MDACC every 21 days for evaluation. Patients receiving induction treatment administration will return to MDACC to begin their cohort-specific treatment administration.

4.7.3 <u>Specialized Evaluations Required for Safety Monitoring</u>

4.7.3.1 LVEF Assessment

All patients in Cohorts 1 and 2 will undergo evaluation of left ventricular dysfunction by ECHO at screening. Further, evaluation of LVEF by ECHO must be performed at C2D1, and then every 3 months until discontinuation of cobimetinib.

In patients restarting cobimetinib after a dose reduction or interruption after experiencing a decrease in LVEF, evaluate LVEF at approximately 2 weeks, 4 weeks, 10weeks, and 16 weeks, and then as clinically indicated.

4.7.3.2 Prolongation of QT Interval

Due to risk of QT interval prolongation with vemurafenib, all patients in Cohort 1 will undergo ECG per the schedule of assessments outlined in Appendix 1.

4.7.3.3 Secondary Malignancies

Due to risk of cutaneous squamous cell carcinoma, all patients in Cohort 1 will undergo complete dermatologic evaluation on screening, Day 1 of Cycle 1 and every three cycles thereafter during treatment (i.e., Day 1 of Cycles 4, 7, 10, etc.), at the treatment discontinuation visit (unless performed within the previous 12 weeks), 6 months (□ 2 weeks) after the last dose of study treatment, and as clinically indicated.

4.7.3.4 Dermatologic Exam

See section 4.7.3.3. In addition, all cohorts should undergo a dermatologic exam as clinically indicated due to potential dermatologic toxicity associated with atezolizumab. These examinations may be done via telemedicine. A follow-up in person with a physician will be done if clinically indicated.

4.7.3.5 Ophthalmologic Exam

All patients in Cohorts 1 and 2 should undergo a complete ophthalmologic examination, at screening, C2D1, then every 3rd course, then day 1 of cycles 15 and beyond (every 4 treatment cycles), and as clinically indicated if patients reports new or worsening visual disturbances. After the screening visit, follow up evaluations may be performed by telemedicine video visits by an ophthalmologist. If ocular side effects (including blurred vision) are noted on telemedicine visit during the study, it is recommended that an evaluation by an ophthalmologist be performed in clinic and additional tests conducted as clinically warranted. In particular, if a patient were to present signs and symptoms suggestive of keratitis (such as eye inflammation, lacrimation, light sensitivity, blurred vision, eye pain and/or red eye), a prompt ophthalmologist consult is required.

Using the Amsler Grid in the Eyehandbook app examinations of the retinal will be performed. Amsler grid will be used to detect early signs of serous retinopathy, CRVO, retinal edema. Amsler Grid technique is already being used by Ophthalmology for screening and monitoring of Macular/retinal diseases. Snellen vision screening on the Eyehandbook app along with external eye exam including the extraocular movement and confrontational visual field will be done. These modalities along with the patient's symptoms, if any, will provide indication of iritis, uveitis, conjunctivas, and dry eyes. The color vision in the Eyehandbook app will be used to detect optic nerve deterioration.

4.7.4 <u>Treatment Discontinuation Visit</u>

Patients who discontinue from treatment will be asked to return to the clinic no more than 30 days after the last treatment for a treatment discontinuation visit. The visit at which a response assessment shows progressive disease may be used as the treatment discontinuation visit.

4.7.5 Follow-Up Assessments

All patients who discontinue treatment without objective disease progression will be followed up with tumor assessment every 3 months until disease progression or the start of a different anti-cancer therapy.

Survival follow-up via phone call or visit with physician will occur every 3 months for the first 2 years once they are off study treatment, then every 6 months during Years 3 and 4, and yearly thereafter until patient death, withdrawal of consent, patient is lost to follow-up, or study termination by Genentech, whichever occurs first..

4.7.6 Post-Treatment Evaluations

Tumor assessments (cross-sectional imaging by CT, MRI or PET/CT) will be performed every 3 months until disease progression or start of a different anti-cancer therapy. Imaging may be performed locally if images can be transferred or mailed to the principal investigator's site for evaluation.

Female patients of reproductive potential who are not surgically sterile must practice adequate birth control for a minimum of 6 months post-treatment. Male patients who are not surgically sterile must practice adequate birth control for a minimum of 6 months post-treatment.

5. STATISTICAL CONSIDERATIONS

5.1 DETERMINATION OF SAMPLE SIZE

For the primary endpoint the sample size will be a minimum of 36 ATC patients. The total number of ATC + PDTC patients will be 60, however a minimum of 36 ATC patients are required to be enrolled in order to evaluate the primary endpoint.

Sample Size Justification

For N=36, with a 33 month accrual period and 3 month post accrual follow-up, the power is 90% to detect an observed 9 month median OS vs. a historical 5 month OS with a one-sided 5% alpha. The power is 79% to detect an observed 8 month median OS and 70% for 7.5 month median OS. Calculations were done using the DSTPLAN program.

5.2 PLANNED EFFICACY EVALUATIONS

Primary endpoint: To determine if targeted therapy + atezolizumab (cohorts 1-3) will lead to improved overall survival (OS) in patients with anaplastic thyroid carcinoma (ATC). Only ATC patients who are enrolled in cohorts with targeted therapy (1-3) will be analyzed for the primary efficacy endpoint.

Secondary endpoints:

1. To evaluate the efficacy (RECIST and irRC response rate, progression-free survival [PFS]) of targeted therapy + atezolizumab (cohorts 1-3) in ATC and poorly differentiated thyroid cancer (PDTC).

2. To determine the OS in patients with PDTC treated with targeted therapy + atezolizumab (cohorts 1-3).

3. To determine the efficacy (RECIST/irRC response rate, progression-free survival [PFS]) and OS of ATC and PDTC patients treated with taxanes + atezolizumab (cohort 4)

4. To evaluate the efficacy and OS of each targeted therapy cohort (1-3) separately and in each disease group (ATC and PDTC).

5.3 PRIMARY EFFICACY VARIABLES

Overall survival in ATC patients who are enrolled on cohorts 1-3

5.4 SECONDARY EFFICACY VARIABLES

Progression-free survival, objective response rate (CR/PR) in ATC and PDTC patients (all cohorts).

Patients who become resectable while on clinical trial will be analyzed separately for overall survival and progression-free survival.

5.5 METHOD OF ANALYSIS

Analysis Plan

Overall survival is defined as the time from start date of cohort specific treatment to death from any cause will be estimated using the Kaplan-Meier method. Progression-free survival is defined as the time from start date of cohort specific treatment to progression or death (whichever occurs first) will be estimated using the Kaplan-Meier method. Median and probabilities at selected times will be estimated with 95% confidence intervals. Objective response (CR/PR) rate will be estimated along with exact binomial 95% confidence interval

Sample size = 66 total (minimum of 36 ATC patients for primary and secondary objectives and no more than 20 PDTC for secondary objectives)

5.6 FUTILITY MONITORING PLAN

All patients who receive at least 1 dose of study drug (vemurafenib, cobimetinib, bevacizumab, atezolizumab) in any cohort will be included in futility monitoring. Cohorts 1-3 will be included in one futility monitoring plan. We will monitor time to death continuously using a Bayesian method (Thall et al, 2005, Clinical Trials, 2:467-478) that assumes the median time to death follows an Inverse Gamma distribution and that the individual death times follow an exponential distribution. Let mE represent the median in the newly accrued (experimental) patients and let mS represent the historical median to be used for comparison. Based on somewhat limited historical data the median death time is about 5 months. For the historical median we specify an Inverse Gamma prior with alpha = 8.25 and beta = 36.25 (which has mean = 5.0 and SD = 2.0). For the experimental median we specify an Inverse Gamma prior with alpha = 2.25 and beta = 6.25 (which has a mean of 5.0 and SD = 10.0). The maximum sample size is 36 and the expected accrual rate is 1.1 per month (trial duration = 33

months). There will be 3 months of post-accrual follow-up. Monitoring will be continuous with the rule to stop if Pr(mE > mS + 4 | data) < 0.025. The operating characteristics were computed using One-Arm Time-to-Event Simulator version 3.0.2. The rule will be implemented using the Department of Biostatistics Clinical Trial Conduct website.

Operating Characteristics

Median (m)	PET (%)	Ave # Pts	Ave Dur. (m)
3.0	100%	7.4	9.7
5.0	85%	17.0	18.4
7.0	34%	27.8	27.6
9.0	15%	31.8	31.3
110	8%	33.6	33.0
13.0	5%	34.5	33.5

Where PET = probability of early termination
Dur. = duration of trial

A separate futility monitoring plan will be used for cohort 4. We will monitor time to death continuously using a Bayesian method (Thall et al, 2005, Clinical Trials, 2:467 - 478) that assumes the median time to death follows an Inverse Gamma distribution and that the individual death times follow an exponential distribution. Let mE represent the median in the newly accrued (experimental) patients and let mS represent the historical median to be used for comparison. Based on somewhat limited historical data the median death time is about 3 months. For the historical median we specify an Inverse Gamma prior with alpha = 4.25 and beta = 9.75 (which has mean = 3.0 and SD = 2.0). For the experimental median we specify an Inverse Gamma prior with alpha = 2.09 and beta = 3.27 (which has a mean of 3.0 and SD = 10.0). The maximum sample size is 36 and the expected accrual rate is 0.5 per month (trial duration = 28 months). There will be 3 months of post-accrual follow-up. Monitoring will be continuous with the rule to stop if Pr(mE > mS + 2 | data) < 0.025. The operating characteristics were computed using One-Arm Time-to-Event Simulator version 3.0.2. The rule will be implemented using the Department of Biostatistics Clinical Trial Conduct website.

Operating Characteristics

Median (m)	PET (%)	Ave # Pts	Ave Dur. (m)
1.0	100%	3.6	10.2
2.0	80%	7.5	18.0
3.0	33%	11.4	24.2
4.0	17%	12.5	26.5
5.0	8%	13.2	27.8
6.0	5%	13.5	28.0

Where PET = probability of early termination
Dur. = duration of trial

6. ASSESSMENT OF SAFETY

1. To evaluate the safety of targeted therapy + atezolizumab in cohorts 1-3.

2. To evaluate the safety of taxanes + atezolizumab in cohort 4.

All patients who receive at least 1 dose of study drug (vemurafenib, cobimetinib, bevacizumab, atezolizumab) in any cohort will be eligible for safety outcome, and will be monitored for toxicity. We will stop accrual and reevaluate dosing if the proportion of patients with grade 4 or 5 toxicities exceeds 20%.

Safety assessments will consist of monitoring and reporting AEs and SAEs that are considered related to atezolizumab, vemurafenib, cobimetinib, bevacizumab, nab-paclitaxel, paclitaxel, all events of death, and any study-specific issue of concern. The monitoring rule will start once 3 patients have accrued and will occur through cycle 2. Toxicity monitoring occur within each treatment cohort.

6.1 RISKS ASSOCIATED WITH ATEZOLIZUMAB

Atezolizumab has been associated with risks such as the following: IRRs and immune-related hepatitis, pneumonitis, colitis, pancreatitis, diabetes mellitus, hypothyroidism, hyperthyroidism, adrenal insufficiency, hypophysitis, Guillain-Barré syndrome, myasthenic syndrome or myasthenia gravis, meningoencephalitis, myocarditis, nephritis, myositis, and severe cutaneous adverse reactions. Immune-mediated reactions may involve any organ system and may lead to hemophagocytic lymphohistiocytosis and macrophage activation syndrome. In addition, systemic immune activation (described below) is a potential risk when atezolizumab is given in combination with other immunomodulating agents.
Refer to Section 6 of the Atezolizumab Investigator's Brochure for a detailed description of anticipated safety risks for atezolizumab.

Systemic immune activation is a rare condition characterized by an excessive immune response. Given the mechanism of action of atezolizumab, systemic immune activation is considered a potential risk when given in combination with other immunomodulating agents. Systemic immune activation should be included in the differential diagnosis for patients who, in the absence of an alternative etiology, develop a sepsis-like syndrome after administration of atezolizumab, and the initial evaluation should include the following:

- CBC with peripheral smear
- PT, PTT, fibrinogen, and D-dimer
- Ferritin
- Triglycerides
- AST, ALT, and total bilirubin
- LDH

- Complete neurologic and abdominal examination (assess for hepatosplenomegaly)

If systemic immune activation is still suspected after the initial evaluation, contact the Medical Monitor for additional recommendations.

6.2 SAFETY PARAMETERS AND DEFINITIONS

6.2.1 Adverse Events

An Adverse Event is defined as any untoward medical occurrence in a patient regardless of its causal relationship to study treatment. An AE can be any unfavorable and unintended sign (including any clinically significant abnormal laboratory test result), symptom, or disease temporally associated with the use of the study treatment, whether or not it is considered to be study drug(s) related. Included in this definition are any newly occurring events and any previous condition that has increased in severity or frequency since the administration of study.

Attribution - the determination of whether an adverse event is related to a medical treatment or procedure.

Definite - the adverse event is clearly related to the investigational agent(s).

Probable - the adverse event is likely related to the investigational agent(s).

Possible - the adverse event may be related to the investigational agent(s).

Unlikely - The adverse event is doubtfully related to the investigational agent(s).

Unrelated - The adverse event is clearly NOT related to the investigational agent(s).

Recommended Adverse Event Recording Guidelines

Attribution	Grade 1	Grade 2	Grade 3	Grade 4	Grade 5
Unrelated	Phase I	Phase I	Phase I<br>Phase II	Phase I<br>Phase II<br>Phase III	Phase I<br>Phase II<br>Phase III
Unlikely	Phase I	Phase I	Phase I<br>Phase II	Phase I<br>Phase II<br>Phase III	Phase I<br>Phase II<br>Phase III
Possible	Phase I<br>Phase II	Phase I<br>Phase II<br>Phase III	Phase I<br>Phase II<br>Phase III	Phase I<br>Phase II<br>Phase III	Phase I<br>Phase II<br>Phase III
Probable	Phase I<br>Phase II	Phase I<br>Phase II<br>Phase III	Phase I<br>Phase II<br>Phase III	Phase I<br>Phase II<br>Phase III	Phase I<br>Phase II<br>Phase III
Definitive	Phase I<br>Phase II	Phase I<br>Phase II<br>Phase III	Phase I<br>Phase II<br>Phase III	Phase I<br>Phase II<br>Phase III	Phase I<br>Phase II<br>Phase III

The investigator (or physician designee) is responsible for verifying and providing source documentation for all adverse events and assigning the attribution for all adverse events for subjects enrolled.

6.2.2 Adverse Event (AE) Severity

Severity of the adverse events (AEs) -The severity of the adverse events (AEs) will be graded according to the National Cancer Institute Common Terminology Criteria for Adverse Events (NCI CTCAE) 5.0. Events not included in the NCI CTCAE will be scored as follows:

General grading:

- Grade 1: Mild: discomfort present with no disruption of daily activity, no treatment required beyond prophylaxis.
- Grade 2: Moderate: discomfort present with some disruption of daily activity, require treatment.
- Grade 3: Severe: discomfort that interrupts normal daily activity, not responding to first line treatment.
- Grade 4: Life Threatening: discomfort that represents immediate risk of death

6.2.3 Serious Adverse Event (SAE) Reporting Requirements for M D Anderson Sponsor Single Site IND Protocols

An adverse event or suspected adverse reaction is considered “serious” if, in the view of either the investigator or the sponsor, it results in any of the following outcomes:

- Death
- A life-threatening adverse event
- Inpatient hospitalization or prolongation of existing hospitalization
- A persistent or significant incapacity or substantial disruption of the ability to conduct normal life functions.
- A congenital anomaly/birth defect.

Important medical events that may not result in death, be life-threatening, or require hospitalization may be considered a serious adverse drug experience when, based upon appropriate medical judgment, they may jeopardize the patient or subject and may require medical or surgical intervention to prevent one of the outcomes listed in this definition. Examples of such medical events include allergic bronchospasm requiring intensive treatment in an emergency room or at home, blood dyscrasias or convulsions that do not result in

inpatient hospitalization, or the development of drug dependency or drug abuse (21 CFR 312.32).

- Important medical events as defined above, may also be considered serious adverse events. Any important medical event can and should be reported as an SAE if deemed appropriate by the Principal Investigator or the IND Sponsor, IND Office.
- All events occurring during the conduct of a protocol and meeting the definition of a SAE must be reported to the IRB in accordance with the timeframes and procedures outlined in "The University of Texas M. D. Anderson Cancer Center Institutional Review Board Policy on Reporting Adverse Events for Drugs and Devices".
- Serious adverse events will be captured from the time of the first protocol-specific intervention, until 90 days after the last dose of drug unless the participant withdraws consent.
- Serious adverse events must be followed until clinical recovery is complete and laboratory tests have returned to baseline, progression of the event has stabilized, or there has been acceptable resolution of the event.
- All SAEs, expected or unexpected/ initial or follow up, must be reported to the IND Office within 5 working days of knowledge of the event regardless of the attribution.
- Death or life-threatening events that are unexpected, possibly, probably or definitely related to drug must be reported (initial or follow up) to the IND Office within 24 hours of knowledge of the event.
- Additionally, any serious adverse events that occur after the 90 day time period that are related to the study treatment must be reported to the IND Office. This may include the development of new malignancies.
- The electronic SAE application (eSAE) will be utilized for safety reporting to the IND Office and MD Anderson IRB.
- All events reported to the supporting company must also be reported to the IND Office

Reporting to FDA:

Serious adverse events will be forwarded to FDA by the IND Sponsor according to 21 CFR 312.32.

It is the responsibility of the PI and the research team to ensure serious adverse events are reported according to the Code of Federal Regulations, Good Clinical Practices, the protocol guidelines, the sponsor's guidelines, and Institutional Review Board policy.

6.3 METHODS AND TIMING FOR ASSESSING AND RECORDING SAFETY VARIABLES

6.3.1 The investigator is responsible for ensuring that all AEs and SAEs that are observed or reported during the study are collected and reported to Genentech, Inc., in accordance with

CFR 312.32 (Investigational New Drug [IND] Safety Reports). Adverse Event Reporting Period

The study period during which all AEs and SAEs must be reported begins after informed consent is obtained and initiation of study treatment and ends 90 days following the last administration of study treatment or study discontinuation/termination, whichever is earlier. After this period, investigators should only report SAEs that are attributed to prior study treatment.

6.3.2 Assessment of Adverse Events

All AEs and SAEs, whether volunteered by the patient, discovered by study personnel during questioning, or detected through physical examination, laboratory test, or other means, will be reported appropriately. Each reported AE or SAE will be described by its duration (i.e., start and end dates), regulatory seriousness criteria if applicable, suspected relationship to the study drugs (see following guidance), and actions taken.

To ensure consistency of AE and SAE causality assessments, investigators should apply the following general guideline:

Yes **(definitive, probable, possible, unlikely)**

There is a plausible temporal relationship between the onset of the AE and administration of any study drug, and the AE cannot be readily explained by the patient's clinical state, intercurrent illness, or concomitant therapies; and/or the AE follows a known pattern of response to any study drug; and/or the AE abates or resolves upon discontinuation of any study drug or dose reduction and, if applicable, reappears upon re-challenge.

No(unrelated)

Evidence exists that the AE has an etiology other than any study drug (e.g., pre-existing medical condition, underlying disease, intercurrent illness, or concomitant medication); and/or the AE has no plausible temporal relationship to any study drug administration (e.g., cancer diagnosed 2 days after first dose of study drug).

Expected AEs are those AEs that are listed or characterized in the Package Insert (PI) or current Investigator's Brochure.

Unexpected AEs are those not listed in the PI or current Investigator's Brochure or not identified. This includes AEs for which the specificity or severity is not consistent with the description in the PI or Investigator's Brochure. For example, under this definition, hepatic necrosis would be unexpected if the PI or Investigator's Brochure only referred to elevated hepatic enzymes or hepatitis.

6.4 PROCEDURES FOR ELICITING, RECORDING, AND REPORTING ADVERSE EVENTS

6.4.1 Eliciting Adverse Events

A consistent methodology for eliciting AEs at all patient evaluation time points should be adopted. Examples of non-directive questions include:

- "How have you felt since your last clinical visit?"
- "Have you had any new or changed health problems since you were last here?"

6.4.2 Specific Instructions for Recording Adverse Events

Investigators should use correct medical terminology/concepts when reporting AEs or SAEs. Avoid colloquialisms and abbreviations.

6.4.2.1 Diagnosis versus Signs and Symptoms

If known at the time of reporting, a diagnosis should be reported rather than individual signs and symptoms (e.g., record only liver failure or hepatitis rather than jaundice, asterixis, and elevated transaminases). However, if a constellation of signs and/or symptoms cannot be medically characterized as a single diagnosis or syndrome at the time of reporting, it is acceptable to report the information that is currently available. If a diagnosis is subsequently established, it should be reported as follow-up information.

6.4.2.2 Deaths

All deaths that occur during the protocol-specified AE reporting period (see Section 6.3.1), regardless of attribution, will be reported to the appropriate parties. When recording a death, the event or condition that caused or contributed to the fatal outcome should be reported as the single medical concept. If the cause of death is unknown and cannot be ascertained at the time of reporting, report "Unexplained Death." Deaths that occur during the protocol-specified adverse event reporting period (see Section 6.4.2.12) that are attributed by the investigator solely to progression of disease should be recorded only in the study eCRF.

6.4.2.3 Pre-existing Medical Conditions

A pre-existing medical condition is one that is present at the start of the study. Such conditions should be reported as medical and surgical history. A pre-existing medical condition should be re-assessed throughout the trial and reported as an AE or SAE only if the frequency, severity, or character of the condition worsens during the study. When reporting such events, it is important to convey the concept that the pre-existing condition has changed by including applicable descriptors (e.g., "more frequent headaches").

6.4.2.4 Hospitalizations for Medical or Surgical Procedures

Any AE that results in hospitalization or prolonged hospitalization should be documented and reported as an SAE. If a patient is hospitalized to undergo a medical or surgical procedure as a result of an AE, the event responsible for the procedure, not

the procedure itself, should be reported as the SAE. For example, if a patient is hospitalized to undergo coronary bypass surgery, record the heart condition that necessitated the bypass as the SAE.

Hospitalizations for the following reasons do not require reporting:

- Hospitalization or prolonged hospitalization for diagnostic or elective surgical procedures for pre-existing conditions,
- Hospitalization or prolonged hospitalization required to allow efficacy measurement for the study, or
- Hospitalization or prolonged hospitalization for scheduled therapy of the target disease of the study.

6.4.2.5 Pregnancies in Female Patients

Female patients of childbearing potential will be instructed to immediately inform the investigator if they become pregnant during the study or within 6 months after the last dose of study drug. A Pregnancy Report CRF should be completed by the investigator immediately (i.e., no more than 24 hours after learning of the pregnancy) and submitted via fax. A pregnancy report will automatically be generated and sent to Genentech Drug Safety. Pregnancy should not be recorded on the Adverse Event CRF. The investigator should discontinue study drug and counsel the patient, discussing the risks of the pregnancy and the possible effects on the fetus. Monitoring of the patient should continue until conclusion of the pregnancy. Any SAEs associated with the pregnancy (e.g., an event in the fetus, an event in the mother during or after the pregnancy, or a congenital anomaly/birth defect in the child) should be reported on the Adverse Event CRF.

A Clinical Trial Pregnancy Reporting Form and fax cover sheet should be completed and faxed to Genentech Drug Safety or its designee immediately (i.e., no more than 24 hours after learning of the pregnancy), using the fax numbers provided to investigators (see "Protocol Administrative and Contact Information & List of Investigators").

6.4.2.6 Pregnancies in Female Partners of Male Patients

Male patients will be instructed through the ICF to immediately inform the investigator if their partner becomes pregnant during the study or within 6 months after completing treatment with atezolizumab, cobimetinib, bevacizumab, and/or vemurafenib. Male patients who received study treatment should not attempt to father a child until end of study . A Pregnancy Report CRF should be completed by the investigator immediately (i.e., no more than 24 hours after learning of the pregnancy) and faxed to Genentech Drug
Safety. Attempts should be made to collect and report details of the course and outcome of any pregnancy in the partner of a male patient exposed to study drug. The pregnant partner will need to sign an Authorization for Use and Disclosure of Pregnancy

Health Information to allow for follow-up on her pregnancy. Once the authorization has

been signed, the investigator will update the Pregnancy Report CRF with additional information on the course and outcome of the pregnancy. An investigator who is contacted by the male patient or his pregnant partner may provide information on the risks of the pregnancy and the possible effects on the fetus, to support an informed decision in cooperation with the treating physician and/or obstetrician.

6.4.2.7 Abortions

Any spontaneous abortion should be classified as an SAE (as Genentech consider spontaneous abortions to be medically significant events), recorded on the Adverse Event CRF, and reported to Genentech Drug Safety immediately (i.e., no more than 24 hours after learning of the event)

6.4.2.8 Congenital Anomalies/Birth Defects

Any congenital anomaly/birth defect in a child born to a female patient or female partner of a male patient exposed to study drug should be classified as an SAE, recorded on the Adverse Event CRF, and reported to Genentech Drug Safety immediately (i.e., no more than 24 hours after learning of the event)

6.4.2.9 Post-Study Adverse Events

The investigator should expeditiously report any SAE occurring after a patient has completed or discontinued study participation if attributed to prior exposure to any study drug. If the investigator should become aware of the development of cancer or a congenital anomaly in a subsequently conceived offspring of a female subject including pregnancy occurring in the partner of a male study subject who participated in the study, this should be reported as an SAE adequately to Genentech drug Safety during follow up period.

6.4.2.10 Safety Reconciliation (Case Transmission Verification of Single Case Reports)

The Investigator agrees to conduct reconciliation for the product. Genentech and the Investigator will agree to the reconciliation periodicity and format, but agree at minimum to exchange quarterly line listings of cases received by the other party.

If discrepancies are identified, the Investigator and Genentech will cooperate in resolving the discrepancies. The responsible individuals for each party shall handle the matter on a case-by-case basis until satisfactory resolution. The Investigator shall receive reconciliation guidance documents within the ‘Activation Package’.

Following Case Transmission Verification, single case reports which have not been received by Genentech shall be forwarded by the Sponsor to Genentech within five (5) calendar days from request by Genentech.

At the end of the study, a final cumulative Case Transmission Verification (CTV) report will be sent to Genentech.

6.4.2.11 Genentech-investigator agrees to conduct reconciliation for the product.

Genentech and Genentech-investigator will agree to the reconciliation periodicity and format, but agree at minimum to exchange quarterly line listings of cases received by the other party. If discrepancies are identified, Genentech-investigator and Genentech will cooperate in resolving the discrepancies. The responsible individuals for each party shall handle the matter on a case-by-case basis until satisfactory resolution.Adverse Events of Special Interest

Adverse events of special interest (AESIs) are defined as a potential safety problem, identified as a result of safety monitoring of the IMP.

The following AEs are considered of special interest and must be reported to the Genentech Drug Safety expeditiously (see Section 6.4.2.12 for reporting instructions), irrespective of regulatory seriousness criteria:

Adverse events of special interest are required to be reported by the investigator to Genentech immediately irrespective of regulatory seriousness criteria (i.e., no more

than 24 hours after learning of the event; see Appendix 8 for reporting instructions). Adverse events of special interest for this study are listed below.

<u>Adverse Events Specific to all study drugs:</u>

- Cases of potential drug-induced liver injury that include an elevated ALT or AST in combination with either an elevated bilirubin or clinical jaundice, as defined by Hy's Law and based on the following observations:
  - Treatment-emergent ALT or AST $> 3 \times$ ULN in combination with total bilirubin $> 2 \times$ ULN
  - Treatment-emergent ALT or AST $> 3 \times$ ULN in combination with clinical jaundice
- Data related to Suspected transmission of an infectious agent by the study drug (STIAMP), as defined below

  Any organism, virus, or infectious particle (e.g., prion protein transmitting transmissible spongiform encephalopathy), pathogenic or non-pathogenic, is considered an infectious agent. A transmission of an infectious agent may be suspected from clinical symptoms or laboratory findings that indicate an infection in a patient exposed to a medicinal product. This term applies <u>only</u> when a contamination of study treatment is suspected.

Events Specific to Atezolizumab

- Pneumonitis
- Colitis
- Endocrinopathies: diabetes mellitus, pancreatitis, adrenal insufficiency, hyperthyroidism, and hypophysitis
- Hepatitis, including AST or ALT $>$ 10 x ULN
- Systemic lupus erythematosus
- Neurological disorders: Guillain-Barré syndrome, myasthenic syndrome or myasthenia gravis, and meningoencephalitis

Events suggestive of hypersensitivity, infusion-related reactions, cytokine-release syndrome, influenza-like illness, and macrophage activating syndrome, hemophagocytic lymphohistiocytosis .

- Nephritis
- Ocular toxicities (e.g., uveitis, retinitis, optic neuritis)
- Myositis
- Myopathies, including rhabdomyolysis
- Grade $\geq$ 2 cardiac disorders (e.g., atrial fibrillation, myocarditis, pericarditis)
- Vasculitis
- Autoimmune hemolytic anemia

- Severe cutaneous reactions (e.g., Stevens-Johnson syndrome, dermatitis bullous, toxic epidermal necrolysis)

<u>Events Specific to Cobimetinib</u>

- Any grade retinal vein occlusion
- Any grade serous retinopathy, including events of retinal detachment, retinal pigment epithelium detachment, neurosensory retinal detachment or central serous chorioretinopathy
- Grade ≥ 3 photosensitivity
  - Rhabdomyolysis or Grade ≥ 3 CPK elevation
  - Grade ≥ 3 hemorrhage or any grade cerebral hemorrhage
- Grade ≥ 3 rash
  - Grade ≥ 3 diarrhea
- Grade ≥ 3 QT interval prolongation
  - Symptomatic heart failure and/or Grade ≥ 2 left ventricular dysfunction
  - Pneumonitis
  - Significant liver toxicity:
    AST and/or ALT > 10 x upper limit of normal

Events Specific to Vemurafenib

- Acute Kidney Injury
- Bone Marrow Toxicity
- Cutaneous Squamous Cell Carcinomas
- Gastrointestinal Polyps
- Hypersensitivity and Severe Cutaneous Reactions
- Liver Injury
- New/Second Primary Melanomas
- Non cutaneous Squamous Cell Carcinomas
- Pancreatitis
- Photosensitivity
- Potentiation of Radiation Toxicity
- Progression of RAS Mutant Malignancies
- QT Prolongation
- Retinal Vein Occlusion
- Uveitis
- VII Nerve Paralysis

<u>Events Specific to Bevacizumab</u>

Hypertension ≥ grade 3

Proteinuria ≥ grade 3
GI perforation, abscesses and fistulae (any grade)
Wound healing complications =/> grade 3
Haemorrhage ≥ grade 3 (any grade CNS bleeding; > grade 2 haemoptysis)
Arterial thromboembolic events (any grade)
Venous thromboembolic events ≥ grade 3
Posterior reversible encephalopathy syndrome (PRES - any grade)
CHF ≥ grade 3
Non-GI fistula or abscess ≥ grade 2

4403

6.4.2.12 Adverse Event Reporting

Investigators must report all SAEs to Genentech within the timelines described below. In addition to recording in the IND eSAE database, the completed MD Anderson Cancer Center IND Office internal electronic serious adverse event (eSAE) reporting form should be sent immediately upon completion to Genentech Drug Safety at:

Sponsor-Investigator will be responsible for collecting all protocol-defined Adverse Events (AEs)/Serious Adverse Events (SAEs), AEs of Special Interest (AESIs), Special Situation Reports (including pregnancy reports) and Product Complaints (with or without an AE) originating from the Study for the Product.

All protocol-defined AEs, SAEs, AESIs, Special Situation Reports (including pregnancy reports) and Product Complaints *with* an AE should be sent to:

E-mail: usds_aereporting-d@gene.com

OR

Fax: (650) 238-6067

All Product Complaints *without* an AE should be sent to:

Phone: 800-334-0290 (M-F: 5 am to 5 pm PST)

Relevant follow-up information should be submitted to Genentech Drug Safety as soon as it becomes available.

SAE and AESI reports, whether related or unrelated to atezolizumab, cobimetinib, vemurafenib and/or bevacizumab, will be transmitted to Genentech within 24 hours of the Awareness Date.

Additional reporting requirements to Genentech include the following:

- Any reports of pregnancy following the start of administration with the atezolizumab, cobimetinib, vemurafenib and/or bevacizumab and within the follow-up period (for female subjects within 6 months after the last dose of atezolizumab,

cobimetinib, vemuraf enib and/or bevacizumab or the partner of a male subject within three months of completing therapy) will be transmitted to Genentech within 24 hours of the Awareness Date.

- All non-serious atezolizumab, cobimetinib, vemurafenib and/or bevacizumab AEs originating from the study will be forwarded Genentech quarterly.

Note: Investigators should also report events to their IRB as required.

Follow-Up Information

Additional information may be added to a previously submitted report by any of the following methods:

- Adding to the original MD Anderson Cancer Center internal eSAE reporting form and submitting it as follow-up
- Adding supplemental summary information and submitting it as follow-up with the original MD Anderson Cancer Center internal eSAE reporting form
- Summarizing new information and faxing it with a cover letter including patient identifiers (i.e., date of birth, initial, patient number), protocol description and number, if assigned, brief AE description, and notation that additional or follow-up information is being submitted. (The patient identifiers are important so that the new information is added to the correct initial report.)

Occasionally Genentech may contact the reporter for additional information, clarification, or current status of the patient for whom and AE was reported. Relevant follow-up information should be submitted to Genentech Drug Safety as soon as it becomes available and/or upon request.

- **<u>Other Special Situation Reports</u>**

  In addition to all SAEs, pregnancy reports and AESIs, the following other Special Situations Reports should be collected even in the absence of an Adverse Event and transmitted to Genentech within thirty (30) calendar days:

  - Data related to the Product usage during breastfeeding
  - Data related to overdose, abuse, misuse or medication error (including potentially exposed or intercepted medication errors)
  - In addition, reasonable attempts should be made to obtain and submit the age or age group of the patient, in order to be able to identify potential safety signals specific to a particular population

- **<u>Product Complaints</u>**

All Product Complaints (with or without an AE) shall be forwarded to Genentech within fifteen (15) calendar days of the awareness date.

A Product Complaint is defined as any written or oral information received from a complainant that alleges deficiencies related to identity, quality, safety, strength, purity, reliability, durability, effectiveness, or performance of a product after it has been released and distributed to the commercial market or clinical trial.

Follow-Up Information

Additional information may be added to a previously submitted report by any of the following methods:

- Adding to the original MD Anderson Cancer Center internal eSAE reporting form and submitting it as follow-up
- Adding supplemental summary information and submitting it as follow-up with the original MD Anderson Cancer Center internal eSAE reporting form
- Summarizing new information and faxing it with a cover letter including patient identifiers (i.e., date of birth, initial, patient number), protocol description and number, if assigned, brief AE description, and notation that additional or follow-up information is being submitted. (The patient identifiers are important so that the new information is added to the correct initial report.)

Occasionally Genentech may contact the Investigator for additional information, clarification, or current status of the patient for whom and AE was reported. Relevant follow-up information should be submitted to Genentech Drug Safety as soon as it becomes available and/or upon request.

6.4.3 Additional Reporting Requirements for IND

For investigator-sponsored IND studies, some additional reporting requirements for the FDA apply in accordance with the guidance set forth in 21 CFR § 600.80.

Events meeting the following criteria need to be submitted to the FDA as expedited IND Safety Reports according to the following guidance and timelines:

7 Calendar Day Telephone or Fax Report

The investigator is required to notify the FDA of any fatal or life-threatening AE that is unexpected and assessed by the investigator to be possibly related to the use of atezolizumab, cobimetinib, vemurafenib and/or bevacizumab. An unexpected AE is one that is not already described in the atezolizumab, cobimetinib, vemurafenib and/or bevacizumab Investigator's Brochure. Such reports are to be telephoned or faxed to the FDA and Genentech within 7 calendar days of first learning of the event.

15 Calendar Day Written Report

The Investigator is also required to notify the FDA and all participating investigators, in a written IND Safety Report, of any serious, unexpected AE that is considered reasonably or possibly related to the use of study drugs. An unexpected AE is one that is not already described in the atezolizumab, cobimetinib, vemurafenib and/or bevacizumab Investigator's Brochure.

Written IND Safety reports should include an Analysis of Similar Events in accordance with regulation 21 CFR § 312.32. All safety reports previously filed by the investigator with the IND concerning similar events should be analyzed and the significance of the new report in light of the previous, similar reports commented on.

Written IND safety reports with analysis of similar events are to be submitted to the FDA, Genentech, and all participating investigators within 15 calendar days of first learning of the event. The FDA prefers these reports on a MedWatch 3500 form, but alternative formats are acceptable (e.g., summary letter).

Contact Information for IND Safety Reports

FDA fax number for IND safety reports:

Fax: (800) FDA-0178

All written IND safety reports submitted to the FDA by the investigator must also be sent to the following:

Fax: (650) 225-4682 or (650) 225-4630
Site's IRB: Dr. Aman Buzdar 713-792-2933
Email: usds_aereporting-d@gene.com
And Sponsor-Investigator will be responsible for the distribution of safety information to Site IRB.

For questions related to safety reporting, please contact Genentech Drug Safety:

Tel: (888) 835-2555
Fax: (650) 225-4682 or (650) 225-4630

6.4.4 IND Annual Reports

Copies of all IND annual reports submitted to the FDA by the IND Sponsor-investigator should be sent to Genentech Drug Safety.

Copies of such reports should be emailed to Genentech at: Genentech Drug Safety CTV mail box: ctvist_drugsafety@gene.com

Other Reports

The IND Sponsor will forward a copy of the Publication to Genentech upon completion of the Study

6.5 STUDY CLOSE-OUT

Any study report submitted to the FDA by the Genentech-investigator should be copied to Genentech. This includes all IND annual reports and the Clinical Study Report (final study report). Additionally, any literature articles that are a result of the study should be sent to Genentech. Copies of such reports should be mailed to the assigned Clinical Operations contact for the study:

atezolizumab Protocols

Email: anti-pdl-1-mpd3280a-gsur@gene.com

And to Genentech Drug Safety CTV oversight mail box at: ctvist_drugsafety@gene.com

QUERIES

Queries related to the Study will be answered by the Sponsor. However, responses to all safety queries from regulatory authorities or for publications will be discussed and coordinated between the Parties. The Parties agree that Genentech shall have the final say and control over safety queries relating to the Product. The Sponsor agrees that it shall not answer such queries from regulatory authorities and other sources relating to the Product independently but shall redirect such queries to Genentech.

Both Parties will use all reasonable effort to ensure that deadlines for responses to urgent requests for information or review of data are met. The Parties will clearly indicate on the request the reason for urgency and the date by which a response is required.

SAFETY CRISIS MANAGEMENT

In case of a safety crisis, e.g., where safety issues have a potential impact on the indication(s), on the conduct of the Study, may lead to labeling changes or regulatory actions that limit or restrict the way in which the Product is used, or where there is media involvement, the Party where the crisis originates will contact the other Party as soon as possible.

The Parties agree that Genentech shall have the final say and control over safety crisis management issues relating to the Product. The Sponsor agrees that it shall not answer such queries from media and other sources relating to the Product but shall redirect such queries to Genentech.

COMPLIANCE WITH PHARMACOVIGILANCE AGREEMENT / AUDIT

The Parties shall follow their own procedures for adherence to AE reporting timelines.

Each Party shall monitor and, as applicable, request feedback from the other Party regarding AE report timeliness in accordance with its own procedures. The Parties agree to provide written responses in a timely manner to inquiries from the other Party regarding AE reports received outside the agreed upon Agreement timelines. If there is any detection of trends of increasing or persistent non-compliance to transmission timelines stipulated in this Agreement, both Parties agree to conduct ad hoc or institute a regular joint meeting to address the issue.

In case of concerns related to non-compliance of processes, other than exchange timelines, with this Agreement, the Parties will jointly discuss and collaborate on clarifying and resolving the issues causing non-

compliance. Every effort will be made by the non-compliant Party to solve the non-compliance issues and inform the other Party of the corrective and preventative actions taken.

Upon justified request, given sufficient notice of no less than sixty (60) calendar days, an audit under the provisions of this Agreement can be requested by either Party. The Parties will then discuss and agree in good faith upon the audit scope, agenda and execution of the audit. The requesting Party will bear the cost of the audit.

6.6 IND OFFICE REVIEW

The investigator is responsible for completing an efficacy/safety summary report and submitting it to the IND Medical Affairs and Safety Group for review. This should be submitted after the first 6 evaluable patients per cohort, complete 1 cycle of study treatment, and every 6 evaluable patients per cohort, thereafter.

7. ETHICAL CONSIDERATIONS

7.1 COMPLIANCE WITH LAWS AND REGULATIONS

Patients who comply with the requirements of the protocol, are tolerating study treatment, and may be receiving benefit will be offered dosing beyond Cycle 1 at the investigator’s discretion after a careful assessment and thorough discussion of the potential risks and benefits of continued treatment with the patient. Such patients may have the option to receive study drugs as long as they continue to experience clinical benefit in the opinion of the investigator until the earlier of unacceptable toxicity, symptomatic deterioration attributed to disease progression, or any of the other reasons for treatment discontinuation listed in Section 4.4.

8. STUDY MEDICAL MONITORING REQUIREMENTS

This clinical research study will be monitored both internally by the PI and externally by the U.T. MD Anderson Cancer Center IND Office monitoring group following a protocol specific monitoring plan. In terms of internal review, the PI will continuously monitor and tabulate AEs. Appropriate reporting to the U.T. MD Anderson Cancer Center IRB will be made. The PI of this study will also continuously monitor the conduct, data, and safety of this study to ensure that:

- Interim analyses occur as scheduled,
- Stopping rules for toxicity and/or response are met,
- Risk/benefit ratio is not altered to the detriment of the subjects,
- Appropriate internal monitoring of AEs and outcomes is done,
- Over-accrual does not occur,
- Under-accrual is addressed with appropriate amendments or actions, and
- Data are being appropriately collected in a reasonably timely manner.

Monitoring will be carried out on an ongoing basis. The severity, relatedness , and whether or not the event is expected will be reviewed.

8.1 STUDY MEDICATION ACCOUNTABILITY

If study drug will be provided by Genentech, the recipient will acknowledge receipt of the drug by returning the INDRR-1 form indicating shipment content and condition. Damaged supplies will be replaced.

Accurate records of all study drug dispensed from and returned to the study site should be recorded by using the institution’s drug inventory log or the National Cancer Institute drug accountability log.

All partially used or empty containers should be disposed of at the study site according to institutional standard operating procedure. Return unopened, expired, or unused study drug with the Inventory of Returned Clinical Material form as directed by Genentech.

8.2 DATA COLLECTION

The study coordinator and investigators are responsible for ensuring that the eligibility checklist is completed in a legible and timely manner for every patient enrolled in the study, and that data are recorded on the appropriate forms and in a timely manner. Any errors on source data should be lined through, but not obliterated, with the correction inserted, initialed, and dated by the study coordinator or PI. All source documents will be available for inspection by the FDA and the U.T. MD Anderson Cancer Center IRB.

9. DATA SECURITY AND CONFIDENTIALITY

Data Security/Confidentiality

Participant confidentiality and privacy is strictly held in trust by the participating investigator, their staff, the safety and oversight monitor(s), and the sponsor(s) and funding agency. This confidentiality is extended to the data being collected as part of this study. Data that could be used to identify a specific study participant will be held in strict confidence within the research team. No personally identifiable information from the study will be released to any unauthorized third party without prior written approval of the sponsor/funding agency, as applicable.

All research activities will be conducted in as private a setting as possible.

Access to Study Records

Study records may be accessed by IRB approved study personnel, or authorized inspectors. The study monitor, other authorized representatives of the sponsor or funding agency, representatives of the Institutional Review Board (IRB), regulatory agencies or representatives from companies or organizations supplying the product, may inspect all documents and records required to be maintained by the investigator, including but not limited to, medical records (office, clinic, or hospital) and pharmacy records for the participants in this study. The clinical study site will permit access to such records.

Methods of Storage of Study Records

All data collected from MD Anderson Cancer Center (MDACC) sources will be maintained on a password protected server compliant with HIPAA. Study staff will have role based restricted access to directories and files on the server, according to project responsibilities. Only those with data entry permissions can add records. The PI or a delegate will review the conditions under which data will be released to recipient-investigators. Each application for use will need IRB approval and consents, if appropriate. The level of identifiability will determine the process for review and approval as well as the way information is shared.

Any study data or records maintained in paper documents will be stored in the offices of the PI or other delegated study staff, in a locked cabinet or other comparable controlled environment, and will be accessible only to authorized study team members or authorized inspectors.

Duration of Study Record Storage

The study participant's contact information will be securely stored at each clinical site for internal use during the study. At the end of the study, all records will continue to be kept in a secure location for as long a period as dictated by the reviewing IRB, Institutional policies, or sponsor/funding agency requirements.

Sharing of Study Records

There are no plans to share study data with entities external to MD Anderson Cancer Center, aside from authorized inspectors as applicable (i.e. authorized representatives of the sponsor or funding agency, representatives of the Institutional Review Board (IRB), regulatory agencies or representatives from companies or organizations supplying the product). If data will be shared, IRB approval will be sought, and applicable inter-institutional agreements executed, prior to data sharing.

10. REFERENCES

Ain, K. B., M. J. Egorin and P. A. DeSimone (2000). "Treatment of anaplastic thyroid carcinoma with paclitaxel: phase 2 trial using ninety-six-hour infusion. Collaborative Anaplastic Thyroid Cancer Health Intervention Trials (CATCHIT) Group." Thyroid **10**(7): 587-594.
Alberts, B., A. Johnson, J. Lewis, M. Raff, K. Roberts and P. Walter (2008). Mechanisms of Cell Communication. Molecular biology of the cell. New York, Garland Science.

Brauner, E., V. Gunda, P. Vanden Borre, D. Zurakowski, Y. S. Kim, K. V. Dennett, S. Amin, G. J. Freeman and S. Parangi (2016). "Combining BRAF inhibitor and anti PD-L1 antibody dramatically improves tumor regression and anti tumor immunity in an immunocompetent murine model of anaplastic thyroid cancer." Oncotarget **7**(13): 17194-17211.

Brose, M. S., M. E. Cabanillas, E. E. Cohen, L. J. Wirth, T. Riehl, H. Yue, S. I. Sherman and E. J. Sherman (2016). "Vemurafenib in patients with BRAF(V600E)-positive metastatic or unresectable papillary thyroid cancer refractory to radioactive iodine: a non-randomised, multicentre, open-label, phase 2 trial." Lancet Oncol **17**(9): 1272-1282.

Cabanillas, M. E., N. L. Busaidy, S. A. Khan, B. G. Gunn, R. Dadu, S. N. Rao and S. G. Waguespack (2016). "Molecular Diagnostics and anaplastic thyroid carcinoma: the time has come to harvest the high hanging fruit." Intl. J Endocrine Oncology **3**(3): 221-233.

Cabanillas, M. E., M. Zafereo, G. B. Gunn and R. Ferrarotto (2016). "Anaplastic Thyroid Carcinoma: Treatment in the Age of Molecular Targeted Therapy." J Oncol Pract **12**(6): 511-518.

Dadu, R., P. A. Vilalobos, E. R. Para Cuentas, J. ROdriguez Canales, I. Wistuba, S. Zhou, M. D. Williams and M. E. Cabanillas (2016). "Anaplastic

thyroid cancer (ATC) is a hot immunogenic environment: immunoprofiling of a large cohort of ATC tumors." Thyroid **26**(Suppl 1): Abstr 12.

Davies, H., G. R. Bignell, C. Cox, P. Stephens, S. Edkins, S. Clegg, J. Teague, H. Woffendin, M. J. Garnett, W. Bottomley, N. Davis, E. Dicks, R. Ewing, Y. Floyd, K. Gray, S. Hall, R. Hawes, J. Hughes, V. Kosmidou, A. Menzies, C. Mould, A. Parker, C. Stevens, S. Watt, S. Hooper, R. Wilson, H. Jayatilake, B. A. Gusterson, C. Cooper, J. Shipley, D. Hargrave, K. Pritchard-Jones, N. Maitland, G. Chenevix-Trench, G. J. Riggins, D. D. Bigner, G. Palmieri, A. Cossu, A. Flanagan, A. Nicholson, J. W. Ho, S. Y. Leung, S. T. Yuen, B. L. Weber, H. F. Seigler, T. L. Darrow, H. Paterson, R. Marais, C. J. Marshall, R. Wooster, M. R. Stratton and P. A. Futreal (2002). "Mutations of the BRAF gene in human cancer." Nature **417**(6892): 949-954.

Falchook, G. S., M. Millward, D. Hong, A. Naing, S. Piha-Paul, S. G. Waguespack, M. E. Cabanillas, S. I. Sherman, B. Ma, M. Curtis, V. Goodman and R. Kurzrock (2015). "BRAF inhibitor dabrafenib in patients with metastatic BRAF-mutant thyroid cancer." Thyroid **25**(1): 71-77.

Flaherty, K. T., I. Puzanov, K. B. Kim, A. Ribas, G. A. McArthur, J. A. Sosman, P. J. O'Dwyer, R. J. Lee, J. F. Grippo, K. Nolop and P. B. Chapman (2010). "Inhibition of mutated, activated BRAF in metastatic melanoma." N Engl J Med **363**(9): 809-819.

Higashiyama, T., Y. Ito, M. Hirokawa, M. Fukushima, T. Uruno, A. Miya, F. Matsuzuka and A. Miyauchi (2010). "Induction chemotherapy with weekly paclitaxel administration for anaplastic thyroid carcinoma." Thyroid **20**(1): 7-14.

Hyman, D. M., I. Puzanov, V. Subbiah, J. E. Faris, I. Chau, J. Y. Blay, J. Wolf, N. S. Raje, E. L. Diamond, A. Hollebecque, R. Gervais, M. E. Elez-Fernandez, A. Italiano, R. D. Hofheinz, M. Hidalgo, E. Chan, M. Schuler, S. F. Lasserre, M. Makrutzki, F. Sirzen, M. L. Veronese, J. Tabernero and J. Baselga (2015). "Vemurafenib in Multiple Nonmelanoma Cancers with BRAF V600 Mutations." N Engl J Med **373**(8): 726-736.

Iyer, P., R. Dadu, N. Busaidy, R. Ferrarotto, M. Gule-Monroe, C. Lu, M. D. Williams and M. E. Cabanillas (2016). "Harvesting high-hanging fruit: targeted therapy for BRAF mutant and BRAF wild-type anaplastic thyroid cancer." Thyroid **26**(Suppl 1): abstr 194.

Kim, K. B., M. E. Cabanillas, A. J. Lazar, M. D. Williams, D. L. Sanders, J. L. Ilagan, K. Nolop, R. J. Lee and S. I. Sherman (2013). "Clinical Responses to Vemurafenib in Patients with Metastatic Papillary Thyroid Cancer Harboring BRAF Mutation." Thyroid.

Landa, I., T. Ibrahimpasic, L. Boucai, R. Sinha, J. A. Knauf, R. H. Shah, S. Dogan, J. C. Ricarte-Filho, G. P. Krishnamoorthy, B. Xu, N. Schultz, M. F. Berger, C. Sander, B. S. Taylor, R. Ghossein, I. Ganly and J. A. Fagin (2016). "Genomic and transcriptomic hallmarks of poorly differentiated and anaplastic thyroid cancers." J Clin Invest.

Prager, G., O. Koperek, M. Mayerhofer, L. Muellauer, F. Wrba, B. Niederle, C. Zielinski and M. Raderer (2016). "Sustained Response to Vemurafenib in an BRAF V600E-mutated Anaplastic Thyroid Carcinoma Patient." Thyroid.

Rosove, M. H., P. F. Peddi and J. A. Glaspy (2013). "BRAF V600E inhibition in anaplastic thyroid cancer." N Engl J Med **368**(7): 684-685.

Schweppe, R. E., J. P. Klopper, C. Korch, U. Pugazhenthi, M. Benezra, J. A. Knauf, J. A. Fagin, L. A. Marlow, J. A. Copland, R. C. Smallridge and B. R. Haugen (2008). "Deoxyribonucleic acid profiling analysis of 40 human thyroid cancer cell lines reveals cross-contamination resulting in cell line redundancy and misidentification." J Clin Endocrinol Metab **93**(11): 4331-4341.

Smallridge, R. C., K. B. Ain, S. L. Asa, K. C. Bible, J. D. Brierley, K. D. Burman, E. Kebebew, N. Y. Lee, Y. E. Nikiforov, M. S. Rosenthal, M. H. Shah, A. R. Shaha, R. M. Tuttle and T. American Thyroid Association Anaplastic Thyroid Cancer Guidelines (2012). "American Thyroid Association guidelines for management of patients with anaplastic thyroid cancer." Thyroid **22**(11): 1104-1139.

Takahashi, S., N. Kiyota, T. Yamazaki, N. Chayahra, K. Nakano, L. Inagaki, K. Toda, T. Enokida, H. Minami, Y. Imamura, T. Sasaki, K. Fujino, C. Ductus and M. Tahara (2016). "Phase II study of lenvatinib in patients with differentiated, medullary, and anaplastic thyroid cancer: Final analysis results." J Clin Oncol **34**(suppl): abstr 6088.

Appendix 1 Study Flowchart

Cohort 1 (BRAF mutated): Vemurafenib, cobimetinib, atezolizumab triplet[q]

	Screening			Treatment Period (cycles are 28 days) * +3/-5 day window, **+3 day window								Progression	Study Completion/Early Term Visit	Follow-Up[l]
Day	-28 through -1	Run-in [h]	Run-In D15	C1D1	C1D15	C2D1	C2D15	C3+D1	C3+D15	C4D1 then every 3rd cycle D1	C6D15, then every 3rd cycle D15			
Informed consent	X													
Demographic data	X													
General med history & baseline conditions	X													
Pregnancy test[f]	X			X		X*		X*						
Vital signs [a]	X			X	X*	X*	X*	X*	X*				X	
Weight (& height at screening only)	X			X	X*	X*	X*	X*	X*				X	
Physical examination[b]	X			X		X*		X*					X	
Hematology [c]	X			X	X*	X*	X*	X*	X*				X	
Chemistry [d]	X			X	X*	X*	X*	X*	X*				X	
Coagulation [e]	X													
PK trough levels (suspension cobimetinib and crushed vemurafenib patients only)[p]			X											
Additional Blood Collection[n]	X					X*						X		
Urinalysis	X					X*		X*					X	
ECHO[f]	X			See Footnote "f"									X	
ECG [g]	X[g]		X		X[g]		X[g]		X[g]		X[g]		X[g]	
Nab-paclitaxel/Paclitaxel[o]	X													
Cobimetinib[n]		X		X	X**	X**	X**	X**	X**					
Vemurafenib[n]		X		X	X*	X*	X*	X*	X*					
atezolizumab[n]				X	X*	X*	X*	X*	X*					
Response assessment [i]	X			Scans will be done every 8 weeks (+3/-7 day window)									X	
Opthalmologic exam [j]	X	See Footnote "j"												
Dermatologic exam [k]	X			X[k]*						X*			X*	X[k]
Concomitant medications [l]	X					X*		X*					X	
Adverse events	X					X*		X*					X	X
Biopsy	X[m]					X*						X		

ł Pregnancy test, serum or urine at baseline, urine prior to every cycle for women of child-bearing potential; All WOCBP must have a negative serum or urine pregnancy test within 14 days prior to start of study drug.
a Heart rate, respiratory rate, systolic and diastolic blood pressure while the patient is in a seated position, and temperature.
b Physical examination only on day 1 of each cycle. If physical examinations are assessed within 7 days of the Cycle 1 Day 1 visit, they do not have to be repeated at Day 1. After 6 months on trial, if stable from a disease and AE standpoint (in the opinion of the PI), patients physical exam visits with MD will be at every re-staging visit only. Research nurse will visit (either face-to-face or via telephone) with the patient prior to the start of the cycle, unless the patient is on atezolizumab hold, in which case the research nurse visit will occur when the patient returns for physical exam with physician. All physical exams may be conducted via telemedicine.
c Hemoglobin, hematocrit, platelet count, RBC count, WBC count, percent and absolute differential count. Not necessary to perform on Day 15 of cycle if patient is not being treated with Atezolizumab.
d Sodium, potassium, chloride, bicarbonate, glucose, BUN, creatinine, calcium, phosphorus, magnesium, total and direct bilirubin, total protein, albumin, ALT, AST, LDH, alkaline phosphatase, creatinine phosphokinase, uric acid, TSH, free T4, thyroglobulin; LFTs (AST, ALT, total bilirubin) must be tested prior to Atezolizumab administration on C1D1; Not necessary to perform on Day 15 of cycle if patient is not being treated with Atezolizumab. Screening labs also includes testing for Hep B surface antigen (HBVsAg) and Hep B core antibody (HBVcAb), and Hep C virus antibody
e Prothrombin time/International Normalized Ratio, activated Partial thromboplastin time, fibrinogen, D-dimer.
f All patients will undergo evaluation of left ventricular dysfunction by ECHO at screening. Further, evaluation of LVEF by ECHO must be performed at the following time points: Cycle 2, Day 1 ± 2 weeks; Day 1 of Cycles 5, 8, 11, 14 (every 3 treatment cycles) ± 2 weeks; Day 1 of Cycles 18+ (every 4 treatment cycles) ± 2 weeks.
g ECG results will be evaluated by the investigator for determination of eligibility at screening and for the purposes of real-time cardiac safety monitoring and suitability for continued study treatment. All ECG recordings must be performed using a standard high-quality, high-fidelity digital electrocardiograph machine equipped with computer-based interval measurements. Paper copies of ECG tracings will be kept as part of the patient's permanent study file at the site. Triplicate 12-lead ECG recordings will be obtained at screening. Single 12-lead ECG recordings will be obtained on Run-In Day 15 (+ 3 days); on Day 15 (+ 3 days) of Cycles 1, 2, 3, and every three cycles thereafter (i.e., Day 15 of Cycles 6, 9, 12, etc.) (+ 3 days); at the treatment discontinuation visit (+ 3 days); and as clinically indicated. If patient is on atezolizumab hold, Day 15 ECGs may be performed on Day 1 beginning after Cycle 3.
h Run-in period and subsequent dosing: vemurafenib 960mg bid (day 1-21) + cobimetinib 60mg q day (day 1-21) run-in. On days 21-28 of the run-in, vemurafenib will be reduced to 720 mg bid and cobimetinib will be held. atezolizumab 840 mg q2 weeks will be started on cycle 1, day 1 (after the run in period). Vemurafenib will be dose reduced to 720mg bid (days 1-28) and cobimetinib 60mg will be taken on days 1-21 once atezolizumab is started. Patients who have > grade 3 LFTs (AST, ALT or total bilirubin) will not receive atezolizumab but may continue on vemurafenib + cobimetinib with dose reduction. If after dose reductions, the LFTs are below grade 3, patient may start atezolizumab.
i Response assessment by cross-sectional imaging such as CT, MRI, or PET-CT as per standard of care for each disease. Imaging may occur up to 7 days prior to scheduled day. Baseline assessment must occur within 28 days prior to day 1. Screening brain MRI or CT required unless already obtained within 3 months prior to screening. PET/CTs may be substituted for CT of chest at baseline and re-staging, if in the opinion of the treating physician, target lesions can be accurately measured.
j Results of standard of care tests or examinations performed prior to obtaining informed consent and within 28 days prior to Day 1 may be used; such tests do not need to berepeated for screening. Eye exams will also be performed at the following time points: Cycle 2, Day 1 ± 2 weeks; Day 1 of Cycles 5, 8, and 11 (every 3 treatment cycles) ± 2 weeks; Day 1 of Cycles 15+ (every 4 treatment cycles) ± 2 weeks. After the screening visit, follow up evaluations may be performed by telemedicine video visits by an ophthalmologist. If ocular side effects (including blurred vision) are noted on telemedicine visit during the study, it is recommended that an evaluation by an ophthalmologist be performed in clinic and additional tests conducted as clinically warranted. In particular, if a patient were to present signs and symptoms suggestive of keratitis (such as eye inflammation, lacrimation, light sensitivity, blurred vision, eye pain and/or red eye), a prompt ophthalmologist consult is required.
kDue to the risk of cutaneous squamous cell carcinoma, all patients will undergo complete dermatologic evaluation at screening, on Day 1 of Cycle 1 and every three cycles thereafter during treatment (i.e., Day 1 of Cycles 4, 7, 10, etc.) at the treatment discontinuation visit (unless performed within the previous 12 weeks), 6 months (□2 weeks) after the last dose of vemurafenib, and as clinically indicated. These visits may be conducted via telemedicine.
l All patients who discontinue treatment without objective disease progression will be followed up with tumor assessment every 3 months or as determined by PI or designee until disease progression or the start of a different anti-cancer therapy. Survival follow-up via phone call or visit with physician will occur every 3 months or as determined by PI or designee for the first 2 years once they are off study treatment, then every 6 months during Years 3 and 4, and yearly thereafter until patient death, withdrawal of consent, patient is lost to follow-up, or study termination by Genentech, whichever occurs first..
[m]Screening biopsy is optional for all patients.
[n]Additional blood collections will occur at screening, C2D1 and at initial radiographic progression for a) isolation of germ-line DNA from PMBCs ;b) isolation of plasma for cell-free DNA analysis of genomic abnormalities using gene panels; c) potential flow cytometry analyses for phenotypic and functional studies and potential liquid biopsy studies.
[o] Nab-paclitaxel/Paclitaxel may be administered at a local facility
Includes testing for Hep B surface antigen (HBVsAg) and Hep B core antibody (HBVcAb), and Hep C virus antibody
[p] Blood draw for trough level PKs to occur prior to Run-In D15 dose of cobimetinib and vemurafenib
[q] When visits are delayed, **the cycle continues to count;** when the patient returns, Day 1 of the next cycle begins

Cohort 2 (RAS, NF1, or NF2 mutated, including patients with tumors with MAPK activating mutations at or above MEK:**):**
Cobimetinib, atezolizumab combination[p]

	Screening	Treatment Period (cycles are 28 days) * +3/-5 day window, **+3 day window						Progression	Study Completion/Early Term Visit	Follow-Up[j]
Day	-28 through -1	C1D1	C1D15	C2D1	C2D15	C3+D1	C3+D15			
Informed consent	X									
Demographic data	X									
General med history & baseline conditions	X									
Pregnancy test[f]	X	X		X*		X*				
Vital signs [a]	X	X	X*	X*	X*	X*	X*		X	
Weight (& height at screening only)	X	X	X*	X*	X*	X*	X*		X	
Physical examination[b]	X	X		X*		X*			X	
Hematology [c]	X	X[m]	X*	X*	X*	X*	X*		X	
Chemistry [d]	X	X[m]	X*	X*	X*	X*	X*		X	
Coagulation [e]	X									
PK trough levels (suspension cobimetinib patients only)[o]			X							
Additional Blood Collection[l]	X			X*				X		
Urinalysis	X			X*		X*			X	
ECHO[f]	X	See Footnote "f"							X	
Nab-paclitaxel/Paclitaxel[n]	X									
Cobimetinib[g]		X	X**	X**	X**	X**	X**			
atezolizumab[g]		X	X*	X*	X*	X*	X*			
Response assessment [h]	X	Scans will be done every 8 weeks (+3/-7 day window)							X	
Opthalmologic exam[i]	X	See Footnote "i"								
Concomitant medications[j]	X			X*		X*			X	
Adverse events	X			X*		X*			X	X
Biopsy	X[k]			X*				X		

	Screening	Treatm ent Period (cycles are 28 days) * +3/-5 d y window, **+3 day window						Progression	Study Completion/Early Term Visit	Follow-Up[illegible]
Day	-28 through -1	C1D1	C1D15	C2D1	C2D15	C3+D1	C3+D15			

[f] Pregnancy test, serum or urine at baseline, urine prior to every cycle for women of child-bearing potential; All WOCBP must have a negative serum or urine pregnancy test within 14 days prior to start of study drug.
[a] Heart rate, respiratory rate, systolic and diastolic blood pressure while the patient is in a seated position, and temperature. Vital signs, including weight and height, assessed within 7 days of the Cycle 1 Day 1 visit do not have to be repeated at Day 1.
[b] Physical examination only on day 1 of each cycle. If physical examinations are assessed within 7 days of the Cycle 1 Day 1 visit, they do not have to be repeated at Day 1. After 6 months on trial, if stable from a disease and AE standpoint (in the opinion of the PI), patients physical exam visits with MD will be at every re-staging visit only. Research nurse will visit (either face-to-face or via telephone) with the patient prior to the start of the cycle, unless the patient is on atezolizumab hold, in which case the research nurse visit will occur when the patient returns for physical exam with physician. All physical exams may be conducted via telemedicine.
[c] Hemoglobin, hematocrit, platelet count, RBC count, WBC count, percent and absolute differential count. Not necessary to perform on Day 15 of cycle if patient is not being treated with Atezolizumab.
[d] Sodium, potassium, chloride, bicarbonate, glucose, BUN, creatinine, calcium, phosphorus, magnesium, total and direct bilirubin, total protein, albumin, ALT, AST, LDH, alkaline phosphatase, creatinine phosphokinase, CPK, uric acid, TSH, free T4, thyroglobulin. Not necessary to perform on Day 15 of cycle if patient is not being treated with Atezolizumab. **Screening** labs also includes testing for **Hep B surface antigen (HBVsAg) and Hep B core antibody (HBVcAb), and Hep C virus antibody**
[e] Prothrombin time/International Normalized Ratio, activated Partial thromboplastin time, fibrinogen, D-dimer.
[f] All patients will undergo evaluation of left ventricular dysfunction by ECHO at screening. Further, evaluation of LVEF by ECHO must be performed at the following time points: Cycle 2, Day 1 ± 2 weeks; Day 1 of Cycles 5, 8, 11, and 14 (every 3 treatment cycles) ± 2 weeks; Day 1 of Cycles 18+ (every 4 treatment cycles) ± 2 weeks..
[g] cobimetinib 60 mg orally on Days 1−21 plus atezolizumab 840 mg IV on Day 1 and Day 15 in a 28-day cycle
[h] Response assessment by cross-sectional imaging such as CT, MRI, or PET-CT as per standard of care for each disease. Imaging may occur up to 7 days prior to scheduled day. Baseline assessment must occur within 28 days prior to day 1. Screening brain MRI or CT required unless already obtained within 3 months prior to screening. PET/CTs may be substituted for CT of chest at baseline and re-staging, if in the opinion of the treating physician, target lesions can be accurately measured
[i] Results of standard of care tests or examinations performed prior to obtaining informed consent and within 28 days prior to Day 1 may be used; such tests do not need to be repeated for screening. Eye exams will also be performed at the following time points: Cycle 2, Day 1 ± 2 weeks; Day 1 of Cycles 5, 8, and 11 (every 3 treatment cycles) ± 2 weeks; Day 1 of Cycles 15+ (every 4 treatment cycles) ± 2 weeks. After the screening visit, follow up evaluations may be performed by telemedicine video visits by an ophthalmologist. If ocular side effects (including blurred vision) are noted on telemedicine visit during the study, it is recommended that an evaluation by an ophthalmologist be performed in clinic and additional tests conducted as clinically warranted. In particular, if a patient were to present signs and symptoms suggestive of keratitis (such as eye inflammation, lacrimation, light sensitivity, blurred vision, eye pain and/or red eye), a prompt ophthalmologist consult is required.
[j] All patients who discontinue treatment without objective disease progression will be followed up with tumor assessment every 3 months or as determined by PI or designee until disease progression or the start of a different anti-cancer therapy. Survival follow-up via phone call or visit with physician will occur every 3 months or as determined by PI or designee for the first 2 years once they are off study treatment, then every 6 months during Years 3 and 4, and yearly thereafter until patient death, withdrawal of consent, patient is lost to follow-up, or study termination by Genentech, whichever occurs first.
[K] Screening biopsy is optional for all patients.

[l] Additional blood collections will occur at screening, C2D1 and at initial radiographic progression for a)isolation of germ-line DNA from PMBCs; b)isolation of plasma for cell-free DNA analysis of genomic abnormalities using gene panels ;c)potential flow cytometry analyses for phenotypic and functional studies and potential liquid biopsy studies.

[m] If screening hematology or chemistry labs were done within 7 days of the Cycle 1 Day 1 visit, they do not need to be repeated at Day 1.
[n] Nab-paclitaxel/Paclitaxel may be administered at a local facility
o Blood draw for trough level PKs to occur prior to C1 D15 dose of cobimetinib
p When visits are delayed, the cycle continues to count; when the patient returns, Day 1 of the next cycle begins

Cohort 3 (non-BRAF, non-RAS mutated): Bevacizumab, atezolizumab combination[o]

	Screening	Treatment Period – Every 21 Days *+3/-5 day window				Progression	Study Completion/Early Term Visit	Follow-Up[h]
Day	-28 through -1	C1D1	C2D1	C3D1	C4+D1			
Informed consent	X							
Demographic data	X							
General med history & baseline conditions	X							
Pregnancy test[f]	X	X	X*	X*	X*			
Vital signs [a]	X	X	X*	X*	X*		X	
Weight (& height at screening only)	X	X	X*	X*	X*		X	
Physical examination[i]	X	X	X*	X*	X*		X	
Hematology [b]	X	X[m]	X*	X*	X*		X	
Chemistry [c]	X	X[m]	X*	X*	X*		X	
Coagulation [d]	X							
Additional Blood Collection[n]	X		X*			X		

Day	Screening	Treatment Period – Every 21 Days *+3/-5 day window				Progression	Study Completion/Early Term Visit	Follow-Up[h]
	-28 through -1	C1D1	C2D1	C3D1	C4+D1			
Urinalysis[j]	X	X	X*	X*	X*		X	
Nab-paclitaxel/Paclitaxel[n]	X							
Bevacizumab administration[g]		X	X*	X*	X*			
atezolizumab administration[g]		X	X*	X*	X*			
Response assessment [e]	X	Scans will be done every 6 weeks (+3/-7 day window)					X	
Concomitant medications	X	X	X*	X*	X*		X	
Adverse events	X	X	X*	X*	X*		X	X
Biopsy	X[f]		X*			X		

[l] Pregnancy test, serum or urine at baseline, urine prior to every cycle for women of child-bearing potential; All WOCBP must have a negative serum or urine pregnancy test within 14 days prior to start of study drug. Vital signs, including weight and height, assessed within 7 days of the Cycle 1 Day 1 visit do not have to be repeated at Day 1. [a]Heart rate, respiratory rate, systolic and diastolic blood pressure while the patient is in a seated position, and temperature.

[b]Hemoglobin, hematocrit, platelet count, RBC count, WBC count, percent and absolute differential count (neutrophils, bands, eosinophils, lymphocytes, monocytes, basophils, other cells).

[c]Sodium, potassium, chloride, bicarbonate, glucose, BUN, creatinine, calcium, phosphorus, magnesium, total and direct bilirubin, total protein, albumin, ALT, AST, LDH, alkaline phosphatase, and uric acid, TSH, free T4, thyroglobulin. **Screening** labs also includes testing for **Hep B surface antigen (HBVsAg) and Hep B core antibody (HBVcAb), and Hep C virus antibody**

[d] Prothrombin time/International Normalized Ratio, activated Partial thromboplastin time, fibrinogen, D-dimer.

[e] Response assessment by cross-sectional imaging such as CT, MRI, or PET-CT as per standard of care for each disease. Imaging may occur up to 7 days prior to scheduled day. Baseline assessment must occur within 28 days prior to day 1. Screening brain MRI or CT required unless already obtained within 3 months prior to screening. PET/CTs may be substituted for CT of chest at baseline and re-staging, if in the opinion of the treating physician, target lesions can be accurately measured

[f] Screening biopsy is optional for all patients.

[g] atezolizumab 1200 mg q3 weeks; bevacizumab 15 mg/kg q3 weeks

[h] Additional blood collections will occur at screening, C2D1 and at initial radiographic progression for a)isolation of germ-line DNA from PMBCs; b)isolation of plasma for cell-free DNA analysis of genomic abnormalities using gene panels; c)potential flow cytometry analyses for phenotypic and functional studies and potential liquid biopsy studies.

[i] Physical examination only on day 1 of each cycle. If physical examinations are assessed within 7 days of the Cycle 1 Day 1 visit, they do not have to be repeated at Day 1. After 6 months on trial, if stable from a disease and AE standpoint (in the opinion of the PI), patients physical exam visits with MD will be at every re-staging visit only. Research nurse will visit with the patient prior to the start of the cycle.

[j] Proteinuria must be assessed before each bevacizumab administration unless proteinuria has been determined by 24-hour urine collection; For 2+ proteinuria: may administer bevacizumab without dose modification and collect 24-hour urine prior to subsequent bevacizumab administration

[h] All patients who discontinue treatment without objective disease progression will be followed up with tumor assessment every 3 months or as determined by PI or designee until disease progression or the start of a different anti-cancer therapy. Survival follow-up via phone call or visit with physician will occur every 3 months or as determined by PI or designee for the first 2 years once they are off study treatment, then every 6 months during Years 3 and 4, and yearly thereafter until patient death, withdrawal of consent, patient is lost to follow-up, or study termination by Genentech, whichever occurs first

[m] If screening hematology or chemistry labs were done within 7 days of the Cycle 1 Day 1 visit, they do not need to be repeated at Day 1.

[n] Nab-paclitaxel/Paclitaxel may be administered at a local facility.

[o] When visits are delayed, **the cycle continues to count;** when the patient returns, Day 1 of the next cycle begins

Cohort 4: Nab-paclitaxel (or paclitaxel), atezolizumab[n]

Day	Screening	Treatment Period—Every 21 days* +3/-5 day window				Progression	Study Completion/Early Term Visit	Follow-Up[j]
	-28 through -1	C1D1	C2D1	C3D1	C4D1			
Informed consent	X							
Demographic data	X							
General med history & baseline conditions [n]	X							
Pregnancy test[l]	X	X	X*	X*	X*			
Vital signs [a]	X	X	X*	X*	X*		X	
Weight (& height at screening only)	X	X	X*	X*	X*		X	
Physical examination[i]	X	X	X*	X*	X*		X	

Day	Screening	Treatment Period—Every 21 days* +3/-5 day window				Progression	Study Completion/Early Term Visit	Follow-Up[j]
	-28 through -1	C1D1	C2D1	C3D1	C4D1			
Hematology [b]	X	X[m]	X*	X*	X*		X	
Chemistry [c]	X	X[m]	X*	X*	X*		X	
Coagulation [d]	X							
Additional Blood Collection[h]	X		X*			X		
Urinalysis	X	X	X*	X*	X*		X	
Nab-paclitaxel/Paclitaxel	X*							
atezolizumab administration [e]		X	X*	X*	X*			
Response assessment [f]	X	Scans will be done every 6 weeks (+3/-7 day window)					X	
Concomitant medications	X	X	X*	X*	X*		X	
Adverse events	X	X	X*	X*	X*		X	X
Biopsy	X[g]		X*			X		

[i] Pregnancy test, serum or urine at baseline, urine prior to every cycle for women of child-bearing potential; All WOCBP must have a negative serum or urine pregnancy test within 14 days prior to start of study drug.
[a]Heart rate, respiratory rate, systolic and diastolic blood pressure while the patient is in a seated position, and temperature. Vital signs, including weight and height, assessed within 7 days of the Cycle 1 Day 1 visit do not have to be repeated at Day 1.[b]Hemoglobin, hematocrit, platelet count, RBC count, WBC count, percent and absolute differential count (neutrophils, bands, eosinophils, lymphocytes, monocytes, basophils, other cells).
[c]Sodium, potassium, chloride, bicarbonate, glucose, BUN, creatinine, calcium, phosphorus, magnesium, total and direct bilirubin, total protein, albumin, ALT, AST, LDH, alkaline phosphatase, and uric acid, TSH, free T4, thyroglobulin. **Screening** labs also includes testing for **Hep B surface antigen (HBVsAg) and Hep B core antibody (HBVcAb), and Hep C virus antibody**
[d] Prothrombin time/International Normalized Ratio, activated Partial thromboplastin time, fibrinogen, D-dimer.
[e] atezolizumab 1200mg IV every 21 days. Paclitaxel may be substituted for nab-paclitaxel but nab-paclitaxel is preferred. The dose of nab-paclitaxel is 100mg/m2 on days 1, 8, and 15. The dose of paclitaxel is 175mg/m^2 every 21 days. Any patient assigned to cohort 4 is eligible to receive nab-paclitaxel at a local facility.
[f] Response assessment by cross-sectional imaging such as CT, MRI, or PET-CT as per standard of care for each disease. Imaging may occur up to 7 days prior to scheduled day. Baseline assessment must occur within 28 days prior to day 1. Screening brain MRI or CT required unless already obtained within 3 months prior to screening. PET/CTs may be substituted for CT of chest at baseline and re-staging, if in the opinion of the treating physician, target lesions can be accurately measured.
[g] Screening biopsy is optional all patients.
[h] Additional blood collections will occur at screening, C2D1 and at initial radiographic progression for a)isolation of germ-line DNA from PMBCs; b)isolation of plasma for cell-free DNA analysis of genomic abnormalities using gene panels; c)potential flow cytometry analyses for phenotypic and functional studies and potential liquid biopsy studies.
[i] Physical examination only on day 1 of each cycle. If physical examinations are assessed within 7 days of the Cycle 1 Day 1 visit, they do not have to be repeated at Day 1. After 6 months on trial, if stable from a disease and AE standpoint (in the opinion of the PI), patients physical exam visits with MD will be at every re-staging visit only. Research nurse will visit with the patient prior to the start of the cycle.
[j]All patients who discontinue treatment without objective disease progression will be followed up with tumor assessment every 3 months or as determined by PI or designee until disease progression or the start of a different anti-cancer therapy. Survival follow-up via phone call or visit with physician will occur every 3 months or as determined by PI or designee for the first 2 years once they are off study treatment, then every 6 months during Years 3 and 4, and yearly thereafter until patient death, withdrawal of consent, patient is lost to follow-up, or study termination by Genentech, whichever occurs first.
[m] If screening hematology or chemistry labs were done within 7 days of the Cycle 1 Day 1 visit, they do not need to be repeated at Day 1.
[n] When visits are delayed, **the cycle continues to count;** when the patient returns, Day 1 of the next cycle begins

Appendix 2 Calculation of Creatinine Clearance Using the Cockcroft-Gault Formula

$$\text{Creatinine Clearance (men)} = \frac{(140\text{-Age}) \times \text{Lean Body Weight [kilograms]}}{\text{Serum Creatinine (mg/dL)} \times 72}$$

$$\text{Creatinine Clearance (women)} = \frac{0.85 \times (140\text{-Age}) \times \text{Lean Body Weight [kilograms]}}{\text{Serum Creatinine (mg/dL)} \times 72}$$

Appendix 3 Current National Cancer Institute Common Terminology Criteria for Adverse Events (NCI CTCAE)

Please use the following link to the NCI CTCAE website:

http://ctep.cancer.gov/protocolDevelopment/electronic_applications/ctc.htm

Appendix 4 Response Evaluation Criteria in Solid Tumors (RECIST)

Modified Excerpt from Original Publication

Selected sections from the Response Evaluation Criteria in Solid Tumors (RECIST), Version 1.1[1] are presented below, with slight modifications and the addition of explanatory text as needed for clarity.[2]

Measurability of Tumor at Baseline

Definitions

At baseline, tumor lesions/lymph nodes will be categorized measurable or non-measurable as follows:

a. Measurable Tumor Lesions

Tumor Lesions. Tumor lesions must be accurately measured in at least one dimension (longest diameter in the plane of measurement is to be recorded) with a minimum size of:

- 10 mm by CT or MRI scan (CT/MRI scan slice thickness/interval no greater than 5 mm)
- 10-mm caliper measurement by clinical examination (lesions that cannot be accurately measured with calipers should be recorded as non-measurable)
- 20 mm by chest X-ray

Malignant Lymph Nodes. To be considered pathologically enlarged and measurable, a lymph node must be □ 15 mm in the short axis when assessed by CT scan (CT scan slice thickness recommended to be no greater than 5 mm). At baseline and in follow-up, only the short axis will be measured and followed. See also notes below on "Baseline Documentation of Target and Non-Target Lesions" for information on lymph node measurement.

b. Non-Measurable Tumor Lesions

Non-measurable tumor lesions encompass small lesions (longest diameter □ 10 mm or pathological lymph nodes with □ 10 to □ 15 mm short axis), as well as truly non-measurable lesions. Lesions considered truly non-measurable include: leptomeningeal disease, ascites, pleural or pericardial effusion, inflammatory breast disease, lymphangitic involvement of skin or lung, peritoneal spread, and abdominal masses/abdominal organomegaly identified by physical examination that is not measurable by reproducible imaging techniques.

---

[1] Eisenhauer EA, Therasse P, Bogaerts J, et al. New response evaluation criteria in solid tumors: Revised RECIST guideline (Version 1.1). Eur J Cancer 2009;45:228–47.

[2] For consistency within this document, the section numbers and cross-references to other sections within the article have been deleted and minor formatting changes have been made.

c. Special Considerations Regarding Lesion Measurability

Bone lesions, cystic lesions, and lesions previously treated with local therapy require particular comment, as outlined below.

Bone lesions:

- Bone scan, positron emission tomography (PET) scan, or plain films are not considered adequate imaging techniques to measure bone lesions. However, these techniques can be used to confirm the presence or disappearance of bone lesions.
- Lytic bone lesions or mixed lytic-blastic lesions, with identifiable soft tissue components, that can be evaluated by cross-sectional imaging techniques such as CT or MRI can be considered measurable lesions if the soft tissue component meets the definition of measurability described above.
- Blastic bone lesions are non-measurable.

Cystic lesions:

- Lesions that meet the criteria for radiographically defined simple cysts should not be considered malignant lesions (neither measurable nor non-measurable) since they are, by definition, simple cysts.
- Cystic lesions thought to represent cystic metastases can be considered measurable lesions if they meet the definition of measurability described above. However, if non-cystic lesions are present in the same patient, these are preferred for selection as target lesions.

Lesions with prior local treatment:

- Tumor lesions situated in a previously irradiated area, or in an area subjected to other loco-regional therapy, are usually not considered measurable unless there has been demonstrated progression in the lesion. Study protocols should detail the conditions under which such lesions would be considered measurable.

Target Lesions: Specifications by Methods of Measurements

a. Measurement of Lesions

All measurements should be recorded in metric notation, using calipers if clinically assessed. All baseline evaluations should be performed as close as possible to the treatment start and never more than 4 weeks before the beginning of the treatment.

b. Method of Assessment

The same method of assessment and the same technique should be used to characterize each identified and reported lesion at baseline and during study. Imaging-based evaluation should always be the preferred option.

Clinical Lesions. Clinical lesions will only be considered measurable when they are superficial and □ 10 mm in diameter as assessed using calipers (e.g., skin nodules).

For the case of skin lesions, documentation by color photography, including a ruler to estimate the size of the lesion, is suggested.

Chest X-Ray. Chest CT is preferred over chest X-ray, particularly when progression is an important endpoint, since CT is more sensitive than X-ray, particularly in identifying new lesions. However, lesions on chest X-ray may be considered measurable if they are clearly defined and surrounded by aerated lung.

CT, MRI. CT is the best currently available and reproducible method to measure lesions selected for response assessment. This guideline has defined measurability of lesions on CT scan based on the assumption that CT slice thickness is 5 mm or less. When CT scans have slice thickness greater than 5 mm, the minimum size for a measurable lesion should be twice the slice thickness. MRI is also acceptable.

If prior to enrollment it is known that a patient is unable to undergo CT sc ans with intravenous (IV) contrast due to allergy or renal insufficiency, the decision as to whether a non-contrast CT or MRI (without IV contrast) will be used to evaluate the patient at baseline and during the study should be guided by the tumor type under investigation and the anatomic location of the disease. For patients who develop contraindications to contrast after baseline contrast CT is done, the decision as to whether non-contrast CT or MRI (enhanced or non-enhanced) will be performed should also be based on the tumor type and the anatomic location of the disease and should be optimized to allow for comparison with the prior studies if possible. Each case should be discussed with the radiologist to determine if substitution of these other approaches is possible and, <u>if not, the patient should be considered not evaluable from that point forward.</u> Care must be taken in measurement of target lesions on a different modality and interpretation of non-target disease or new lesions since the same lesion may appear to have a different size using a new modality.

Ultrasound. Ultrasound is not useful in assessment of lesion size and should not be used as a method of measurement.

Endoscopy, Laparoscopy, Tumor Markers, Cytology, Histology. The utilization of these techniques for objective tumor evaluation cannot generally be advised.

<u>Tumor Response Evaluation</u>

Assessment of Overall Tumor Burden and Measurable Disease

To assess objective response or future progression, it is necessary to estimate the overall tumor burden at baseline and to use this as a comparator for subsequent measurements. Measurable disease is defined by the presence of at least one measurable lesion, as detailed above.

Baseline Documentation of Target and Non-Target Lesions

When more than one measurable lesion is present at baseline, all lesions up to a maximum of five lesions total (and a maximum of two lesions per organ) representative of all involved organs should be identified as target lesions and will be recorded and measured at baseline. This means in instances where patients have only one or two organ sites involved, a maximum of two lesions (one site) and four lesions (two sites), respectively, will be recorded. Other lesions (albeit measurable) in those organs will be recorded as non-measurable lesions (even if the size is □ 10 mm by CT scan).

Target lesions should be selected on the basis of their size (lesions with the longest diameter) and be representative of all involved organs, but additionally, should lend themselves to reproducible repeated measurements. It may be the case that, on occasion, the largest lesion does not lend itself to reproducible measurement, in which circumstance the next largest lesion that can be measured reproducibly should be selected.

Lymph nodes merit special mention since they are normal anatomical structures that may be visible by imaging even if not involved by tumor. As noted above, pathological nodes that are defined as measurable and may be identified as target lesions must meet the criterion of a short axis of □ 15 mm by CT scan. Only the short axis of these nodes will contribute to the baseline sum. The short axis of the node is the diameter normally used by radiologists to judge if a node is involved by solid tumor. Nodal size is normally reported as two dimensions in the plane in which the image is obtained (for CT scan, this is almost always the axial plane; for MRI the plane of acquisition may be axial, sagittal, or coronal). The smaller of these measures is the short axis. For example, an abdominal node that is reported as being 20 mm □ 30 mm has a short axis of 20 mm and qualifies as a malignant, measurable node. In this example, 20 mm should be recorded as the node measurement. All other pathological nodes (those with short axis □ 10 mm but □ 15 mm) should be considered non-target lesions. Nodes that have a short axis □ 10 mm are considered non-pathological and should not be recorded or followed.

A sum of the diameters (longest for non-nodal lesions, short axis for nodal lesions) for all target lesions will be calculated and reported as the baseline sum of diameters. If lymph nodes are to be included in the sum, then, as noted above, only the short axis is added into the sum. The baseline sum of diameters will be used as a reference to further characterize any objective tumor regression in the measurable dimension of the disease.

All other lesions (or sites of disease), including pathological lymph nodes, should be identified as non-target lesions and should also be recorded at baseline. Measurements are not required and these lesions should be followed as “present,” “absent,” or in rare cases “unequivocal progression.”

In addition, it is possible to record multiple non-target lesions involving the same organ as a single item on the Case Report Form (CRF) (e.g., “multiple enlarged pelvic lymph nodes” or “multiple liver metastases”).

Response Criteria

a. Evaluation of Target Lesions

This section provides the definitions of the criteria used to determine objective tumor response for target lesions.

- Complete response (CR): disappearance of all target lesions

  Any pathological lymph nodes (whether target or non-target) must have reduction in short axis to □ 10 mm.

- Partial response (PR): at least a 30% decrease in the sum of diameters of target lesions, taking as reference the baseline sum of diameters
- Progressive disease (PD): at least a 20% increase in the sum of diameters of target lesions, taking as reference the smallest sum on study (nadir), including baseline

  In addition to the relative increase of 20%, the sum must also demonstrate an absolute increase of at least 5 mm.

  The appearance of one or more new lesions is also considered progression.

- Stable disease (SD): neither sufficient shrinkage to qualify for PR nor sufficient increase to qualify for PD, taking as reference the smallest sum on study

b. Special Notes on the Assessment of Target Lesions

Lymph Nodes. Lymph nodes identified as target lesions should always have the actual short axis measurement recorded (measured in the same anatomical plane as the baseline examination), even if the nodes regress to □ 10 mm on study. This means that when lymph nodes are included as target lesions, the sum of lesions may not be zero even if CR criteria are met since a normal lymph node is defined as having a short axis □ 10 mm.

Target Lesions That Become Too Small to Measure. While on study, all lesions (nodal and non-nodal) recorded at baseline should have their actual measurements recorded at each subsequent evaluation, even when very small (e.g., 2 mm). However, sometimes lesions or lymph nodes that are recorded as target lesions at baseline become so faint on CT scan that the radiologist may not feel comfortable assigning an exact measure and may report them as being too small to measure. When this occurs, it is important that a value be recorded on the CRF as follows:

- If it is the opinion of the radiologist that the lesion has likely disappeared, the measurement should be recorded as 0 mm.
- If the lesion is believed to be present and is faintly seen but too small to measure, a default value of 5 mm should be assigned and BML (below measurable limit) should be ticked. (Note: It is less likely that this rule will be used for lymph nodes since they usually have a definable size when normal and are frequently surrounded by

fat such as in the retroperitoneum; however, if a lymph node is believed to be present and is faintly seen but too small to measure, a default value of 5 mm should be assigned in this circumstance as well and BML should also be ticked.)

<u>To reiterate, however, if the radiologist is able to provide an actual measure, that should be recorded, even if it is below 5 mm, and, in that case, BML should not be ticked.</u>

Lesions That Split or Coalesce on Treatment. When non-nodal lesions fragment, the longest diameters of the fragmented portions should be added together to calculate the target lesion sum. Similarly, as lesions coalesce, a plane between them may be maintained that would aid in obtaining maximal diameter measurements of each individual lesion. If the lesions have truly coalesced such that they are no longer separable, the vector of the longest diameter in this instance should be the maximal longest diameter for the coalesced lesion.

c. Evaluation of Non-Target Lesions

This section provides the definitions of the criteria used to determine the tumor response for the group of non-target lesions. While some non-target lesions may actually be measurable, they need not be measured and, instead, should be assessed only qualitatively at the time points specified in the protocol.

- CR: disappearance of all non-target lesions and (if applicable) normalization of tumor marker level)

  All lymph nodes must be non-pathological in size (□ 10 mm short axis).
- Non-CR/Non-PD: persistence of one or more non-target lesion(s) and/or (if applicable) maintenance of tumor marker level above the normal limits
- PD: unequivocal progression of existing non-target lesions

  The appearance of one or more new lesions is also considered progression.

d. Special Notes on Assessment of Progression of Non-Target Disease

When the Patient Also Has Measurable Disease. In this setting, to achieve unequivocal progression on the basis of the non-target disease, there must be an overall level of substantial worsening in non-target disease in a magnitude that, even in the presence of SD or PR in target disease, the overall tumor burden has increased sufficiently to merit discontinuation of therapy. A modest increase in the size of one or more non-target lesions is usually not sufficient to qualify for unequivocal progression status. The designation of overall progression solely on the basis of change in non-target disease in the face of SD or PR of target disease will therefore be extremely rare.

When the Patient Has Only Non-Measurable Disease. This circumstance arises in some Phase III trials when it is not a criterion of study entry to have measurable disease. The same general concepts apply here as noted above; however, in this instance, there is no measurable disease assessment to factor into the interpretation of an

increase in non-measurable disease burden. Because worsening in non-target disease cannot be easily quantified (by definition: if all lesions are truly non-measurable), a useful test that can be applied when assessing patients for unequivocal progression is to consider if the increase in overall disease burden based on the change in non-measurable disease is comparable in magnitude to the increase that would be required to declare PD for measurable disease, that is, an increase in tumor burden representing an additional 73% increase in volume (which is equivalent to a 20% increase in diameter in a measurable lesion). Examples include an increase in a pleural effusion from "trace" to "large" or an increase in lymphangitic disease from localized to widespread or may be described in protocols as "sufficient to require a change in therapy." If unequivocal progression is seen, the patient should be considered to have had overall PD at that point. While it would be ideal to have objective criteria to apply to non-measurable disease, the very nature of that disease makes it impossible to do so; therefore, the increase must be substantial.

e. New Lesions

The appearance of new malignant lesions denotes disease progression; therefore, some comments on detection of new lesions are important. There are no specific criteria for the identification of new radiographic lesions; however, the finding of a new lesion should be unequivocal, that is, not attributable to differences in scanning technique, change in imaging modality, or findings thought to represent something other than tumor (for example, some "new" bone lesions may be simply healing or flare of pre-existing lesions). This is particularly important when the patient's baseline lesions show partial or complete response. For example, necrosis of a liver lesion may be reported on a CT scan report as a "new" cystic lesion, which it is not.

A lesion identified during the study in an anatomical location that was not scanned at baseline is considered a new lesion and will indicate disease progression.

If a new lesion is equivocal, for example because of its small size, continued therapy and follow-up evaluation will clarify if it represents truly new disease. If repeat scans confirm there is definitely a new lesion, then progression should be declared using the date of the initial scan.

Evaluation of Response

a. Timepoint Response (Overall Response)

It is assumed that at each protocol-specified timepoint, a response assessment occurs. Table 1 provides a summary of the overall response status calculation at each timepoint for patients who have measurable disease at baseline.

When patients have non-measurable (therefore non-target) disease only, Table 2 is to be used.

Table 1. Timepoint Response: Patients with Target Lesions (with or without Non-Target Lesions)

Target Lesions	Non-Target Lesions	New Lesions	Overall Response
CR	CR	No	CR
CR	Non-CR/non-PD	No	PR
CR	Not evaluated	No	PR
PR	Non-PD or not all evaluated	No	PR
SD	Non-PD or not all evaluated	No	SD
Not all evaluated	Non-PD	No	NE
PD	Any	Yes or no	PD
Any	PD	Yes or no	PD
Any	Any	Yes	PD

CR = complete response; NE = not evaluable; PD = progressive disease; PR = partial response; SD = stable disease.

Table 2. Timepoint Response: Patients with Non-Target Lesions Only

Non-Target Lesions	New Lesions	Overall Response
CR	No	CR
Non-CR/non-PD	No	Non-CR/non-PD [a]
Not all evaluated	No	NE
Unequivocal PD	Yes or no	PD
Any	Yes	PD

CR = complete response; NE = not evaluable; PD = progressive disease.

[a] "Non-CR/non-PD" is preferred over "stable disease" for non-target disease since stable disease is increasingly used as an endpoint for assessment of efficacy in some trials; thus, assigning "stable disease" when no lesions can be measured is not advised.

b. Missing Assessments and Not-Evaluable Designation

When no imaging/measurement is done at all at a particular timepoint, the patient is not evaluable at that timepoint. If only a subset of lesion measurements are made at an assessment, usually the case is also considered not evaluable at that timepoint, unless a convincing argument can be made that the contribution of the individual missing lesion(s) would not change the assigned timepoint response. This would be most likely to happen in the case of PD. For example, if a patient had a baseline sum of 50 mm with three measured lesions and, during the study, only two lesions were assessed, but those gave a sum of 80 mm; the patient will have achieved PD status, regardless of the contribution of the missing lesion.

If one or more target lesions were not assessed either because the scan was not done or the scan could not be assessed because of poor image quality or obstructed view, the response for target lesions should be “unable to assess” since the patient is not evaluable. Similarly, if one or more non-target lesions are not assessed, the response for non-target lesions should be “unable to assess” except where there is clear progression. Overall response would be “unable to assess” if either the target response or the non-target response is “unable to assess,” except where this is clear evidence of progression as this equates with the case being not evaluable at that timepoint.

Table 3. Best Overall Response When Confirmation Is Required

Overall Response at First Timepoint	Overall Response at Subsequent Timepoint	Best Overall Response
CR	CR	CR
CR	PR	SD, PD, or PR [a]
CR	SD	SD, provided minimum duration for SD was met; otherwise, PD
CR	PD	SD, provided minimum duration for SD was met; otherwise, PD
CR	NE	SD, provided minimum duration for SD was met; otherwise, NE
PR	CR	PR
PR	PR	PR
PR	SD	SD
PR	PD	SD, provided minimum duration for SD was met; otherwise, PD
PR	NE	SD, provided minimum duration for SD was met; otherwise, NE
NE	NE	NE

CR = complete response; NE = not evaluable; PD = progressive disease; PR = partial response; SD = stable disease.

[a] If a CR is truly met at the first timepoint, any disease seen at a subsequent timepoint, even disease meeting PR criteria relative to baseline, qualifies as PD at that point (since disease must have reappeared after CR). Best response would depend on whether the minimum duration for SD was met. However, sometimes CR may be claimed when subsequent scans suggest small lesions were likely still present and in fact the patient had PR, not CR, at the first timepoint. Under these circumstances, the original CR should be changed to PR and the best response is PR.

c. Special Notes on Response Assessment

When nodal disease is included in the sum of target lesions and the nodes decrease to “normal” size (□ 10 mm), they may still have a measurement reported on scans. This measurement should be recorded even though the nodes are normal in order not to overstate progression should it be based on increase in size of the nodes. As noted earlier, this means that patients with CR may not have a total sum of “zero” on the CRF.

Patients with a global deterioration of health status requiring discontinuation of treatment without objective evidence of disease progression at that time should be reported as “symptomatic deterioration.” Every effort should be made to document objective progression even after discontinuation of treatment. Symptomatic deterioration is not a descriptor of an objective response; it is a reason for stopping study therapy. The objective response status of such patients is to be determined by evaluation of target and non-target disease as shown in Tables 1 − 3.

For equivocal findings of progression (e.g., very small and uncertain new lesions; cystic changes or necrosis in existing lesions), treatment may continue until the next scheduled assessment. If at the next scheduled assessment progression is confirmed, the date of progression should be the earlier date when progression was suspected.

In studies for which patients with advanced disease are eligible (i.e., primary disease still or partially present), the primary tumor should also be captured as a target or non-target lesion, as appropriate. This is to avoid an incorrect assessment of complete response if the primary tumor is still present but not evaluated as a target or non-target lesion.

Appendix 5 Immune-Related Response Criteria

INTRODUCTION

Increasing clinical experience indicates that traditional response criteria (e.g., Response Evaluation Criteria in Solid Tumors, Version 1.1 [RECIST v1.1] and World Health Organization [WHO]) may not be sufficient to characterize fully activity in the new era of target therapies and/or biologics. In studies with cytokines, cancer vaccines, and monoclonal antibodies, complete response, partial response, or stable disease has been shown to occur after an increase in tumor burden as characterized by progressive disease by traditional response criteria. Therefore, conventional response criteria may not adequately assess the activity of immunotherapeutic agents because progre ssive disease (by initial radiographic evaluation) does not necessarily reflect therapeutic failure. Long-term effect on the target disease must also be captured. The immune-related response criteria[3] (irRC) are criteria that attempt to do that by enhancing characterization of new response patterns that have been observed with immunotherapeutic agents (i.e., ipilimumab). (Note: The irRC only index and measurable new lesions are taken into account.)

GLOSSARY

Term	Definition
SPD	sum of the products of the two largest perpendicular diameters
Tumor burden	$SPD_{index\ lesions} + SPD_{new,\ measurable\ lesions}$
Nadir	minimally recorded tumor burden
irCR	immune-related complete response
irPD	immune-related progressive disease
irPR	immune-related partial response
irSD	immune-related stable disease
irBOR	immune-related best overall response

BASELINE ASSESSMENT USING irRC

Step 1. Identify the index lesions (five lesions per organ, up to ten visceral lesions and five cutaneous lesions).

Step 2. Calculate the SPD of all of these index lesions:

$$SPD = \sum_i \text{(Largest diameter of lesion i)} \times \text{(Second largest diameter of lesion i)}.$$

[3] Wolchok JD, Hoos A, O'Day S, et al. Guidelines for the evaluation of immune therapy activity in solid tumors: immune-related response criteria. Clin Can Res 2009;15:7412−20.

POST-BASELINE ASSESSMENTS USING irRC

Step 1. Calculate the SPD of the index lesions.

Step 2. Identify new, measurable lesions (□ 5 □ 5 mm; up to five new lesions per organ: five new cutaneous lesions and ten visceral lesions).

Step 3. Calculate the SPD of the new, measurable lesions.

Step 4. Calculate the tumor burden:

$$\text{Tumor burden} = SPD_{\text{index lesions}} + SPD_{\text{new, measurable lesions}}$$

Step 5. Calculate the change in tumor burden relative to baseline and the change in tumor burden relative to nadir.

Step 6. Derive the overall response using the table below.

Overall Response	Criterion
irCR	Complete disappearance of all lesions (whether measurable or not, and no new lesions) confirmed by a repeat, consecutive assessment □ 4 weeks from the date first documented
irPR	Decrease in tumor burden □ 50% relative to baseline confirmed by a consecutive assessment □ 4 weeks from the date first documented
irSD	Criteria for irCR, irPR, and irPD are not met; does not require confirmation
irPD	Increase in tumor burden □ 25% relative to nadir confirmed by a consecutive assessment □ 4 weeks from the date first documented

irCR = immune-related complete response; irPD = immune-related progressive disease; irPR = immune-related partial response; irSD = immune-related stable disease.

DETERMINATION OF irBOR

Once a patient has completed all tumor assessments, his/her irBOR may be determined:

Condition	irBOR
At least one irCR	irCR
At least one irPR and no irCR	irPR
At least one irSD and no irCR and no irPR	irSD
At least one irPD and no irCR, no irPR, and no irSD	irPD

irBOR = immune-related best overall response; irCR = immune-related complete response; irPD = immune-related progressive disease; irPR = immune-related partial response; irSD = immune-related stable disease.

Appendix 6 Eastern Cooperative Oncology Group (ECOG) Performance Status Scale

Grade	Description
0	Fully active, able to carry on all pre-disease performance without restriction
1	Restricted in physically strenuous activity but ambulatory and able to carry out work of a light or sedentary nature, e.g., light housework or office work
2	Ambulatory and capable of all self -care but unable to carry out any work activities; up and about □ 50% of waking hours
3	Capable of only limited self-care, confined to a bed or chair □ 50% of waking hours
4	Completely disabled; cannot carry on any self -care; totally confined to bed or chair
5	Dead

Appendix 7 Anaphylaxis Precautions

EQUIPMENT NEEDED

- Tourniquet
- Oxygen
- Epinephrine for subcutaneous, intravenous, and/or endotracheal use in accordance with standard practice
- Antihistamines
- Corticosteroids
- Intravenous infusion solutions, tubing, catheters, and tape

PROCEDURES

In the event of a suspected anaphylactic reaction during study drug infusion, the following procedures should be performed:

1. Stop the study drug infusion.
2. Apply a tourniquet proximal to the injection site to slow systemic absorption of study drug. Do not obstruct arterial flow in the limb.
3. Maintain an adequate airway.
4. Administer antihistamines, epinephrine, or other medications as required by patient status and directed by the physician in charge.
5. Continue to observe the patient and document observation.

Appendix 8 Safety Reporting Fax Cover Sheet

Genentech
A Member of the Roche Group

GENENTECH SUPPORTED RESEARCH

AE/SAE FAX No: (650) 238-6067

E-mail: usds_aereporting-d@gene.com

Page 1 of ___

Genentech Study Number	
Principal Investigator	
Site Name	
Reporter name	
Reporter Telephone #	
Reporter Fax #	
Initial Report Date	___________/_____/_____<br>dd / mmm / yyyy
Follow-up Report Date	___________/_____/_____<br>dd / mmm / yyyy
Patient Initials<br>(Please enter a dash if the patient has no middle name)	____-_____-_____

SAE or Safety Reporting questions, contact Genentech Safety: (888) 835 -2555

PLEASE PLACE MEDWATCH REPORT or SAFETY REPORT BEHIND THIS COVER SHEET.

Appendix 9 Cohort 1 (Vemurafenib/Cobimetinib/atezolizumab) AE Management

Guidelines for Management of Patients Who Experience Specific Adverse Events

Event	Action to Be Taken
**General guidance for dose modifications and treatment delays and discontinuation**	• There will be no dose modifications for atezolizumab.<br>• The dose of cobimetinib can be reduced by 20 mg (one dose level) up to two times (i.e., from 60 mg to 40 mg and then from 40 mg to 20 mg). If further dose reduction is indicated after two dose reductions, the patient must discontinue cobimetinib but may continue treatment with atezolizumab and/or vemurafenib at the investigator's discretion.<br>• For Arm A, the dose of vemurafenib can be reduced by 240 mg BID (i.e., 480 mg/day) (one dose level) up to two times (i.e., from 960 mg to 720 mg BID and then from 720 mg to 480 mg BID). For Arm B, the dose of vemurafenib can be reduced by 240 mg BID (i.e., 480 mg/day) (one dose level) one time (i.e., from 720 mg to 480 mg BID) followed by a reduction in the number of vemurafenib placebo tablets (i.e., vemurafenib dose remains at 480 mg BID) (second "dose reduction") to preserve blinding. If further dose reduction is indicated after two dose reductions, the patient must discontinue vemurafenib but may continue treatment with atezolizumab and/or cobimetinib at the investigator's discretion.<br>• If atezolizumab is withheld and corticosteroids are initiated for treatment of a toxicity, corticosteroids must be tapered over □ 1 month to □ 10 mg/day oral prednisone or equivalent before atezolizumab can be resumed. If atezolizumab is withheld for □105 days, the patient should be discontinued from atezolizumab. Study treatment may be withheld for □ 105 days to allow for patients to taper off corticosteroids prior to resuming treatment. atezolizumab can be resumed after being withheld for □105 days if the investigator and the Medical Monitor agree that the patient is likely to derive clinical benefit.<br>• If either cobimetinib or vemurafenib is withheld for □ 28 days because of toxicity, the patient should be discontinued from that drug, unless resumption of treatment is approved by the Medical Monitor after discussion with the investigator.<br>• After dose reduction, consideration may be given to allow for dose escalation of cobimetinib or vemurafenib by a maximum of one dose level following resolution of the adverse event that resulted in dose modification, provided there are no safety concerns.

BID = twice daily.

[a] If corticosteroids have been initiated, they must be tapered over □1 month to □10 mg/day oral prednisone or equivalent before atezolizumab can be resumed.

[b] atezolizumab may be withheld for a period of time beyond 12 weeks to allow for corticosteroids to be reduced to □10 mg/day oral prednisone or equivalent. The acceptable length of the extended period of time must be agreed upon by the investigator and the Medical Monitor.

[c] Resumption of atezolizumab may be considered in patients who are deriving benefit and have fully recovered from the immune-related event. Patients can be rechallenged with atezolizumab only after approval has been documented by both the investigator (or an appropriate delegate) and the Medical Monitor.

Guidelines for Management of Patients Who Experience Specific Adverse Events (cont.)

Event	Action to Be Taken
**IRRs, anaphylaxis, and hypersensitivity reaction**	• Guidelines for management of IRRs are provided in the atezolizumab Investigator's Brochure for atezolizumab.<br>• For anaphylaxis precautions, see **Error! Reference source not found.**.<br>• For severe hypersensitivity reactions, permanently discontinue all study treatment.
**Gastrointestinal toxicity**	
Gastrointestinal event s: general guidance	• All events of diarrhea or colitis should be thoroughly evaluated for other more common etiologies.<br>• For events of significant duration or magnitude or associated with signs of systemic inf lammation or acute phase reactants (e.g., increased CRP, platelet count, or bandemia): Perform sigmoidoscopy (or colonoscopy, if appropriate) with colonic biopsy, with three to five specimens for standard paraffin block to check for inf lammation and lymphocytic infiltrates to confirm colitis diagnosis.
Abdominal pain in combination with blood or mucus in stool<br>**<u>and/or</u>**<br>Grade □ 3 diarrhea with possible colitis (e.g., peritoneal signs, ileus, or fever)	• Withhold all study treatment.<br>• Discontinue medications that may exacerbate colitis (e.g., NSAIDS).<br>• Initiate maximum supportive care and monitor patient closely.<br>• Investigate etiology, ruling out bowel perforation. Refer patient to GI specialist.<br>• If immune-related colitis is suspected, consider treatment with 1−2 mg/kg/day IV methylprednisolone or equivalent and convert to 1−2 mg/kg/day oral prednisone or equivalent upon improvement. Consider TNF antagonists for refractory diarrhea.<br>• If event resolves to Grade 1 or better within 12 weeks, resume atezolizumab at fixed dose. If not, permanently discontinue atezolizumab. [a,b,c]<br>• If event resolves to Grade 1 or better within 28 days, resume cobimetinib and vemurafenib with doses reduced by one level. If not, permanently discontinue cobimetinib and vemurafenib.<br>• When study treatment is resumed, administer supportive care and/or prophylactic treatment.

CRP = C-reactive protein; GI = gastrointestinal; IV = intravenous; NSAID = non-steroidal anti-inflammatory drug; TNF = tumor necrosis factor.

[a] If corticosteroids have been initiated, they must be tapered over □1 month to □10 mg/day oral prednisone or equivalent before atezolizumab can be resumed.

[b] atezolizumab may be withheld for a period of time beyond 12 weeks to allow for corticosteroids to be reduced to □10 mg/day oral prednisone or equivalent. The acceptable length of the extended period of time must be agreed upon by the investigator and the Medical Monitor.

[c] Resumption of atezolizumab may be considered in patients who are deriving ben efit and have fully recovered from the immune-related event. Patients can be rechallenged with atezolizumab only after approval has been documented by both the investigator (or an appropriate delegate) and the Medical Monitor.

Guidelines for Management of Patients Who Experience Specific Adverse Events (cont.)

Event	Action to Be Taken
**Gastrointestinal toxicity (cont.)**	
Diarrhea, Grade 1 or 2	• Continue all study treatment.<br>• Initiate maximum supportive care and monitor patient closely.<br>• Investigate etiology, referring patient to GI specialist for evaluation of possible colitis if appropriate.
Diarrhea, Grade 3	• Withhold all study treatment.<br>• Discontinue medications that may exacerbate colitis (e.g., NSAIDS) while investigating etiology.<br>• Initiate maximum supportive care and monitor patient closely.<br>• Investigate etiology, referring patient to GI specialist for evaluation of possible colitis, including biopsy if appropriate.<br>• If event resolves to Grade 1 or better within 12 weeks, resume atezolizumab at fixed dose. If not, permanently discontinue atezolizumab. [a,b,c]<br>• If event resolves to Grade 1 or better within 28 days, resume cobimetinib and vemurafenib with doses reduced by one level. If not, permanently discontinue cobimetinib and vemurafenib.
Diarrhea, Grade 4	• Permanently discontinue all study treatment and contact Medical Monitor. [c]<br>• Discontinue medications that may exacerbate colitis (e.g., NSAIDS) while investigating etiology.<br>• Initiate maximum supportive care and monitor patient closely.<br>• Investigate etiology, referring patient to GI specialist for evaluation of possible colitis, including biopsy if appropriate.<br>• Initiate treatment with 1−2 mg/kg/day IV methylprednisolone or equivalent and convert to 1−2 mg/kg/day oral prednisone or equivalent upon improvement.<br>• If event does not improve within 48 hours after initiating corticosteroids, consider adding an immunosuppressive agent.<br>• If event resolves to Grade 1 or better, taper corticosteroids over □ 1 month

GI = gastrointestinal; IV = intravenous; NSAID = non-steroidal anti-inflammatory drug.

[a] If corticosteroids have been initiated, they must be tapered over □1 month to □10 mg/day oral prednisone or equivalent before atezolizumab can be resumed.

[b] atezolizumab may be withheld for a period of time beyond 12 weeks to allow for corticosteroids to be reduced to □10 mg/day oral prednisone or equivalent. The acceptable length of the extended period of time must be agreed upon by the investigator and the Medical Monitor.

[c] Resumption of atezolizumab may be considered in patients who are deriving benefit and have fully recovered from the immune-related event. Patients can be rechallenged with atezolizumab only after approval has been documented by both the investigator (or an appropriate delegate) and the Medical Monitor.

Guidelines for Management of Patients Who Experience Specific Adverse Events (cont.)

Event	Action to Be Taken
**New skin lesion**	
Cutaneous primary malignancy or skin lesion suggestive of cutaneous primary malignancy	• Continue all study treatment.<br>• Biopsy or excise lesion and submit specimen, along with paired normal skin sample (one sample per patient) to central laboratory.<br>• Treat per institutional guidelines.
**Dermatolgic toxicity**	
General guidance	• A dermatologist should evaluate persistent and/or severe rash or pruritus. A biopsy should be considered unless contraindicated.
Photosensitivity, Grade 1	• Continue all study treatment.<br>• Initiate supportive care.

[a] If corticosteroids have been initiated, they must be tapered over [illegible]1 month to [illegible]10 mg/day oral prednisone or equivalent before atezolizumab can be resumed.

[b] atezolizumab may be withheld for a period of time beyond 12 weeks to allow for corticosteroids to be reduced to [illegible]10 mg/day oral prednisone or equivalent. The acceptable length of the extended period of time must be agreed upon by the investigator and the Medical Monitor.

[c] Resumption of atezolizumab may be considered in patients who are deriving benefit and have fully recovered from the immune-related event. Patients can be rechallenged with atezolizumab only after approval has been documented by both the investigator (or an appropriate delegate) and the Medical Monitor.

Guidelines for Management of Patients Who Experience Specific Adverse Events (cont.)

Event	Action to Be Taken
**Dermatologic toxicity (cont.)**	
Photosensitivity, Grade 2	• Continue atezolizumab.<br>• Initiate supportive care per institutional guidelines.<br>**First episode:**<br>• If event does not resolve to Grade 1 or better within 7 days, withhold cobimetinib and vemurafenib.<br>• If treatment is withheld and event resolves to Grade 1 or better within 28 days, resume cobimetinib at current dose and resume vemurafenib with dose reduced by one level.<br>• If event does not resolve to Grade 1 or better within 28 days, permanently discontinue cobimetinib and vemurafenib.<br>**Subsequent episodes:**<br>• Withhold cobimetinib and vemurafenib.<br>• If event resolves to Grade 1 or better within 28 days, resume cobimetinib at current dose and resume vemurafenib with dose reduced by one level.<br>• If event does not resolve to Grade 1 or better within 28 days, permanently discontinue cobimetinib and vemurafenib.
Photosensitivity, Grade 3	• Withhold cobimetinib and vemurafenib. Continue atezolizumab.<br>• Initiate supportive care per institutional guidelines.<br>• If event resolves to Grade 1 or better within 28 days, resume cobimetinib at current dose and resume vemurafenib with dose reduced by one level. If not, permanently discontinue cobimetinib and vemurafenib.
Photosensitivity, Grade 4	• Initiate supportive care per institutional guidelines.<br>• If event resolves to Grade 1 or better within 28 days, resume cobimetinib at current dose and resume vemurafenib with dose reduced by one level. If not, permanently discontinue cobimetinib and vemurafenib.<br>• Continue atezolizumab if clinically indicated.

[a] If corticosteroids have been initiated, they must be tapered over □1 month to □10 mg/day oral prednisone or equivalent before atezolizumab can be resumed.

[b] atezolizumab may be withheld for a period of time beyond 12 weeks to allow for corticosteroids to be reduced to □10 mg/day oral prednisone or equivalent. The acceptable length of the extended period of time must be agreed upon by the investigator and the Medical Monitor.

[c] Resumption of atezolizumab may be considered in patients who are deriving benefit and have fully recovered from the immune-related event. Patients can be rechallenged with atezolizumab only after approval has been documented by both the investigator (or an appropriate delegate) and the Medical Monitor.

Guidelines for Management of Patients Who Experience Specific Adverse Events (cont.)

Event	Action to Be Taken
**Dermatologic toxicity (cont.)**	
Dermatologic event, Grade 1	• Continue all study treatment.<br>• Initiate maximum supportive care (e.g., antihistamines, topical corticosteroids).
Dermatologic event, Grade 2	• Continue all study treatment.<br>• Consider patient referral to dermatologist.<br>• Initiate maximum supportive care (e.g., antihistamines, topical corticosteroids). If event does not improve, consider treatment with higher-potency topical corticosteroids.
Dermatologic event, Grade 3	• Refer patient to dermatologist. A biopsy should be performed if appropriate, and, if possible, photographs of the rash should be obtained and submitted to Genentech.<br>**Acneiform rash:**<br>• Withhold atezolizumab and cobimetinib. Continue vemurafenib.<br>• Refer patient to dermatologist.<br>• If event does not improve within 48−72 hours, consider treatment with 10 mg/day oral prednisone or equivalent, increasing dose to 1−2 mg/kg/day if event still does not improve.<br>• If event resolves to Grade 2 or better within 12 weeks, resume atezolizumab at fixed dose. If not, permanently discontinue atezolizumab. [a,b,c]<br>• If event resolves to Grade 2 or better within 28 days, resume cobimetinib with dose reduced by one level. If not, permanently discontinue cobimetinib.<br>• When study treatment is resumed, consider treatment with topical corticosteroids and oral antibiotics.<br>**Non-acneiform (e.g., maculo-papular) rash:**<br>• Withhold vemurafenib. Continue atezolizumab and cobimetinib.<br>• If event resolves to Grade 2 or better within 28 days, resume vemurafenib with dose reduced by one level. If not, permanently discontinue vemurafenib.

[a] If corticosteroids have been initiated, they must be tapered over □1 month to □10 mg/day oral prednisone or equivalent before atezolizumab can be resumed.

[b] atezolizumab may be withheld for a period of time beyond 12 weeks to allow for corticosteroids to be reduced to □10 mg/day oral prednisone or equivalent. The acceptable length of the extended period of time must be agreed upon by the investigator and the Medical Monitor.

[c] Resumption of atezolizumab may be considered in patients who are deriving benefit and have fully recovered from the immune-related event. Patients can be rechallenged with atezolizumab only after approval has been documented by both the investigator (or an appropriate delegate) and the Medical Monitor.

Guidelines for Management of Patients Who Experience Specific Adverse Events (cont.)

Event	Action to Be Taken
**Dermatologic toxicity (cont.)**	
Dermatologic event, Grade 4	• Permanently discontinue all study treatment and contact Medical Monitor. [c]
**Elevations in ALT, AST, and/or bilirubin <u>during run-in period</u>**	
Elevation in ALT, AST, and/or bilirubin, Grade 1 or 2 (run-in period)	• Continue cobimetinib and vemurafenib.
Elevation in ALT, AST, and/or bilirubin, Grade 3 (run-in period)	• Withhold vemurafenib. Continue cobimetinib.<br>• If event resolves to Grade 1 or better within 28 days, resume vemurafenib with dose reduced by one level. If not, discontinue patient from study.
Elevation in ALT, AST, and/or bilirubin, Grade 4 (run-in period)	**First episode:**<br>• Withhold cobimetinib and vemurafenib.<br>• If event resolves to Grade 1 or better within 28 days, resume cobimetinib and vemurafenib with doses reduced by one level. If not, discontinue patient from study.<br>**Subsequent episodes:**<br>• Discontinue patient from study.
**Elevations in ALT, AST, and/or bilirubin <u>during triple combination period</u>**	
ALT, AST, and bilirubin [illegible] 3 [illegible] ULN (triple combination period)	• Continue all study treatment.<br>• Monitor ALT, AST, and bilirubin at least weekly.

ULN = upper limit of normal.

[a] If corticosteroids have been initiated, they must be tapered over [illegible] 1 month to [illegible] 10 mg/day oral prednisone or equivalent before atezolizumab can be resumed.

[b] atezolizumab may be withheld for a period of time beyond 12 weeks to allow for corticosteroids to be reduced to [illegible] 10 mg/day oral prednisone or equivalent. The acceptable length of the extended period of time must be agreed upon by the investigator and the Medical Monitor.

[c] Resumption of atezolizumab may be considered in patients who are deriving ben efit and have fully recovered from the immune-related event. Patients can be rechallenged with atezolizumab only after approval has been documented by both the investigator (or an appropriate delegate) and the Medical Monitor.

Guidelines for Management of Patients Who Experience Specific Adverse Events (cont.)

Event	Action to Be Taken
**Elevations in ALT, AST, and/or bilirubin <u>during triple combination period</u> (cont.)**	
ALT or AST □ 3 □ upper limit of normal (ULN) to 5 □ ULN in combination with bilirubin □ 3 □ ULN (triple combination period)	• Consider withholding all study treatment.<br>• Monitor ALT, AST, and bilirubin at least weekly. If values worsen, monitor at least every other day.<br>• If values have not worsened and have not resolved to □ 3 □ ULN upon re-evaluation 1−20 days after event onset, consider treatment with 1−2 mg/kg/day oral prednisone or equivalent.<br>• If values have not worsened and have not resolved to □ 3 □ ULN upon re-evaluation □ 21 days after event onset, initiate treatment with 1−2 mg/kg/day oral prednisone or equivalent.<br>• If values have worsened upon re-evaluation, initiate treatment with 1−2 mg/kg/day IV methylprednisolone or equivalent and convert to 1−2 mg/kg/day oral prednisone or equivalent upon improvement. If values do not improve within 48 hours after initiating corticosteroids, consider adding an immunosuppressive agent.<br>• If treatment is withheld and values resolve to □ 3 □ ULN before or within 12 weeks after initiation of corticosteroids, resume atezolizumab at fixed dose. [a,b]<br>• If values do not resolve to □ 3 □ ULN within 12 weeks after initiation of corticosteroids, permanently discontinue atezolizumab. [a,b,c]<br>• If treatment is withheld and values resolve to □ 3 □ ULN before or within 28 days (with or without corticosteroids), resume cobimetinib and vemurafenib as follows:<br>− If values did not worsen prior to resolution, resume cobimetinib and vemurafenib at current doses.<br>− If values worsened prior to resolution:<br><u>First Episode</u>: Resume cobimetinib at current dose and resume vemurafenib with dose reduced by one level.<br><u>Second Episode</u>: Resume cobimetinib and vemurafenib with doses reduced by one level.<br><u>Third Episode</u>: Resume cobimetinib with dose reduced by one level and permanently discontinue vemurafenib.<br><u>Fourth Episode</u>: Permanently discontinue cobimetinib.<br>• If values do not resolve to □ 3 □ ULN within 28 days after initiation of corticosteroids, permanently discontinue cobimetinib and vemurafenib.

[a] If corticosteroids have been initiated, they must be tapered over □1 month to □10 mg/day oral prednisone or equivalent before atezolizumab can be resumed.

[b] atezolizumab may be withheld for a period of time beyond 12 weeks to allow for corticosteroids to be reduced to □10 mg/day oral prednisone or equivalent. The acceptable length of the extended period of time must be agreed upon by the investigator and the Medical Monitor.

[c] Resumption of atezolizumab may be considered in patients who are deriving benefit and have fully recovered from the immune-related event. Patients can be rechallenged with atezolizumab only after approval has been documented by both the investigator (or an appropriate delegate) and the Medical Monitor.

Guidelines for Management of Patients Who Experience Specific Adverse Events (cont.)

Event	Action to Be Taken
**Elevations in ALT, AST, and/or bilirubin <u>during triple combination period</u> (cont.)**	
ALT or AST □ 5 □ ULN to 10 □ ULN in combination with bilirubin □ 1.5 □ ULN (triple combination period)	• Withhold all study treatment.<br>• Monitor ALT, AST, and bilirubin at least every other day.<br>• If values do not improve within 48 hours, initiate treatment with 1−2 mg/kg/day IV methylprednisolone or equivalent and convert to 1−2 mg/kg/day oral prednisone or equivalent upon improvement. If values do not improve within 48 hours after initiating corticosteroids, consider adding an immunosuppressive agent.<br>• If values improve within 48 hours but subsequently worsen or do not resolve to □ 3 □ ULN within 14 days after event onset, initiate treatment with 1−2 mg/kg/day IV methylprednisolone or equivalent and convert to 1−2 mg/kg/day oral prednisone or equivalent upon improvement. If values do not improve within 48 hours after initiating corticosteroids, consider adding an immunosuppressive agent.<br>• If values resolve to □ 3 □ ULN before or within 12 weeks after initiation of corticosteroids, resume atezolizumab at fixed dose.[a,b]<br>• If values do not resolve to □ 3 □ ULN within 12 weeks after initiation of corticosteroids, permanently discontinue atezolizumab.[a,b,c]<br>• If values resolve to □ 3 □ ULN before or within 28 days after initiation of corticosteroids, resume cobimetinib and vemurafenib as follows:<br><u>First Episode</u>: Resume cobimetinib at current dose and resume vemurafenib with dose reduced by one level.<br><u>Second Episode</u>: Resume cobimetinib and vemurafenib with doses reduced by one level.<br><u>Third Episode</u>: Resume cobimetinib with dose reduced by one level and permanently discontinue vemurafenib.<br><u>Fourth Episode</u>: Permanently discontinue cobimetinib.<br>• If values do not resolve to □ 3 □ ULN within 28 days after initiation of corticosteroids, permanently discontinue cobimetinib and vemurafenib.

ULN = upper limit of normal.

[a] If corticosteroids have been initiated, they must be tapered over □1 month to □10 mg/day oral prednisone or equivalent before atezolizumab can be resumed.

[b] atezolizumab may be withheld for a period of time beyond 12 weeks to allow for corticosteroids to be reduced to □10 mg/day oral prednisone or equivalent. The acceptable length of the extended period of time must be agreed upon by the investigator and the Medical Monitor.

[c] Resumption of atezolizumab may be considered in patients who are deriving ben efit and have fully recovered from the immune-related event. Patients can be rechallenged with atezolizumab only after approval has been documented by both the investigator (or an appropriate delegate) and the Medical Monitor.

Guidelines for Management of Patients Who Experience Specific Adverse Events (cont.)

Event	Action to Be Taken
**Elevations in ALT, AST, and/or bilirubin <u>during triple combination period</u> (cont.)**	
ALT or AST □ 5 □ ULN to 10 □ ULN in combination with bilirubin □ 1.5 □ ULN to 3 □ ULN<br>**<u>or</u>**<br>ALT or AST □ 10 □ ULN in combination with bilirubin □ 3 □ ULN (triple combination period)	• Withhold all study treatment.<br>• Initiate treatment with 1−2 mg/kg/day IV methylprednisolone or equivalent and convert to 1−2 mg/kg/day oral prednisone or equivalent upon improvement. If values do not improve within 48 hours after initiating corticosteroids, consider adding an immunosuppressive agent.<br>• Monitor ALT, AST, and bilirubin at least every other day.<br>• If values resolve to □ 3 □ ULN within 12 weeks, resume atezolizumab at fixed dose. If not, permanently discontinue atezolizumab.[a,b,c]<br>• If values resolve to □ 3 □ ULN within 28 days, resume cobimetinib at current dose and resume vemurafenib with dose reduced by one level. If not, permanently discontinue cobimetinib and vemurafenib.
ALT □ 10 □ ULN with or without elevation of AST or bilirubin<br>**<u>or</u>**<br>Confirmed cases of Hy's law with no identifiable cause other than study treatment (triple combination period)	• Permanently discontinue all study treatment and contact Medical Monitor.[c]<br>• Initiate treatment with 1−2 mg/kg/day IV methylprednisolone or equivalent and convert to 1−2 mg/kg/day oral prednisone or equivalent upon improvement. If values do not improve within 48 hours after initiating corticosteroids, consider adding an immunosuppressive agent.

IV = intravenous; ULN = upper limit of normal.

[a] If corticosteroids have been initiated, they must be tapered over □1 month to □10 mg/day oral prednisone or equivalent before atezolizumab can be resumed.

[b] atezolizumab may be withheld for a period of time beyond 12 weeks to allow for corticosteroids to be reduced to □10 mg/day oral prednisone or equivalent. The acceptable length of the extended period of time must be agreed upon by the investigator and the Medical Monitor.

[c] Resumption of atezolizumab may be considered in patients who are deriving ben efit and have fully recovered from the immune-related event. Patients can be rechallenged with atezolizumab only after approval has been documented by both the investigator (or an appropriate delegate) and the Medical Monitor.

Guidelines for Management of Patients Who Experience Specific Adverse Events (cont.)

Event	Action to Be Taken
**Pulmonary events**	
Pulmonary event, Grade 1	• Continue all study treatment and monitor closely.<br>• Re-evaluate on serial imaging.<br>• Consider patient referral to pulmonary specialist.<br>• For recurrent events, treat as a Grade 3 or 4 event.
Pulmonary event, Grade 2	• Withhold atezolizumab. Consider withholding cobimetinib and vemurafenib.<br>• Refer patient to pulmonary and infectious disease specialists and consider bronchoscopy or BAL.<br>• Initiate treatment with 1−2 mg/kg/day oral prednisone or equivalent.<br>• If event resolves to Grade 1 or better within 12 weeks, resume atezolizumab at fixed dose. If not, permanently discontinue atezolizumab and contact Medical Monitor. [a,b,c]<br>• If cobimetinib and vemurafenib are withheld and event resolves to Grade 1 or better within 28 days, resume cobimetinib and vemurafenib at current doses.<br>• If event does not resolve to Grade 1 or better within 28 days, permanently discontinue cobimetinib and vemurafenib.<br>• For recurrent events, treat as a Grade 3 or 4 event.
Pulmonary event, Grade 3 or 4	• Permanently discontinue atezolizumab and contact Medical Monitor. [c] Withhold cobimetinib and vemurafenib.<br>• Bronchoscopy or BAL is recommended.<br>• Initiate treatment with 1−2 mg/kg/day oral prednisone or equivalent.<br>• If event does not improve within 48 hours after initiating corticosteroids, consider adding an immunosuppressive agent.<br>• If event resolves to Grade 1 or better, taper corticosteroids over □ 1 month.<br>• If event resolves to Grade 1 or better within 28 days, cobimetinib and vemurafenib may be resumed with doses reduced by one level. If not, permanently discontinue cobimetinib and vemurafenib.

BAL = bronchoscopic alveolar lavage.

[a] If corticosteroids have been initiated, they must be tapered over □1 month to □10 mg/day oral prednisone or equivalent before atezolizumab can be resumed.

[b] atezolizumab may be withheld for a period of time beyond 12 weeks to allow for corticosteroids to be reduced to □10 mg/day oral prednisone or equivalent. The acceptable length of the extended period of time must be agreed upon by the investigator and the Medical Monitor.

[c] Resumption of atezolizumab may be considered in patients who are deriving benefit and have fully recovered from the immune-related event. Patients can be rechallenged with atezolizumab only after approval has been documented by both the investigator (or an appropriate delegate) and the Medical Monitor.

Guidelines for Management of Patients Who Experience Specific Adverse Events (cont.)

Event	Action to Be Taken
**Endocrine disorders**	
Asymptomatic hypothyroidism	• Continue all study treatment.<br>• Initiate treatment with thyroid replacement hormone.<br>• Monitor TSH weekly.
Symptomatic hypothyroidism	• Withhold atezolizumab. Continue cobimetinib and vemurafenib.<br>• Initiate treatment with thyroid replacement hormone.<br>• Monitor TSH weekly.<br>• Consider patient referral to endocrinologist.<br>• When symptoms are controlled and thyroid function is improving, resume atezolizumab.
Asymptomatic hyperthyroidism	**TSH □ 0.1 mU/L and □ 0.5 mU/L:**<br>• Continue all study treatment.<br>• Monitor TSH every 4 weeks.<br>**TSH □ 0.1 mU/L:**<br>• Follow guidelines for symptomatic hyperthyroidism.
Symptomatic hyperthyroidism	• Withhold atezolizumab. Continue cobimetinib and vemurafenib.<br>• Initiate treatment with anti-thyroid drug such as methimazole or carbimazole as needed.<br>• Consider patient referral to endocrinologist.<br>• When symptoms are controlled and thyroid function is improving, resume atezolizumab.<br>• For lif e-threatening immune-related hyperthyroidism, permanently discontinue atezolizumab, withhold cobimetinib and vemurafenib, and contact Medical Monitor.[c] If event becomes clinically manageable within 28 days, resume cobimetinib and vemurafenib with doses reduced by one level. If not, permanently discontinue cobimetinib and vemurafenib.

TSH = thyroid-stimulating hormone.

[a] If corticosteroids have been initiated, they must be tapered over □1 month to □10 mg/day oral prednisone or equivalent before atezolizumab can be resumed.

[b] atezolizumab may be withheld for a period of time beyond 12 weeks to allow for corticosteroids to be reduced to □10 mg/day oral prednisone or equivalent. The acceptable length of the extended period of time must be agreed upon by the investigator and the Medical Monitor.

[c] Resumption of atezolizumab may be considered in patients who are deriving benefit and have fully recovered from the immune-related event. Patients can be rechallenged with atezolizumab only after approval has been documented by both the investigator (or an appropriate delegate) and the Medical Monitor.

Guidelines for Management of Patients Who Experience Specific Adverse Events (cont.)

Event	Action to Be Taken
**Endocrine disorders (cont.)**	
Adrenal insufficiency, Grade 2, 3, or 4	• Withhold atezolizumab. Continue cobimetinib and vemurafenib.<br>• Refer patient to endocrinologist.<br>• Perform appropriate imaging.<br>• Initiate treatment with 1−2 mg/kg/day IV methylprednisolone or equivalent and convert to 1−2 mg/kg/day oral prednisone or equivalent upon improvement.<br>• If event resolves to Grade 1 or better and patient is stable on replacement therapy within 12 weeks, resume atezolizumab at current dose.<br>• If event does not resolve to Grade 1 or better or patient is not stable on replacement therapy within 12 weeks, permanently discontinue atezolizumab and contact Medical Monitor. [a,b,c]
Hyperglycemia, Grade 1 or 2	• Continue all study treatment.<br>• Initiate treatment with insulin if needed. Consider cobimetinib dose modification per general guidelines as clinically indicated.<br>• Monitor for glucose control.
Hyperglycemia, Grade 3 or 4	• Withhold atezolizumab. Continue cobimetinib and vemurafenib.<br>• Initiate treatment with insulin.<br>• Monitor for glucose control.<br>• When symptoms resolve and glucose levels are stable, resume atezolizumab.

IV = intravenous.

[a] If corticosteroids have been initiated, they must be tapered over □1 month to □10 mg/day oral prednisone or equivalent before atezolizumab can be resumed.

[b] atezolizumab may be withheld for a period of time beyond 12 weeks to allow for corticosteroids to be reduced to □10 mg/day oral prednisone or equivalent. The acceptable length of the extended period of time must be agreed upon by the investigator and the Medical Monitor.

[c] Resumption of atezolizumab may be considered in patients who are deriving ben efit and have fully recovered from the immune-related event. Patients can be rechallenged with atezolizumab only after approval has been documented by both the investigator (or an appropriate delegate) and the Medical Monitor.

Event	Management
Hypophysitis (pan-hypopituitarism), Grade 2 or 3	• Withhold atezolizumab for up to 12 weeks after event onset. [a]<br>• Refer patient to endocrinologist.<br>• Perform brain MRI (pituitary protocol).<br>• Initiate treatment with corticosteroids equivalent to 1–2 mg/kg/day IV methylprednisolone and convert to 1–2 mg/kg/day oral prednisone or equivalent upon improvement.<br>• Initiate hormone replacement if clinically indicated.<br>• If event resolves to Grade 1 or better, resume atezolizumab. [b]<br>• If event does not resolve to Grade 1 or better while withholding atezolizumab, permanently discontinue atezolizumab. [c]<br>• For recurrent hypophysitis, treat as a Grade 4 event.
Hypophysitis (pan-hypopituitarism), Grade 4	• Permanently discontinue atezolizumab. [c]<br>• Refer patient to endocrinologist.<br>• Perform brain MRI (pituitary protocol).<br>• Initiate treatment with corticosteroids equivalent to 1–2 mg/kg/day IV methylprednisolone and convert to 1–2 mg/kg/day oral prednisone or equivalent upon improvement.<br>• Initiate hormone replacement if clinically indicated.

MRI = magnetic resonance imaging; TSH = thyroid-stimulating hormone.

[a] Atezolizumab may be withheld for a longer period of time (i.e., > 12 weeks after event onset) to allow for corticosteroids (if initiated) to be reduced to the equivalent of ≤ 10 mg/day oral prednisone. The acceptable length of the extended period of time must be determined by the investigator.

[b] If corticosteroids have been initiated, they must be tapered over ≥ 1 month to the equivalent of ≤ 10 mg/day oral prednisone before atezolizumab can be resumed.

[c] Resumption of atezolizumab may be considered in patients who are deriving benefit and have fully recovered from the immune-related event. Patients can be re-challenged with atezolizumab only after approval has been documented by the investigator (or an appropriate delegate).

Guidelines for Management of Patients Who Experience Specific Adverse Events (cont.)

Event	Action to Be Taken
**Pancreatic events**	
Amylase and/or lipase elevation, Grade 1	• Continue all study treatment.<br>• Monitor amylase and lipase prior to dosing.
Amylase and/or lipase elevation, Grade 2	• Continue all study treatment.<br>• Monitor amylase and lipase weekly.<br>• For prolonged elevation (e.g., □ 3 weeks), consider treatment with 10 mg/day oral prednisone or equivalent.
Amylase and/or lipase elevation, Grade 3 or 4	• Withhold atezolizumab and vemurafenib. Continue cobimetinib.<br>• Refer patient to GI specialist.<br>• Monitor amylase and lipase every other day.<br>• If no improvement, consider treatment with 1−2 mg/kg/day oral prednisone or equivalent.<br>• If event resolves to Grade 1 or better within 12 weeks, resume atezolizumab. If not, permanently discontinue atezolizumab and contact Medical Monitor. [a,b,c]<br>• If event resolves to Grade 1 or better within 28 days, resume vemurafenib with dose reduced by one level. If not, permanently discontinue vemurafenib.<br>• For recurrent events, permanently discontinue all study treatment and contact Medical Monitor. [c]

GI = gastrointestinal; IRR = infusion-related reaction; IV = intravenous.

[a] If corticosteroids have been initiated, they must be tapered over □1 month to □10 mg/day oral prednisone or equivalent before atezolizumab can be resumed.

[b] atezolizumab may be withheld for a period of time beyond 12 weeks to allow for corticosteroids to be reduced to □10 mg/day oral prednisone or equivalent. The acceptable length of the extended period of time must be agreed upon by the investigator and the Medical Monitor.

[c] Resumption of atezolizumab may be considered in patients who are deriving benefit and have fully recovered from the immune-related event. Patients can be rechallenged with atezolizumab only after approval has been documented by both the investigator (or an appropriate delegate) and the Medical Monitor.

Guidelines for Management of Patients Who Experience Specific Adverse Events (cont.)

Event	Action to Be Taken
**Pancreatic events (cont.)**	
Pancreatitis, Grade 3	• Withhold all study treatment.<br>• Refer patient to GI specialist.<br>• Initiate treatment with 1−2 mg/kg/day IV methylprednisolone or equivalent and convert to 1−2 mg/kg/day oral prednisone or equivalent upon improvement.<br>• If event resolves to Grade 1 or better within 12 weeks, resume atezolizumab. If not, permanently discontinue atezolizumab and contact Medical Monitor. [a,b,c]<br>• If event resolves to Grade 1 or better within 28 days, resume cobimetinib at current dose and resume vemurafenib with dose reduced by one level. If not, permanently discontinue cobimetinib and vemurafenib.<br>• For recurrent events, permanently discontinue all study treatment and contact Medical Monitor. [c]
Pancreatitis, Grade 4	• Permanently discontinue atezolizumab and contact Medical Monitor. Consider permanently discontinuing cobimetinib and vemurafenib.<br>• Refer patient to GI specialist.<br>• Initiate treatment with 1−2 mg/kg/day IV methylprednisolone or equivalent and convert to 1−2 mg/kg/day oral prednisone or equivalent upon improvement.<br>• If event does not improve within 48 hours after initiating corticosteroids, consider adding an immunosuppressive agent.<br>• If event resolves to Grade 1 or better, taper corticosteroids over □ 1 month.<br>• If cobimetinib and vemurafenib are withheld and event resolves to Grade 1 or better within 28 days, resume cobimetinib at current dose and resume vemurafenib with dose reduced by one level. If event does not resolve to Grade 1 or better within 28 days, permanently discontinue cobimetinib and vemurafenib.

GI = gastrointestinal; IRR = infusion-related reaction; IV = intravenous.

[a] If corticosteroids have been initiated, they must be tapered over □1 month to □10 mg/day oral prednisone or equivalent before atezolizumab can be resumed.

[b] atezolizumab may be withheld for a period of time beyond 12 weeks to allow for corticosteroids to be reduced to □10 mg/day oral prednisone or equivalent. The acceptable length of the extended period of time must be agreed upon by the investigator and the Medical Monitor.

[c] Resumption of atezolizumab may be considered in patients who are deriving benefit and have fully recovered from the immune-related event. Patients can be rechallenged with atezolizumab only after approval has been documented by both the investigator(or an appropriate delegate) and the Medical Monitor.

Guidelines for Management of Patients Who Experience Specific Adverse Events (cont.)

Event	Action to Be Taken
**Neurologic disorders**	
Neuropathy, Grade 1	• Continue all study treatment.<br>• Investigate etiology.
Neuropathy, Grade 2	• Withhold atezolizumab. Continue cobimetinib and vemurafenib.<br>• Investigate etiology.<br>• Initiate treatment as per institutional guidelines.<br>• If event resolves to Grade 1 or better within 12 weeks, resume atezolizumab. If not, permanently discontinue atezolizumab and contact Medical Monitor. [a,b,c]
Neuropathy, Grade 3	• Permanently discontinue atezolizumab and contact Medical Monitor.[c] Continue cobimetinib and vemurafenib.<br>• Initiate treatment as per institutional guidelines.
Neuropathy, Grade 4	• Permanently discontinue atezolizumab and contact Medical Monitor.[c] Withhold cobimetinib and vemurafenib.<br>• Initiate treatment as per institutional guidelines.<br>• If patient stabilizes within 28 days, consider resuming cobimetinib at current dose and vemurafenib with dose reduced by one level. If not, permanently discontinue cobimetinib and vemurafenib.
Myasthenia gravis or Guillain-Barre syndrome, Grades 1, 2, or 3	• Permanently discontinue atezolizumab and contact Medical Monitor.[c] Continue cobimetinib and vemurafenib.<br>• Refer patient to neurologist.<br>• Initiate treatment as per institutional guidelines.<br>• Consider treatment with 1−2 mg/kg/day oral or IV prednisone or equivalent.

IV = intravenous.

[a] If corticosteroids have been initiated, they must be tapered over □1 month to □10 mg/day oral prednisone or equivalent before atezolizumab can be resumed.

[b] atezolizumab may be withheld for a period of time beyond 12 weeks to allow for corticosteroids to be reduced to □10 mg/day oral prednisone or equivalent. The acceptable length of the extended period of time must be agreed upon by the investigator and the Medical Monitor.

[c] Resumption of atezolizumab may be considered in patients who are deriving benefit and have fully recovered from the immune-related event. Patients can be rechallenged with atezolizumab only after approval has been documented by both the investigator(or an appropriate delegate) and the Medical Monitor.

Guidelines for Management of Patients Who Experience Specific Adverse Events (cont.)

Event	Action to Be Taken
**Neurological disorders (cont.)**	
Myasthenia gravis or Guillain-Barré syndrome, Grade 4	• Permanently discontinue atezolizumab and contact Medical Monitor.[c] Withhold cobimetinib and vemurafenib.<br>• Refer patient to neurologist.<br>• Initiate treatment as per institutional guidelines.<br>• Consider treatment with 1−2 mg/kg/day oral or IV prednisone or equivalent.<br>• If patient stabilizes within 28 days, resume cobimetinib and vemurafenib at current doses. If not, permanently discontinue cobimetinib and vemurafenib.
**Immune-related meningoencephalitis, any grade**	• Permanently discontinue atezolizumab and contact Medical Monitor.[c] Withhold cobimetinib and vemurafenib.<br>• Refer patient to neurologist.<br>• Initiate treatment with 1−2 mg/kg/day IV methylprednisolone or equivalent and convert to 1−2 mg/kg/day oral prednisone or equivalent upon improvement.<br>• If event does not improve within 48 hours after initiating corticosteroids, consider adding an immunosuppressive agent.<br>• If event resolves to Grade 1 or better, taper corticosteroids over □ 1 month.<br>• If patient stabilizes within 28 days, resume cobimetinib and vemurafenib at current doses. If not, permanently discontinue cobimetinib and vemurafenib.
**Ocular toxicity**	
Potential immune-related ocular toxicity (e.g., uveitis, retinal events), Grade 1	• Continue all study treatment.<br>• Patient referral to ophthalmologist is strongly recommended.<br>• Initiate treatment with topical corticosteroid eye drops and topical immunosuppressive therapy.<br>• If symptoms persist, treat as a Grade 2 event.

[a] If corticosteroids have been initiated, they must be tapered over □1 month to □10 mg/day oral prednisone or equivalent before atezolizumab can be resumed.

[b] atezolizumab may be withheld for a period of time beyond 12 weeks to allow for corticosteroids to be reduced to □10 mg/day oral prednisone or equivalent. The acceptable length of the extended period of time must be agreed upon by the investigator and the Medical Monitor.

[c] Resumption of atezolizumab may be considered in patients who are deriving benefit and have fully recovered from the immune-related event. Patients can be rechallenged with atezolizumab only after approval has been documented by both the investigator (or an appropriate delegate) and the Medical Monitor.

Guidelines for Management of Patients Who Experience Specific Adverse Events (cont.)

Event	Action to Be Taken
**Ocular toxicity (cont.)**	
Potential immune-related ocular toxicity (e.g., uveitis, retinal events), Grade 2	• Withhold atezolizumab and vemurafenib. Continue cobimetinib.<br>• Patient referral to ophthalmologist is strongly recommended.<br>• Initiate treatment with topical corticosteroid eye drops and topical immunosuppressive therapy.<br>• If event resolves to Grade 1 or better within 12 weeks, resume atezolizumab. If not, permanently discontinue atezolizumab and contact Medical Monitor. [a,b,c]<br>• If event resolves to Grade 1 or better within 28 days, resume vemurafenib with dose reduced by one level. If not, permanently discontinue vemurafenib.
Potential immune-related ocular toxicity (e.g., uveitis, retinal events), Grade 3 or 4	• Permanently discontinue atezolizumab and contact Medical Monitor. [c] Withhold cobimetinib and vemurafenib.<br>• Refer patient to ophthalmologist.<br>• Initiate treatment with 1−2 mg/kg/day oral prednisone or equivalent.<br>• If event resolves to Grade 1 or better, taper corticosteroids over ≥ 1 month.<br>• If event resolves to Grade 1 or better within 28 days, resume cobimetinib at current dose and resume vemurafenib with dose reduced by one level. If not, permanently discontinue cobimetinib and vemurafenib.

[a] If corticosteroids have been initiated, they must be tapered over ≥1 month to ≤10 mg/day oral prednisone or equivalent before atezolizumab can be resumed.

[b] atezolizumab may be withheld for a period of time beyond 12 weeks to allow for corticosteroids to be reduced to ≤10 mg/day oral prednisone or equivalent. The acceptable length of the extended period of time must be agreed upon by the investigator and the Medical Monitor.

[c] Resumption of atezolizumab may be considered in patients who are deriving benefit and have fully recovered from the immune-related event. Patients can be rechallenged with atezolizumab only after approval has been documented by both the investigator (or an appropriate delegate) and the Medical Monitor.

Guidelines for Management of Patients Who Experience Specific Adverse Events (cont.)

Event	Action to Be Taken
**Ocular toxicity (cont.)**	
Other ocular toxicity (i.e., not immune related)	• Withhold cobimetinib and vemurafenib. Continue atezolizumab.<br>• Refer patient to ophthalmologist.<br>**Retinal vein occlusion:**<br>• Permanently discontinue cobimetinib and vemurafenib.<br>**Serous retinopathy, Grade 1:**<br>• Resume cobimetinib and vemurafenib at current doses.<br>• Continue ophthalmology follow-up.<br>**Serous retinopathy, Grade 2 or 3:**<br>• Resume vemurafenib at current dose.<br>• If event resolves to Grade 1 or better within 28 days, resume cobimetinib with dose reduced by one level. If not, permanently discontinue cobimetinib.<br>**Serous retinopathy, Grade 4:**<br>• Permanently discontinue cobimetinib. Withhold vemurafenib.<br>• If event resolves to Grade 1 or better within 28 days, reduce vemurafenib with dose reduced by one level. If not, permanently discontinue vemurafenib.<br>**All other ocular events, Grade 1:**<br>• Resume cobimetinib and vemurafenib at current doses.<br>**All other ocular events, Grade 2, 3, or 4:**<br>• If event resolves to Grade 1 or better within 28 days, resume cobimetinib and vemurafenib at current doses. If not, permanently discontinue cobimetinib and vemurafenib.

[a] If corticosteroids have been initiated, they must be tapered over □1 month to □10 mg/day oral prednisone or equivalent before atezolizumab can be resumed.

[b] atezolizumab may be withheld for a period of time beyond 12 weeks to allow for corticosteroids to be reduced to □10 mg/day oral prednisone or equivalent. The acceptable length of the extended period of time must be agreed upon by the investigator and the Medical Monitor.

[c] Resumption of atezolizumab may be considered in patients who are deriving ben efit and have fully recovered from the immune-related event. Patients can be rechallenged with atezolizumab only after approval has been documented by both the investigator (or an appropriate delegate) and the Medical Monitor.

Guidelines for Management of Patients Who Experience Specific Adverse Events (cont.)

Event	Action to Be Taken
**QT interval prolongation**	
QTcF □ 500 ms with change from baseline in QTcF of □ 60 ms	• Continue all study treatment.
QTcF □ 500 ms with change from baseline in QTcF of □ 60 ms<br>**<u>or</u>**<br>QTcF □ 500 ms with change from baseline in QTcF of □ 60 ms	• Withhold vemurafenib. Continue atezolizumab and cobimetinib.<br>• Rule out other risk factors for arrhythmia (e.g., myocardial ischemia). Check for electrolyte disturbances (e.g., potassium, magnesium, and calcium).<br>• Evaluate concomitant medications to determine if there is co-administration of drugs that prolong QT interval.<br>• Refer patient to cardiologist.<br>• Monitor ECG weekly.<br>• If QTcF improves to □ 500 ms or baseline within 28 days, resume vemurafenib with dose reduced by one level. Repeat ECG at 2 and 4 weeks after resuming vemurafenib, on Day 15 of each subsequent cycle for three cycles, and then every 3 months thereafter.<br>• If QTcF does not improve to □ 500 ms or baseline within 28 days, permanently discontinue vemurafenib.
QTcF □ 500 ms with change from baseline in QTcF of □ 60 ms	• Permanently discontinue vemurafenib. Continue atezolizumab and cobimetinib.<br>• Rule out other risk factors for arrhythmia (e.g., myocardial ischemia). Check for electrolyte disturbances (e.g., potassium, magnesium, and calcium).<br>• Evaluate concomitant medications to determine if there is co-administration of drugs that prolong QT interval.<br>• Refer patient to cardiologist.<br>• Monitor ECG weekly until QTcF improves to □ 500 ms or baseline.

QTcF = QT interval corrected using Fridericia's formula.

[a] If corticosteroids have been initiated, they must be tapered over □1 month to □10 mg/day oral prednisone or equivalent before atezolizumab can be resumed.

[b] atezolizumab may be withheld for a period of time beyond 12 weeks to allow for corticosteroids to be reduced to □10 mg/day oral prednisone or equivalent. The acceptable length of the extended period of time must be agreed upon by the investigator and the Medical Monitor.

[c] Resumption of atezolizumab may be considered in patients who are deriving benefit and have fully recovered from the immune-related event. Patients can be rechallenged with atezolizumab only after approval has been documented by both the investigator (or an appropriate delegate) and the Medical Monitor.

Guidelines for Management of Patients Who Experience Specific Adverse Events (cont.)

Event	Action to Be Taken
**Asymptomatic LVEF decrease from baseline**	
LVEF □ 50%<br>**<u>or</u>**<br>LVEF 40%−49% with □ 10% absolute decrease from baseline	• Continue all study treatment.
LVEF □ 40%<br>**<u>or</u>**<br>LVEF 40%−49% with □ 10% absolute decrease from baseline	• Withhold cobimetinib. Continue atezolizumab and vemurafenib.<br>• Re-evaluate LVEF at 14 days.<br>• If patient has a □ 10% absolute decrease from baseline in LVEF, resume cobimetinib with dose reduced by one level.<br>• If patient has an LVEF of □ 40% or a □ 10% absolute decrease from baseline, permanently discontinue cobimetinib.
**Symptomatic LVEF decrease from baseline**	• Withhold cobimetinib. Continue atezolizumab and vemurafenib.<br>• Patient referral to cardiologist is strongly recommended.<br>• Re-evaluate LVEF at 28 days.<br>**Patient is asymptomatic:**<br>• If patient has a □ 10% absolute decrease from baseline in LVEF, resume cobimetinib with dose reduced by one level.<br>• If patient has an LVEF of □ 40% or a □ 10% absolute decrease from baseline, permanently discontinue cobimetinib.<br>**Patient is symptomatic:**<br>• Permanently discontinue cobimetinib.

LVEF = left ventricular ejection fraction.

[a] If corticosteroids have been initiated, they must be tapered over □1 month to □10 mg/day oral prednisone or equivalent before atezolizumab can be resumed.

[b] atezolizumab may be withheld for a period of time beyond 12 weeks to allow for corticosteroids to be reduced to □10 mg/day oral prednisone or equivalent. The acceptable length of the extended period of time must be agreed upon by the investigator and the Medical Monitor.

[c] Resumption of atezolizumab may be considered in patients who are deriving benefit and have fully recovered from the immune-related event. Patients can be rechallenged with atezolizumab only after approval has been documented by both the investigator (or an appropriate delegate) and the Medical Monitor.

Guidelines for Management of Patients Who Experience Specific Adverse Events (cont.)

Event	Action to Be Taken
**Rhabdomyolysis or CPK elevation**	
General guidance	• Consider withholding cobimetinib while investigating etiology. Continue atezolizumab and vemurafenib.<br>• Assess patient for any history of strenuous physical activity, blunt trauma, or recent intramuscular injections.<br>• Evaluate for cardiac injury and rhabdomyolysis. If evidence of clinically significant cardiac injury or rhabdomyolysis, consider permanent discontinuation of cobimetinib.
Asymptomatic CPK elevation, Grade 1, 2, or 3	• Continue all study treatment.
Rhabdomyolysis or symptomatic CPK elevation, Grade 1, 2, or 3	• Withhold cobimetinib. Continue atezolizumab and vemurafenib.<br>• If event improves by at least one grade within 28 days, resume cobimetinib with dose reduced by one level. If not, permanently discontinue cobimetinib.
Rhabdomyolysis or CPK elevation (symptomatic or asymptomatic), Grade4	• Withhold cobimetinib. Continue atezolizumab and vemurafenib.<br>• If event improves to Grade □ 3 within 28 days, resume cobimetinib with dose reduced by one level. If not, permanently discontinue cobimetinib.
**Grade 3 or 4 or intolerable Grade 2 treatment-related toxicities not described above**	• Withhold all study treatment.<br>• If event resolves to Grade 1 or better within 12 weeks, resume atezolizumab at fixed dose. If not, permanently discontinue atezolizumab. [a,b,c]<br>• If event resolves to Grade 1 or better within 28 days, resume cobimetinib and vemurafenib with doses reduced by one level. If not, permanently discontinue cobimetinib and vemurafenib.

CPK = creatine phosphokinase.

[a] If corticosteroids have been initiated, they must be tapered over □1 month to □10 mg/day oral prednisone or equivalent before atezolizumab can be resumed.

[b] atezolizumab may be withheld for a period of time beyond 12 weeks to allow for corticosteroids to be reduced to □10 mg/day oral prednisone or equivalent. The acceptable length of the extended period of time must be agreed upon by the investigator and the Medical Monitor.

[c] Resumption of atezolizumab may be considered in patients who are deriving ben efit and have fully recovered from the immune-related event. Patients can be rechallenged with atezolizumab only after approval has been documented by both the investigator (or an appropriate delegate) and the Medical Monitor.

Guidelines for Management of Patients Who Experience Specific Adverse Events (cont.)

Event	Action to Be Taken
**Hemorrhage**	
Grade 3 events	• Interrupt cobimetinib treatment. There is no data on the effectiveness of cobimetinib dose modifications for hemorrhage events.<br>• Clinical judgment should be applied when considering restarting cobimetinib treatment.<br>• Vemurafenib dosing can be continued when cobimetinib treatment is interrupted, if clinically indicated.<br>• Continue atezolizumab treatment.
Grade 4 events or cerebral hemorrhage (all grades)	• Interrupt cobimetinib treatment.<br>• Permanently discontinue cobimetinib for hemorrhage events attributed to cobimetinib.<br>• Interrupt vemurafenib and restart if event improves within 28 days.<br>• Continue atezolizumab unless clinically indicated.

[a] If corticosteroids have been initiated, they must be tapered over □1 month to □10 mg/day oral prednisone or equivalent before atezolizumab can be resumed.

[b] atezolizumab may be withheld for a period of time beyond 12 weeks to allow for corticosteroids to be reduced to □10 mg/day oral prednisone or equivalent. The acceptable length of the extended period of time must be agreed upon by the investigator and the Medical Monitor.

[c] Resumption of atezolizumab may be considered in patients who are deriving benefit and have fully recovered from the immune-related event. Patients can be rechallenged with atezolizumab only after approval has been documented by both the investigator (or an appropriate delegate) and the Medical Monitor.

Appendix 10 Cohort 2 (Cobimetinib+atezolizumab) AE Management

Guidelines for Management of Patients Who Experience Adverse Events in Atezo + Cobi Arm

Event	Action to Be Taken
**IRRs, anaphylaxis, and hypersensitivity reaction**	• Guidelines for management of IRRs for atezolizumab are provided in Appendix 6.<br>• For anaphylaxis precautions, see Appendix 5.<br>• For severe hypersensitivity reactions, permanently discontinue atezolizumab and
**Gastrointestinal toxicity**	
General guidance	• All events of diarrhea or colitis should be thoroughly evaluated for more common effects.<br>• For events of significant duration or magnitude or associated with signs of system reactants (e.g., increased CRP, platelet count, or bandemia): Perform sigmoidos appropriate) with colonic biopsy, with three to five specimens for standard paraffin and lymphocytic infiltrates to confirm colitis diagnosis.<br>• Administer anti-diarrheal agents and other supportive care per institutional guideli care outlined below:<br><u>Medication</u><br>− Initiate loperamide (4 mg) every 6 hours around the clock, alternating with diph (5 mg)/atropine sulfate (0.5 mg) every 6 hours around the clock, until no loose<br>− If Grade □ 2 diarrhea persists after 48 hours of treatment with loperamide and d initiating second-line agents (e.g., octreotide, budesonide, tincture of opium).<br><u>Oral Supplementation</u><br>− Initiate potassium and/or magnesium if serum levels are less than the lower lim<br>− Consider rehydration therapy with oral electrolyte solution for Grade □ 1 diarrh_<br><u>Dietary Modifications</u><br>− Instruct patient to eat small meals and eliminate lactose-containing products fr<br>− Suggest diet of bananas, rice, apples, and toast, while avoiding fiber from vege<br>− Encourage adequate hydration with salt-containing liquids (e.g., broth, sports d

Atezo + Cobi = atezolizumab plus cobimetinib; CRP = C-reactive protein; IRR = infusion-related reaction.

Event	Action to Be Taken
**Gastrointestinal toxicity (cont.)**	
Diarrhea, Grade 1 or Grade 2 (tolerable)	• Continue atezolizumab and cobimetinib.<br>• Initiate supportive care and monitor patient closely.<br>• Investigate etiology, referring patient to GI specialist for evaluation of possible coli
Diarrhea, Grade 2 (intolerable) or Grade 3	• Withhold atezolizumab and cobimetinib.<br>• Initiate supportive care and monitor patient closely.<br>• Discontinue medications that may exacerbate colitis (e.g., NSAIDS) while investig<br>• Investigate etiology, referring patient to GI specialist for evaluation of possible coli appropriate.<br>• If event resolves to Grade 1 or better within 12 weeks after event onset, resume a permanently discontinue atezolizumab and cobimetinib and contact Medical Moni<br>• If event resolves to Grade 1 or better within 28 days, resume cobimetinib with dos permanently discontinue cobimetinib.
Diarrhea, Grade 4	• Permanently discontinue atezolizumab and cobimetinib and contact Medical Moni<br>• Initiate supportive care and monitor patient closely.<br>• Discontinue medications that may exacerbate colitis (e.g., NSAIDs) while investig<br>• Rule out bowel perforation.<br>• Investigate etiology, referring patient to GI specialist for evaluation of possible coli

Atezo + Cobi = atezolizumab plus cobimetinib; GI = gastrointestinal; NSAID = non-steroidal anti-inflammatory drug.

[a] **If corticosteroids have been initiated, they must be tapered over □1 month to □10 mg/day oral prednisone or equivalent befo**

[b] Atezolizumab may be withheld for a longer period of time (i.e., □ 12 weeks after event onset) to allow for corticosteroids to be prednisone or equivalent. The acceptable length of the extended period of time must be agreed upon by the investigator an

[c] Resumption of atezolizumab may be considered in patients who are deriving benefit and have fully recovered from the immun re-challenged with atezolizumab only after approval has been documented by both the investigator (or an appropriate del eg

Event	Action to Be Taken
**Gastrointestinal toxicity (cont.)**	
Colitis, Grade 1	• Continue atezolizumab and cobimetinib.<br>• Initiate supportive care and monitor patient closely.<br>• Discontinue medications that may exacerbate colitis (e.g., NSAIDs).<br>• Refer patient to GI specialist for evaluation and confirmatory biopsy if symptoms p
Colitis, Grade 2	• Withhold atezolizumab and cobimetinib.<br>• Initiate supportive care and monitor patient closely.<br>• Discontinue medications that may exacerbate colitis (e.g., NSAIDs).<br>• Refer patient to GI specialist for evaluation and confirmatory biopsy.<br>• For recurrent events or events that persist □ 5 days, initiate treatment with 1−2 mg equivalent.<br>• If event resolves to Grade 1 or better within 12 weeks after event onset, resume a permanently discontinue atezolizumab and cobimetinib and contact Medical Moni<br>• If event resolves to Grade 1 or better within 28 days, resume cobimetinib with dos permanently discontinue cobimetinib.

Atezo + Cobi = atezolizumab plus cobimetinib; GI = gastrointestinal; NSAID = non-steroidal anti-inflammatory drug.

[a] If corticosteroids have been initiated, they must be tapered over □ 1 month to □ 10 mg/day oral prednisone or equivalent befo
[b] Atezolizumab may be withheld for a longer period of time (i.e., □ 12 weeks after event onset) to allow for corticosteroids to be prednisone or equivalent. The acceptable length of the extended period of time must be agreed upon by the investigator and

[c] Resumption of atezolizumab may be considered in patients who are deriving benefit and have fully recovered from the immur re-challenged with atezolizumab only after approval has been documented by both the investigator (or an appropriate delega

5720

Event	Action to Be Taken
**Gastrointestinal toxicity (cont.)**	
Colitis, Grade 3	• Withhold atezolizumab and cobimetinib.<br>• Initiate supportive care and monitor patient closely.<br>• Discontinue medications that may exacerbate colitis (e.g., NSAIDs).<br>• Refer patient to GI specialist for evaluation and confirmatory biopsy.<br>• Initiate treatment with 1−2 mg/kg/day IV methylprednisolone or equivalent and co prednisone or equivalent upon improvement.<br>• If event resolves to Grade 1 or better within 12 weeks after event onset, resume a permanently discontinue atezolizumab and cobimetinib and contact Medical Moni<br>• If event resolves to Grade 1 or better within 28 days, resume cobimetinib with dos permanently discontinue cobimetinib.
Colitis, Grade 4	• Permanently discontinue atezolizumab and cobimetinib and contact Medical Moni<br>• Initiate supportive care and monitor patient closely.<br>• Discontinue medications that may exacerbate colitis (e.g., NSAIDs).<br>• Refer patient to GI specialist for evaluation and confirmatory biopsy.<br>• Initiate treatment with 1−2 mg/kg/day IV methylprednisolone or equivalent and cor prednisone or equivalent upon improvement.<br>• If event does not improve within 48 hours after initiating corticosteroids, consider agent.<br>• If event resolves to Grade 1 or better, taper corticosteroids over □ 1 month

Atezo + Cobi = atezolizumab plus cobimetinib; GI = gastrointestinal; NSAID = non-steroidal anti-inflammatory drug.

[a] If corticosteroids have been initiated, they must be tapered over □ 1 month to □ 10 mg/day oral prednisone or equivalent befo [b] Atezolizumab may be withheld for a longer period of time (i.e., □ 12 weeks after event onset) to allow for corticosteroids to be prednisone or equivalent. The acceptable length of the extended period of time must be agreed upon by the investigator and

[c] Resumption of atezolizumab may be considered in patients who are deriving benefit and have fully recovered from the immur re-challenged with atezolizumab only after approval has been documented by both the investigator (or an appropriate delega

Event	Action to Be Taken
**Dermatologic toxicity**	
General guidance	• A dermatologist should evaluate persistent and/or severe rash or pruritus. A bio contraindicated.
Dermatologic event, Grade 1 or 2	• Continue atezolizumab and cobimetinib.<br>• Initiate supportive care (e.g., antihistamines, topical corticosteroids). If event doe treatment with higher-potency topical corticosteroids.<br>• For Grade 2 rash, consider referral to dermatologist.<br>**Acneiform rash:**<br>• Consider initiating treatment with topical corticosteroids (e.g., hydrocortisone 2.5 antibiotics (minocycline, doxycycline, or antibiotics covering skin flora) as clinicall
Dermatologic event, Grade 3	• Withhold atezolizumab and cobimetinib.<br>• Refer patient to dermatologist. A biopsy should be performed if appropriate.<br>• Consider initiating treatment with 10 mg/day oral prednisone or equivalent, incre event does not improve within 48−72 hours.<br>• If event resolves to Grade 2 or better within 12 weeks after event onset, resume permanently discontinue atezolizumab and cobimetinib and contact Medical Mon<br>• If event resolves to Grade 2 or better within 28 days, resume cobimetinib with do permanently discontinue cobimetinib.<br>**Acneiform rash:**<br>• Consider initiating treatment with topical corticosteroids (e.g., hydrocortisone 2.5 antibiotics (minocycline, doxycycline, or antibiotics covering skin flora) when rest

Atezo + Cobi = atezolizumab plus cobimetinib.

[a] If corticosteroids have been initiated, they must be tapered over □ 1 month to □ 10 mg/day oral prednisone or equivalent befo [b]

Atezolizumab may be withheld for a longer period of time (i.e., □ 12 weeks after event onset) to allow for corticosteroids to be prednisone or equivalent. The acceptable length of the extended period of time must be agreed upon by the investigator and

[c] Resumption of atezolizumab may be considered in p atients who are deriving benefit and have fully recovered from the immu re-challenged with atezolizumab only after approval has been documented by both the investigator (or an appropriate delega

Event	Action to Be Taken
**Dermatologic toxicity (cont.)**	
Dermatologic event, Grade 4	• Permanently discontinue atezolizumab and cobimetinib and contact Medical Mon
**Elevations in ALT, AST, and/or bilirubin**	

AST/ALT □ULN to □3 □ULN with total bilirubin □2□ULN	• Continue atezolizumab and cobimetinib.
AST/ALT □3 □ULN to 5□ULN with total bilirubin □ULN to □ 2 □ ULN	• Continue atezolizumab and cobimetinib.<br>• Monitor LFTs at least weekly.<br>• Consider patient referral to a hepatologist and liver biopsy.<br>**Suspected immune-related events of □ 5 days' duration:**<br>• Consider withholding atezolizumab.<br>• Consider initiation of treatment with 1−2 mg/kg/day oral prednisone or equivalent<br>• If atezolizumab is withheld and event resolves to AST/ALT □ 3 □ ULN with total b after event onset, resume atezolizumab at fixed dose. If not, permanently discon cobimetinib and contact Medical Monitor. [a, b, c]

Atezo + Cobi = atezolizumab plus cobimetinib; LFT = liver function test; ULN = upper limit of normal.

[a] If corticosteroids have been initiated, they must be tapered over □ 1 month to □ 10 mg/day oral prednisone or equivalent befo [b] Atezolizumab may be withheld for a longer period of time (i.e., □ 12 weeks after event onset) to allow for corticosteroids to be prednisone or equivalent. The acceptable length of the extended period of time must be agreed upon by the investigator and

[c] Resumption of atezolizumab may be considered in patients who are deriving benefit and have fully recovered from the immur re-challenged with atezolizumab only after approval has been documented by both the investigator (or an appropriate delega

Event	Action to Be Taken
**Elevations in ALT, AST, and/or bilirubin (cont.)**	
AST/ALT □ 5 □ ULN to □ 10 □ ULN with total bilirubin □ULN to □2 □ULN	• Continue atezolizumab and cobimetinib.<br>• Monitor LFTs at least weekly.<br>• Consider patient referral to hepatologist and liver biopsy.<br>**Suspected immune-related events:**<br>• Withhold atezolizumab.<br>• Consider initiation of treatment with 1−2 mg/kg/day oral prednisone or equivalent<br>• If corticosteroids are initiated and event does not improve within 48 hours, consid agent.<br>• If event resolves to AST/ALT □ 3 □ ULN with total bilirubin □ 2 □ULN within 12 we atezolizumab at fixed dose. If not, permanently discontinue atezolizumab and co Monitor.[a, b, c]
AST/ALT □ULN to □3 □ULN with total bilirubin □2 □ULN	• Investigate causes for elevated bilirubin and initiate treatment as indicated per in<br>• Use best medical judgment when determining whether to continue study treatme

Atezo + Cobi = atezolizumab plus cobimetinib; LFT = liver function test; ULN = upper limit of normal.

[a] If corticosteroids have been initiated, they must be tapered over □ 1 month to □ 10 mg/day oral prednisone or equivalent befo

Atezolizumab may be withheld for a longer period of time (i.e., □ 12 weeks after event onset) to allow for corticosteroids to be prednisone or equivalent. The acceptable length of the extended period of time must be agreed upon by the investigator and

[c] Resumption of atezolizumab may be considered in patients who are deriving benefit and have ful ly recovered from the immu re-challenged with atezolizumab only after approval has been documented by both the investigator (or an appropriate delega

Event	Action to Be Taken
**Pulmonary events**	
General guidance	• All pulmonary events should be thoroughly evaluated for other commonly report pneumonia/infection, lymphangitic carcinomatosis, pulmonary embolism, heart f pulmonary disease, or pulmonary hypertension.
Pulmonary event, Grade 1	• Continue atezolizumab and cobimetinib.<br>• Re-evaluate on serial imaging.<br>• Consider patient referral to pulmonary specialist.
Pulmonary event, Grade 2	• Withhold atezolizumab and cobimetinib.<br>• Refer patient to pulmonary and infectious disease specialists and consider bron<br>• If bronchoscopy is consistent with immune-related etiology, initiate treatment wi or equivalent.<br>• If event resolves to Grade 1 or better within 12 weeks after event onset, resume not, permanently discontinue atezolizumab and cobimetinib and contact Medica<br>• If event resolves to Grade 1 or better within 28 days, resume cobimetinib at cur<br>• For recurrent events, treat as a Grade 3 or 4 event.

Atezo + Cobi = atezolizumab plus cobimetinib; BAL = bronchoscopic alveolar lavage.

[a] **If corticosteroids have been initiated, they must be tapered over □1 month to □10 mg/day oral prednisone or equivalent befo**

[b] Atezolizumab may be withheld for a longer period of time (i.e., □ 12 weeks after event onset) to allow for corticosteroids to be prednisone or equivalent. The acceptable length of the extended period of time must be agreed upon by the investigator an c

[c] Resumption of atezolizumab may be considered in p atients who are deriving benefit and have fully recovered from the immu re-challenged with atezolizumab only after approval has been documented by both the investigator (or an appropriate delega

Event	Action to Be Taken
**Pulmonary events (cont.)**	
Pulmonary event, Grade 3 or 4	• Permanently discontinue atezolizumab and cobimetinib and contact Medical Mo<br>• Refer patient to pulmonary and infectious disease specialists and consider bron<br>• If bronchoscopy is consistent with immune-related etiology, initiate treatment wi or equivalent.<br>• If pulmonary event does not improve within 48 hours or worsens, consider addi<br>• If event resolves to Grade 1 or better, taper corticosteroids over □ 1 month.
**Endocrine disorders**	
Asymptomatic hypothyroidism	• Follow guidelines for atezolizumab in Appendix 6.<br>• Continue cobimetinib.
Symptomatic hypothyroidism	• Follow guidelines for atezolizumab in Appendix 6.<br>• Continue cobimetinib.

Atezo + Cobi = atezolizumab plus cobimetinib; BAL = bronchoscopic alveolar lavage.

[a] **If corticosteroids have been initiated, they must be tapered over □1 month to □10 mg/day oral prednisone or equivalent befo**

[b] Atezolizumab may be withheld for a longer period of time (i.e., □ 12 weeks after event onset) to allow for corticosteroids to be prednisone or equivalent. The acceptable length of the extended period of time must be agreed upon by the investigator an

[c] Resumption of atezolizumab may be considered in p atients who are deriving benefit and have fully recovered from the immu re-challenged with atezolizumab only after approval has been documented by both the investigator (or an appropriate delega

Event	Action to Be Taken
**Endocrine disorders (cont.)**	
Asymptomatic hyperthyroidism	**TSH □ 0.1 mU/L and □ 0.5 mU/L:**<br>• Follow guidelines for atezolizumab in Appendix 6.<br>• Continue cobimetinib.<br>**TSH □ 0.1 mU/L:**<br>• Follow guidelines for symptomatic hyperthyroidism.
Symptomatic hyperthyroidism	• Follow guidelines for atezolizumab in Appendix 6.<br>• Continue cobimetinib.<br>• For life-threatening immune-related hyperthyroidism, withhold cobimetinib. If ev manageable within 28 days, resume cobimetinib with dose reduced by one leve cobimetinib.
Symptomatic adrenal insufficiency, Grade 2, 3, or 4	• Follow guidelines for atezolizumab in Appendix 6<br>• Continue cobimetinib.
Hyperglycemia, any grade	• Continue cobimetinib. Consider cobimetinib dose modification per general guid

Atezo + Cobi = atezolizumab plus cobimetinib; TSH = thyroid-stimulating hormone.

[a] If corticosteroids have been initiated, they must be tapered over □1 month to □10 mg/day oral prednisone or equivalent befo

[b] Atezolizumab may be withheld for a longer period of time (i.e., □ 12 weeks after event onset) to allow for corticosteroids to be prednisone or equivalent. The acceptable length of the extended period of time must be agreed upon by the investigator an

[c] Resumption of atezolizumab may be considered in patients who are deriving benefit and have fully recovered from the immun re-challenged with atezolizumab only after approval has been documented by both the investigator (or an appropriate del eg

Event	Action to Be Taken
**Pancreatic events**	
Amylase and/or lipase elevation, Grade 3 or 4	• Follow guidelines for atezolizumab in Appendix 6.<br>• Continue cobimetinib.<br>• For recurrent events, permanently discontinue cobimetinib.
Pancreatitis, Grade 2	• Follow guidelines for atezolizumab in Appendix 6.<br>• Continue cobimetinib.
Pancreatitis, Grade 3	• Follow guidelines for atezolizumab in Appendix 6.<br>• Withhold cobimetinib.<br>• If event resolves to Grade 1 or better within 28 days, resume cobimetinib at cur<br>discontinue cobimetinib.<br>• For recurrent events, permanently discontinue cobimetinib.
Pancreatitis, Grade 4	• Follow guidelines for atezolizumab in Appendix 6.<br>• Consider permanently discontinuing cobimetinib.<br>• If cobimetinib is withheld and event resolves to Grade 1 or better within 28 day<br>dose. If event does not resolve to Grade 1 or better within 28 days, permanent

Atezo + Cobi = atezolizumab plus cobimetinib.

[a] If corticosteroids have been initiated, they must be tapered over □1 month to □10 mg/day oral prednisone or equivalent befo

[b] Atezolizumab may be withheld for a longer period of time (i.e., □ 12 weeks after event onset) to allow for corticosteroids to b prednisone or equivalent. The acceptable length of the extended period of time must be agreed upon by the investigator an

[c] Resumption of atezolizumab may be considered in patients who are deriving benefit and have fully recovered from the immu re-challenged with atezolizumab only after approval has been documented by both the investigator (or an appropriate deleg

Event	Action to Be Taken
**Neurologic disorder**	
Neuropathy, Grade 1, 2, or 3	• Follow guidelines for atezolizumab in Appendix 6.<br>• Continue cobimetinib.
Neuropathy, Grade 4	• Follow guidelines for atezolizumab in Appendix 6.<br>• Withhold cobimetinib.<br>• If patient stabilizes within 28 days, consider resuming cobimetinib at current do discontinue cobimetinib.
Myasthenia gravis or Guillain-Barré syndrome, Grade 4	• Follow guidelines for atezolizumab in Appendix 6.<br>• Withhold cobimetinib.<br>• If patient stabilizes within 28 days, resume cobimetinib at current dose. If not, p cobimetinib.
**Immune-related meningoencephalitis, any grade**	• Follow guidelines for atezolizumab in Appendix 6.<br>• Withhold cobimetinib.<br>• If patient stabilizes within 28 days, resume cobimetinib at current dose. If not, p cobimetinib.

Atezo + Cobi = atezolizumab plus cobimetinib.

[a] **If corticosteroids have been initiated, they must be tapered over □1 month to □10 mg/day oral prednisone or equivalent befo**

[b] Atezolizumab may be withheld for a longer period of time (i.e., □ 12 weeks after event onset) to allow for corticosteroids to b prednisone or equivalent. The acceptable length of the extended period of time must be agreed upon by the investigator an

[c] Resumption of atezolizumab may be considered in patients who are deriving benefit and have fully recovered from the immu re-challenged with atezolizumab only after approval has been documented by both the investigator (or an appropriate del eg

Event	Action to Be Taken
**Ocular toxicity**	
General guidance	• An ophthalmologist should evaluate visual complaints. Ophthalmology evaluat visual acuity testing, intra-ocular pressure measurements, slit-lamp ophthalmos visual field, and optical coherence tomography. A fluorescein angiogram and/c should be considered, if clinically indicated.
Serous retinopathy, Grade 1 or Grade 2 (tolerable)	• Continue atezolizumab and cobimetinib.<br>• Continue ophthalmology follow-up as clinically indicated.
Serous retinopathy, Grade 2 (intolerable), Grade 3, or Grade 4	• Withhold cobimetinib. Continue atezolizumab as clinically indicated.<br>• Undergo complete ophthalmologic examination.<br>• If event improves by at least one grade within 28 days, resume cobimetinib wit not, permanently discontinue cobimetinib.
Retinal vein occlusion (any grade)	• Permanently discontinue cobimetinib. Continue atezolizumab.<br>• Initiate treatment as per institutional guidelines.

Atezo + Cobi = atezolizumab plus cobimetinib.

[a] If corticosteroids have been initiated, they must be tapered over □1 month to □10 mg/day oral prednisone or equivalent befo

[b] Atezolizumab may be withheld for a longer period of time (i.e., □ 12 weeks after event onset) to allow for corticosteroids to b prednisone or equivalent. The acceptable length of the extended period of time must be agreed upon by the investigator an

[c] Resumption of atezolizumab may be considered in patients who are deriving benefit and have fully recovered from the immu re-challenged with atezolizumab only after approval has been documented by both the investigator (or an appropriate deleg

Event	Action to Be Taken
**Ocular toxicity (cont.)**	
Potential immune-related ocular toxicity (e.g., uveitis, retinal events), Grade 1	• Continue atezolizumab and cobimetinib.<br>• Initiate treatment with topical corticosteroid eye drops and topical immunosupp<br>• If symptoms persist, treat as a Grade 2 event.
Potential immune-related ocular toxicity (e.g., uveitis, retinal events), Grade 2	• Withhold atezolizumab. Continue cobimetinib.<br>• Initiate treatment with topical corticosteroid eye drops and topical immunosupp<br>• If event resolves to Grade 1 or better within 12 weeks after event onset, resum permanently discontinue atezolizumab and cobimetinib and contact Medical M
Potential immune-related ocular toxicity (e.g., uveitis, retinal events), Grade 3 or 4	• Permanently discontinue atezolizumab and cobimetinib and contact Medical M

Atezo + Cobi = atezolizumab plus cobimetinib.

[a] If corticosteroids have been initiated, they must be tapered over □1 month to □10 mg/day oral prednisone or equivalent befo

[b] Atezolizumab may be withheld for a longer period of time (i.e., □ 12 weeks after event onset) to allow for corticosteroids to b prednisone or equivalent. The acceptable length of the extended period of time must be agreed upon by the investigator an

[c] Resumption of atezolizumab may be considered in patients who are deriving benefit and have fully recovered from the immu re-challenged with atezolizumab only after approval has been documented by both the investigator (or an appropriate deleg

Event	Action to Be Taken
**Asymptomatic LVEF decrease from baseline**	
LVEF □ 50% **<u>or</u>** LVEF 40%−49% with □ 10% absolute decrease from baseline	• Continue atezolizumab and cobimetinib.
LVEF □ 40% **<u>or</u>** LVEF 40%−49% with □ 10% absolute decrease from baseline	• Withhold cobimetinib. Continue atezolizumab as clinically indicated.<br>• Re-evaluate LVEF at 14 days.<br>• If patient has a □ 10% absolute decrease from baseline in LVEF, resume co one level. After resuming cobimetinib, re-evaluate LVEF at 2 weeks, 4 week then every 12 weeks (i.e., three cycles) thereafter, or as clinically indicated,<br>• If patient has an LVEF of □ 40% or a □ 10% absolute decrease from baseline cobimetinib. Continue LVEF assessments every 6 weeks, or as clinically in 50% or to the lower limit of normal.

Atezo + Cobi = atezolizumab plus cobimetinib; LVEF = left ventricular ejection fraction.

[a] If corticosteroids have been initiated, they must be tapered over □1 month to □10 mg/day oral prednisone or equivalent befo

[b] Atezolizumab may be withheld for a longer period of time (i.e., □ 12 weeks after event onset) to allow for corticosteroids to b prednisone or equivalent. The acceptable length of the extended period of time must be agreed upon by the investigator an

[c] Resumption of atezolizumab may be considered in patients who are deriving benefit and have fully recovered from the immu re-challenged with atezolizumab only after approval has been documented by both the investigator (or an appropriate deleg

Event	Action to Be Taken
**Ocular toxicity**	
General guidance	• An ophthalmologist should evaluate visual complaints. Ophthalmology evaluat visual acuity testing, intra-ocular pressure measurements, slit-lamp ophthalmos visual field, and optical coherence tomography. A fluorescein angiogram and/c should be considered, if clinically indicated.
Serous retinopathy, Grade 1 or Grade 2 (tolerable)	• Continue atezolizumab and cobimetinib.<br>• Continue ophthalmology follow-up as clinically indicated.
Serous retinopathy, Grade 2 (intolerable), Grade 3, or Grade 4	• Withhold cobimetinib. Continue atezolizumab as clinically indicated.<br>• Undergo complete ophthalmologic examination.<br>• If event improves by at least one grade within 28 days, resume cobimetinib wit not, permanently discontinue cobimetinib.
Retinal vein occlusion (any grade)	• Permanently discontinue cobimetinib. Continue atezolizumab.<br>• Initiate treatment as per institutional guidelines.

Atezo + Cobi = atezolizumab plus cobimetinib.

[a] If corticosteroids have been initiated, they must be tapered over □1 month to □10 mg/day oral prednisone or equivalent befo

[b] Atezolizumab may be withheld for a longer period of time (i.e., □ 12 weeks after event onset) to allow for corticosteroids to b prednisone or equivalent. The acceptable length of the extended period of time must be agreed upon by the investigator an

[c] Resumption of atezolizumab may be considered in patients who are deriving benefit and have fully recovered from the immu re-challenged with atezolizumab only after approval has been documented by both the investigator (or an appropriate deleg

5830

Event	Action to Be Taken
**Ocular toxicity (cont.)**	
Potential immune-related ocular toxicity (e.g., uveitis, retinal events), Grade 1	• Continue atezolizumab and cobimetinib.<br>• Initiate treatment with topical corticosteroid eye drops and topical immunosupp<br>• If symptoms persist, treat as a Grade 2 event.
Potential immune-related ocular toxicity (e.g., uveitis, retinal events), Grade 2	• Withhold atezolizumab. Continue cobimetinib.<br>• Initiate treatment with topical corticosteroid eye drops and topical immunosupp<br>• If event resolves to Grade 1 or better within 12 weeks after event onset, resum permanently discontinue atezolizumab and cobimetinib and contact Medical Mo
Potential immune-related ocular toxicity (e.g., uveitis, retinal events), Grade 3 or 4	• Permanently discontinue atezolizumab and cobimetinib and contact Medical M

Atezo + Cobi = atezolizumab plus cobimetinib.

[a] If corticosteroids have been initiated, they must be tapered over □1 month to □10 mg/day oral prednisone or equivalent befo

[b] Atezolizumab may be withheld for a longer period of time (i.e., □ 12 weeks after event onset) to allow for corticosteroids to b prednisone or equivalent. The acceptable length of the extended period of time must be agreed upon by the investigator an

[c] Resumption of atezolizumab may be considered in patients who are deriving benefit and have fully recovered from the immu re-challenged with atezolizumab only after approval has been documented by both the investigator (or an appropriate deleg

Event	Action to Be Taken
**Asymptomatic LVEF decrease from baseline**	
LVEF □ 50%<br>**<u>or</u>**<br>LVEF 40%−49% with □ 10% absolute decrease from baseline	• Continue atezolizumab and cobimetinib.
LVEF □ 40%<br>**<u>or</u>**<br>LVEF 40%−49% with □ 10% absolute decrease from baseline	• Withhold cobimetinib. Continue atezolizumab as clinically indicated.<br>• Re-evaluate LVEF at 14 days.<br>• If patient has a □ 10% absolute decrease from baseline in LVEF, resume co one level. After resuming cobimetinib, re-evaluate LVEF at 2 weeks, 4 week then every 12 weeks (i.e., three cycles) thereafter, or as clinically indicated,<br>• If patient has an LVEF of □ 40% or a □ 10% absolute decrease from baseline cobimetinib. Continue LVEF assessments every 6 weeks, or as clinically in 50% or to the lower limit of normal.

Atezo + Cobi = atezolizumab plus cobimetinib; LVEF = left ventricular ejection fraction.

[a] If corticosteroids have been initiated, they must be tapered over □1 month to □10 mg/day oral prednisone or equivalent befo

[b] Atezolizumab may be withheld for a longer period of time (i.e., □ 12 weeks after event onset) to allow for corticosteroids to b prednisone or equivalent. The acceptable length of the extended period of time must be agreed upon by the investigator an

[c] Resumption of atezolizumab may be considered in patients who are deriving benefit and have fully recovered from the immu re-challenged with atezolizumab only after approval has been documented by both the investigator (or an appropr iate deleg

5848

Event	Action to Be Taken
**Rhabdomyolysis or CPK elevation**	
General guidance	• Evaluate for cardiac cause (check ECG, serum cardiac troponin, and CPK-iso rhabdomyolysis (clinical examination; serum creatinine, potassium, calcium, p and urine myoglobin).<br>• Assess patient for any history of strenuous physical activity, blunt trauma, or r
Asymptomatic CPK elevation, Grade 1, 2, or 3	• Continue atezolizumab and cobimetinib.<br>• Recheck CPK at least once a week.
Asymptomatic CPK elevation, Grade 4	• Withhold atezolizumab and cobimetinib.<br>• If event resolves to Grade 3 or better within 28 days, resume cobimetinib with permanently discontinue cobimetinib.<br>• Resumption of atezolizumab may be considered in patients who are deriving b Medical Monitor.
Rhabdomyolysis or symptomatic CPK elevation	• Withhold atezolizumab and cobimetinib.<br>• If event improves by at least one grade and symptoms resolve within 28 days, reduced by one level. If not, permanently discontinue cobimetinib.<br>• Resumption of atezolizumab may be considered in patients who are deriving b Medical Monitor.

Atezo + Cobi = atezolizumab plus cobimetinib.

[a] If corticosteroids have been initiated, they must be tapered over □1 month to □10 mg/day oral prednisone or equivalent befo

[b] Atezolizumab may be withheld for a longer period of time (i.e., □ 12 weeks after event onset) to allow for corticosteroids to b prednisone or equivalent. The acceptable length of the extended period of time must be agreed upon by the investigator an

[c] Resumption of atezolizumab may be considered in patients who are deriving benefit and have fully recovered from the immu re-challenged with atezolizumab only after approval has been documented by both the investigator (or an appropriate del eg

5849

Event	Action to Be Taken
**Hemorrhage**	
Grade 3 hemorrhage other than cerebral hemorrhage	• Withhold cobimetinib. Continue atezolizumab.<br>• If event resolves to Grade 1 or better within 28 days, resume cobimetinib with<br>permanently discontinue cobimetinib.
Grade 4 hemorrhage or any grade cerebral hemorrhage	• Permanently discontinue cobimetinib if event is attributed to cobimetinib; other<br>Continue atezolizumab.<br>• Clinical judgment should be applied when considering whether cobimetinib sh<br>There are no data on the effectiveness of cobimetinib dose modifications for h
**Atezolizumab-related toxicity not described above**	
Grade 1 or 2	• Follow guidelines for atezolizumab in Appendix 6.<br>• Continue cobimetinib.
Grade 3 or 4	• Follow guidelines for atezolizumab in Appendix 6.<br>• Withhold cobimetinib.<br>• If event resolves to Grade 2 or better within 28 days, resume cobimetinib at cu<br>discontinue cobimetinib.
**Grade 3 or 4 or intolerable Grade 2 treatment-related toxicities not described above or in Appendix 6**	• Withhold atezolizumab and cobimetinib.<br>• If event resolves to Grade 1 or better within 12 weeks after event onset, resum<br>not, permanently discontinue atezolizumab and cobimetinib and contact Medi<br>• If event resolves to Grade 1 or better within 28 days, resume cobimetinib with<br>permanently discontinue cobimetinib.

Atezo + Cobi = atezolizumab plus cobimetinib.

[a] If corticosteroids have been initiated, they must be tapered over □1 month to □10 mg/day oral prednisone or equivalent befo

[b] Atezolizumab may be withheld for a longer period of time (i.e., □ 12 weeks after event onset) to allow for corticosteroids to b
prednisone or equivalent. The acceptable length of the extended period of time must be agreed upon by the investigator an

[c] Resumption of atezolizumab may be considered in patients who are deriving benefit and have fully recovered from the immu
re-challenged with atezolizumab only after approval has been documented by both the investigator (or an appropriate deleg

Appendix 11 Guidelines for Managing atezolizumab-related AEs

Gastrointestinal Events

Immune-related colitis has been associated with the administration of atezolizumab. Management guidelines for diarrhea or colitis are provided in the following table.

All events of diarrhea or colitis should be thoroughly evaluated for other more common etiologies. For events of significant duration or magnitude or associated with signs of systemic inflammation or acute-phase reactants (e.g., increased CRP, platelet count, or bandemia): Perform sigmoidoscopy (or colonoscopy, if appropriate) with colonic biopsy, with three to five specimens for standard paraffin block to check for inflammation and lymphocytic infiltrates to confirm colitis diagnosis.

Management Guidelines for Gastrointestinal Events (Diarrhea or Colitis)

Event	Management
Diarrhea or colitis, Grade 1	• Continue atezolizumab.<br>• Initiate symptomatic treatment.<br>• Endoscopy is recommended if symptoms persist for > 7 days.<br>• Monitor closely.
Diarrhea or colitis, Grade 2	• Withhold atezolizumab for up to 12 weeks after event onset.[a]<br>• Initiate symptomatic treatment.<br>• Patient referral to GI specialist is recommended.<br>• For recurrent events or events that persist > 5 days, initiate treatment with 1–2 mg/kg/day oral prednisone or equivalent.<br>• If event resolves to Grade 1 or better, resume atezolizumab.[b]<br>• If event does not resolve to Grade 1 or better while withholding atezolizumab, permanently discontinue atezolizumab and contact Medical Monitor.[c]
Diarrhea or colitis, Grade 3	• Withhold atezolizumab for up to 12 weeks after event onset.[a]<br>• Refer patient to gastrointestinal specialist for evaluation and confirmatory biopsy.<br>• Initiate treatment with 1–2 mg/kg/day IV methylprednisolone or equivalent and convert to 1–2 mg/kg/day oral prednisone or equivalent upon improvement.<br>• If event resolves to Grade 1 or better, resume atezolizumab.[b]<br>• If event does not resolve to Grade 1 or better while withholding atezolizumab, permanently discontinue atezolizumab and contact Medical Monitor.[c]
**Event**	**Management**
Diarrhea or colitis, Grade 4	• Permanently discontinue atezolizumab and contact Medical Monitor.[c]<br>• Refer patient to gastrointestinal specialist for evaluation and confirmation biopsy.<br>• Initiate treatment with 1–2 mg/kg/day IV methylprednisolone or equivalent and convert to 1–2 mg/kg/day oral prednisone or equivalent upon improvement.<br>• If event does not improve within 48 hours after initiating corticosteroids, consider adding an immunosuppressive agent.<br>• If event resolves to Grade 1 or better, taper corticosteroids over ≥1 month.

IV=intravenous

[a] Atezolizumab may be withheld for a longer period of time (i.e., > 12 weeks after event onset) to allow for corticosteroids (if initiated) to be reduced to ≤10 mg/day oral prednisone or equivalent. The acceptable length of the extended period of time must be agreed upon by the investigator and the Medical Monitor.

[b] If corticosteroids have been initiated, they must be tapered over ≥1 month to ≤10 mg/day oral prednisone or equivalent before atezolizumab can be resumed.

[c] Resumption of atezolizumab may be considered in patients who are deriving benefit and have fully recovered from the immune-related event. Patients can be rechallenged with atezolizumab only after approval has been documented by both the investigator (or an appropriate delegate) and the Medical Monitor.

Endocrine Events

Thyroid disorders or adrenal insufficiency has been associated with the administration of atezolizumab. Management guidelines for endocrine events are provided in the following table.

Patients with unexplained symptoms such as fatigue, myalgias, impotence, mental status changes, or constipation should be investigated for the presence of thyroid, pituitary, or adrenal endocrinopathies. The patient should be referred to an endocrinologist if an endocrinopathy is suspected. Thyroid-stimulating hormone (TSH) and free T3 and T4 levels should be measured to determine whether thyroid abnormalities are present. TSH, prolactin, and a morning cortisol level will help to differentiate primary adrenal insufficiency from primary pituitary insufficiency.

Management Guidelines for Endocrine Events

Event	Management
Asymptomatic hypothyroidism	• Continue atezolizumab.<br>• Initiate treatment with thyroid replacement hormone.<br>• Monitor TSH weekly.
Symptomatic hypothyroidism	• Withhold atezolizumab.<br>• Initiate treatment with thyroid replacement hormone.<br>• Monitor TSH weekly.<br>• Consider patient referral to endocrinologist.<br>• Resume atezolizumab when symptoms are controlled and thyroid function is improving.
Asymptomatic hyperthyroidism	TSH ≥ 0.1 mU/L and < 0.5 mU/L:<br>• Continue atezolizumab.<br>• Monitor TSH every 4 weeks.<br>TSH < 0.1 mU/L:<br>• Follow guidelines for symptomatic hyperthyroidism.
Symptomatic hyperthyroidism	• Withhold atezolizumab.<br>• Initiate treatment with anti-thyroid drug such as methimazole or carbimazole as needed.<br>• Consider patient referral to endocrinologist.<br>• Resume atezolizumab when symptoms are controlled and thyroid function is improving.<br>• Permanently discontinue atezolizumab and contact Medical Monitor for life-threatening immune-related hyperthyroidism.[c]

Event	Management
Symptomatic adrenal insufficiency, Grade 2–4	• Withhold atezolizumab for up to 12 weeks after event onset.[a]<br>• Refer patient to endocrinologist.<br>• Perform appropriate imaging.<br>• Initiate treatment with 1–2 mg/kg/day IV methylprednisolone or equivalent and convert to 1–2 mg/kg/day oral prednisone or equivalent upon improvement.<br>• If event resolves to Grade 1 or better and patient is stable on replacement therapy, resume atezolizumab.[b]<br>• If event does not resolve to Grade 1 or better or patient is not stable on replacement therapy while withholding atezolizumab, permanently discontinue atezolizumab and contact Medical Monitor.[c]
Hyperglycemia Grade 1 or 2	• Continue atezolizumab.<br>• Initiate treatment with insulin if needed.<br>• Monitor for glucose control.
Hyperglycemia, Grade 3 or 4	• Withhold atezolizumab.<br>• Initiate treatment with insulin.<br>• Monitor for glucose control.<br>• Resume atezolizumab when symptoms resolve and glucose levels are stable.
Hypophysitis pan-hypopituitarism), Grade 2-3	• Withhold atezolizumab for up to 12 weeks after event onset.[a]<br>• Refer patient to endocrinologist.<br>• Perform brain MRI (pituitary protocol).<br>• Initiate treatment with 1–2 mg/kg/day IV methylprednisolone or equivalent and convert to 1–2 mg/kg/day oral prednisone or equivalent upon improvement.[a]<br>• Initiate hormone replacement therapy if clinically indicated.<br>• If event resolves to Grade 1 or better, resume atezolizumab.[b]<br>• If event does not resolve to Grade 1 or better while withholding atezolizumab, permanently discontinue atezolizumab and contact Medical Monitor.[c]<br>• For recurrent hypophysitis, treat as a Grade 4 event.

Event	Management
Hypophysitis (pan-hypopituitarism), Grade 4	• Permanently discontinue atezolizumab and contact Medical Monitor.[c]<br>• Refer patient to endocrinologist.<br>• Perform brain MRI (pituitary protocol).<br>• Initiate treatment with 1–2 mg/kg/day IV methylprednisolone or equivalent and convert to 1–2 mg/kg/day oral prednisone or equivalent upon improvement.[a]<br>• Initiate hormone replacement therapy if clinically indicated.

MRI = magnetic resonance imaging; TSH = thyroid-stimulating hormone, IV = intravenous

[a] Atezolizumab may be withheld for a longer period of time (i.e., > 12 weeks after event onset) to allow for corticosteroids (if initiated) to be reduced to ≤ 10 mg/day oral prednisone or equivalent. The acceptable length of the extended period of time must be agreed upon by the investigator and the Medical Monitor.

If corticosteroids have been initiated, they must be tapered over ≥ 1 month to ≤ 10 mg/day oral prednisone or equivalent before atezolizumab can be resumed.

Resumption of atezolizumab may be considered in patients who are deriving benefit and have fully recovered from the immune-related event. Patients can be rechallenged with atezolizumab only after approval has been documented by both the investigator (or an appropriate delegate) and the Medical Monitor.

RENAL EVENTS

Immune-related nephritis has been associated with the administration of atezolizumab. Eligible patients must have adequate renal function. Renal function, including serum creatinine, should be monitored throughout study treatment. Patients with abnormal renal function should be evaluated and treated for other more common etiologies (including prerenal and postrenal causes, and concomitant medications such as non-steroidal anti-inflammatory drugs). Refer the patient to a renal specialist if clinically indicated. A renal biopsy may be required to enable a definitive diagnosis and appropriate treatment.

Patients with signs and symptoms of nephritis, in the absence of an identified alternate etiology, should be treated according to the guidelines below.

Table Management Guidelines for Renal Events

Event	Management
Renal event, Grade 1	• Continue atezolizumab.<br>• Monitor kidney function, including creatinine, closely until values resolve to within normal limits or to baseline values.
Renal event, Grade 2	• Withhold atezolizumab for up to 12 weeks after event onset. [a]<br>• Refer patient to renal specialist.<br>• Initiate treatment with corticosteroids equivalent to 1–2 mg/kg/day oral prednisone.<br>• If event resolves to Grade 1 or better, resume atezolizumab. [b]<br>• If event does not resolve to Grade 1 or better while withholding atezolizumab, permanently discontinue atezolizumab. [c]
Renal event, Grade 3 or 4	• Permanently discontinue atezolizumab.<br>• Refer patient to renal specialist and consider renal biopsy.<br>• Initiate treatment with corticosteroids equivalent to 1–2 mg/kg/day oral prednisone.<br>• If event does not improve within 48 hours after initiating corticosteroids, consider adding an immunosuppressive agent.<br>• If event resolves to Grade 1 or better, taper corticosteroids over ≥1 month.

[a] Atezolizumab may be withheld for a longer period of time (i.e., >12 weeks after event onset) to allow for corticosteroids (if initiated) to be reduced to the equivalent of ≤10 mg/day oral prednisone. The acceptable length of the extended period of time must be determined by the investigator.

[b] If corticosteroids have been initiated, they must be tapered over ≥1 month to the equivalent of ≤10 mg/day oral prednisone before atezolizumab can be resumed.

[c] Resumption of atezolizumab may be considered in patients who are deriving benefit and have fully recovered from the immune-related event. Patients can be re-challenged with atezolizumab only after approval has been documented by the investigator (or an appropriate delegate).

Ocular Events

An ophthalmologist should evaluate visual complaints (e.g., uveitis, retinal events). Management guidelines for ocular events are provided in the following table.

Management Guidelines for Ocular Events

Event	Management
Ocular event, Grade 1	• Continue atezolizumab.<br>• Patient referral to ophthalmologist is strongly recommended.<br>• Initiate treatment with topical corticosteroid eye drops and topical immunosuppressive therapy.<br>• If symptoms persist, treat as a Grade 2 event.
Ocular event, Grade 2	• Withhold atezolizumab for up to 12 weeks after event onset.[a]<br>• Patient referral to ophthalmologist is strongly recommended.<br>• Initiate treatment with topical corticosteroid eye drops and topical immunosuppressive therapy.<br>• If event resolves to Grade 1 or better, resume atezolizumab.[b]<br>• If event does not resolve to Grade 1 or better while withholding atezolizumab, permanently discontinue atezolizumab and contact Medical Monitor.[c]
Ocular event, Grade 3 or 4	• Permanently discontinue atezolizumab and contact Medical Monitor.[c]<br>• Refer patient to ophthalmologist.<br>• Initiate treatment with 1–2 mg/kg/day oral prednisone or equivalent.<br>• If event resolves to Grade 1 or better, taper corticosteroids over ≥1 month.

[a] Atezolizumab may be withheld for a longer period of time (i.e., > 12 weeks after event onset) to allow for corticosteroids (if initiated) to be reduced to ≤10 mg/day oral prednisone or equivalent. The acceptable length of the extended period of time must be agreed upon by the investigator and the Medical Monitor.

[b] If corticosteroids have been initiated, they must be tapered over ≥1 month to ≤10 mg/day oral prednisone or equivalent before atezolizumab can be resumed.

[c] Resumption of atezolizumab may be considered in patients who are deriving benefit and have fully recovered from the immune-related event. Patients can be rechallenged with atezolizumab only after approval has been documented by both the investigator (or an appropriate delegate) and the Medical Monitor.

5921

Immune-Related Myocarditis

Immune-related myocarditis has been associated with the administration of atezolizumab. Immune-related myocarditis should be suspected in any patient presenting with signs or symptoms suggestive of myocarditis, including, but not limited to, dyspnea, chest pain, palpitations, fatigue, decreased exercise tolerance, or syncope. Immune-related myocarditis needs to be distinguished from myocarditis resulting from infection (commonly viral, e.g. in a patient who reports a recent history of gastrointestinal illness), ischemic events, underlying arrhythmias, exacerbation of pre-existing cardiac conditions, or progression of malignancy. All patients with possible myocarditis should be urgently evaluated by performing cardiac enzyme assessment, an ECG, a chest X-ray, an echocardiogram, and a cardiac MRI as appropriate per institutional guidelines. A cardiologist should be consulted. An endomyocardial biopsy may be considered to enable a definitive diagnosis and appropriate treatment, if clinically indicated. Patients with signs and symptoms of myocarditis, in the absence of an identified alternate etiology, should be treated according to the guidelines below.

Table 48 Management Guidelines for Immune-Related Myocarditis

Event	Management
Immune-related myocarditis, Grade 1	• Refer patient to cardiologist<br>• Initiate treatment as per institutional guidelines.
Immune-related myocarditis, Grade 2	• Withhold atezolizumab for up to 12 weeks after event onset [a] and contact Medical Monitor.<br>• Refer patient to cardiologist<br>• Initiate treatment as per institutional guidelines and consider antiarrhythmic drugs, temporary pacemaker, ECMO, or VAD as appropriate.<br>• Consider treatment with 1–2 mg/kg/day IV methylprednisolone or equivalent and convert to 1–2 mg/kg/day oral prednisone or equivalent upon improvement.[a]<br>• If event resolves to Grade 1 or better, resume atezolizumab.[b]<br>• If event does not resolve to Grade 1 or better while withholding atezolizumab, permanently discontinue atezolizumab and contact Medical Monitor.[c]
Immune-related myocarditis, Grade 3-4	• Permanently discontinue atezolizumab and contact Medical Monitor.[c]<br>• Refer patient to cardiologist<br>• Initiate treatment as per institutional guidelines and consider antiarrhythmic drugs, temporary pacemaker, ECMO, or VAD as appropriate.<br>• Initiate treatment with 1–2 mg/kg/day IV methylprednisolone or equivalent and convert to 1–2 mg/kg/day oral prednisone or equivalent upon improvement.[a,b]<br>• If event does not improve within 48 hours after initiating corticosteroids, consider adding an immunosuppressive agent.<br>• If event resolves to Grade 1 or better, taper corticosteroids over ≥1 month.

ECMO = extracorporeal membrane oxygenation; VAD = ventricular assist device; IV = intravenous.

[a] Atezolizumab may be withheld for a longer period of time (i.e., >12 weeks after event onset) to allow for corticosteroids (if initiated) to be reduced to ≤10 mg/day oral prednisone or equivalent. The acceptable length of the extended period of time must be agreed upon by the investigator and the Medical Monitor.

[b] If corticosteroids have been initiated, they must be tapered over ≥1 month to ≤10 mg/day oral prednisone or equivalent before atezolizumab can be resumed.

[c] Resumption of atezolizumab may be considered in patients who are deriving benefit and have fully recovered from the immune-related event. Patients can be rechallenged with atezolizumab only after approval has been documented by both the investigator (or an appropriate delegate) and the Medical Monitor.

Infusion-Related Reactions and Cytokine-Release Syndrome

No premedication is indicated for the administration of Cycle 1 of atezolizumab. However, patients who experience an infusion-related reaction (IRR) or cytokine-release cyndrome (CRS) with atezolizumab may receive medication with antihistamines, antipyretics and/or analgesics (e.g., acetaminophen). Metamizole (dipyrone) is prohibited in treating atezolizumab-associated IRRs because of its potential for causing agranulocytosis.

IRRs are known to occur with the administration of monoclonal antibodies and have been reported with atezolizumab. These reactions, which are thought to be due to release of cytokines and/or other chemical mediators, occur within 24 hours of atezolizumab administration and are generally mild to moderate in

severity.

CRS is defined as a supraphysiologic response following administration of any immune therapy that results in activation or engagement of endogenous or infused T cells and/or other immune effector cells. Symptoms can be progressive, always include fever at the onset, and may include hypotension, capillary leak (hypoxia), and end-organ dysfunction [58]. CRS has been well documented with chimeric antigen receptor T-cell therapies and bispecific T-cell engager antibody therapies but has also been reported with immunotherapies that target PD-1 or PD-L1 [59, 60], including atezolizumab.

There may be significant overlap in signs and symptoms of IRRs and CRS, and in recognition of the challenges in clinically distinguishing between the two, consolidated guidelines for medical management of IRRs and CRS are provided in Table 7. For subsequent cycles, IRRs should be managed according to institutional guidelines.

Table 7 Management Guidelines for Infusion-Related Reactions and Cytokine-Release Syndrome

Event	Management
Grade 1[a]<br>Fever[b] with or without constitutional symptoms	● Immediately interrupt infusion.<br>● Upon symptom resolution, wait 30 minutes and then restart infusion at half the rate being given at the time of event onset.<br>● If the infusion is tolerated at the reduced rate for 30 minutes, the infusion rate may be increased to the original rate.<br>● If symptoms recur, discontinue infusion of this dose.<br>● Administer symptomatic treatment,[c] including maintenance of IV fluids for hydration.<br>● In case of rapid decline or prolonged CRS (> 2 days) or in patients with significant symptoms and/or comorbidities, consider managing as per Grade 2.<br>● For subsequent infusions, consider administration of oral premedication with antihistamines, anti-pyretics, and/or analgesics, and monitor closely for IRRs and/or CRS.
Grade 2[a]<br>Fever[b] with hypotension not requiring vasopressors **and/or** Hypoxia requiring low-flow oxygen[d] by nasal cannula or blow-by	● Immediately interrupt atezolizumab infusion.<br>● Upon symptom resolution, wait for 30 minutes and then restart infusion at half the rate being given at the time of event onset.<br>● If symptoms recur, discontinue infusion of this dose.<br>● Administer symptomatic treatment.[c]<br>● For hypotension, administer IV fluid bolus as needed.<br>● Monitor cardiopulmonary and other organ function closely (in the ICU, if appropriate). Administer IV fluids as clinically indicated and manage constitutional symptoms and organ toxicities as per institutional practice.<br>● Rule out other inflammatory conditions that can mimic CRS (e.g., sepsis). If no improvement within 24 hours, initiate workup and assess for signs and symptoms of HLH or MAS.<br>● Consider IV corticosteroids (e.g., methylprednisolone 2 mg/kg/day or dexamethasone 10 mg every 6 hours).<br>● Consider anti-cytokine therapy.[e]<br>● Consider hospitalization until complete resolution of symptoms. If no improvement within 24 hours, manage as per Grade 3, that is, hospitalize patient (monitoring in the ICU is recommended), permanently discontinue atezolizumab.<br>● If symptoms resolve to Grade 1 or better for 3 consecutive days, the next dose of atezolizumab may be administered. For subsequent infusions, consider administration of oral premedication with antihistamines, anti-pyretics, and/or analgesics and monitor closely for IRRs and/or CRS.

	● If symptoms do not resolve to Grade 1 or better for 3 consecutive days, contact the sponsor-investigator.
Grade 3[a]<br>Fever[b] with hypotension requiring a vasopressor (with or without vasopressin)<br>**and/or**<br>Hypoxia requiring high-flow oxygen[d] by nasal cannula, face mask, non-rebreather mask, or venturi mask	● Permanently discontinue atezolizumab.[f]<br>● Administer symptomatic treatment.[c]<br>● For hypotension, administer IV fluid bolus and vasopressor as needed.<br>● Monitor cardiopulmonary and other organ function closely; monitoring in the ICU is recommended. Administer IV fluids as clinically indicated and manage constitutional symptoms and organ toxicities as per institutional practice.<br>● Rule out other inflammatory conditions that can mimic CRS (e.g., sepsis). If no improvement within 24 hours, initiate workup and assess for signs and symptoms of HLH or MAS.<br>● Administer IV corticosteroids (e.g., methylprednisolone 2 mg/kg/day or dexamethasone 10 mg every 6 hours).<br>● Consider anti-cytokine therapy.[e]<br>● Hospitalize patient until complete resolution of symptoms. If no improvement within 24 hours, manage as per Grade 4, that is, admit patient to ICU and initiate hemodynamic monitoring, mechanical ventilation, and/or IV fluids and vasopressors as needed; for patients who are refractory to anti-cytokine therapy, experimental treatments may be considered at the discretion of the investigator.
Grade 4[a]<br>Fever[b] with hypotension requiring multiple vasopressors (excluding vasopressin) and/or<br>Hypoxia requiring oxygen by positive pressure (e.g., CPAP, BiPAP, intubation and mechanical ventilation)	● Permanently discontinue atezolizumab.[f]<br>● Administer symptomatic treatment.[c]<br>● Admit patient to ICU and initiate hemodynamic monitoring, mechanical ventilation, and/or IV fluids and vasopressors as needed. Monitor other organ function closely. Manage constitutional symptoms and organ toxicities as per institutional practice.<br>● Rule out other inflammatory conditions that can mimic CRS (e.g., sepsis). If no improvement within 24 hours, initiate workup and assess for signs and symptoms of HLH or MAS.<br>● Administer IV corticosteroids (e.g., methylprednisolone 2 mg/kg/day or dexamethasone 10 mg every 6 hours).<br>● Consider anti-cytokine therapy.[e] For patients who are refractory to anti-cytokine therapy, experimental treatments[g] may be considered at the discretion of the investigator.<br>● Hospitalize patient until complete resolution of symptoms.

ASTCT= American Society for Transplantation and Cellular Therapy; BiPAP= bi-level positive airway pressure; CAR= chimeric antigen receptor; CPAP= continuous positive airway pressure; CRS= cytokine-release syndrome; HLH= hemophagocytic lymphohistiocytosis; IRR = infusion-related reaction; MAS= macrophage activation syndrome.

Note: The management guidelines have been adapted from NCCN guidelines for management of CAR T-cell-related toxicities (Version 2.2019).

a. Grading system for management guidelines is based on ASTCT consensus grading for CRS. NCI CTCAE (version as specified in the protocol) should be used when reporting severity of IRRs, CRS, or organ toxicities associated with CRS on the Adverse Event eCRF. Organ toxicities associated with CRS should not influence overall CRS grading.
b. Fever is defined as temperature ≥ 38°C not attributable to any other cause. In patients who develop CRS and then receive anti-pyretic, anti-cytokine, or corticosteroid therapy, fever is no longer required when subsequently determining event severity (grade). In this case, the grade is driven by the presence of hypotension and/or hypoxia.

c. Symptomatic treatment may include oral or IV antihistamines, anti-pyretics, analgesics, bronchodilators, and/or oxygen. For bronchospasm, urticaria, or dyspnea, additional treatment may be administered as per institutional practice.
d. Low flow is defined as oxygen delivered at ≤ 6 L/min, and high flow is defined as oxygen delivered at > 6 L/min.
e. There are case reports where anti-cytokine therapy has been used for treatment of CRS with immune checkpoint inhibitors (Rotz et al. 2017; Adashek and Feldman 2019), but data are limited, and the role of such treatment in the setting of antibody-associated CRS has not been established.
f. Resumption of atezolizumab may be considered in patients who are deriving benefit and have fully recovered from the immune-related event. Patients can be re-challenged with atezolizumab according to institutional guidelines and the above table. For subsequent infusions, administer oral premedication with antihistamines, anti-pyretics, and/or analgesics, and monitor closely for IRRs and/or CRS. Premedication with corticosteroids and extending the infusion time may also be considered after considering the benefit-risk ratio.
g. Refer to Riegler et al. [61] for information on experimental treatments for CRS.

Pancreatic Events

Symptoms of abdominal pain associated with elevations of amylase and lipase, suggestive of pancreatitis, have been associated with the administration of atezolizumab. The differential diagnosis of acute abdominal pain should include pancreatitis. Appropriate work-up should include an evaluation for ductal obstruction, as well as serum amylase and lipase tests. Management guidelines for pancreatic events, including pancreatitis, are provided in the following table.

Management Guidelines for Pancreatic Events, Including Pancreatitis

Event	Management
Amylase and/or lipase elevation, Grade 2	• Continue atezolizumab.<br>• Monitor amylase and lipase weekly.<br>• For prolonged elevation (e.g., > 3 weeks), consider treatment with 10 mg/day oral prednisone or equivalent.
Amylase and/or lipase elevation, Grade 3 or 4	• Withhold atezolizumab for up to 12 weeks after event onset.[a]<br>• Refer patient to gastrointestinal specialist.<br>• Monitor amylase and lipase every other day.<br>• If no improvement, consider treatment with 1−2 mg/kg/day oral prednisone or equivalent.<br>• If event resolves to Grade 1 or better, resume atezolizumab.[b]<br>• If event does not resolve to Grade 1 or better while withholding atezolizumab, permanently discontinue atezolizumab and contact Medical Monitor.[c]<br>• For recurrent events, permanently discontinue atezolizumab and contact Medical Monitor. c
Immune-related pancreatitis, Grade 2 or 3	• Withhold atezolizumab for up to 12 weeks after event onset.[a]<br>• Refer patient to gastrointestinal specialist.<br>• Initiate treatment with 1−2 mg/kg/day IV methylprednisolone or equivalent and convert to 1−2 mg/kg/day oral prednisone or equivalent upon improvement.<br>• If event resolves to Grade 1 or better, resume atezolizumab.<br>• If event does not resolve to Grade 1 or better while withholding atezolizumab, permanently discontinue atezolizumab and contact Medical Monitor.[c]<br>• For recurrent events, permanently discontinue atezolizumab and contact Medical Monitor.[c]

5984

Event	Management
Immune-related pancreatitis, Grade 4	• Permanently discontinue atezolizumab and contact Medical Monitor.[c]<br>• Refer patient to gastrointestinal specialist.<br>• Initiate treatment with 1–2 mg/kg/day IV methylprednisolone or equivalent and convert to 1–2 mg/kg/day oral prednisone or equivalent upon improvement.<br>• If event does not improve within 48 hours after initiating corticosteroids, consider adding an immunosuppressive agent.<br>• If event resolves to Grade 1 or better, taper corticosteroids over ≥1 month.

IV = intravenous

[a] Atezolizumab may be withheld for a longer period of time (i.e., >12 weeks after event onset) to allow for corticosteroids (if initiated) to be reduced to ≤10 mg/day oral prednisone or equivalent. The acceptable length of the extended period of time must be agreed upon by the investigator and the Medical Monitor.

[b] If corticosteroids have been initiated, they must be tapered over ≥1 month to ≤10 mg/day oral prednisone or equivalent before atezolizumab can be resumed.

[c] Resumption of atezolizumab may be considered in patients who are deriving benefit and have fully recovered from the immune-related event. Patients can be rechallenged with atezolizumab only after approval has been documented by both the investigator (or an appropriate delegate) and the Medical Monitor.

Dermatologic Events

Treatment-emergent rash has been associated with atezolizumab. The majority of cases of rash were mild in severity and self limited, with or without pruritus. Although uncommon, cases of severe cutaneous adverse reactions such as Stevens-Johnson syndrome and toxic epidermal necrolysis have been reported with atezolizumab. A dermatologist should evaluate persistent and/or severe rash or pruritus. A biopsy should be considered unless contraindicated. Management guidelines for dermatologic events are provided in the following table.

Event	Management
Dermatologic event, Grade 1	• Continue atezolizumab.<br>• Consider treatment with topical corticosteroids and/or other symptomatic therapy (e.g., antihistamines).
Dermatologic event, Grade 2	• Continue atezolizumab.<br>• Consider patient referral to dermatologist for evaluation and, if indicated, biopsy.<br>• Initiate treatment with topical corticosteroids.<br>• Consider treatment with higher-potency topical corticosteroids if event does not improve.
Dermatologic event, Grade 3	• Withhold atezolizumab for up to 12 weeks after event onset. [a]<br>• Refer patient to dermatologist for evaluation and, if indicated, biopsy.<br>• Initiate treatment with corticosteroids equivalent to 10 mg/day oral prednisone, increasing dose to 1–2 mg/kg/day if event does not improve within 48–72 hours.<br>• If event resolves to Grade 1 or better, resume atezolizumab. [b]<br>• If event does not resolve to Grade 1 or better while withholding atezolizumab, permanently discontinue atezolizumab. [c]
Dermatologic event, Grade 4	• Permanently discontinue atezolizumab. [c]
Stevens-Johnson syndrome or toxic epidermal necrolysis (any grade)	**Additional guidance for Stevens-Johnson syndrome or toxic epidermal necrolysis:**<br>• Withhold atezolizumab for suspected Stevens-Johnson syndrome or toxic epidermal necrolysis.<br>• Confirm diagnosis by referring patient to a specialist (dermatologist, ophthalmologist, or urologist as relevant) for evaluation and, if indicated, biopsy.<br>• Follow the applicable treatment and management guidelines above.<br>• If Stevens-Johnson syndrome or toxic epidermal necrolysis is confirmed, permanently discontinue atezolizumab.

[a] Atezolizumab may be withheld for a longer period of time (i.e., $>$12 weeks after event onset) to allow for corticosteroids (if initiated) to be reduced to the equivalent of $\leq$10 mg/day oral prednisone. The acceptable length of the extended period of time must be determined by the investigator.

[b] If corticosteroids have been initiated, they must be tapered over $\geq$1 month to the equivalent of $\leq$10 mg/day oral prednisone before atezolizumab can be resumed.

[c] Resumption of atezolizumab may be considered in patients who are deriving benefit and have fully recovered from the immune-related event. Patients can be re-challenged with atezolizumab only after approval has been documented by the investigator (or an appropriate delegate).

Management Guidelines for Dermatologic Events

Neurologic Disorders

Myasthenia gravis and Guillain-Barré syndrome have been observed with single-agent atezolizumab. Patients may present with signs and symptoms of sensory and/or motor neuropathy. Diagnostic work-up is essential for an accurate characterization to differentiate between alternative etiologies. Management guidelines for neurologic disorders are provided in the following table.

Management Guidelines for Neurologic Disorders

Event	Management
Immune-related neuropathy, Grade 1	• Continue atezolizumab.<br>• Investigate etiology.
Immune-related neuropathy, Grade 2	• Withhold atezolizumab for up to 12 weeks after event onset.[a]<br>• Investigate etiology.<br>• Initiate treatment as per institutional guidelines.<br>• If event resolves to Grade 1 or better, resume atezolizumab.[b]<br>• If event does not resolve to Grade 1 or better while withholding atezolizumab, permanently discontinue atezolizumab and contact Medical Monitor.[c]
Immune-related neuropathy, Grade 3 or 4	• Permanently discontinue atezolizumab and contact Medical Monitor.[c]<br>• Initiate treatment as per institutional guidelines.
Myasthenia gravis and Guillain-Barré syndrome (any grade)	• Permanently discontinue atezolizumab and contact Medical Monitor.[c]<br>• Refer patient to neurologist.<br>• Initiate treatment as per institutional guidelines.<br>• Consider initiation of 1−2 mg/kg/day oral or IV prednisone or equivalent.

IV = intravenous

[a] Atezolizumab may be withheld for a longer period of time (i.e., > 12 weeks after event onset) to allow for corticosteroids (if initiated) to be reduced to ≤ 10 mg/day oral prednisone or equivalent. The acceptable length of the extended period of time must be agreed upon by the investigator and the Medical Monitor.

[b] If corticosteroids have been initiated, they must be tapered over ≥1 month to ≤ 10 mg/day oral prednisone or equivalent before atezolizumab can be resumed.

[c] Resumption of atezolizumab may be considered in patients who are deriving benefit and have fully recovered from the immune-related event. Patients can be rechallenged with atezolizumab only after approval has been documented by both the investigator (or an appropriate delegate) and the Medical Monitor.

Immune-Related Meningoencephalitis

Immune-related meningoencephalitis is an identified risk associated with the administration of atezolizumab. Immune-related meningoencephalitis should be suspected in any patient presenting with signs or symptoms suggestive of meningitis or encephalitis, including, but not limited to, headache, neck pain, confusion, seizure, motor or sensory dysfunction, and altered or depressed level of consciousness. Encephalopathy from metabolic or electrolyte imbalances needs to be distinguished from potential meningoencephalitis resulting from infection (bacterial, viral, or fungal) or progression of malignancy, or secondary to a paraneoplastic process.

All patients being considered for meningoencephalitis should be urgently evaluated with a CT scan and/or MRI scan of the brain to evaluate for metastasis, inflammation, or edema. If deemed safe by the treating physician, a lumbar puncture should be performed and a neurologist should be consulted.

Patients with signs and symptoms of meningoencephalitis, in the absence of an identified alternate etiology, should be treated according to the guidelines in the following table.

Management Guidelines for Immune-Related Meningoencephalitis

Event	Management
Immune-related meningoencephalitis, all grades	• Permanently discontinue atezolizumab and contact Medical Monitor. [a]<br>• Refer patient to neurologist.<br>• Initiate treatment with 1–2 mg/kg/day IV methylprednisolone or equivalent and convert to 1–2 mg/kg/day oral prednisone or equivalent upon improvement.<br>• If event does not improve within 48 hours after initiating corticosteroids, consider adding an immunosuppressive agent.<br>• If event resolves to Grade 1 or better, taper corticosteroids over ≥1 month.

IV = intravenous.

[a] Resumption of atezolizumab may be considered in patients who are deriving benefit and have fully recovered from the immune-related event. Patients can be rechallenged with atezolizumab only after approval has been documented by both the investigator (or an appropriate delegate) and the Medical Monitor.

Pulmonary Events

Dyspnea, cough, fatigue, hypoxia, pneumonitis, and pulmonary infiltrates have been associated with the administration of atezolizumab. Patients will be assessed for pulmonary signs and symptoms throughout the study and will also have CT scans of the chest performed at every tumor assessment.

All pulmonary events should be thoroughly evaluated for other commonly reported etiologies such as pneumonia or other infection, lymphangitic carcinomatosis, pulmonary embolism, heart failure, chronic obstructive pulmonary disease, or pulmonary hypertension. Management guidelines for pulmonary events are provided in the following table.

Management Guidelines for Pulmonary Events, Including Pneumonitis

Event	Management
Pulmonary event, Grade 1	• Continue atezolizumab and monitor closely.<br>• Re-evaluate on serial imaging.<br>• Consider patient referral to pulmonary specialist.
Pulmonary event, Grade 2	• Withhold atezolizumab for up to 12 weeks after event onset. [a]<br>• Refer patient to pulmonary and infectious disease specialists and consider bronchoscopy or BAL.<br>• Initiate treatment with 1–2 mg/kg/day oral prednisone or equivalent.<br>• If event resolves to Grade 1 or better, resume atezolizumab. [b]<br>• If event does not resolve to Grade 1 or better while withholding atezolizumab, permanently discontinue atezolizumab and contact Medical Monitor. [c]<br>• For recurrent events, treat as a Grade 3 or 4 event.
Pulmonary event, Grade 3 or 4	• Permanently discontinue atezolizumab and contact Medical Monitor. [c]<br>• Bronchoscopy or BAL is recommended.<br>• Initiate treatment with 1–2 mg/kg/day oral prednisone or equivalent.<br>• If event does not improve within 48 hours after initiating corticosteroids, consider adding an immunosuppressive agent.<br>• If event resolves to Grade 1 or better, taper corticosteroids over ≥ 1 month.

BAL = bronchoscopic alveolar lavage; IVIG = intravenous immunoglobulin

[a] Atezolizumab may be withheld for a longer period of time (i.e., > 12 weeks after event onset) to allow for corticosteroids (if initiated) to be reduced to ≤ 10 mg/day oral prednisone or equivalent. The acceptable length of the extended period of time must be agreed upon by the investigator and the Medical Monitor.

[b] If corticosteroids have been initiated, they must be tapered over ≥ 1 month to ≤ 10 mg/day oral prednisone or equivalent before atezolizumab can be resumed.

[c] Resumption of atezolizumab may be considered in patients who are deriving benefit and have fully recovered from the immune-related event. Patients can be re-challenged with atezolizumab only after approval has been documented by both the investigator (or an appropriate delegate) and the Medical Monitor.

Hepatic Events

Immune-related hepatitis has been associated with the administration of atezolizumab. Eligible patients must have adequate liver function, as manifested by measurements of total bilirubin and hepatic transaminases, and liver function will be monitored throughout study treatment. Management guidelines for hepatic events are provided in the following table.

Patients with right upper-quadrant abdominal pain and/or unexplained nausea or vomiting should have LFTs performed immediately and reviewed before administration of the next dose of study drug. For patients with elevated LFTs, concurrent medication, viral hepatitis, and toxic or neoplastic etiologies should be considered and addressed, as appropriate.

Management Guidelines for Hepatic Events

Event	Management
Hepatic event, Grade 1	• Continue atezolizumab.<br>• Monitor LFTs until values resolve to within normal limits.
Hepatic event, Grade 2	**All events:**<br>• Monitor LFTs more frequently until return to baseline values.<br>**Events of > 5 days' duration:**<br>• Withhold atezolizumab for up to 12 weeks after event onset. [a]<br>• Initiate treatment with 1–2 mg/kg/day oral prednisone or equivalent.<br>• If event resolves to Grade 1 or better, resume atezolizumab. [b]<br>• If event does not resolve to Grade 1 or better while withholding atezolizumab, permanently discontinue atezolizumab and contact Medical Monitor. [c]
Hepatic event, Grade 3 or 4	• Permanently discontinue atezolizumab and contact Medical Monitor. [c]<br>• Consider patient referral to gastrointestinal specialist for evaluation and liver biopsy to establish etiology of hepatic injury.<br>• Initiate treatment with 1–2 mg/kg/day oral prednisone or equivalent.<br>• If event does not improve within 48 hours after initiating corticosteroids, consider adding an immunosuppressive agent.<br>• If event resolves to Grade 1 or better, taper corticosteroids over ≥ 1 month.

LFTs = liver function tests.

[a] Atezolizumab may be withheld for a longer period of time (i.e., > 12 weeks after event onset) to allow for corticosteroids (if initiated) to be reduced to ≤ 10 mg/day oral prednisone or equivalent. The acceptable length of the extended period of time must be agreed upon by the investigator and the Medical Monitor.

[b] If corticosteroids have been initiated, they must be tapered over ≥ 1 month to ≤ 10 mg/day oral prednisone or equivalent before atezolizumab can be resumed.

[c] Resumption of atezolizumab may be considered in patients who are deriving benefit and have fully recovered from the immune-related event. Patients can be rechallenged with atezolizumab only after approval has been documented by both the investigator (or an appropriate delegate) and the Medical Monitor.

[d] appendix 9 and 10 give further information on managing hepatic events in the setting of cobimetinib plus or minus vemurafenib and supercede the instructions on this table

Management Guidelines for Immune-Related Myositis

Event	Management
Immune-related myositis, Grade 1	• Continue atezolizumab<br>• Refer patient to rheumatologist or neurologist.<br>• Initiate treatment as per institutional guidelines.
Immune-related myositis, Grade 2	• Withhold atezolizumab for up to 12 weeks after event onset [a] and contact Medical Monitor.<br>• Refer patient to rheumatologist or neurologist.<br>• Initiate treatment as per institutional guidelines.<br>• Consider treatment with corticosteroid equivalent to 1-2 mg/kg/day IV methylprednisolone and convert to 1-2 mg/kg/day oral prednisone or equivalent upon improvement.<br>• If corticosteroids are initiated and event does not improve within 48 hours after initiating corticosteroids, consider adding an immunosuppressive agent.<br>• If event resolves to Grade 1 or better, resume atezolizumab. [b]<br>• If event does not resolve to Grade 1 or better while withholding atezolizumab, permanently discontinue atezolizumab and contact Medical Monitor. [c]
Immune-related myositis, Grade 3	• Withhold atezolizumab for up to 12 weeks after event onset[a] and contact Medical Monitor.<br>• Refer patient to rheumatologist or neurologist.<br>• Initiate treatment as per institutional guidelines.<br>• Respiratory support may be required in more severe cases.<br>• Initiate treatment with corticosteroids equivalent to 1-2 mg/kg/day IV methylprednisolone or higher-dose bolus if patient is severely compromised (e.g. cardiac or respiratory symptoms, dysphagia, or weakness that severely limits mobility); convert to 1-2 mg/kg/day oral prednisolone or equivalent upon improvement.<br>• If event does not improve within 48 hours after initiating corticosteroids, consider adding an immunosuppressive agent.<br>• If event resolves to Grade 1 or better, resume atezolizumab[b].<br>• If event does not resolve to Grade 1 or better while withholding atezolizumab, permanently discontinue atezolizumab and contact Medical Monitor [c].<br>• For recurrent events, treat as a Grade 4 event.

Event	Management
Immune-related myositis, Grade 4	• Permanently discontinue atezolizumab and contact Medical Monitor.[c]<br>• Refer patient to rheumatologist or neurologist.<br>• Initiate treatment as per institutional guidelines. Respiratory support may be required in more severe cases.<br>• Initiate treatment with corticosteroids equivalent to 1-2 mg/kg/day IV methylprednisolone or higher-dose bolus if patient is severely compromised (e.g. cardiac or respiratory symptoms, dysphagia, or weakness that severely limiting mobility); convert to 1-2 mg/kg/day oral prednisone or equivalent upon improvement.<br>• If event does not improve within 48 hours after initiating corticosteroids, consider adding an immunosuppressive agent.<br>• If event resolves to Grade 1 or better, taper corticosteroids over ≥ 1 month.

[a] Atezolizumab may be withheld for a longer period of time (i.e., > 12 weeks after event onset) to allow for corticosteroids (if initiated) to be reduced to ≤ 10 mg/day oral prednisone or equivalent. The acceptable length of the extended period of time must be agreed upon by the investigator and the Medical Monitor.

[b] If corticosteroids have been initiated, they must be tapered over ≥ 1 month to ≤ 10 mg/day oral prednisone or equivalent before atezolizumab can be resumed.

[c] Resumption of atezolizumab may be considered in patients who are deriving benefit and have fully recovered from the immune-related event. Patients can be re-challenged with atezolizumab only after approval has been documented by both the investigator (or an appropriate delegate) and the Medical Monitor.

HEMOPHAGOCYTIC LYMPHOHISTIOCYTOSIS AND MACROPHAGE ACTIVATION SYNDROME

Immune-mediated reactions may involve any organ system and may lead to hemophagocytic lymphohistiocytosis (HLH) and macrophage activation syndrome (MAS).

Patients with suspected HLH should be diagnosed according to published criteria by McClain and Eckstein (2014). A patient should be classified as having HLH if five of the following eight criteria are met:

Fever $\geq$38.5°C

Splenomegaly

Peripheral blood cytopenia consisting of at least two of the following:

- Hemoglobin $<$90 g/L (9 g/dL) ($<$100 g/L [10 g/dL] for infants $<$4 weeks old)
- Platelet count $< 100 \times 10^9$/L (100,000/μL)
- ANC $< 1.0 \times 10^9$/L (1000/μL)

Fasting triglycerides $>$2.992 mmol/L (265 mg/dL) and/or fibrinogen $<$1.5 g/L (150 mg/dL)

Hemophagocytosis in bone marrow, spleen, lymph node, or liver

Low or absent natural killer cell activity

Ferritin $>$500 mg/L (500 ng/mL)

Soluble interleukin 2 (IL-2) receptor (soluble CD25) elevated ≥ 2 standard deviations above age-adjusted laboratory-specific norms

Patients with suspected MAS should be diagnosed according to published criteria for systemic juvenile idiopathic arthritis by Ravelli et al. (2016). A febrile patient should be classified as having MAS if the following criteria are met:

Ferritin > 684 mg/L (684 ng/mL)

At least two of the following:

- Platelet count ≤ 181 × 10^9/L (181,000/μL)
- AST ≥ 48 U/L
- Triglycerides > 1.761 mmol/L (156 mg/dL)
- Fibrinogen ≤ 3.6 g/L (360 mg/dL)

Patients with suspected HLH or MAS should be treated according to the guidelines in Table 1.

Table 1 Management Guidelines for Suspected Hemophagocytic Lymphohistiocytosis or Macrophage Activation Syndrome

Event	Management
Suspected HLH or MAS	• Permanently discontinue atezolizumab and contact Medical Monitor.<br>• Consider patient referral to hematologist.<br>• Initiate supportive care, including intensive care monitoring if indicated per institutional guidelines.<br>• Consider initiation of IV corticosteroids and/or an immunosuppressive agent.<br>• If event does not improve within 48 hours after initiating corticosteroids, consider adding an immunosuppressive agent.<br>• If event resolves to Grade 1 or better, taper corticosteroids over ≥ 1 month.

HLH = hemophagocytic lymphohistiocytosis; MAS = macrophage activation syndrome.

References

McClain KL, Eckstein O. Clinical features and diagnosis of hemophagocytic lymphohistiocytosis. Up to Date [resouce on the Internet]. 2014 [updated 29 October 2018; cited: 17 May 2019]. Available from: https://www.uptodate.com/contents/clinical-features-and-diagnosis-of-hemophagocytic-lymphohistiocytosis.

Ravelli A, Minoia F, Davi S, et al. 2016 classification criteria for macrophage activation syndrome complicating systemic juvenile idiopathisc arthritis: a European League Against Rheumatism/American College of Rheumatology/Paediatric Rheumatology International Trials Organisation Collaborative Initiative. Ann Rheum Dis 2016;75:481–9.

Appendix 12 Translational studies – atezolizumab combinations with chemotherapy for anaplastic and poorly differentiated thyroid carcinomas.

1. Samples Collection and Processing:

1.1. Core needle biopsy (CNB) or excisional biopsy: A fresh CNB or excisional biopsy (screening (optional), prior to cycle 2, and at progression) will be obtained with the purpose of research studies before and after treatment and sent to the MD Anderson Institutional Tissue Bank (ITB) immediately after collection. At least 5 tissue cores will be obtained from the CNB/surgical procedure. The number of cores obtained will be affected by the patient clinical condition at the time of biopsy and determined by the radiologist who is performing the procedure. It is important to note that in some patients, the biopsy sample will also be required for clinical diagnosis. In such case, the first specimen will be prioritized for clinical specimen processing. In most instances, a rapid on site evaluation (ROSE) is available locally to evaluate the adequacy of clinical sample, thus additional biopsies may be procured for this research project. Nevertheless, the amount tissue available for correlative studies can be variable. Core biopsy is typically performed using 21-18 gauge needle and with condition permitting, up to 5 cores should be collected.

These cores/surgical excision pieces will be processed for (Figure 1):

Cores or excision pieces 1 and 2: Immediate and overnight fixation in 10% buffered formalin for paraffin embedding, usually within 20-24 hour after fixation. For biopsies performed on Friday, fixation time may extend to 48 hours (FFPE samples). The FFPE sample is important as it also provides a histological confirmation for the presence and cellularity of tumor cells. FFPE samples will also be prioritized for immune gene expression profiling by NanoString or other to be determined assays (Figure 1)

Cores or excision pieces 3 to 5: Flash freezing in liquid nitrogen will be obtained for RNA (RNA-sequencing) and DNA (targeted or whole exome sequencing (WES), among other analysis. Potentially, flow cytometry from fresh tissue tumor tissues could be performed in selected cases.

The tissue and blood will be processed as follows:

A. FFPE Tissues: Immediate and overnight fixation in 10% buffered formalin for paraffin embedding, usually within 20-24 hour after fixation. For biopsies performed on Friday, fixation time may extend to 48 hours (FFPE samples). For pathology evaluation, at least one sample per 1-cm of diameter will be submitted for FFPE processing and pathology analysis. Hematoxylin and eosin (H&E)-stained sections from all FFPE diagnostic slides (tumor and adjacent normal level) will be scanned in Aperio image analysis for pathological evaluation and biomarker analysis

B. Fresh Tissues: Flash freezing in liquid nitrogen for genomic studies including RNA-sequencing and Whole Exome Sequencing (WES).

1.2. Blood Specimens: 64 ml of blood will be collected at several time points (screening, prior to cycle 2, and at progression) for: a) isolation of germ-line DNA from peripheral mononuclear blood cells (PMBCs); b) isolation of plasma for cell free DNA analysis of genomic abnormalities using gene panels; and, c) potential flow cytometry analyses for phenotypic and functional studies.

2. Biomarker Analysis:

2.1. Histology evaluation of tumor tissue: H&E-stained sections from CNBs and surgical excisions will be used to confirm the presence of tumor cells, as well as their abundance (tumor cellularity), stromal components and lymphocytic infiltrates. Hematoxylin and eosin (H&E)-stained sections from all FFPE diagnostic slides (tumor, normal and lymph nodes) will be scanned in Aperio™ digital pathology scanner analysis for pathological evaluation and selection of 1 or 2 blocks (depending on tumor availability) for biomarker analysis.

From the tumor tissue specimens, the following pathological analysis will be performed: 1) tumor diagnosis using the World Health Organization (WHO) classification; 2) lowest degree of tumor differentiation; 3) percentage of areas of necrosis; 4) percentage of areas of fibrosis; 5) percentage of viable tumor tissue; and, 6) percentage of viable malignant cells.

Central Review: The scanned H&E-stained slides will be available for pathology analysis at the TMP Pathology and Biomarker Lab (TMP-IL) chaired by Dr. Ignacio I. Wistuba, MD Anderson Cancer Center.

2.2. Quality Control (QC) of tumor tissue: All tissue specimens collected will be reviewed by reference pathologists. At least, three types of QC activities for specimens collected will be performed: a) histology/cytology examination of the tissues and cells; b) tissue quality assessment of fresh specimens for extraction of DNA, RNA and proteins, and to prepare histology specimens such as whole sections for immunohistochemistry and immunofluorescence; and, c) quality assessment of DNA, RNA and protein extracted. All histology stained samples will be scanned and digital images will be available for review.

2.3. Immunohistochemistry (IHC) and Immunofluorescence (IF) analyses: Fresh frozen and FFPE tissues will be used for analysis of immune markers. For immunohistochemistry (IHC) and multiplex immunofluorescence (IF) analyses, histology sections obtained from FFPE samples will be utilized (Figure 1). IHC and IF will be performed using autostainers. All antibodies used will be optimized for IHC/IF by examination of positive and negative controls and testing of the antibodies standard methods, including Western blotting. All pathology slides will be scanned into a digital image scanner and analyzed using image analysis software; IHC analysis will be performed using the Aperio Image Toolbox™ (Leica Biosystems) and IF analysis using, among pthers, the Vectra Inform™ (Perkin-Elmer) software. The following markers will be performed using optimized and validated protocols, as follows:

IHC assays: Staining of tumor tissue for PD-L1 will be conducted screening, prior to cycle 3 and at the time of initial radiographic progression and performed using the proper IHC assay. Briefly, 4µm-thick histology sections will be used for IHC will be performed on autostainers (Leica Bond Max, Leica Biosystems, Vista, CA). All antibodies have been optimized for IHC by examination of positive and negative controls and testing of the antibodies by Western blotting. To perform quantitative image analysis if the expression of each marker, all IHC slides will be scanned into a digital image scanner (Aperio™ AT Turbo, Leica Biosystems, Buffalo Grove, IL), and analyzed using the Aperio Image Genie Toolbox™ software (Leica Biosystems, Buffalo Grove, IL). Five random 1-mm square areas within the tumor region will be selected for analysis. The expression of IHC marker(s) in malignant cells will be evaluated using the Aperio™ digital H-score system which includes the percentage of positive cells (0 to 100) and intensity (0 to 3+), with a total score ranging from 0 to 300. The expression of markers in inflammatory cells will be examined using an infiltrate density score established by the number of cells expres sing a determined marker by tissue area.

Multiplex IF Analysis: Up to 20 immune markers distributed in 3-4 panels will be utilized. For multiplex IF analysis, we will use the Opal chemistry and multispectral microscopy Vectra system (Perkin-Elmer) which includes the Nuance software; analysis will be performed using the InForm software. The expression of protein markers and inflammatory cells will be examined using an infiltrate density score established by the number of cells expressing a determined marker by tissue area. The data and digital images will be deposited in a central database for review by pathologists. Among other markers, we will study the expression of the following CD3, CD4, CD8, PD-L1, PD-1, FOXP3, CD45RO, CD57, CD68, and Granzyme B; additional markers will be selected according the results of the gene expression analysis and may include other immunotherapy targets (e.g., OX-40, Vista, GITR, TIM-3, LAG-3, NKp46/CD16, etc.) and proliferation markers (e.g., Ki67).

2.4. Nucleic acids and protein extraction: Blood (plasma and PMBCs), tumor (CNB and surgical excision specimens) samples will be subjected to DNA, RNA and protein extraction using standard methods. DNA and RNA quantity and integrity will be assessed using NanoDrop 1000 spectrophotometer (Nanodrop technologies) and Pico-green analyses. Also, protein lysate will be extracted using standard methods.

2.5. Molecular analysis of tumor tissues: Using FFPE and/or fresh frozen for CNBs/surgical excisions (Figure 1), the following analysis will be performed:

Immuno-oncology (IO) gene expression signatures: Using FFPE tumor tissues, IO panels of genes will be examined using the Nanostring technolog y (nCounter). This assay will be used to measure expression levels of drug targets, tumor infiltrate composition, and total immune cell composition using a single section of FFPE tumor tissue. The Nanostring methodology offers a cost-effective way to analyze the expression levels of up to 800 genes simultaneously, with precision superior to qPCR. The current Nanostring PanCancer Immune Profiling Panel includes 770 genes and combines markers for 24 different immune cell types and populations, 30 common cancer antigens and genes that represent all categories of immune responses including

key checkpoint blockade genes. Alternatively, we will apply the HTG Edge Seq technology, also known as quantitative nuclease protection assay or HTG Edge Chemistry that enables extraction and amplification-free quantitation of mRNA from FFPE tissues without RNA extraction. Their Immuno-Oncology Assay examines the expression of 549 genes implicated in the host immune response to tumors.

Next Generation Sequencing (NGS) analysis: To study tumor molecular abnormalities, fresh, and alternatively, FFPE tumor tissues before and after treatment will be examined for targeted gene panel NGS (analysis of mutations, copy number, indels, translocations), whole exome sequencing (WES) and RNA sequencing. Targeted NGS: Different sequencing platforms can be used to sequence DNA extracted from clinical samples. These platforms have a minimum input of 10ng of sample which make it amenable to sequencing with minimal DNA. The panels available are, among others, CMS50, CMS400, Oncomine and Foundation Medicine. They range from 50 to 409 oncogenes and tumor suppressor genes, with coverage of hotspots and whole exomes. Because targeted NGS is performed as standard of care on all ATC patients, this will only be repeated on the screening biopsy in select patients (ie, those treated with systemic therapy prior to the screening biopsy). All these platforms are available at facilities at MD Anderson Cancer Center. WES and RNA-seq: Illumina Hi-seq platform is available at the Sequencing Facilities at MD Anderson Cancer Center

2.6. Flow cytometry Analysis: This type of analysis may be applied to two types of specimens.

Cryopreserved PBMCs**:** High order flow cytometry panels are available f or analysis of tumor tissue and blood specimens. The panels will focus on 1) delineation of major immune cell types (T cells, B cells, NK cells, DC), 2) determination of T cell differentiation status and limited functionality (IFN□, TNF□, GB) and 3) defining the expression level of costimulatory and co-inhibitory molecules on T cells. The proposed studies may be conducted retrospectively on cryopreserved PBMCs. Briefly, 40 cc (at screening, prior to cycle 3 and at the time of initial radiographic progression) of heparinized peripheral blood from patients will be processed fresh (within 24h of being drawn) for PBMC isolation. PBMCs will be cryopreserved and stored in liquid nitrogen until use.

Flow cytometry of freshly disaggregated tumor tissue. In selected cases, fresh tissue may be available for flow cytometry analysis. Tumor tissue will be stored in HBSS for up to 24h before processing for flow cytometry. Fresh tissue will be mechanically disaggregated or digested according to needs and panel design. The cells will be processed as a single cell suspension and stained according to each customized panel.

2.7. Liquid biopsy analysis: Liquid biopsies are non-invasive blood tests that detect tumor cell free DNA (cfDNA) that are shed into the blood from the primary tumor and from metastatic sites. cfDNA testing offers the opportunity to take serial samples in order to monitor tumor genomic changes in real time. There are several platforms available at MD Anderson Cancer Center, including the application of droplet digital PCR (ddPCR) platform in a small panel of hot spots/genes or a larger panel of genes using NGS

platform. Additionally, isolation of circulating tumor cells (CTCs) and exosomes for genotyping DNA purposes are available as optional analysis of blood compartments.

Figure 1 Tissue

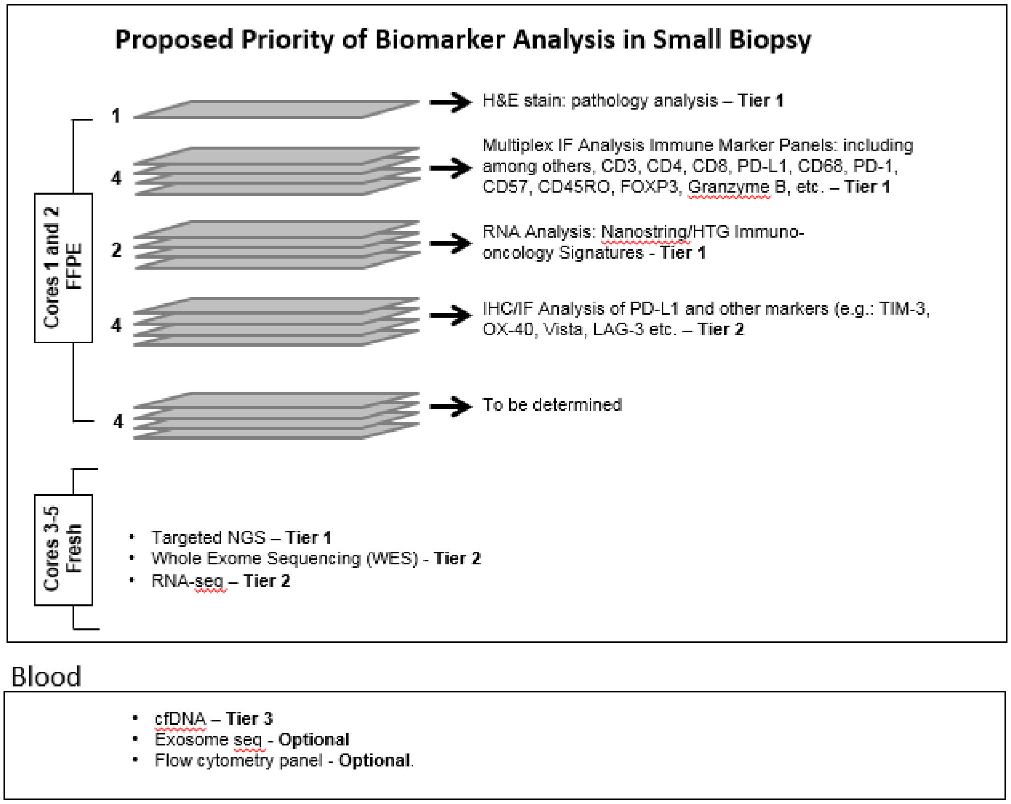

Appendix 13 Patient Instructions for Oral Vemurafenib

Vemurafenib may be crushed if you are unable to swallow the pills whole, however, *vemurafenib must never be diluted in water*. Please follow the instructions below if you are unable to swallow vemurafenib pills whole.

Pick the instructions appropriate to your situation. For example, if you are able to swallow, choose instruction “A”. If you must put your medications in the PEG tube please choose instruction “B”:

You will need the following materials before you start:
Disposable gloves
Face masks
A pill/tablet crusher (you can f ind this at any regular pharmacy)
3 ounces baby food fruit puree with <u>ascorbic acid</u> (vitamin C) or <u>lemon juice</u> in the ingredient list
Zipper bags (snack or sandwich sized)
Scissors
Additional materials for for instruction B:
A 60 cc syringe with cap
Container to hold water for flush
60 mL of water for flush

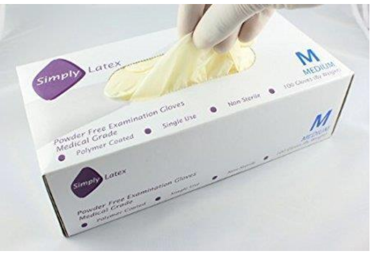

Disposable gloves

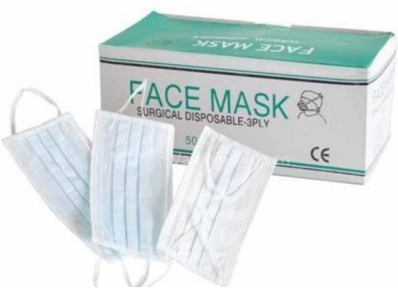

Face masks

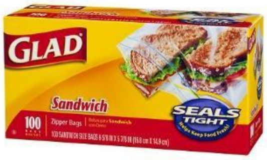

Zipper bags (sandwich or snack sized)

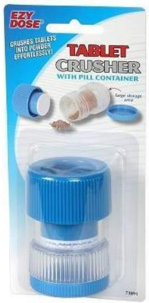

Pill crusher

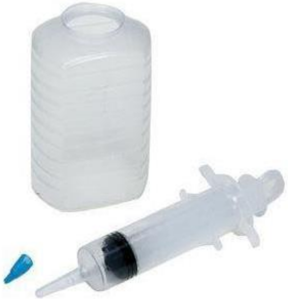
Syringe with cap and container for flush (for PEG tube administration only)

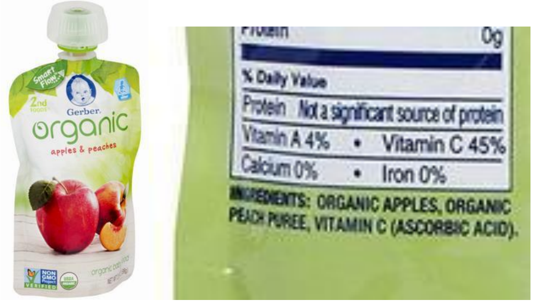

Baby food with ascorbic acid or lemon juice in the ingredient list

Instruction A: oral administration for patients who are able to swallow pureed foods

1. Always wear a face mask and gloves when handling chemotherapy
2. Place approximately 2-3 ounces of baby food in a zipper bag
3. Place vemurafenib tablets in a pill crusher and crush them into a fine powder. Do not crush more than 2 tablets at a time in order to obtain a fine powder.
4. Add the crushed vemurafenib to baby food in the zipper bag. Zip the bag and knead.
5. Cut a corner of the zipper bag and squeeze the entire contents of the baby food/vemurafenib mix into your mouth.

Instruction B: for patients administering vemurafenib by the PEG tube:

1. Always wear a face mask and gloves when handling chemotherapy
2. Place approximately 2-3 ounces of baby food in a zipper bag (about half the baby food)
3. Place vemurafenib tablets in a pill crusher and crush them into a fine powder. Do not crush more than 2 tablets at a time in order to obtain a fine powder.
4. Add the crushed vemurafenib to baby food in the zipper bag. Zip the bag and knead.
5. Cut a corner of the zipper bag.
6. Remove the plunger from a 60cc syringe (with cap on) squeeze the entire contents of the baby food/vemurafenib mix into it.
7. Replace the plunger, point the tip of syringe upward to allow for the baby food mix to move away from the tip. Remove the syringe cap and remove the air in the syringe.
8. Administer the baby food mixture in the PEG tube.

Rinse Steps:

1. After administration of the baby food mixture from the 60 cc syringe, close the tip port of the syringe with a cap and remove the plunger
2. Pour the remaining baby food into the syringe.
3. Replace the plunger, point the tip of syringe upward to allow for the baby food mix to move away from the tip. Remove the syringe cap and remove the air in the syringe.
4. Administer the baby food in the PEG tube
5. Flush the PEG tube with 60 mL or more of water.

Appendix 14 COVID-19 Pandemic: Standard Operating Procedures and Guidelines

Beginning March 1, 2020 and continuing throughout the duration of the pandemic, the following guidelines are implemented and permitted whenever necessary to ensure patient safety and continued treatment:

- Patients may be infused with a commercial supply of atezolizumab locally if unable to return to MD Anderson
- Patients may be held from atezolizumab while continuing oral investigational therapy, which will be shipped directly to the patient, in multiple cycles on a case-by-case basis
- Physical exams may be performed by a local physician
- Telehealth visits may occur by video and/or phone with an MD Anderson physician and/or an MD Anderson research study manager/research nurse
- Labs and imaging may continue to be performed off site
- EKGs and ECHOs may be perfomed off site
- Ophthalmology visits may be temporarily suspended unless clinically indicated in the opinion of the PI
- For physical exams, labs, and/or any other protocol procedures which do not occur on site or off site, deviations will be filed as appropriate
