# Supplementary material for: Anti–Programmed Death Ligand 1 Plus Targeted Therapy in Anaplastic Thyroid Carcinoma: A Nonrandomized Clinical Trial
Source: JAMA Oncol. 2024 Oct 24;10(12):1672–80. doi: 10.1001/jamaoncol.2024.4729 (PMC11581602; doi:10.1001/jamaoncol.2024.4729)
Supplement: Supplement 2. — eMethods 1. Entry criteria eMethods 2. Doses of study drugs eMethods 3. Patient instructions for alternative drug administration of vemurafenib eMethods 4. Futility analysis eFigure 1. Cohort 1 run-in and dosing eFigure 2. Treatment assignment eFigure 3. Oncoprint eFigure 4. Swimmer Plot eTable. Adverse Events amongst all cohorts [file jamaoncol-e244729-s002.pdf]

Supplemental Online Content

Cabanillas ME, Dadu R, Ferrarotto R, et al. Anti–programmed death ligand 1 plus targeted therapy in anaplastic thyroid carcinoma: a nonrandomized clinical trial. *JAMA Oncol*. Published online October 24, 2024. doi:10.1001/jamaoncol.2024.4729

eMethods 1. Entry criteria

eMethods 2. Doses of study drugs

eMethods 3. Patient instructions for alternative drug administration of vemurafenib

eMethods 4. Futility analysis

eFigure 1. Cohort 1 run-in and dosing

eFigure 2. Treatment assignment

eFigure 3. Oncoprint

eFigure 4. Swimmer Plot

eTable. Adverse Events amongst all cohorts

This supplemental material has been provided by the authors to give readers additional information about their work.

© 2024 Cabanillas ME, et al. *JAMA Oncol*.

eMethods 1. Entry criteria

<u>Inclusion Criteria</u>

1. Histologically confirmed anaplastic thyroid or poorly differentiated thyroid carcinomas.

2. Patients deemed to have unresectable locoregional disease or metastatic disease. Patients who are unwilling to undergo surgery or external beamradiation are also eligible.

3. Patients with PDTC must have at least one target lesion by RECIST v1.1. This is not a requirement forATC patients.

4. Total bilirubin ≤1.5 x upper limit of normal (ULN). Total bilirubin≤ 3 x ULN for patients with Gilbert's syndrome. AST (SGOT)/ALT (SGPT) ≤2.5 x ULN, (5 x ULN for patients withconcurrent liver metastases). Serum creatinine ≤ within 1.5 x ULN. ANC ≥ 1.0 x 10 ^9/L; PLT ≥ 100 x10^9/L.

5. For patients receiving therapeutic anticoagulation: stable anticoagulant regimen andstable INR during the 28 days immediately preceding initiation of study treatment

6. Subjects must be willing to undergo tumor biopsy after treatment with atezolizumab, unless in the opinion of the treating physician, a biopsy is not feasible or safe.

7. ECOG PS</=2

8. Age >/= 18years.

9. Age and Reproductive Status a) Males and Females, >/=18 years. Women of childbearing potential (WOCBP)* must have a negative serum or urine pregnancy test within 14 days prior to the start of study drug and must use effective contraceptives throughout the duration of the study. Males who are sexually active with WOCBP must agree to use effective contraception throughout the duration of the study. Azoospermic males and WOCBP who are continuously not heterosexually active are exempt from contraceptive requirements.
*A Women of childbearing potential (WOCBP) is defined as any female who has experienced menarche and who has not undergone surgical sterilization (hysterectomy or bilateral oophorectomy) and is not postmenopausal. Menopause is defined as 12 months of amenorrhea in a woman over age 45 years in the absence of other biological or physiological causes.

10. Negative hepatitis B surface antigen (HBsAg) test at screening

11. Ability to provide informed consent.

12. **ADDITIONAL INCLUSION CRITERIA FOR BRAF MUTATION (COHORT 1):** Patients with a BRAFV600E mutation being considered for the triplet combination (vemurafenib + cobimetinib + atezolizumab) must meet the following endorgan function criteria: ANC≥ 1.5 × 10^9/L without granulocyte colony-stimulating factor support, WBC count ≥ 2.5 × 10^9/L, Lymphocyte count ≥ 0.5 × 10^9/L, Platelet count ≥ 100 × 10^9/L without transfusion,

© 2024 American Medical Association. All rights reserved.

Hemoglobin ≥ 9.0 g/L without transfusion. Serum albumin ≥ 2.5 g/L, Total bilirubin ≤ 1.5 × ULN, AST and ALT ≤ 2.0 × ULN, Alkaline phosphatase (ALP) ≤ 2.5 × ULN or, for patients with documented liver or bone metastases, ALP ≤ 5 × ULN, Serum creatinine ≤ 1.5 × ULN or creatinine clearance (CrCl) ≥ 40 mL/min on the basis of measured CrCl from a 24 -hour urine collection or Cockcroft-Gault glomerular filtration rate estimation:
CrCl = ((140 − age) / (serum creatinine in mg/dL)) × (weight in kg) (× 0.85 if female)/ 72).
Patients with BRAF mutation may be screened for eligibility in cohorts 2, 3, or 4 (in this order of preference) if they do not meet the entry criteria for cohort 1.

<u>Exclusion Criteria</u>

1. Subjects with an active, known or suspected autoimmune disease. Subjects with type I diabetes mellitus on stable insulin regimen, hypothyroidism only requiring hormone replacement, skin disorders (such as vitiligo, psoriasis, or alopecia) not requiring systemic treatment, or conditions not expected to recur in the absence of an external trigger are permitted to enroll.

2. For patients not receiving therapeutic anticoagulation: INR or aPTT >1.5 x ULN within 28 days prior to initiation of study treatment

3. Prior treatment with anti-PD-1, or anti-PD-L1 therapeutic antibody or pathway targeting agents. Patients who have received prior treatment with anti-CTLA-4 may be enrolled, provided the following requirements are met:
Minimum of 12 weeks from the first dose of anti-CTLA-4 and > 6 weeks from the last dose
No history of severe immune-related adverse effects from anti-CTLA 4 (NCI CTCAE Grade 3 and 4)

4. Known clinically significant liver disease, including active viral, alcoholic, or other hepatitis; cirrhosis; fatty liver; and inherited liver disease

5. History of HIV infection or active hepatitis B (chronic or acute) or hepatitis C infection
Patients with past or resolved hepatitis B infection (defined as having a negative hepatitis B surface antigen[HBsAg] test and apositive anti-HBc[antibody to hepatitis B core antigen] antibody test) are eligible. However, patients with past or resolved HBV should be monitored for reactivation by a specialist.
Patients positive for hepatitis C virus (HCV) antibody are eligible only if polymerase chain reaction (PCR) is negative for HCV RNA.. *Current treatment with anti-viral therapy for HBV.*

6. Pregnant or lactating women. All pre-menopausal women being screened must have a negative serum pregnancy test within 14 days prior to commencement of dosing. Women of non-childbearing potential may be included if they are either surgically sterile or have been postmenopausal for >/= 1 year. Fertile men and women must use an effective method of contraception during treatment and for at least 6 months after completion of treatment as directed by their physician.

7. Untreated brain metastases.

8. Chemotherapy within 21 days of enrollment with the exception of paclitaxel or nab-

© 2024 American Medical Association. All rights reserved.

paclitaxel (Abraxane). Patients who have received one course of these agents prior to study entry are eligible. (One course of weekly paclitaxel or nab-paclitaxel is 3 doses. One

© 2024 American Medical Association. All rights reserved.

course of every 3 week dosing of paclitaxel or nab-paclitaxel is 1 dose). Patients who have received prior radiosensitizing chemotherapy are eligible.

9. The use of corticosteroids is not allowed for 10 days prior to initiation of atezolizumab except patients who are taking steroids for physiological replacement. Inhaled or topical steroids, and adrenal replacement steroid doses are permitted in the absence of active autoimmune disease. This does not apply to patients receiving steroids as pre-medications for paclitaxel administration.

10. Grade ≥ 2 uncontrolled hypertension (patients with a history of hypertension controlled with anti-hypertensive medication to Grade ≤ 1 are eligible)

11. Cobi Specific Exclusion Criteria

- Prior treatment with a MEK inhibitor
- Poorly controlled hypertension, defined as sustained, uncontrolled, non-episodic baseline hypertension (blood pressure [BP] consistently above 159/99 mmHg) despite optimal medical management
- History or presence of an abnormal ECG that is deemed clinically significant by the investigator, including complete left bundle branch block, second- or third-degree atrioventricular heart block, or evidence of prior myocardial infarction
- Known allergy or hypersensitivity to any component of the cobimetinib formulation
- History of malabsorption syndrome or other condition that would interfere with enteral absorption or results in the inability or unwillingness to swallow pills
- History or evidence of inherited bleeding diathesis or significant coagulopathy at risk for bleeding
- Any Grade ≥ 3 hemorrhage or bleeding event within 28 days prior to initiation of study treatment
- History of stroke, reversible ischemic neurological defect, or transient ischemic attack within 6 months prior to initiation of study treatment
- Consumption of foods, supplements, or drugs that are strong or moderate CYP3A4 enzyme inducers or inhibitors (e.g., St. John's wort, hyperforin, grapefruit juice) within 2 weeks prior to initiation of study treatment

12. ADDITIONAL EXCLUSION CRITERIA FOR non-BRAF/non-RAS MUTATION (COHORT 3):
a. Patients with clinically significant hemoptysis or tumor bleeding within two weeks prior to first dose of targeted therapy.
b. Patients with suspected tracheal or esophageal invasion are excluded from cohort3 due to the high risk of trachealesophageal fistula.
Patients excluded from cohort 3 may be enrolled on taxane + atezolizumab cohort (cohort 4).

13. **ADDITIONAL EXCLUSION CRITERIA FOR COHORTS 1 and 2:** Ocular Exclusion Criteria for cobimetinib containing cohorts—cohorts 1 and 2. (However, these patients may be assigned other cohorts if they do not meet the ocular exclusion criteria):
History of or evidence of retinal pathology on ophthalmologic examination that is considered a risk factor for neurosensory retinal detachment, central serous chorioretinopathy, retinal vein occlusion (RVO), or neovascular macular degeneration Patients will be excluded from participation in cohorts 1 and 2 if they currently are known to have any of the following risk

© 2024 American Medical Association. All rights reserved.

factors for RVO, unless a retinal specialist has determined that the risk of retinal detachment is low:

a History of serous retinopathy
b. History of retinal vein occlusion
c. History of ongoing serous retinopathy or RVO at baseline

14. **ADDITIONAL EXCLUSION CRITERIA FOR PATIENTS IN COHORT 1 (vemurafenib+cobimetinib+atezolizumab):** Cardiac Exclusion Criteria: History of clinically significant cardiac dysfunction, including the following:

a. Mean (average of triplicate measurements) QTc interval corrected using Fridericia's method (QTcF) >= 480 ms at screening, or uncorrectable abnormalities in serum electrolytes (sodium, potassium, calcium, magnesium, and phosphorus)

15. **ADDITIONAL EXCLUSION CRITERIA FOR PATIENTS IN COHORTS 1 and 2 (cobimetinib-containing cohorts):** Cardiac Exclusion Criteria:

a. Unstable angina, or new-onset angina within 3 months prior to initiation of study treatment
b. Symptomatic congestive heart failure, defined as New York Heart Association Class II or higher
c. Myocardial infarction within 3 months prior to initiation of study treatment
d. Left ventricular ejection fraction below the institutional lower limit of normal or below 50%, whichever is lower

eMethods 2. Doses of study drugs

- Cohort 1, BRAF mutant: vemurafenib 960mg bid (day 1-21) + cobimetinib 60mg qday (day 1-21) run-inbefore starting atezolizumab. Vemurafenib will be dose reduced to 720mg bid (at cycle 1 day 21) and cobimetinib 60mg will be taken on days 1-21. Atezolizumab 840 mg IV on Day 1 and Day 15 in a 28-day cycle, will be started on Cycle 1, Day 1. Patients who have > grade 3 LFTs (AST, ALT or total bilirubin) will not receive atezolizumab but may continue on vemurafenib + cobimetinib with dose reduction. If after dose reductions, the LFTs are below grade 3, patient may start atezolizumab. See figure 2.

- Cohort 2, RAS, NF1, or NF2 mutant, including patients with tumors with MAPK activating mutations at or above MEK: cobimetinib 60 mg orally on Days 1−21 plus atezolizumab 840 mg IV on Day 1 and Day 15 in a 28-day cycle.

- Cohort 3, Non-BRAF/non-RAS mutant: atezolizumab 1200 mg IV every 21 days plus bevacizumab 15 mg/kg IV every 21 days in a 21-day cycle.

- Cohort 4, Taxane + atezolizumab: Nab-paclitaxel 100mg/m2 on days 1, 8, and 15. Atezolizumab 1200mg IV every 21 days. Paclitaxel may be substituted for nab-paclitaxel but nab-paclitaxel is preferred. The dose of paclitaxel is 175mg/m2 every 21 days. Any patient assigned to cohort 4 is eligible to receive nab-paclitaxel at a local facility.

© 2024 American Medical Association. All rights reserved.

eMethods 3. Patient instructions for alternative drug administration of vemurafenib

Vemurafenib may be crushed if you are unable to swallow the pills whole, however, *vemurafenib must never be diluted in water*. Please follow the instructions below if you are unable to swallow vemurafenib pills whole.

Instruction A: oral administration for patients who are able to swallow pureed foods

1. Always wear a face mask and gloves when handling chemotherapy
2. Place approximately 2-3 ounces of baby food in a zipper bag
3. Place vemurafenib tablets in a pill crusher and crush them into a fine powder. Do not crush more than 2 tablets at a time in order to obtain a fine powder.
4. Add the crushed vemurafenib to baby food in the zipper bag. Zip the bag and knead.
5. Cut a corner of the zipper bag and squeeze the entire contents of the baby food/vemurafenib mix into your mouth.

Instruction B: for patients administering vemurafenib by the PEG tube:

1. Always wear a face mask and gloves when handling chemotherapy
2. Place approximately 2-3 ounces of baby food in a zipper bag (about half the baby food)
3. Place vemurafenib tablets in a pill crusher and crush them into a fine powder. Do not crush more than 2 tablets at a time in order to obtain a fine powder.
4. Add the crushed vemurafenib to baby food in the zipper bag. Zip the bag and knead.
5. Cut a corner of the zipper bag.
6. Remove the plunger from a 60cc syringe (with cap on) squeeze the entire contents of the baby food/vemurafenib mix into it.
7. Replace the plunger, point the tip of syringe upward to allow for the baby food mix to move away from the tip. Remove the syringe cap and remove the air in the syringe.
8. Administer the baby food mixture in the PEG

tube. Rinse Steps:

1. After administration of the baby food mixture from the 60 cc syringe, close the tip port of the syringe with a cap and remove the plunger
2. Pour the remaining baby food into the syringe.
3. Replace the plunger, point the tip of syringe upward to allow for the baby food mix to move away from the tip. Remove the syringe cap and remove the air in the syringe.
4. Administer the baby food in the PEG tube
5. Flush the PEG tube with 60 mL or more of water.

© 2024 American Medical Association. All rights reserved.

eMethods 4. Futility analysis

All patients who receive at least 1 dose of study drug (vemurafenib, cobimetinib, bevacizumab, atezolizumab) in any cohort will be included in futility monitoring. Cohorts 1-3 will be included in one futility monitoring plan. We will monitor time to death continuously using a Bayesian method (Thall et al, 2005, Clinical Trials, 2:467-478) that assumes the median time to death follows an Inverse Gamma

distribution and that the individual death times follow an exponential distribution. Let mE represent the median in the newly accrued (experimental) patients and let mS represent the historical median to be used for comparison. Based on somewhat limited historical data the median death time is about 5 months. For the historical median we specify an Inverse Gamma prior with alpha = 8.25 and beta = 36.25 (which has mean = 5.0 and SD = 2.0). For the experimental median we specify an Inverse Gamma prior with alpha

= 2.25 and beta = 6.25 (which has a mean of 5.0 and SD = 10.0). The maximum sample size is 36 and the expected accrual rate is 1.1 per month (trial duration = 33 months). There will be 3 months of post-accrual follow-up. Monitoring will be continuous with the rule to stop if Pr(mE > mS + 4 | data) < 0.025. The operating characteristics were computed using One-Arm Time-to-Event Simulator version 3.0.2. The rule will be implemented using the Department of Biostatistics Clinical Trial Conduct website.

Operating Characteristics

Median (m)	PET (%)	Ave # Pts	Ave Dur. (m)
3.0	100%	7.4	9.7
5.0	85%	17.0	18.4
7.0	34%	27.8	27.6
9.0	15%	31.8	31.3
110	8%	33.6	33.0
13.0	5%	34.5	33.5

Where PET = probability of early termination Dur. = duration of trial

A separate futility monitoring plan will be used for cohort 4. We will monitor time to death continuously using a Bayesian method (Thall et al, 2005, Clinical Trials, 2:467 -

478) that assumes the median time to death follows an Inverse Gamma distribution and that the individual death times follow an exponential distribution. Let mE represent the median in the newly accrued (experimental) patients and let mS represent the historical median to be used for comparison. Based on somewhat limited historical data the median death time is about 3 months. For the historical median we specify an Inverse Gamma prior with alpha = 4.25 and beta = 9.75 (which has mean = 3.0 and SD = 2.0). For the experimental median we specify an Inverse Gamma prior with alpha = 2.09 and beta = 3.27 (which has a mean of 3.0 and SD = 10.0). The maximum sample size is 36 and the expected accrual rate is 0.5 per month (trial duration = 28 months). There will be 3 months of post-accrual follow-up. Monitoring will be continuous with the rule to stop if Pr(mE > mS + 2 | data) < 0.025. The

© 2024 American Medical Association. All rights reserved.

operating characteristics were computed using One-Arm Time-to-Event Simulator version 3.0.2. The rule will be implemented using the Department of Biostatistics Clinical Trial Conduct website.

Operating Characteristics

Median (m)	PET (%)	Ave # Pts	Ave Dur. (m)
1.0	100%	3.6	10.2
2.0	80%	7.5	18.0
3.0	33%	11.4	24.2
4.0	17%	12.5	26.5
5.0	8%	13.2	27.8
6.0	5%	13.5	28.0

Where PET = probability of early termination Dur. = duration of trial

© 2024 American Medical Association. All rights reserved.

eFigure 1. Vemurafenib, cobimetinib, atezolizumab (cohort 1) run-in and dosing

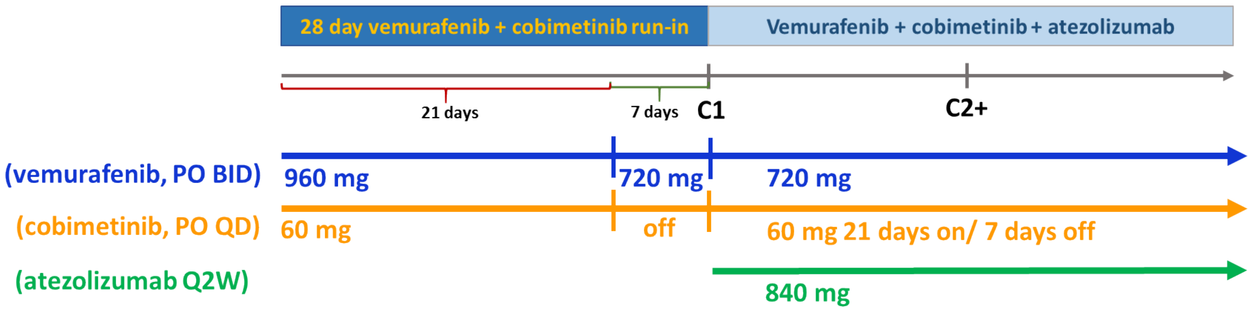

© 2024 American Medical Association. All rights reserved.

eFigure 2. Treatment allocation. Cohort 1 (BRAF) included patients with BRAF V600E mutation and were treated with vemurafenib, cobimetinib, atezolizumab. Cohort 2 (MEK) included patients with RAS or NF mutations and were treated with cobimetinib, atezolizumab. Cohort 3 (VEGF) included patients that did not have BRAF, RAS or NF mutations and these were treated with bevacizumab, atezolizumab. Cohort 4 (CHEMO) included patients who did not qualify for cohorts 1, 2, or 3 and these were treated with paclitaxel or nab-paclitaxel, atezolizumab. The primary analysis included only ATC patients assigned to cohort 1-3.

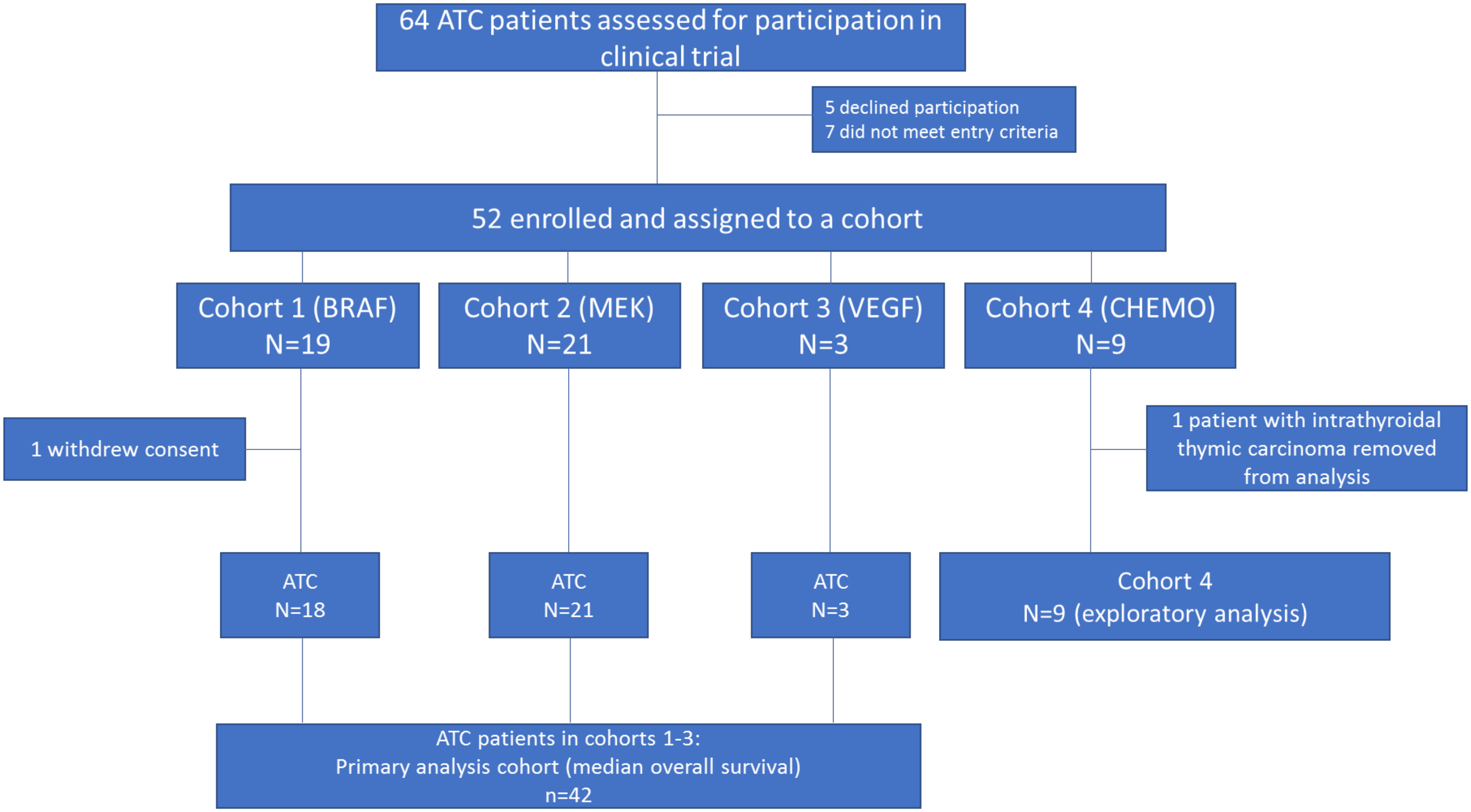

© 2024 American Medical Association. All rights reserved.

eFigure 3. Swimmer plot showing duration of treatment on study, the time alive since starting the study drugs, and RECIST v1.1 response in ATC patients on cohorts 1-3. Patients who had their primary tumor removed are noted on the plot. If the primary tumor was recorded as a target lesion,

© 2024 American Medical Association. All rights reserved.

the response became unevaluable and was not recorded further on the plot.

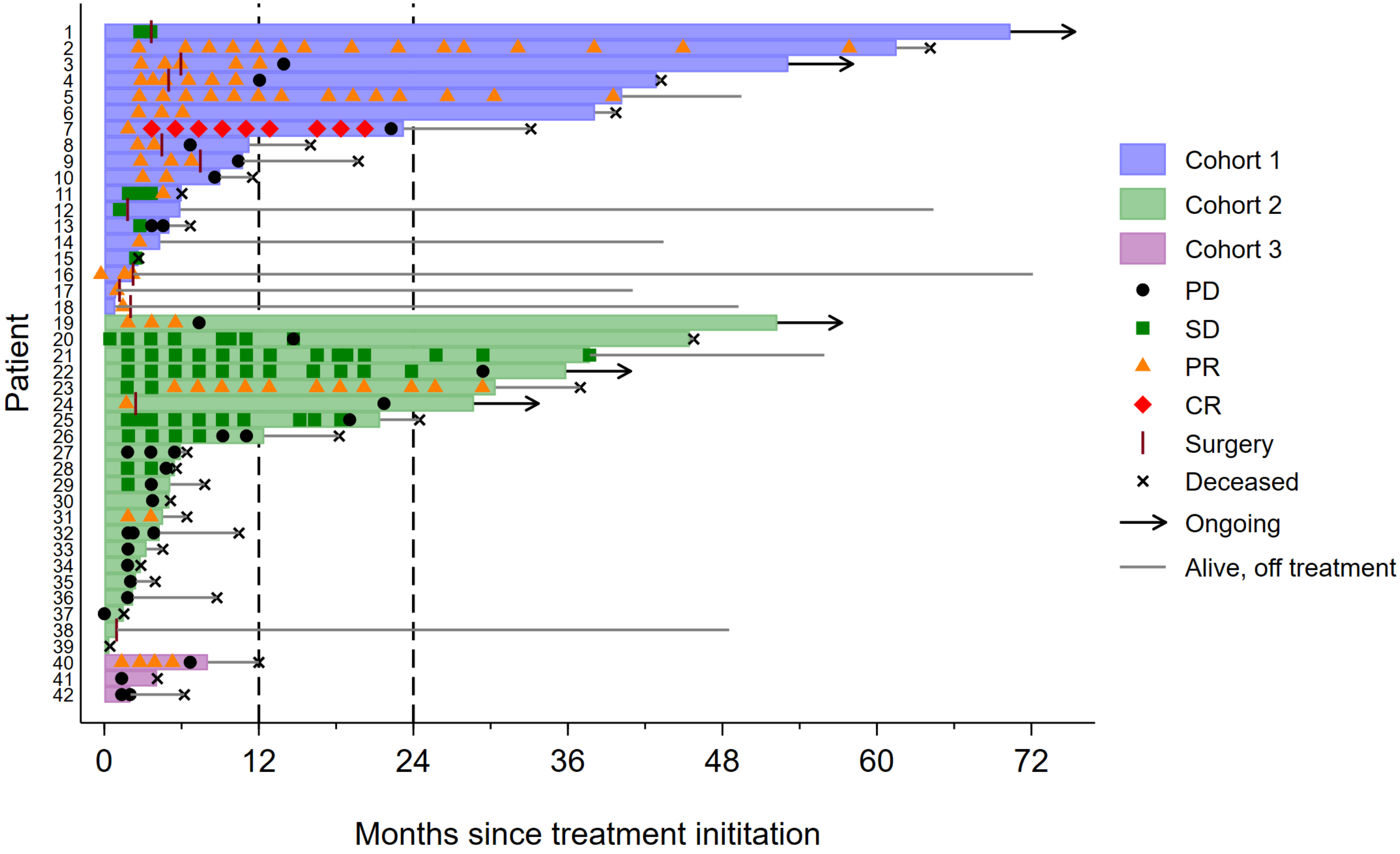

© 2024 American Medical Association. All rights reserved.

© 2024 American Medical Association. All rights reserved.

eTable. Adverse Events amongst all cohorts (identified in >15% of patients)

Adverse Event Name	Grade 1/2	Grade 3/4	All grades
Lymphopenia, n (%)	20 (33.9)	15 (25.4)	35 (59.3)
Fatigue, n (%)	28 (47.5)	2 (3.4)	30 (50.9)
Diarrhea, n (%)	24 (40.7)	5 (8.5)	29 (49.2)
Elevated ALT, n (%)	20 (33.9)	5 (8.5)	25 (42.4)
Rash acneiform, n (%)	25 (42.4)	0	25 (42.4)
Anemia, n (%)	19 (32.2)	4 (6.8)	23 (39)
Rash maculo-papular, n (%)	16 (27.1)	6 (10.2)	22 (37.3)
Proteinuria, n (%)	19 (32.2)	2 (3.4)	21 (35.6)
Skin and subcutaneous tissue disorder, n (%)	18 (30.5)	2 (3.4)	20 (33.9)
Elevated AST, n (%)	16 (27.1)	3 (5.1)	19 (32.2)
Nausea, n (%)	18 (30.5)	0	18 (30.5)
CPK increased, n (%)	14 (23.7)	3 (5.1)	17 (28.8)
Creatinine increased, n (%)	17 (28.8)	0	17 (28.8)
Hyperglycemia, n (%)	14 (23.7)	2 (3.4)	16 (27.1)
Pruritus, n (%)	15 (25.4)	0	15 (25.4)
Alopecia, n (%)	14 (23.7)	0	14 (23.7)
Generalized muscle weakness, n (%)	12 (20.3)	2 (3.4)	14 (23.7)
Elevated Alkaline phosphatase, n (%)	13 (22)	0	13 (22)
Dyspnea, n (%)	10 (16.9)	3 (5.1)	13 (22)
Weight loss, n (%)	13 (22)	0	13 (22)
Edema limbs, n (%)	11 (18.6)	0	11 (18.6)
Fever, n (%)	10 (16.9)	1 (1.7)	11 (18.6)
Hypertension, n (%)	9 (15.3)	2 (3.4)	11 (18.6)
Dysgeusia, n (%)	10 (16.9)	0	10 (16.9)
Vomiting, n (%)	9 (15.3)	1 (1.7)	10 (16.9)
Leukopenia, n (%)	9 (15.3)	1 (1.7)	10 (16.9)
Anorexia, n (%)	9 (15.3)	0	9 (15.3)
Dry skin, n (%)	9 (15.3)	0	9 (15.3)
Hyponatremia, n (%)	7 (11.9)	2 (3.4)	9 (15.3)

Adverse events listed were considered possibly, probably and definitely related seen in 15% or more patients. The highest adverse event was counted once per patient.

© 2024 American Medical Association. All rights reserved.
