# Supplementary material for: Anti–Programmed Death Ligand 1 Plus Targeted Therapy in Anaplastic Thyroid Carcinoma: A Nonrandomized Clinical Trial
Source: JAMA Oncol. 2024 Oct 24;10(12):1672–80. doi: 10.1001/jamaoncol.2024.4729 (PMC11581602; doi:10.1001/jamaoncol.2024.4729)
Supplement: Supplement 3. — The members of The Rare Tumor Initiative Team [file jamaoncol-e244729-s003.pdf]

Supplemental Online Content: Nonauthor Collaborators
*First name, last name, and suffix (if applicable) are required and will appear in PubMed.

The Rare Tumor Initiative Team								
***First Name and Middle Initial(s)**	***Last Name**	***Suffix (eg, Jr, III)**	**Academic Degrees**	**Institution**	**Location (city, state/province, country)**	**Role or Contribution, eg, chair, principal investigator**	**Group (if more than 1 Group listed in the byline) and/or Subgroup (eg, Steering Committee)**	**Actual Role**
Ahmed N	Alrawi		BS, MS	The University of Texas MD Anderson Cancer Center	Houston, TX	Research Data Specialist - APOLLO Platform		Biospecimen Acquisition
Claudio A	Arrechedera		MD	The University of Texas MD Anderson Cancer Center	Houston, TX	Research Scientist, Translational Molecular Pathology		mIF Analysis
Kimberly S	Ayers		MS	The University of Texas MD Anderson Cancer Center	Houston, TX	Clinical Studies Coordinator, Lymphoma-Myeloma		Patient Consent
Caludia A	Bedoya		BS,MS	The University of Texas MD Anderson Cancer Center	Houston, TX	Coordinator, Clincal Res. Program, Gyn Onc & Reprod. Med		Patient Consent
Elizabeth	Burton			The University of Texas MD Anderson Cancer Center	Houston, TX	Rare Tumor Initiative Co-Director		Project Leader
Connie A	Chon		BS	The University of Texas MD Anderson Cancer Center	Houston, TX	HER Sys Analyst, Oncology & Research IS		Data Analysis
Randy A	Chu		BS,MS	The University of Texas MD Anderson Cancer Center	Houston, TX	Program Manager, Rare Tumor Initiative		Sample and Data Management
Shadarra D	Crosby		MHA, MBA	The University of Texas MD Anderson Cancer Center	Houston, TX	Research Data Specialist - APOLLO Platform		Biospecimen Acquisition
Jonathan	Do		BS	The University of Texas MD Anderson Cancer Center	Houston, TX	Sr. Systems Analyst, Enterprise Dev & Integration		Data Analysis
Cibelle FP	Lima		PhD	The University of Texas MD Anderson Cancer Center	Houston, TX	Research Investigator, Translational Molecular Pathology		mIF analysis
Fu	Szu-Chin		MS, PhD	The University of Texas MD Anderson Cancer Center	Houston, TX	Compuatational Scientist, Genomic Medicine		Data Analysis

Supplemental Online Content: Nonauthor Collaborators
*First name, last name, and suffix (if applicable) are required and will appear in PubMed.

*First Name and Middle Initial(s)	*Last Name	*Suffix (eg, Jr, III)	Academic Degrees	Institution	Location (city, state/province, country)	Role or Contribution, eg, chair, principal investigator	Group (if more than 1 Group listed in the byline) and/or Subgroup (eg, Steering Committee)	Actual Role
Andy	Futreal		PhD	The University of Texas MD Anderson Cancer Center	Houston, TX	Co-Leader Rare Tumor Initiative		Project Leader
Ana L	Garcia		BS	The University of Texas MD Anderson Cancer Center	Houston, TX	Sr. Research Histo Tech, Translational Molecular Pathogy		Histology
Celia	Garica-Prieto		BS, MS, PhD	The University of Texas MD Anderson Cancer Center	Houston, TX	Co-Director Rare Tumor Initiative		Project Management
Swati	Gite		MD	The University of Texas MD Anderson Cancer Center	Houston, TX	Research Investigator, Translational Molecular Pathology		Pathology
Curtis	Gumbs		BS,MS	The University of Texas MD Anderson Cancer Center	Houston, TX	Scientific Manager, Genomic Medicine		Genomic Sequencing
Kristin J	Hargraves		BSN,RN	The University of Texas MD Anderson Cancer Center	Houston, TX	Research Nurse Manager, Translational Molecular Pathology		Patient Consent
Meng	He		PhD	The University of Texas MD Anderson Cancer Center	Houston, TX	Research Investigator, Translational Molecular Pathology		mIF Analysis
Chacha	Horombe		MS	The University of Texas MD Anderson Cancer Center	Houston, TX	Sr. Data Integration Developer, Enterprise Dev & Integration		Data Management
Heladio P	Ibarguen		BS,MS	The University of Texas MD Anderson Cancer Center	Houston, TX	Sr. Research Asst., Translational Molecular Pathology		Biospecimen Acquisition
Stacy	Jackson		BS	The University of Texas MD Anderson Cancer Center	Houston, TX	Clinical Studies Coordinator, Translational Molecular Pathology		Patient Consent

*First name, last name, and suffix (if applicable) are required and will appear in PubMed.

*First Name and Middle Initial(s)	*Last Name	*Suffix (eg, Jr, III)	Academic Degrees	Institution	Location (city, state/province, country)	Role or Contribution, eg, chair, principal investigator	Group (if more than 1 Group listed in the byline) and/or Subgroup (eg, Steering Committee)	Actual Role
Jeena J	Jacob		BS,MHA	The University of Texas MD Anderson Cancer Center	Houston, TX	Clinical Studies Coordinator, Translational Molecular Pathology		Patient Consent
Mei	Jiang		BS	The University of Texas MD Anderson Cancer Center	Houston, TX	Laboratory Manager, Translational Molecular Pathology		Biospecimen Acquisition
Isha	Khanduri		MD	The University of Texas MD Anderson Cancer Center	Houston, TX	Research Investigator, Translational Molecular Pathology		Pathology
Walter K	Kinyua		BS	The University of Texas MD Anderson Cancer Center	Houston, TX	Data Integration Developer, Enterprise Dev & Integration		Data Management
Mark	Knafl		MS	The University of Texas MD Anderson Cancer Center	Houston, TX	Computational Data Scientist		Data Analysis
Wenhua	Lang		MS	The University of Texas MD Anderson Cancer Center	Houston, TX	Research Laboratory Manager, Translational Molecular Pathology		Biospecimen Processing
Latasha D	Little		BS,MS	The University of Texas MD Anderson Cancer Center	Houston, TX	Laboratory Manager, Genomic Medicine		Genomic Sequencing
Wei	Lu		MD, PhD	The University of Texas MD Anderson Cancer Center	Houston, TX	Principal Research Scientist, Translational Molecular Pathology		Biospecimen Processing
Saradhi	Mallampati		PhD	The University of Texas MD Anderson Cancer Center	Houston, TX	Department of Pathology/Molecular Genetic Pathology		Pathology
Mary GT	Mendoza		BS	The University of Texas MD Anderson Cancer Center	Houston, TX	Laboratory Coordinator, Translational Molecular Pathology		Histology

Supplemental Online Content: Nonauthor Collaborators
*First name, last name, and suffix (if applicable) are required and will appear in PubMed.

*First Name and Middle Initial(s)	*Last Name	*Suffix (eg, Jr, III)	Academic Degrees	Institution	Location (city, state/province, country)	Role or Contribution, eg, chair, principal investigator	Group (if more than 1 Group listed in the byline) and/or Subgroup (eg, Steering Committee)	Actual Role
Funda	Meric-Bernstam		MD	The University of Texas MD Anderson Cancer Center	Houston, TX	Co-Leader Rare Tumor Initiative		Project Leader
Mohammad M	Mohammad		BS,MS	The University of Texas MD Anderson Cancer Center	Houston, TX	Assoc. Dir. Laboratory Operations, Institute of Personalized Cancer Therapy		Genomic Sequencing
Mario LM	Piubelli		MD	The University of Texas MD Anderson Cancer Center	Houston, TX	Postdoctoral Fellow, Translational Molecular Pathology		mIF Analysis
Sabitha	Prabhakaran		PhD	The University of Texas MD Anderson Cancer Center	Houston, TX	Data Manager - Rare Tumor Initiative		Project Leader
Kenna R	Shaw		PhD	The University of Texas MD Anderson Cancer Center	Houston, TX	Director Rare tumor Initiative		Project Leader
Xiaofei	Song		PhD	The University of Texas MD Anderson Cancer Center	Houston, TX	Computational Scientist, Biostatics and Bioinformatics		Data Analysis
Sandesh	Subramanya		MBA, PhD	The University of Texas MD Anderson Cancer Center	Houston, TX	Scientific Manager - APOLLO Platform		Sample and Data Management
Baohua	Sun		MD, PhD	The University of Texas MD Anderson Cancer Center	Houston, TX	Sr. Research Scientist Translational Molecular Pathology		mIF Analysis
Shumaila	Virani		MSHS	The University of Texas MD Anderson Cancer Center	Houston, TX	Laboratory Coordinator, Institute of PersonalizedCancer Therapy		Genomic Sequencing
Wanlin	Wang		MS	The University of Texas MD Anderson Cancer Center	Houston, TX	Applications Systems Analyst, Enterprise Dev & Integration		Data Management
Ignacio	Wistuba		MD	The University of Texas MD Anderson Cancer Center	Houston, TX	Co-Leader Rare Tumor Initiative		Project Leader
Scott E	Woodman		MD, PhD	The University of Texas MD Anderson Cancer Center	Houston, TX	Associate Professor, Genomic Medicine		Data Analysis

Supplemental Online Content: Nonauthor Collaborators

*First name, last name, and suffix (if applicable) are required and will appear in PubMed.

*First Name and Middle Initial(s)	*Last Name	*Suffix (eg, Jr, III)	Academic Degrees	Institution	Location (city, state/province, country)	Role or Contribution, eg, chair, principal investigator	Group (if more than 1 Group listed in the byline) and/or Subgroup (eg, Steering Committee)	Actual Role
Mingchu	Xu		PhD	The University of Texas MD Anderson Cancer Center	Houston, TX	Machine Learning, Data Analytics, Bioinformatics		Data Analysis
Jianhua	Zhang		MS, PhD	The University of Texas MD Anderson Cancer Center	Houston, TX	Exec. Dir. Comp Genomics, Genomic Medicine		Genomic Sequencing
Qingxiu C	Zhang		PhD	The University of Texas MD Anderson Cancer Center	Houston, TX	Sr. Research Scientist, IPCT		Data Analysis
Shanyu	Zhang		MS	The University of Texas MD Anderson Cancer Center	Houston, TX	Data Analyst, Translational Molecular Pathology		Data Analysis
