# Supplementary material for: Anti–Programmed Death Ligand 1 Plus Targeted Therapy in Anaplastic Thyroid Carcinoma: A Nonrandomized Clinical Trial
Source: JAMA Oncol. 2024 Oct 24;10(12):1672–80. doi: 10.1001/jamaoncol.2024.4729 (PMC11581602; doi:10.1001/jamaoncol.2024.4729)
Supplement: Supplement 4. — Data Sharing Statement [file jamaoncol-e244729-s004.pdf]

Data Sharing Statement

Cabanillas. Anti–Programmed Death Ligand 1 Plus Targeted Therapy in Anaplastic Thyroid Carcinoma. *JAMA Oncol*. Published October 24, 2024. doi:10.1001/jamaoncol.2024.4729

Data
Additional Information: NCT03181100
Data available: No

Additional Information
Explanation for why data not available: We may consider sharing de-identified data upon reasonable request
